# Comparative evolutionary analyses of peste des petits ruminants virus genetic lineages

**DOI:** 10.1093/ve/veae012

**Published:** 2024-03-06

**Authors:** Maxime Courcelle, Habib Salami, Kadidia Tounkara, Modou Moustapha Lo, Aminata Ba, Mariame Diop, Mamadou Niang, Cheick Abou Kounta Sidibe, Amadou Sery, Marthin Dakouo, Lanceï Kaba, Youssouf Sidime, Mohamed Keyra, Alpha Oumar Sily Diallo, Ahmed Bezeid El Mamy, Ahmed Salem El Arbi, Yahya Barry, Ekaterina Isselmou, Habiboullah Habiboullah, Baba Doumbia, Mohamed Baba Gueya, Joseph Awuni, Theophilus Odoom, Patrick Tetteh Ababio, Daniel Nana Yaw TawiahYingar, Caroline Coste, Samia Guendouz, Olivier Kwiatek, Geneviève Libeau, Arnaud Bataille

**Affiliations:** ASTRE, University of Montpellier, CIRAD, INRAE, Montpellier F-34398, France; CIRAD, UMR ASTRE, Montpellier F-34398, France; ASTRE, University of Montpellier, CIRAD, INRAE, Montpellier F-34398, France; CIRAD, UMR ASTRE, Montpellier F-34398, France; Institut Sénégalais de Recherches Agricoles, Laboratoire National d’Elevage et de Recherches Vétérinaires (LNERV), Dakar-Hann BP 2057, Sénégal; ASTRE, University of Montpellier, CIRAD, INRAE, Montpellier F-34398, France; CIRAD, UMR ASTRE, Montpellier F-34398, France; Laboratoire Central Vétérinaire (LCV), Bamako BP 2295, Mali; Institut Sénégalais de Recherches Agricoles, Laboratoire National d’Elevage et de Recherches Vétérinaires (LNERV), Dakar-Hann BP 2057, Sénégal; Institut Sénégalais de Recherches Agricoles, Laboratoire National d’Elevage et de Recherches Vétérinaires (LNERV), Dakar-Hann BP 2057, Sénégal; Institut Sénégalais de Recherches Agricoles, Laboratoire National d’Elevage et de Recherches Vétérinaires (LNERV), Dakar-Hann BP 2057, Sénégal; Laboratoire Central Vétérinaire (LCV), Bamako BP 2295, Mali; Laboratoire Central Vétérinaire (LCV), Bamako BP 2295, Mali; Laboratoire Central Vétérinaire (LCV), Bamako BP 2295, Mali; Laboratoire Central Vétérinaire (LCV), Bamako BP 2295, Mali; Institut Supérieur des Sciences et de Médecine Vétérinaire, Dalaba BP 2201, Guinea; Institut Supérieur des Sciences et de Médecine Vétérinaire, Dalaba BP 2201, Guinea; Institut Supérieur des Sciences et de Médecine Vétérinaire, Dalaba BP 2201, Guinea; Institut Supérieur des Sciences et de Médecine Vétérinaire, Dalaba BP 2201, Guinea; Office National de Recherches et de Développement de l’Elevage (ONARDEL), Nouakchott BP 167, Mauritania; Office National de Recherches et de Développement de l’Elevage (ONARDEL), Nouakchott BP 167, Mauritania; Office National de Recherches et de Développement de l’Elevage (ONARDEL), Nouakchott BP 167, Mauritania; Office National de Recherches et de Développement de l’Elevage (ONARDEL), Nouakchott BP 167, Mauritania; Office National de Recherches et de Développement de l’Elevage (ONARDEL), Nouakchott BP 167, Mauritania; Office National de Recherches et de Développement de l’Elevage (ONARDEL), Nouakchott BP 167, Mauritania; Office National de Recherches et de Développement de l’Elevage (ONARDEL), Nouakchott BP 167, Mauritania; Accra Veterinary Laboratory, Veterinary Services Directorate, Accra M161, Ghana; Accra Veterinary Laboratory, Veterinary Services Directorate, Accra M161, Ghana; Accra Veterinary Laboratory, Veterinary Services Directorate, Accra M161, Ghana; Accra Veterinary Laboratory, Veterinary Services Directorate, Accra M161, Ghana; ASTRE, University of Montpellier, CIRAD, INRAE, Montpellier F-34398, France; CIRAD, UMR ASTRE, Montpellier F-34398, France; ASTRE, University of Montpellier, CIRAD, INRAE, Montpellier F-34398, France; CIRAD, UMR ASTRE, Montpellier F-34398, France; ASTRE, University of Montpellier, CIRAD, INRAE, Montpellier F-34398, France; CIRAD, UMR ASTRE, Montpellier F-34398, France; ASTRE, University of Montpellier, CIRAD, INRAE, Montpellier F-34398, France; CIRAD, UMR ASTRE, Montpellier F-34398, France; ASTRE, University of Montpellier, CIRAD, INRAE, Montpellier F-34398, France; CIRAD, UMR ASTRE, Montpellier F-34398, France

**Keywords:** *morbillivirus*, phylogenetics, evolution, small ruminants, epizootic, endemic

## Abstract

Peste des petits ruminants virus (PPRV) causes a highly infectious disease affecting mainly goats and sheep in large parts of Africa, Asia, and the Middle East and has an important impact on the global economy and food security. Full genome sequencing of PPRV strains has proved to be critical to increasing our understanding of PPR epidemiology and to inform the ongoing global efforts for its eradication. However, the number of full PPRV genomes published is still limited and with a heavy bias towards recent samples and genetic Lineage IV (LIV), which is only one of the four existing PPRV lineages. Here, we generated genome sequences for twenty-five recent (2010–6) and seven historical (1972–99) PPRV samples, focusing mainly on Lineage II (LII) in West Africa. This provided the first opportunity to compare the evolutionary pressures and history between the globally dominant PPRV genetic LIV and LII, which is endemic in West Africa. Phylogenomic analysis showed that the relationship between PPRV LII strains was complex and supported the extensive transboundary circulation of the virus within West Africa. In contrast, LIV sequences were clearly separated per region, with strains from West and Central Africa branched as a sister clade to all other LIV sequences, suggesting that this lineage also has an African origin. Estimates of the time to the most recent common ancestor place the divergence of modern LII and LIV strains in the 1960s–80s, suggesting that this period was particularly important for the diversification and spread of PPRV globally. Phylogenetic relationships among historical samples from LI, LII, and LIII and with more recent samples point towards a high genetic diversity for all these lineages in Africa until the 1970s–80s and possible bottleneck events shaping PPRV’s evolution during this period. Molecular evolution analyses show that strains belonging to LII and LIV have evolved under different selection pressures. Differences in codon usage and adaptative selection pressures were observed in all viral genes between the two lineages. Our results confirm that comparative genomic analyses can provide new insights into PPRV’s evolutionary history and molecular epidemiology. However, PPRV genome sequencing efforts must be ramped up to increase the resolution of such studies for their use in the development of efficient PPR control and surveillance strategies.

## Introduction

Peste des petits ruminants (PPR) is a highly infectious disease mainly affecting goats and sheep, though it is also found in other wild and domestic Artiodactyls (Baron et al. 2016; Rahman et al. 2020). The disease has been reported in large parts of Africa, Asia, and the Middle East and has an important impact on the global economy and food security, with low-income livestock owners the most affected (Njeumi et al. 2020). PPR can also be detrimental to wildlife conservation efforts, notably through high mortality levels observed in the past in threatened wild ungulates (Fine et al. 2020). The Food and Agriculture Organization of the United Nations (FAO) and the World Organisation for Animal Health (WOAH) have been coordinating a global effort to eradicate PPR by 2030 (WOAH, FAO 2015) through the implementation of a Global Eradication Plan that is now entering phases II and III (FAO, WOAH 2022).

The causal agent of the disease is PPR virus (PPRV), the sole member of the recently renamed *Morbillivirus caprinae* species (genus: *Morbillivirus*, family: *Paramyxoviridae;* (ITCV 2022)). PPRV has a negative sense single-stranded RNA genome of almost 16 kilobases encodingJ for the nucleocapsid (N), phosphoprotein (P), matrix (M), fusion (F), hemagglutinin (H), and polymerase (L) proteins. The open reading frame of the P gene also codes for two non-structural proteins, C and V, through an alternative reading frame and RNA editing, respectively (Kumar et al. 2014). The N, P, and L proteins interact with the PPRV genome to form the ribonucleoprotein (RNP) complex. H and F are the glycoproteins on the surface of the virus envelope mediating interaction with cell receptors and fusion with the cell membrane, respectively. The M protein interacts with the RNP and glycoproteins and plays an important role in the formation of new virus particles and their budding through the cell membrane (Kumar et al. 2014). The C and V proteins, along with some of the structural proteins, mediate efficient virus replication and PPRV’s capacity to evade immune responses (Chinnakannan, Nanda, and Baron 2013; Kumar et al. 2014; Sanz Bernardo, Goodbourn, and Baron 2017; Linjie et al. 2021).

Based on phylogenetic analyses of PPRV partial N gene sequences, PPRV has been classified into four genetic lineages (LI, LII, LIII, and LIV) (Banyard et al. 2010), although there is only one serotype, with widely available vaccines protecting against all circulating PPRV strains (Hodgson et al. 2018). All strains reported in Asia and the Caucasus region belong to LIV and have led to emergence in new areas such as Georgia (Donduashvili et al. 2018) and Mongolia (Sprygin et al. 2022). PPR has been endemic in some Asian countries for a long time, such as India, where the disease was first officially reported in 1987 but has possibly been present for much longer (Baron et al. 2016). LIII had been reported in some Middle Eastern countries in the past (Banyard et al. 2010), but all recent reports concern LIV strains (Clarke et al. 2017; Alidadi et al. 2021). All four genetic lineages are present in Africa, with LI and LII circulating in West Africa and LIII limited to East Africa (Dundon, Diallo, and Cattoli 2020). LIV has been reported in some African regions since 1997 (Banyard et al. 2010) but appears to have been spreading in new areas of the continent over the past 10–15 years, notably replacing LII in West Africa (Dundon, Diallo, and Cattoli 2020; Mantip et al. 2021; Tounkara et al. 2021). All evidence so far points to an African origin of PPR, first described in 1942 in Côte d’Ivoire (Gargadennec and Lalanne 1942), but probably reported falsely as rinderpest in goats in West Africa since the 19th century (Baron et al. 2016). *Morbillivirus* phylogeny suggests that the ancestor of PPRV may be older than rinderpest or measles, which have been circulating since antiquity (Düx et al. 2020). Therefore, PPRV’s history is likely to be more ancient and complex than described in works based on veterinary records or evolutionary studies.

Full genome sequencing of PPRV strains has proved to be critical for increasing our understanding of PPR epidemiology and to inform strategies for its control and surveillance. Notably, PPRV phylogenomic studies have provided insights into the evolutionary history of the virus (Muniraju et al. 2014; Mahapatra et al. 2021), the dynamics of PPR emergence in China (Liu et al. 2018) and Israel (Clarke et al. 2017), and on the molecular epidemiology of PPRV spillover into wildlife in Mongolia (Benfield et al. 2021). PPRV genome analyses have also been important in exploring the origin and the attenuation process of PPR vaccine strains (Eloiflin et al. 2019; Kwiatek et al. 2022). The new focus of PPR global eradication efforts on episystems—delimitating areas with extensive transboundary PPR circulation—gives increased importance to gathering more PPRV partial and complete genome data to support epidemiologically informed control strategies (FAO, WOAH 2022). However, the number of full PPRV genomes published is still limited, with a heavy bias towards recent samples and LIV strains. Indeed, the dataset made available by the WOAH reference laboratory network for PPR (https://www.ppr-labs-oie-network.org/), curated following well-defined guidelines (Baron and Bataille 2022), provides only 105 validated sequences, of which eighty-six belong to PPRV LIV, whereas other lineages are represented by only a handful of sequences. Moreover, this dataset includes only twelve sequences from strains older than the Year 2000. This paucity of data seriously hinders our capacity to understand PPRV’s evolutionary and transmission dynamics.

Long-term research collaborations in West Africa have made it possible to collect many PPRV samples from multiple countries in the region. Phylogenetic studies based on short portions of the viral genome have shown the extensive transboundary circulation of the endemic LII in West Africa (Tounkara et al. 2019, 2021; Bataille et al. 2021). Moreover, the French Agricultural Research Centre for International Development (CIRAD, Montpellier, France), as a WOAH and FAO reference laboratory for PPR, holds several historical PPRV samples of interest to improve our understanding of PPRV’s evolutionary history. Here, we present the results of an effort to sequence twenty-five samples of PPRV collected in 2010–6 and seven samples in 1972–99, considered here as ‘historical’ due to the paucity of data before 2000. This study focuses mainly on LII in West Africa, providing the first opportunity to compare evolutionary pressures and history between the globally dominant PPRV genetic LIV and LII, which is endemic in West Africa. We assess whether the differences in PPR transmission dynamics between the two lineages (i.e. restricted endemic distribution of LII versus the global expansion of LIV) are reflected in detectable differences in mutation frequencies, selection pressure, and codon usage between the two lineages.

## Results

### Full genome sequencing of historical and recent PPRV isolates from West Africa and other regions

**Figure 1.**
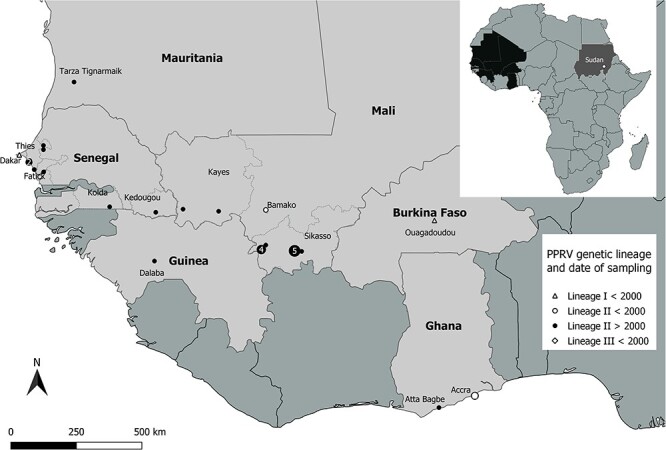
Map of the geographical location of PPRV samples sequenced in this study. The small map in the upper right corner indicates the countries sampled in Africa and the position of the sample collected in Sudan. The sample from India is not represented. Samples belonging to PPRV genetic LI are represented by a triangle, LII by a circle, and LIII by a diamond (in the small map). The symbols used differentiate historical samples (date <2000) from recent samples. Numbers within the symbols indicate the number of samples sequenced, if there is more than one per location.

The full genome sequence of PPR virus was obtained from a total of twenty-five recent (2010–6) goat and sheep tissue samples and seven historical (1972–99) virus isolates (Table 1) stored at the WOAH/FAO and the EU reference laboratory for PPR at CIRAD, Montpellier, France (https://eurl-ppr.cirad.fr/; https://www.ppr-labs-oie-network.org/). Tissue samples had been collected previously in multiple West African countries by their respective national veterinary services within the framework of other studies (Kwiatek et al. 2007; Tounkara et al. 2019, 2021; Bataille et al. 2021) (Fig. 1; Table 1). Historical PPRV isolates included strains from the four genetic lineages collected in Senegal and Burkina Faso (LI), Ghana (LII), Sudan (LIII), and India (LIV). Genomes were obtained by Illumina high-throughput sequencing after random or targeted amplification of complementary DNA (cDNA), depending on the samples (Table 1). The percentage of coverage for all genomes was >99 per cent, with the mean depth varying from 79 to 18,023 reads per site (Table 1; Sequence Read Archive accession number: PRJNA717034). The missing portions corresponded mostly to the 3′ and 5′ extremities of the genomes, which were retrieved using a rapid amplification of cDNA-ends (RACE) approach. A final, complete consensus sequence was obtained for all thirty-two samples (GenBank accession numbers: OR286474–OR286505). Multiple recombination tests were used to confirm that none of the new PPRV genomes were contaminated by portions of genomes from other PPRV strains manipulated in our laboratory (Baron and Bataille 2022). The genomes were then aligned with a set of already published PPRV genomes (total of 103 sequences), curated as in Baron and Bataille (2022), to create multiple datasets to be used in subsequent analyses, including two datasets specific for LII and LIV and one for the coding region of each PPRV gene.

**Table 1.** List of samples and genome sequencing results.

									Reference	
Name	Region	Village/commune	Year	Latitude	Longitude	Genetic lineage	Mean depth	Accession number	Sampling	Sequencing
	Senegal									
Senegal/Dakar/1994*	Dakar	Dakar	1994	14.74	−17.41	I	573	OR286474	Kwiatek et al. (2007)	Victoria et al. (2009)
Senegal/Soum/2012/2*	Fatick	Soum	2012	14.09	−16.48	II	4,407	OR286488	Bataille et al. (2021)	Victoria et al. (2009)
Senegal/Kedougou/2016/31	Kedougou	Kedougou	2016	12.55	−12.20	II	9,520	OR286499	Tounkara et al. (2021)	Eloiflin et al. (2019)
Senegal/Pakour/2013/2	Kolda	Pakour	2013	12.76	−13.96	II	6,015	OR286483	Bataille et al. (2021)	Victoria et al. (2009)
Senegal/Nguekhokh/2010/2	Thiès	Nguekhokh	2010	14.52	−17.01	II	10,811	OR286476	Bataille et al. (2021)	Victoria et al. (2009)
Senegal/Ngairing/2010/1		Ngairing	2010	14.46	−17.04	II	7,320	OR286489	Bataille et al. (2021)	Victoria et al. (2009)
Senegal/Ngairing/2010/9						II	6,915	OR286477	Bataille et al. (2021)	Victoria et al. (2009)
Senegal/SakhMecke/2012/3		Sâkh Mécké	2012	14.94	−16.49	II	9,754	OR286485	Bataille et al. (2021)	Victoria et al. (2009)
Senegal/GayeMecke/2013/1		Gaye Mécké	2013	15.10	−16.49	II	11,204	OR286487	Bataille et al. (2021)	Victoria et al. (2009)
Senegal/MbourJoal/2013/2		Mbour Joal	2013	14.18	−16.84	II	8,653	OR286486	Bataille et al. (2021)	Victoria et al. (2009)
	Guinea									
Guinea/Dalaba/2013		Dalaba	2013	10.69	−12.25	II	6,205	OR286479	Bataille et al. (2021)	Victoria et al. (2009)
	Mali									
Mali/Bamako/1999*	Bamako	Bamako	*1999*	12.64	−8.00	II	10,785	OR286500	Kwiatek et al. (2007)	Eloiflin et al. (2019)
Mali/Kayes/2016/39	Kayes	Kayes	2016	12.67	−11.16	II	7,913	OR286503	Tounkara et al. (2021)	Eloiflin et al. (2019)
Mali/Kolondieba/2013/1	Sikasso	Kolondieba	2013	11.08	−6.90	II	6,612	OR286496	Bataille et al. (2021)	Eloiflin et al. (2019)
Mali/Kolondieba/2013/4						II	12,297	OR286480	Bataille et al. (2021)	Victoria et al. (2009)
Mali/Kolondieba/2013/5						II	7,981	OR286497	Bataille et al. (2021)	Eloiflin et al. (2019)
Mali/Kolondieba/2013/6						II	9,942	OR286481	Bataille et al. (2021)	Victoria et al. (2009)
Mali/Kolondieba/2013/18						II	15,289	OR286482	Bataille et al. (2021)	Victoria et al. (2009)
Mali/Tousseguela/2014/14		Tousseguela	2014	11.06	−6.63	II	18,023	OR286495	Tounkara et al. (2019)	Eloiflin et al. (2019)
Mali/Samako/2014/9		Samako	2014	11.13	−8.17	II	13,275	OR286491	Tounkara et al. (2019)	Eloiflin et al. (2019)
Mali/Samako/2014/10						II	8,430	OR286492	Tounkara et al. (2019)	Eloiflin et al. (2019)
Mali/Samako/2014/12						II	13,645	OR286493	Tounkara et al. (2019)	Eloiflin et al. (2019)
Mali/Samako/2014/13						II	12,297	OR286494	Tounkara et al. (2019)	Eloiflin et al. (2019)
Mali/Sekou/2014/3		Sekou	2014	11.18	−8.15	II	10,730	OR286490	Tounkara et al. (2019)	Eloiflin et al. (2019)
Mali/Sagabari/2014/10	Kayes	Sagabari	2014	12.59	−9.80	II	12,630	OR286484	Bataille et al. (2021)	Victoria et al. (2009)
	Mauritania									
Mauritania/Tarza/2012		Tarza Tignarmaik	2012	17.52	−15.32	II	155	OR286478	Bataille et al. (2021)	Victoria et al. (2009)
	Burkina Faso									
BurkinaFaso/Ouagadoudou/1988*		Ouagadoudou	*1988*	12.24	−1.56	I		OR286475	Kwiatek et al. (2007)	Victoria et al. (2009)
	Ghana									
Ghana/Accra/1976*		Accra	*1976*	5.55	−0.02	II	79	OR286501	Kwiatek et al. (2007)	Victoria et al. (2009)
Ghana/Accra/1978*			*1978*	5.55	−0.02	II	452	OR286502	Kwiatek et al. (2007)	Victoria et al. (2009)
Ghana/Atta Bagbe/2014*		Atta Bagbe	2014	5.10	−1.39	II	12,899	OR286498	Tounkara et al. (2019)	Eloiflin et al. (2019)
	Sudan									
Sudan/Sinar/1972		Sinar	*1972*	13.55	33.6	III	80	OR286505	Kwiatek et al. (2007)	Victoria et al. (2009)
	India									
India/Kolkota/1995		Kolkota	*1995*	22.57	88.36	IV	829	OR286504	Kwiatek et al. (2007)	Victoria et al. (2009)

All samples sequenced are swabs, except (*), corresponding to strains isolated in cell culture from field samples; genetic lineage corresponds to the PPRV genetic lineage each sample belongs to; year is year of collection, with dates older than 2000 indicated in italics to highlight samples considered as historical; mean depth is the mean number of sequence reads per site obtained by high-throughput sequencing; accession number is the GenBank accession number for each consensus PPRV genome; reference sampling is a research article describing sampling collection and initial partial PPRV N gene sequencing; reference sequencing is a research article describing the method used for high-throughput genome sequencing.

### Phylogenetic analyses, estimation of TMRCA, and evolutionary rates

Analysis with TempEst (Rambaut et al. 2016) confirmed that our dataset contained enough temporal signal to proceed with time-dependent phylogenetic analyses (*R*^2^ = 0.831, correlation coefficient = 0.911; Fig. S1). The sequence Senegal/Dakar/1994 was identified as an outlier, with an incongruent level of genetic divergence compared to the rest of the data, possibly due to the number of passages in cell culture for this isolate (at least ten passages, compared to one to three passages for the other isolates). Due to the limited number of existing PPRV LI sequences and the focus of this study on LII and LIV, we decided to keep the sequence in our analyses, although future phylogenomic analyses concentrating on LI may want to remove it from their analyses.

**Figure 2.**
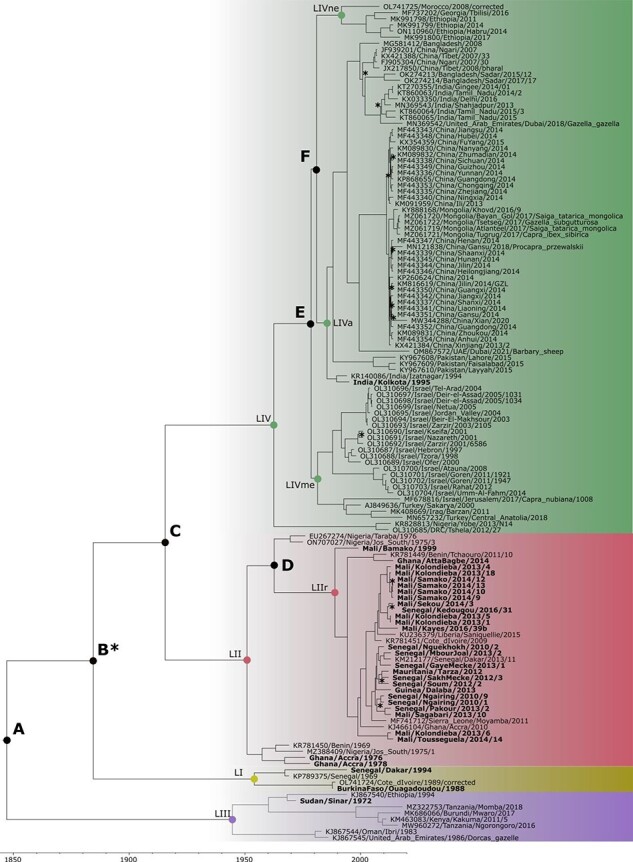
Time-dependent phylogenomic tree of PPRV. The four PPRV genetic lineages (LI, LII, LII, LIV) are represented by coloured backgrounds. Nodes of the tree with estimated TMRCA are indicated with a coloured circle for nodes joining PPRV sequences and by letters for deeper nodes in the tree, as indicated in the text and Table 2. Names of nodes of specific interest follow Table 2: LII recent (LIIr), LIV Asia (LIVa), LIV North-East Africa (LIVne), and LIV Middle East (LIVme). Names of PPRV strains sequenced for this study are indicated in bold. Asterisks indicate the few nodes of the tree with poor posterior probability <0.9.

Bayesian phylogenetic analysis on the complete genome PPRV dataset placed the thirty-two new genomes in the genetic PPRV lineages expected based on results of previous studies using partial PPRV genetic sequences (Tounkara et al. 2019, 2021; Bataille et al. 2021) (Table 1, Figs 1 and 2). Within PPRV LI, Burkina Faso/Ouagadougou/1988 was closely related to Ivory Coast/1989, whereas Senegal/Dakar/1994 clustered with Senegal/1969. The strain Mali/Bamako/1999 had a position in the tree at the base of all the PPRV genomes of LII collected at later dates, all grouped in one well-defined cluster (LIIr in Fig. 2). The strains Ghana/Accra/1976 and Ghana/Accra/1978 were placed in a separate LII cluster with Benin/1969 and Nigeria/Jos South/1975/1. The strain Sudan/Sinar/1972 was most closely related to Ethiopia/1994 within LIII (Fig. 2). Most sequences of the LIV were clustered in well-defined groups corresponding in most part to geographic regions, defined here as Asia (LIVa), North-East Africa (LIVne), and the Middle East (LIVme; Fig. 2). The strain India/Kolkota/1995 was closely related to India/Izatnagar/1994, both placed at the base of LIVa. The two strains Nigeria/Yobe/2013/N14 and DRC/Tshela/2012/27 formed a well-separated group within LIV, a sister clade to all other LIV strains (Fig. 2).

As observed in previous studies based on partial PPRV genetic sequences (Tounkara et al. 2019, 2021; Bataille et al. 2021), the genomes of PPRV strains collected in recent years in West Africa do not form single, country-specific clades within LII but are intricately intermingled (Fig. 2). The phylogenetic relationships in this lineage were comparable to those described using complete N and H gene sequences (Bataille et al. 2021) for the strains common to the two studies (Table 1). Notably, this was the case for the close relationship observed between two strains collected in the regions of Kolda in Senegal (Senegal/Pakour/2013/2) and Kayes in Mali (Mali/Sagabari/2014/10), separated by several hundred kilometres by road (Fig. 1). However, the phylogenetic resolution was much stronger in this study, with most nodes showing posterior probability support >0.90 (Fig. 1). This increased resolution made it possible to identify a close phylogenetic relationship between Senegal/SakhMecke/2012/3 and Mauritania/Tarza/2012, which was not observed in previous studies (Bataille et al. 2021). Another notable transboundary cluster identified in this new phylogenomic analysis grouped three sequences from Sekou and Kolondieba in the Sikasso region of Mali with a strain collected in the Kedougou region of Senegal, at the border with Mali (Figs 1 and 2). The new genome, Ghana/Atta Bagbe/2014, clustered with Benin/2011, with both well separated from all other recent LII strains sequenced in West Africa (Fig. 2).

The substantial increase in the number of PPRV genomes obtained in our study, including some historical samples, gave us the opportunity to provide a new evaluation of evolutionary rates and of the time to the most recent common ancestor (TMRCA) within the PPRV phylogeny. Time-scaled Bayesian phylogenetic analyses provided an estimation of the mean evolutionary rate across the PPRV phylogeny of 6.04E − 4 nucleotide substitutions/site/year (95 per cent High Probability Density (HPD) interval: 4.68E − 4–7.53E − 4). Analyses performed on datasets specific to LII and LIV gave estimations of mean evolutionary rates of 5.27E − 4 (95 per cent HPD interval: 4.72E − 4, 5.88E − 4) and 5.52E − 4 (95 per cent HPD interval: 5.00E − 4, 6.00E − 4) nucleotide substitutions/site/year, respectively. The age of the root of all PPRV sequences was 1847 (95 per cent HPD interval 1764–1914; Node A in Table 2 and Fig. 2), corresponding to the start of the divergence of LIII from other circulating PPRV strains. Divergence of the three other PPRV genetic lineages was estimated to have taken place between the beginning of the 19th century and the middle of the 20th century (Nodes B–C in Table 2 and Fig. 2). The TMRCA of the known sequences of each of the four genetic lineages was quite similar, with mean values ranging between 1945 and 1963. All recent strains belonging to LII had a common ancestor dating to 1994 (node LIIr, 95 per cent HPD interval 1987–2000), and it was estimated that they started to diverge in 1963 (Node D, 95 per cent HPD interval 1953–71). The TMRCA of the different clades of LIV (LIVa, LIVne, and LIVme) ranged between 1981 and 1992, with the divergence of these different clades estimated to have occurred between 1979 and 1981 (Nodes E and F; Table 2, Fig. 2).

**Table 2.** Estimated TMRCA for different nodes of the PPRV phylogeny according to Fig. 2, with associated 95 per cent HDP.

	TMRCA	95 per cent HPD
Root (Node A)	1847	1764–1914
Node B	1884	1829–1927
Node C	1915	1871–1953
Node D	1963	1953–71
Node E	1979	1971–86
Node F	1981	1974–87
LI	1954	1934–67
LII	1951	1937–62
LIII	1945	1921–64
LIV	1963	1938–80
LII recent strains (LIIr)	1994	1987–2000
LIV Asia (LIVa)	1986	1980–91
LIV Middle East (LIVme)	1981	1974–88
LIV North/East Africa (LIVne)	1992	1983–2001

### Selection pressures

Using multiple methods, selection pressures were explored across individual sites, branches, and the coding region of each gene across the phylogeny and separately for PPRV LII and LIV. By comparing the results from these methods, we increased our capacity to identify robust signatures of selection pressure. The fixed effect likelihood (FEL), fast unconstrained Bayesian approximation (FUBAR), and mixed effects model of evolution (MEME) methods were used to detect site-specific selection pressure across the PPRV phylogeny. Some codon positions under positive selection were identified in Genes P and F with all three methods, in H and L genes with only two methods, and in the N and M genes only with MEME (Table 3). A total of six amino acid sites were detected with more than one method for P, but only one or two sites were detected more than once for F, H, and L (Table 3). The FEL analysis was also run for the coding region of each gene, first with LII sequences and then with LIV sequences. In all genes except M, multiple codon positions were under positive selection, but these were different for LII and LIV sequences (Table 4). The exception was Codon Position 476 of the H gene, identified as under positive selection in both lineages. The branch-site unrestricted statistical test for episodic diversification (BUSTED) method was used to identify gene-wide evidence of positive selection in LII and LIV. Using this approach, signs of positive selection were detected only for the L and N genes in LIV (Likelihood Ratio Test (LRT) = 49, P-value < 0.001 and LRT = 6, P-value = 0.007, respectively). The RELAX method was also used to test for the difference in selection pressure between LII and LIV for each gene. A difference in selection pressure was identified only for the L gene (LRT = 20.29, P-value < 0.001).

**Table 3.** Amino acid sites under selection pressure across the whole PPRV phylogeny.

Gene	FEL	FUBAR	MEME
N	–	–	426, 503, 508
P	**52**, 77, **101, 137, 161, 284, 295**	**161**	10, 20, **52**, 98, **101**, 102, 132, **137**, 139, **161**, 209, **284, 295**, 317
M	–	–	212, 311, 319
F	**8**	**8**, 13	**8**, 145
H	**246**, 344, **476**, 574	–	210, 245, **246, 476**, 534
L	124, 614, 623, 1257, **2115**	–	54, 319, 467, 547, 647, 719, 720, 723, 862, 899, 1207, 1508, 1568, 1578, 1594, 1847, 1901, **2115**

Sites detected with multiple methods are in bold; symbol ‘–’ indicates that no site was identified for a gene using this method.

**Table 4.** Amino acid sites under selection pressure according to the FEL method, for LII and LIV PPRV gene sequences.

	FEL-LII	FEL-LIV
**N**	211	46, 426
**P**	52, 79, 102, **137**, 139, 160,	77, **161**, 171, 277, 284,
	166, 222, 244, 276	295, 381
**M**	–	–
**F**	5, 11	**8**, 486
**H**	156, 203, 247, **476**	**476**
**L**	623, 647, 1563, 2122	124, 614, 616, 1257

Sites identified as under positive selection for the complete PPRV sequence dataset using multiple methods (results shown in Table 3) are indicated in bold; symbol ‘–’ indicates that no site were identified for a gene using this method.

### Codon usage

**Figure 3.**
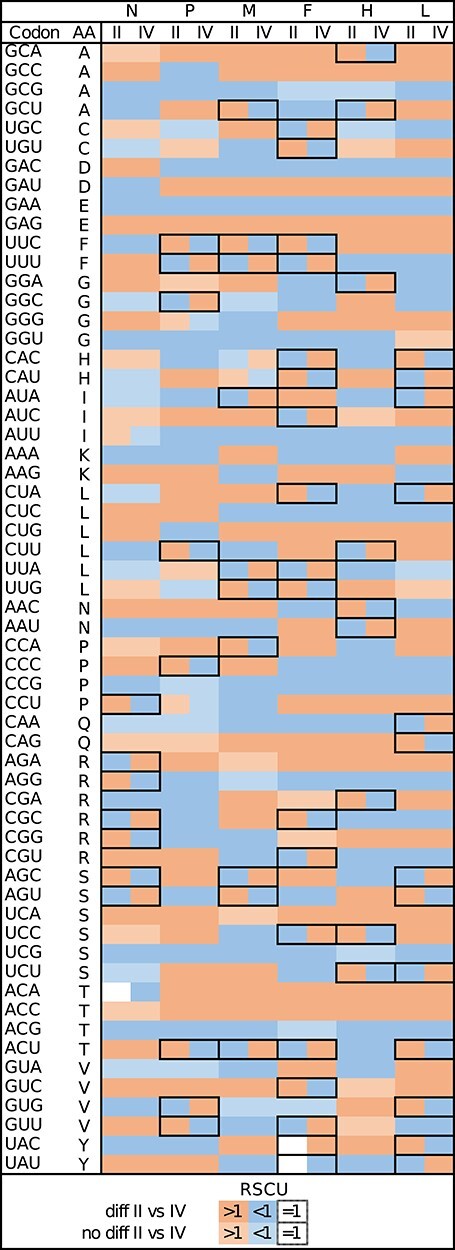
Heatmap of RSCU showing codons used more (>1) or less (<1) than expected in PPRV genes in LII and LIV. Codons with RSCU which significantly differ between lineages (P < 0.05, after correction) are in darker shades of colour. Cases of codons used more than expected for one lineage but used less for another are framed.

A strong body of research shows how synonymous codon usage can be biased by evolutionary and functional factors such as RNA stability, protein secondary structure, mutation pressure, and translational selection (Bahir et al. 2009; Ma et al. 2017; Taylor, Dimitrov, and Afonso 2017). In the case of viruses, codon usage may also be biased to mirror codon preference in their hosts to maximise virus fitness (Bahir et al. 2009; Ma et al. 2017). It is a process especially interesting to study in the case of PPRV, as host susceptibility to PPRV differs widely depending on the host species, breed, environment, and viral strains (Couacy-Hymann et al. 2007; Fakri et al. 2017; Eloiflin et al. 2022). We assessed whether codon usage differed between PPRV lineages in the different viral coding regions using multiple indicators. Relative synonymous codon usage (RSCU) was highly correlated between LII and LIV for all genes (Table 5). However, multiple codons presented significantly different usage for each gene between LII and LIV, with one lineage showing an RSCU value >1, while the other had a value <1, corresponding to positive and negative codon usage bias, respectively (Fig. 3; Table S1). The N gene had the lowest number of codons showing a difference in RSCU between lineages (seven codons coding for three different amino acids), whereas the F gene had the highest number of codons with different usages (eighteen codons, ten amino acids; Fig. 3; Table S1).

**Table 5.** Pearson’s *r* correlation between RSCU values obtained for coding sequences of each PPRV gene of LII and LIV and of mean CAI with mean ENC for PPRV genes in LII and LIV.

		CAI versus ENC	
	RSCU LII versus LIV	LII	LIV
N	0.93***	−0.30	−0.79***
P	0.85***	−0.07	−0.29*
M	0.86***	−0.67***	−0.41**
F	0.76***	0.08	0.24
H	0.87***	−0.48**	0.16
L	0.93***	0.23	−0.71***

*R* values significantly different from 0 are indicated with (*) for p < 0.05, (**) p < 0.01, and (***) p < 0.001.

**Figure 4.**
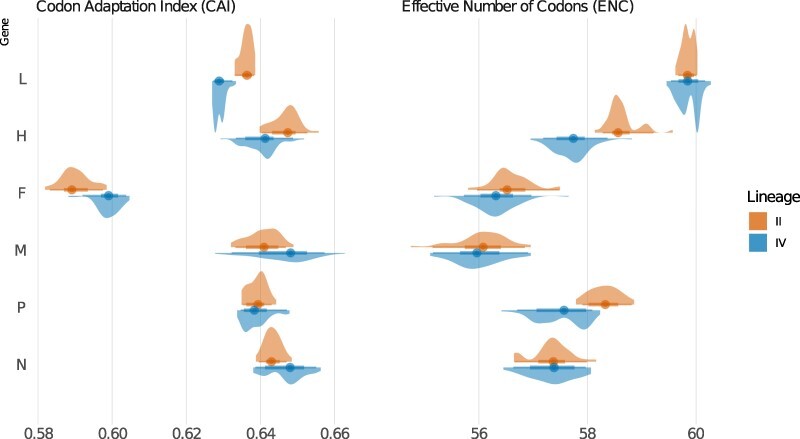
Kernel density estimates of codon usage for each PPRV gene using coding sequences belonging to PPRV LII and LIV, as calculated with the mean CAI (panel on the left), using the genome of *O. aries* as a reference, and with the ENC (panel on the right).

The mean codon adaptation index (CAI) values were also calculated for all genes in LII and LIV as a way to evaluate the adaptation of the different lineages to one of their main host species, sheep (no reference set was available for goats). High CAI values suggest better adaptation to the host translational machinery. The values obtained ranged between 0.590 and 0.647 and were significantly different between lineages for all genes (Wilcoxon test, W = 83–2,415, P-value range = 0.03 to <0.0001, adjusted for multiple comparisons), except for P (W = 1,130, P-value = 0.26; Table S2). CAI values were higher for LIV in Genes N, M, and F but higher for LII in H and L (Fig. 4). However, differences in mean CAI between the two lineages were small (delta = 0.03–0.09; Fig. 4; Table S2). The F gene had CAI values much lower than all other genes (LII = 0.59, LIV = 0.60; Fig. 4, Table S2).

The effective number of codons (ENC) was calculated to evaluate to what extent the different synonymous codons were used for each amino acid in our datasets. The highest value of 61 indicates the use of most synonymous codons and therefore limited evolutionary constraints in codon usage. Mean ENC was significantly higher for LII compared to LIV for the P, F, and H genes (Wilcoxon test, W = 1,249–1,958, P-values <0.001, FDR adjusted; Fig. 4; Table S2). There was no difference in mean ENC between lineages for the N, M, and L genes (Wilcoxon test, W = 931–1,082, P-values = 0.16–0.66, FDR adjusted; Table S2). The lowest ENC values were observed for M (LII = 56.06, LIV = 55.99). The highest ENC values were obtained for L (mean ENC = 59.9 for both lineages, Fig. 4; Table S2).

**Figure 5.**
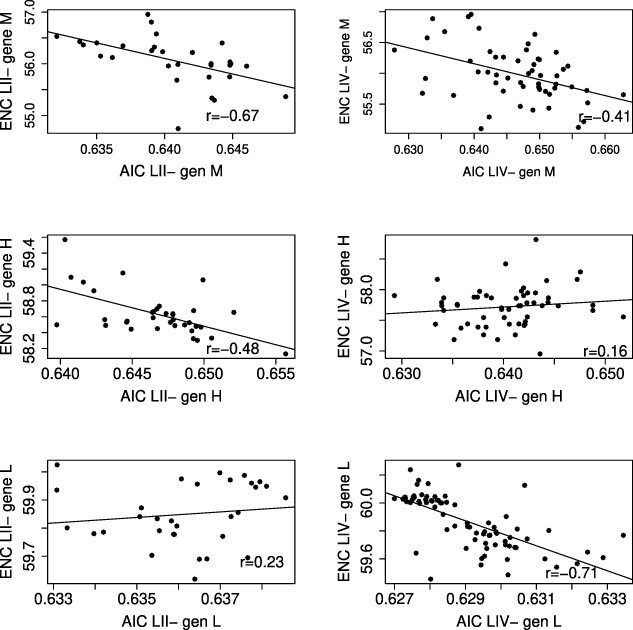
Pearson’s *r* correlation between CAI and ENC for Genes M, H, and L of PPRV strains belonging to LII (left column) and LIV (right column). All correlation results are detailed in Table 4.

ENC values are influenced by both selection and mutation bias, whereas CAI is mostly controlled by selection (Vicario, Moriyama, and Powell 2007). Pearson’s correlations between mean CAI and ENC were carried out to assess the relative importance of selection and mutation bias in patterns of codon usage (Vicario, Moriyama, and Powell 2007; Taylor, Dimitrov, and Afonso 2017). There was a correlation between mean CAI and mean ENC for the M gene in LII and LIV (Table 5), indicating that selection has a strong role in the codon bias observed. A strong correlation was also observed between CAI and ENC for H in LII and for N and L in LIV (Table 5; Fig. 5). CAI and ENC were not correlated for the F gene in both lineages, indicating that codon bias was consistent with random mutation or unknown pressure.

## Discussion

RNA viruses are typically characterised by high evolutionary rates, making it possible to study their evolutionary dynamics at multiple time and spatial scales using virus sequence data (Pybus and Rambaut 2009). However, challenges remain with phylodynamic inferences in complex systems, including dealing with sampling bias (Frost et al. 2015). In the case of PPRV, sequence data are heavily biased towards recent strains of one specific genetic lineage, LIV. Our study represents the beginning of an effort to correct this bias, substantially increasing the number of sequence data for PPRV LII and for virus strains from before 2000, considered here as historical. The new estimate of PPRV nucleotide substitution rate from this study is slightly lower than estimates from previous studies (Muniraju et al. 2014; Benfield et al. 2021; Mahapatra et al. 2021), although with overlapping HPD intervals and remaining within the range expected for RNA viruses (10^−3^ to 10^−4^ substitutions per site per year), bearing in mind that such measurements poorly represent a highly dynamic feature of molecular evolution (Duchêne, Holmes, and Ho 2014). With such a fast pace of evolution, this improved PPRV genome dataset provides us with new opportunities to improve our understanding of the evolutionary processes shaping PPRV transmission and current distribution. More specifically, we are now able to compare evolutionary dynamics between two PPRV genetic lineages with distinct histories and distribution. On the one hand, LII is endemic and restricted to West Africa, with an extensive transboundary circulation of the virus within this region, clearly represented in our phylogenomic analysis and previous studies (Tounkara et al. 2019; Bataille et al. 2021). This highlights the need for West African countries in this shared episystem to harmonise their surveillance and control activities. On the other hand, LIV has spread across Africa, Asia, and the Middle East, producing what appears to be regionally defined sub-lineages specific to East Asia, North and East Africa, and the Middle East. The position of PPRV sequences from Georgia and the United Arab Emirates out of clusters specific to their region shows that transmission events across distant regions can happen on occasion, likely due to international trade (Donduashvili et al. 2018). However, this study and previous phylogenomic analyses suggest that transmission dynamics remain largely regional, with emergence most likely due to the movement of infected animals across borders (Mahapatra et al. 2021).

Adding new sequences from historical strains belonging to the four genetic lineages did not modify previous estimates of the timing of PPRV and genetic lineage emergence based on TMRCA analyses, with early lineage divergences placed between the 18th and 19th century (Muniraju et al. 2014; Benfield et al. 2021; Mahapatra et al. 2021). All point towards the circulation of PPRV more than 100 years earlier than its formal description in 1942. Historic samples of PPRV remain relatively recent (the oldest is from 1969), and time estimates for viral ancestry have been found to be much too recent when calibrations are based on short-term rates of evolution (Sharp and Simmonds 2011), meaning it is likely that PPRV has been circulating over a longer time period. This hypothesis is reinforced by a recent *Morbillivirus*-wide study placing the divergence of rinderpest and measles viruses from the ancestor of PPRV at around the fourth millennium Before Common Era (Düx et al. 2020).

Interestingly, the TMRCA for all four PPRV genetic lineages is placed within a very short period of time, between 1945 and 1963, although these estimates come with a wide variance and depend on the availability of historical samples. It seems unlikely that PPRV samples older than 1969 can be retrieved and that we will ever be able to get more reliable estimates of PPRV lineage divergence events. Still, the new data obtained here for all four lineages can provide some information for more recent events that could affect evolutionary processes in the various PPRV lineages. Notably, the divergence of the recent LII strains was placed in the 1960s, while the divergence of the regional clades within LIV was all placed in the early 1980s. These results suggest that these periods might have been particularly important for the diversification and spread of PPRV globally, possibly in association with political and economic changes such as the independence of African countries and the intensification of the animal trade at a global level. Progress in the eradication of rinderpest virus, and the possible cross-protection of sheep and goats against PPR from subclinical infection with rinderpest, may also have played a role in facilitating PPR spread (Baron et al. 2016).

Despite the limited number of historical samples, our results still point to a complex PPRV phylogenetic history between the 1960s and 1980s. Notably, our phylogenetic tree shows that PPRV LII sequences from the 1970s form two different, well-separated clusters, suggesting that this lineage was very diverse in that period. Four PPRV LII strains collected at that time in Benin, Nigeria, and Ghana form a group with no direct phylogenetic relationship with the recent PPRV LII strains. These results suggest that this cluster died out at some point after 1978. Something similar could be observed for Oman/1983 and UAE/1986 within LIII, although only a few genomes are available for this lineage, limiting our capacity to interpret phylogenomic data. Even fewer genomes are available for LI, but they nevertheless show that this lineage was still genetically diverse in the late 1980s. It would be interesting to obtain the sequence of the LI still circulating in Mali in 2014 to assess its phylogenetic relationship with older LI strains. Sequencing of additional PPRV genomes from historical PPRV strains could help to understand the evolutionary history of the virus, notably the role of bottleneck events in shaping the current virus phylogeny.

Even for recent strains, our phylogenomic analysis suggests that there are still major gaps in the genome data. The separation of Ghana/Atta Bagbe/2014 and Benin/2011 from the rest of the LII genomes shows that the genetic diversity of this lineage is likely to be much larger than what we can currently observe. It is possible that more sequencing efforts in West African countries not yet represented will help complete our understanding of the phylogeny of this lineage. Concerning LIV, the position in the phylogeny of the only two genomes available from West and Central Africa (Nigeria and Democratic Republic of Congo) suggests that this lineage originated in these regions. Sequencing more LIV genomes from West and Central Africa should be a priority for future research in order to resolve the phylogeographic history of this lineage (Benfield et al. 2021; Mahapatra et al. 2021) and to further our understanding of its spread across West Africa in recent years (Tounkara et al. 2018; Dundon, Diallo, and Cattoli 2020). Phylogenetic studies based on partial N gene already point towards the high diversity and wide circulation of the LIV in the region and provide further support for the need of full genome sequencing effort (Dundon, Diallo, and Cattoli 2020; Mantip et al. 2021; Tounkara et al. 2021).

Although still incomplete, the sequence data for PPRV LII and LIV from this study made it possible to implement some initial comparative analyses of evolutionary processes between a lineage that has remained endemic to a region and a lineage that has successfully spread in multiple regions of the globe. No difference in nucleotide substitution rates was observed between LII and LIV, but analyses of selection pressures clearly suggest that these lineages have evolved under different selection regimes. A large majority of codon positions identified as under positive selection in the six structural PPRV genes differ between the two lineages. Some amino acid sites found under selection in only one lineage were also identified when testing the complete PPRV phylogeny. These amino acids may be of special interest for further investigation as they could represent strong signals of lineage-specific adaptation and functional differences. Notably, this is the case for Codon Position 161 in the P gene, coding for glycine in many PPRV sequences, except for multiple LII strains (coding for aspartic acid) and LIV strains (coding for serine). Another example is Codon Position 8 in the F gene, coding almost exclusively for valine in LII, but for threonine, alanine, lysine, or isoleucine in LIV. The impact of these mutations on viral replication and interaction with host cells could be explored in *in vitro* experiments, such as minigenome assays (Abdullah et al. 2018). The results from the methods used to detect selection pressure in branches of the PPR phylogeny also identified differential selection pressure in some viral genes between the two lineages. Notably, both methods suggested differential selection pressure in the L gene for LIV, and this was further supported by the strong correlation observed between the codon usage indicators CAI and ENC for this gene in the same lineage. These results suggest that the spread of LIV was associated with a strong adaptive selection on the viral polymerase protein. The very high ENC values for this gene, indicating the use of almost all existing synonymous codons, may be an additional indication of this gene’s adaptive capacity.

Overall, all analyses based on codon usage pointed towards different biases and, therefore, evolutionary pressure between PPRV LII and LIV. Interestingly, the F gene presented the highest number of positions with different codon usage (RSCU indicator) between PPRV LII and LIV. This gene was uniquely characterised by the lowest level of similarity of codon usage with its ovine host (CAI indicator) and a limited role of selection in codon usage (uncorrelated CAI versus ENC), suggesting that the differences observed are mainly driven by random mutations. In contrast, the effect of selection on codon usage in gene M was strong for both lineages, with the lowest level of synonymous codon usage (ENC) observed among PPRV genes. These results, added to the low number of amino acid sites under positive selection, suggest that purifying selection is strong on both synonymous and nonsynonymous changes in the codons of these two genes. Finally, it is interesting to note that the signature of selection appeared stronger on the H gene for LII according to codon usage and selection pressure analyses. As this lineage remained endemic to West Africa, with a range of hosts that is likely much smaller than for the globally distributed LIV, stronger adaptative selection pressure may have been expected in the latter for this protein directly interacting with host receptors. The addition of new genome sequences from LII and LIV may change some of the selection and codon usage patterns obtained in this study. Furthermore, results obtained here may be biased by the inclusion in our analyses of many sequences obtained from virus isolated in cell culture. Indeed, a large number of PPRV sequences published in the past originated from isolated strains and therefore may contain mutations associated with passage history. Although we have information on number of passages for the new isolates from this study, such information is often lacking for sequences in public databases. Still, differences observed between the two lineages appear numerous and strong, so it seems unlikely that all these differences would disappear with a larger dataset excluding virus isolates. However, future analyses would help identify the most robust results, representing real evolutionary processes.

The new PPRV genomes obtained for this study provided new opportunities to understand the evolutionary history of the virus and to compare for the first time the molecular evolution between two PPRV genetic lineages with different distribution and epidemiology. Our results confirm that comparative genomic analyses can provide new insights that may be helpful for the global campaign for PPR control and guide new lines of research. The process of the spread and diversification of PPRV genetic lineages, and how this could in part be associated with rinderpest eradication, is of special interest, as it may inform us on what to expect during the next phase of PPRV eradication and the risk of emergence of other ruminant viruses. Identification of lineage-specific adaptation markers would also be extremely useful, particularly in the context of the ongoing spread of LIV in Africa. PPRV genome sequencing efforts must be ramped up to increase the resolution of molecular epidemiology analyses so it can become a powerful tool for the development of efficient PPR control and surveillance strategies.

## Material and methods

### Sample preparation and high-throughput sequencing analysis

Total RNA was extracted from all samples (expurgated swabs and isolates) using a NucleoSpin RNA virus extraction kit (Macherey-Nagel) according to the manufacturer’s instructions. Different methods were used between 2014 and 2022 to prepare the library for high-throughput sequencing (Table 1). For the majority of samples, the library was generated using a modified sequence-independent, single-primer amplification method (Victoria et al. 2009). First-strand cDNA was prepared using a RevertAid First Strand cDNA Synthesis Kit (Fischer Scientific) with 0.1 ng–5 µg of RNA template and 5 µM of tagged primers containing eight N residues at the 3′ end (as described in Victoria et al. (2009)) in 20 μl final volume at 25 °C for 5 min, 42 °C for 60 min, and 70 °C for 5 min. Double-stranded cDNA was then synthetised using the DNA Polymerase I, Large (Klenow) Fragment Kit (Invitrogen), with 10 µl of cDNA template in 30 μl final volume at ambient temperature for 60 min. The generated double-stranded DNA was then amplified using Phusion High-Fidelity DNA Polymerase mix and reaction buffer in a final volume of 50 μl with 5 µM of the tagged primers without N residues. Polymerase Chain Reaction (PCR) cycling conditions were 98 °C for 30 sec, then thirty-five cycles of 98 °C for 10 sec, 65 °C for 20 sec, 72 °C for 60 sec, and a final elongation step of 72 °C for 10 min. PCR products were sent to Macrogen (South Korea) for library preparation using the TruSeq kit before sequencing on a HiSeq2500 Illumina platform in 250 bp paired-end mode.

For other samples, cDNA synthesis and PCR amplification were done with specific primers targeting five overlapping ∼3–4 kb regions of the virus as described in a previously published study (Eloiflin et al. 2019). After purification, different amplified fragments were quantified using a NanoDrop^™^ Spectrophotometer (Thermofischer, France). Fragments were pooled in an equimolar manner, and sequencing libraries were prepared using the Nextera kit (Illumina, CA, USA) following the manufacturer’s instructions. The distribution of fragment sizes within each library was analysed using an Agilent 2100 Bioanalyzer with the Agilent high sensitivity DNA kit (Agilent Technologies, Les Ulis, France), according to the manufacturer’s instructions. The quantity and average size of each library were used to pool the libraries in an equimolar manner. Sequencing was done using an Illumina MiSeq machine at the AGAP sequencing platform (CIRAD, Montpellier, France). Often, the 3′ and 5′ ends of the PPRV genome were not obtained with either of the high-throughput sequencing methodologies. In these cases, extremities were obtained using the 5′/3′ RACE Kit 2nd Generation (Roche, France) following the manufacturer’s instructions. RACE and PCR products were sent for Sanger sequencing to Genewiz (UK).

### Sequence data analyses and phylogenetic analyses

Adapter sequences were trimmed, and reads shorter than 50 nt (and their pair) were removed from the dataset using Trimmomatic v0.39 (Bolger, Lohse, and Usadel 2014). Remaining reads were mapped on the closest PPRV available genome with BWA-mem2 (Vasimuddin et al. 2019). The mismatch penalty was doubled (option -B 8) in order to map only PPRV-specific reads. A consensus was produced using the consensus tool of the SAMtools 1.17 suite (Danecek et al. 2021). A second assembly run was performed using this consensus as a reference, and the final consensus was produced in the same way, excluding sites with a coverage inferior to five and mapping quality less than fifty-one (options -d 5—min-MQ 51). Consensus sequences were checked to confirm the absence of contamination using the multiple recombination tests implemented in RDP5 (Martin et al. 2020) as described in Baron and Bataille (2022).

Consensus sequences were aligned to a dataset of publicly available PPRV genomes curated by the WOAH reference laboratories (https://www.ppr-labs-oie-network.org/) using MAFFT v7.271 (Nakamura et al. 2018). Curation included simple edition of sequences with obvious mistakes and removing sequences from strains with a long cell passage history or suspected to contain a mix of multiple strains, as described in Baron and Bataille (2022). In the process of quality checking, sequences OL741724 and OL741725 (Eloiflin et al. 2022) were corrected and revised versions were submitted to GenBank. PPRV genomes appear to have low-quality sequences in the non-coding region between M and F, known to be difficult to sequence, so this region was removed from the alignment (Baron and Bataille 2022). From this alignment of complete curated PPRV genomes, additional datasets specific to PPRV genetic LII and LIV and for each gene-specific coding region were prepared.

We used TempEst v1.5.3 (Rambaut et al. 2016) to verify that our data evolved in a clock-like manner and detect any outlier sequence that did not fit with the overall association between genetic divergence and sampling date. The phylogenetic analyses for this study were conducted within a Bayesian framework using BEAST v1.10.4 (Suchard et al. 2018). To determine the optimal combination of clock model and tree prior for our dataset, we employed the path sampling/stepping-stone sampling marginal likelihood estimation. Subsequently, we selected an uncorrelated relaxed clock model with a lognormal distribution (Drummond et al. 2006) and a coalescent constant size model (Drummond et al. 2006). The DNA evolution model was specified as the general time reversible model with four gamma categories. Two independent Markov chain Monte Carlo chains were executed, each consisting of 500 million steps. Convergence and sample sizes were assessed using Tracer 1.7.1 (Rambaut et al. 2018). Quantiles of the node ages and evolution rates were computed from the log files in R (R. Core Team 2017).

### Detection of selection pressures and codon usage analyses

The methods implemented in datamonkey.org (Weaver et al. 2018) were used to assess selection pressure in the coding sequences of each PPRV gene. Individual sites under positive selection were detected using FEL, FUBAR (Kosakovsky Pond and Frost 2005; Murrell et al. 2013), and MEME analyses (Murrell et al. 2012) across all PPRV lineages. FEL and FUBAR, respectively, employ a maximum-likelihood and a Bayesian approach to infer nonsynonymous (dN) and synonymous (dS) substitution rates, assuming that the selection pressure on each site is constant along the phylogeny. MEME uses a mixed effects maximum-likelihood approach to detect signs of episodic positive or diversifying evolution. The FEL analysis was also run for each gene, first with a subset of branches of the phylogeny corresponding to the LII selected, then with the LIV branches of the phylogeny. The RELAX method (acronym not defined (Wertheim et al. 2014)) was used to test if the stringency of natural selection differed between LII and LIV for each gene. The BUSTED approach (Murrell et al. 2015) was used to identify gene-wide evidence of positive selection in LII and LIV.

Codon usage and CAIs were estimated and compared between LII and LIV for each gene. First, RSCU (Sharp and Li 1986), CAI (Xia 2007), and ENC (Sun, Yang, and Xia 2013) were calculated using DAMBE7 (Xia and Kumar 2018). RSCU values >1 and <1 indicate positive and negative codon usage bias, respectively, whereas codons with values of 1 show no bias. Pearson’s correlations were performed to assess the relationship between codon usage between L II and LIV sequences for each gene. CAI compares the codon usage of the selected dataset with usage in a reference set (sheep, *Ovis aries*). ENC is a calculation based on the content in Guanine and Cytosine (GC content) for the third codon position. Wilcoxon’s tests were performed to detect significant difference in RSCU, CAI, and ENC values obtained within each gene set for LII and LIV, using the false discovery rate method for correction of multiple comparisons. These tests and Pearson’s correlation tests were implemented in R (R. Core Team 2017).

## Supplementary Material

veae012_Supp

## Data Availability

Raw genetic data were deposited in the National Center for Biotechnology Information (NCBI) Sequence Read Archive (accession number: PRJNA717034), and consensus genomes sequences were deposited in NCBI GenBank (accession numbers: OR286474–OR286505).
