# Supplementary material for: Comparative evolutionary analyses of peste des petits ruminants virus genetic lineages
Source: Virus Evol. 2024 Mar 6;10(1):veae012. doi: 10.1093/ve/veae012 (PMC10930206; doi:10.1093/ve/veae012)
Supplement: veae012_Supp [file veae012_supp.zip › suppl_data/Supplementary information-v3.docx]

**Supplementary information**


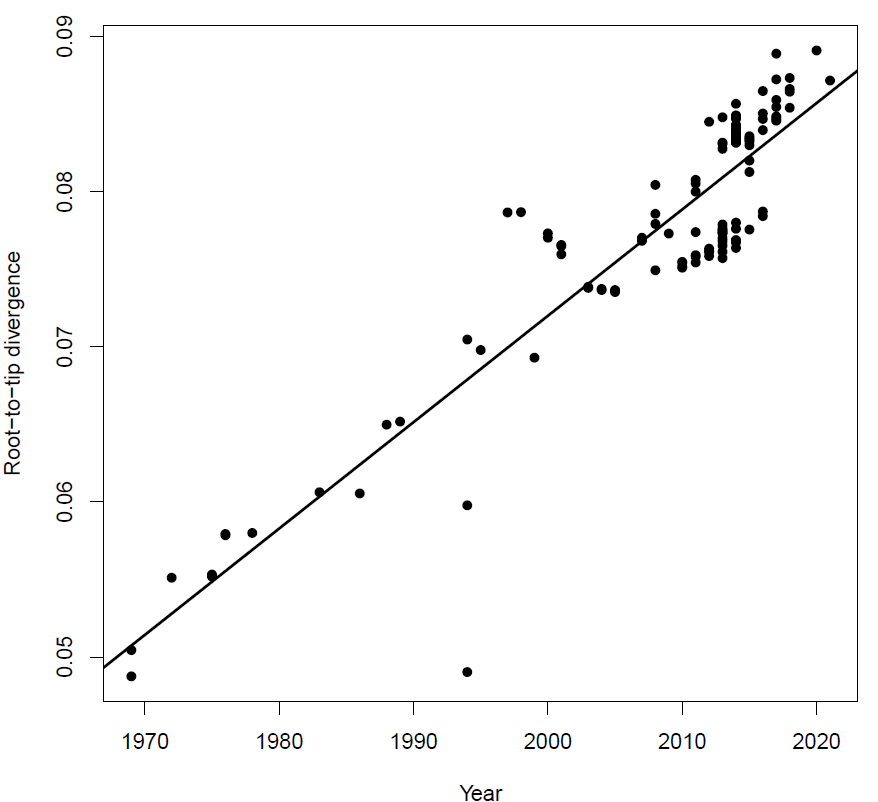


**Figure S1.** Correlation between root-to-tip genetic divergence and sampling date in the PPRV genome dataset as calculated using TemPest (see main text). The outlier dot at the bottom of the graph at date 1994 corresponds to sequence Senegal/Dakar/1994.

**Table S1.** Mean Relative Synonymous Codon Usage (RSCU) value for PPRV gene sequences belonging to either genetic lineage II or IV.

|  |  | N | | P | | M | | F | | H | | L | |
| --- | --- | --- | --- | --- | --- | --- | --- | --- | --- | --- | --- | --- | --- |
| Codon | AA | LII | LIV | LII | LIV | LII | LIV | LII | LIV | LII | LIV | LII | LIV |
| GCA | A | **1.563** | 1.566 | **1.663** | **1.609** | **1.357** | **1.611** | **1.502** | **1.751** | **1.095** | **0.907** | **1.256** | **1.246** |
| GCC | A | **1.117** | **1.051** | **0.974** | **0.923** | **1.33** | **1.458** | **1.158** | **1.099** | **1.582** | **1.477** | **1.313** | **1.348** |
| GCG | A | **0.789** | **0.557** | **0.225** | **0.422** | **0.234** | **0.034** | 0.406 | 0.397 | 0.487 | 0.475 | **0.241** | **0.306** |
| GCU | A | **0.531** | **0.827** | **1.138** | **1.045** | **1.079** | **0.897** | **0.935** | **0.753** | **0.837** | **1.14** | **1.189** | **1.101** |
| UGC | C | 1.875 | 1.938 | 0.508 | 0.551 | **2** | **1.6** | **0.819** | **1.106** | 0.894 | 0.913 | **0.819** | **0.963** |
| UGU | C | 0.125 | 0.063 | 1.492 | 1.449 | **0** | **0.4** | **1.181** | **0.894** | 1.106 | 1.087 | **1.181** | **1.037** |
| GAC | D | **1.221** | **1.288** | **0.846** | **0.798** | **0.526** | **0.589** | **0.906** | **0.972** | **0.675** | **0.503** | **0.882** | **0.837** |
| GAU | D | **0.779** | **0.712** | **1.154** | **1.202** | **1.474** | **1.411** | **1.094** | **1.028** | **1.325** | **1.497** | **1.118** | **1.163** |
| GAA | E | **0.957** | **0.792** | **0.869** | **0.816** | **0.898** | **0.839** | **0.686** | **0.632** | **0.784** | **0.748** | **0.813** | **0.833** |
| GAG | E | **1.043** | **1.208** | **1.131** | **1.184** | **1.102** | **1.161** | **1.314** | **1.368** | **1.216** | **1.252** | **1.187** | **1.167** |
| UUC | F | **0.93** | **0.838** | **1.174** | **0.763** | **1.357** | **0.979** | **1.456** | **0.997** | **1.226** | **1.127** | **1.052** | **1.076** |
| UUU | F | **1.07** | **1.162** | **0.826** | **1.237** | **0.643** | **1.021** | **0.544** | **1.003** | **0.774** | **0.873** | **0.948** | **0.924** |
| GGA | G | **1.541** | **1.44** | 1.41 | 1.463 | **1.241** | **1.349** | **0.882** | **0.936** | **0.992** | **1.166** | **0.634** | **0.769** |
| GGC | G | 0.593 | 0.585 | **0.829** | **1.064** | 0.975 | 0.979 | **0.976** | **0.776** | **1.011** | **1.148** | **0.835** | **0.793** |
| GGG | G | **1.326** | **1.453** | 1.016 | 0.969 | **0.889** | **0.715** | **2.024** | **1.903** | **1.302** | **1.101** | **1.45** | **1.364** |
| GGU | G | **0.539** | **0.522** | **0.744** | **0.503** | **0.895** | **0.957** | **0.118** | **0.385** | **0.695** | **0.585** | 1.081 | 1.075 |
| CAC | H | 1.903 | 1.97 | **0.684** | **0.949** | 0.984 | 1.014 | **0.862** | **1.42** | **0.659** | **0.717** | **1.102** | **0.829** |
| CAU | H | 0.097 | 0.03 | **1.316** | **1.051** | 1.016 | 0.986 | **1.138** | **0.58** | **1.341** | **1.283** | **0.898** | **1.171** |
| AUA | I | 0.57 | 0.6 | **0.769** | **0.91** | **0.915** | **1.05** | **1.452** | **1.293** | **0.745** | **0.7** | **0.96** | **1.04** |
| AUC | I | 1.401 | 1.406 | **1.565** | **1.371** | **1.1** | **1.208** | **0.823** | **1.128** | 1.333 | 1.316 | **1.317** | **1.283** |
| AUU | I | 1.029 | 0.995 | **0.666** | **0.718** | **0.985** | **0.742** | **0.725** | **0.58** | **0.923** | **0.984** | **0.723** | **0.677** |
| AAA | K | **0.866** | **0.901** | **0.962** | **0.821** | **1.045** | **1.084** | **0.929** | **0.698** | **0.88** | **0.871** | **1.059** | **1.134** |
| AAG | K | **1.134** | **1.099** | **1.038** | **1.179** | **0.955** | **0.916** | **1.071** | **1.302** | **1.12** | **1.129** | **0.941** | **0.866** |
| CUA | L | **0.512** | **0.553** | **1.055** | **1.145** | **1.514** | **1.287** | **1.101** | **0.774** | **0.833** | **0.805** | **0.846** | **1.019** |
| CUC | L | **1.321** | **1.536** | **1.252** | **1.558** | **0.587** | **0.616** | **0.606** | **0.558** | **0.885** | **0.694** | **0.876** | **0.755** |
| CUG | L | **1.232** | **1.072** | **0.639** | **0.426** | **1.421** | **1.731** | **1.258** | **1.546** | **1.461** | **1.468** | **1.222** | **1.105** |
| CUU | L | **0.935** | **0.839** | **1.055** | **0.872** | **0.479** | **0.366** | **1.035** | **1.122** | **0.82** | **1.032** | **1.056** | **1.121** |
| UUA | L | 0.532 | 0.539 | 1.182 | 1.119 | **0.689** | **1.297** | **0.799** | **1.209** | **0.832** | **0.735** | 0.753 | 0.748 |
| UUG | L | 1.468 | 1.461 | 0.818 | 0.881 | **1.311** | **0.703** | **1.201** | **0.791** | **1.168** | **1.265** | 1.247 | 1.252 |
| AAC | N | **1.659** | **1.73** | **1.165** | **1.102** | **1.1** | **1.223** | **0.665** | **0.625** | **1.144** | **0.948** | **0.845** | **0.888** |
| AAU | N | **0.341** | **0.27** | **0.835** | **0.898** | **0.9** | **0.777** | **1.335** | **1.375** | **0.856** | **1.052** | **1.155** | **1.112** |
| CCA | P | 1.19 | 1.211 | **1.016** | **1.363** | **1.077** | **0.674** | **1.924** | **1.743** | **0.997** | **0.812** | **1.049** | **1.155** |
| CCC | P | **1.241** | **1.319** | **1.108** | **0.809** | **1.779** | **2.186** | **0.724** | **0.393** | **0.802** | **0.966** | **0.948** | **0.791** |
| CCG | P | **0.203** | **0.563** | 0.843 | 0.86 | **0.303** | **0.47** | **0.224** | **0.447** | **0.696** | **0.761** | **0.747** | **0.722** |
| CCU | P | **1.365** | **0.908** | 1.032 | 0.969 | **0.841** | **0.669** | **1.129** | **1.417** | **1.505** | **1.462** | **1.256** | **1.332** |
| CAA | Q | 0.896 | 0.872 | 0.927 | 0.954 | **0.567** | **0.731** | **0.81** | **0.667** | **0.873** | **0.69** | **0.992** | **1.013** |
| CAG | Q | 1.104 | 1.128 | 1.073 | 1.046 | **1.433** | **1.269** | **1.19** | **1.333** | **1.127** | **1.31** | **1.008** | **0.987** |
| AGA | R | **0.929** | **1.05** | **1.308** | **1.578** | 1.303 | 1.294 | **1.437** | **1.418** | **1.035** | **1.164** | **1.1** | **1.038** |
| AGG | R | **1.071** | **0.95** | **0.692** | **0.422** | 0.697 | 0.706 | **0.563** | **0.582** | **0.965** | **0.836** | **0.9** | **0.962** |
| CGA | R | **0.873** | **0.999** | **0.979** | **0.566** | **1.733** | **1.551** | 1.143 | 1.138 | **1.192** | **0.636** | **1.131** | **1.288** |
| CGC | R | **0.695** | **1.13** | **0.88** | **0.575** | **1.363** | **1.478** | **1.143** | **0.503** | **0.892** | **0.798** | **0.666** | **0.629** |
| CGG | R | **1.115** | **0.767** | **0.515** | **0.632** | **0.444** | **0.472** | 1.106 | 1.138 | **1.23** | **1.606** | **1.537** | **1.411** |
| CGU | R | **1.317** | **1.104** | **1.627** | **2.226** | **0.459** | **0.499** | **0.608** | **1.221** | **0.685** | **0.96** | **0.666** | **0.672** |
| AGC | S | **1.008** | **0.871** | **1.13** | **1.305** | **0.853** | **1.087** | **1.002** | **1.137** | **0.389** | **0.414** | **0.986** | **1.034** |
| AGU | S | **0.992** | **1.129** | **0.87** | **0.695** | **1.147** | **0.913** | **0.998** | **0.863** | **1.611** | **1.586** | **1.014** | **0.966** |
| UCA | S | **1.622** | **1.494** | **1.219** | **1.105** | 2.506 | 2.505 | **2.205** | **1.79** | **1.839** | **2.051** | **1.316** | **1.196** |
| UCC | S | 1.012 | 1.049 | **1.056** | **1.273** | **0.024** | **0.358** | **0.57** | **1.001** | **1.135** | **0.987** | **1.224** | **1.174** |
| UCG | S | **0.755** | **0.803** | **0.242** | **0.301** | **0.012** | **0.102** | **0.45** | **0.675** | 0.025 | 0.021 | **0.534** | **0.553** |
| UCU | S | 0.61 | 0.653 | **1.483** | **1.322** | **1.458** | **1.035** | **0.775** | **0.534** | **1.001** | **0.942** | **0.926** | **1.077** |
| ACA | T | **1** | **0.931** | **1.466** | **1.318** | **1.453** | **1.241** | **1.564** | **1.685** | **1.237** | **1.416** | **1.492** | **1.518** |
| ACC | T | 1.339 | 1.366 | **1.061** | **1.195** | **1.319** | **1.436** | **1.16** | **1.063** | **1.373** | **1.416** | **1.213** | **1.355** |
| ACG | T | **0.217** | **0.487** | **0.468** | **0.519** | **0.379** | **0.23** | 0.285 | 0.224 | **0.423** | **0.28** | **0.286** | **0.254** |
| ACU | T | **1.444** | **1.216** | **1.005** | **0.967** | **0.849** | **1.093** | **0.991** | **1.028** | **0.967** | **0.888** | **1.009** | **0.874** |
| GUA | V | 0.55 | 0.533 | 0.144 | 0.148 | **0.587** | **0.758** | **1.14** | **1.265** | **0.769** | **0.84** | **1.065** | **1.01** |
| GUC | V | **1.488** | **1.779** | **1.742** | **2.062** | **1.935** | **1.513** | **1.302** | **0.742** | 1.029 | 1.052 | **1.146** | **1.23** |
| GUG | V | **0.568** | **0.586** | **0.963** | **1.005** | 0.95 | 0.96 | 0.698 | 0.728 | **1.17** | **1.073** | **1.007** | **0.929** |
| GUU | V | **1.394** | **1.102** | **1.15** | **0.785** | **0.527** | **0.769** | **0.86** | **1.265** | 1.032 | 1.035 | **0.782** | **0.831** |
| UAC | Y | **0.394** | **0.509** | **0.41** | **0.679** | **1.056** | **1.638** | **1** | **1.201** | **1.458** | **1.023** | **1.072** | **0.941** |
| UAU | Y | **1.606** | **1.491** | **1.59** | **1.321** | **0.944** | **0.362** | **1** | **0.799** | **0.542** | **0.977** | **0.928** | **1.059** |

AA, amino acids, represented in the column by their single-letter standard abbreviation; LII, lineage II; LIV, lineage IV; genes of PPRV are named N, P, M, F, H, L; Values in bold indicate values significantly different between lineage Ii and IV within a specific gene (p < 0.05, after correction for multiple comparisons).

**Table S2.** Mean Codon Adaptation Index (CAI) and Effective Number of codons (ENC) values for PPRV gene sequences belonging to lineages II and IV.

|  | **CAI** |  | **ENC** |  |
| --- | --- | --- | --- | --- |
| **Gene** | **II** | **IV** | **II** | **IV** |
| **N** | *0.643* | *0.647* | 57.33 | 57.36 |
| **P** | 0.639 | 0.639 | *58.31* | *57.52* |
| **M** | *0.641* | *0.647* | 56.06 | 55.99 |
| **F** | *0.59* | *0.599* | *56.6* | *56.31* |
| **H** | *0.647* | *0.64* | *58.63* | *57.72* |
| **L** | *0.636* | *0.629* | 59.85 | 59.87 |

Values significantly different between lineage II (LII) and lineage IV (LIV) are presented in italic (Wilcoxon test. p-value < 0.001).

**Dataset S1:** Alignment of PPRV genomes used for this study (fasta format).

>AJ849636/Turkey/Sakarya/2000

accaaacaaagttgggtaaggatagatcttataataactatggactggcaaacttaggagtaaagatcctactgtcggggggaggaggaggagcaagatctttgactatggcgactcttcttaaaagcttagcattgttcaagaggaacaaagacaaagcgccgacggcatcaggttcaggaggggccatccgggggattaagaatgttatcatagtcccgattcccggagactcgtccatcattacccgttcaagactgctcgacaggcttgtcagattggccggagatcctgacatcaacgggtcaaagttaaccggcgtgatgatcagcatgttatctttgttcgtagagtcacccgggcaattgatacagcgaatcacagatgatccagatgttagtatccgccttgttgaggtagttcaaagtactaggtctcagtccgggttgacctttgcatcacgtggtgctgatttagacaacgaggcagacatgtatttttcaactgaggggccctcgagtggaggtaagaaaaggatcaactggtttgagaacagagaaataatagacatagaagtgcaggatccagaagagttcaatatgttgttagcctccatactagcacaagtctggatccttctggccaaggctgttacggcaccggatacggcagctgactcagaattgagaaggtgggttaaatatacacaacaaaggagagtgattggggaatttcgccttgacaaagggtggctggacgcagtccgcaacagaattgcagaagatctatcacttcggcggttcatggtatctctcatacttgacatcaagaggacccccggcaacaagccaaggattgcagaaatgatctgcgacattgacaactatattgtcgaagccggactcgccagtttcatccttactatcaagtttggtatcgaaaccatgtatcctgcattagggctccacgagtttgccggggaattgtccactattgaatccttgatgaacttgtatcaacagctaggagaggttgcaccctacatggtaattctcgagaactcaattcagaacaagtttagtgcaggagcttatcccctcctctggagctatgcgatgggtgtcggagttgggctggagaactcaatggggggcttgaactttggcaggtcatattttgaccctgcctattttcgtctcggacaggagatggtcagaagatctgcaggaaaggtcagctctgtgattgcggccgagctcggcatcacagcagaggaagccaaactagtctcggaaatcgcctcgcaggctggggacgaaagaaccgctagagggactgggcctcgacaggcgcaggtctccttcctccagcacaaaacaggagagggagagtcgtccgcaccagcaaccagagagggggtcaaagctgcgatcccaaacggatccgaagaaagggacaggaagcaaacacgctcaggaaggcccagaggagagacccccagccaactgctcctggaaatcatgccagaggatgaggtctcgcgagagtctggtcaaaaccctcgtgaggctcaaagatcggccgaggcactcttcaggctgcaggccatggccaagattctggaggaccaggaggagggagaagacaacagtcaggtctacaacgacaaggatctcctcggctaagcagacgcaccctctgtcgaaatcagtgacaagacatctcctaccagtattataaaaaacttaggacccaggtccaagcaagcacacatcgacactccaatcagtcgagcggagaccaccgatggcagaagaacaagcataccatgtcaacaaggggctggaatgtatcaagtccctcaaagcctctcccccggatctatccaccatcagagacaccattgagagctggagagaggggctcagcccctcgggccgtgcaacaccgaaccctgatacgtccgagggagatcatcagagtatcaaccaatcatgctcaccagcaatcggaccaaacaaagtctacttgtctcctggagataatctcggatttagagagatcactggcaacgactgtgaggctgggctcggaggagtccaggggaaaagatccaactctcaagtacagcgttactatgtttatagccacgggggtgaagagattgaaggactcgaggatgctgactctctcgtggttcaagcagatcctccagttgctaacgtcttcaatggaggagaggatggatctgacgacagcgatgtggactctggcccagatgatcccagcagagatactctatatgaccggggatctgttgccggcaatgatgtcgctaggtccacagatgtcgaaaaactagaaggtgctgatattcaagaggttcttaactcccagaaaggcaaaggaggaagattccaaggcgggaagaccttacgagtcccggaaatacccgatgtcaagcactccagaccatcagcccaatcaattaaaaagggcacagacgggaactcagtctcatctggaacggtgacagagtgtttatcgataagtggtgcaacccaagctgtgccagagtcaagatgggagtcatcagagcaaaatgcgtctgtggggagtgtcctcaagtctgcgaggagtgcagagacgatccaggggttgacacaagaatctggtaccatagcatcactgactcagcctaaagagaatgactccgagtatgagtatgaggatgacctatttaaagagattcaggacatccgtgcaagcattgccaagatccatgatgacaacaaaactatcctctcaaagcttgattctatactgttattgaaaggagaagtcgacactatcaagaaacaaatcagcaagcagaatataagtatatccaccattgagggccatctctccagtataatgatagctatcccgggctttgggaaggatatcaaggacccaacatccgaggttgagttgaacccagatttaagacccataataagccgtgactctggcagagccctcgcggaggtcctcaagaaacccgctgtggataggtctcagaaaattggaaccaaagccaactccagctcaaagggtcagcttcttaaggatctccagctaaaacctgtcgacaagcaggcaagctctgcaatcgggtttgtcccatccgaccatgaatcatccagaaatgtcatccgctccataatcaagtcgagcaagctaaacactgatcacaaggactatcttctagatttactaaatgatgtgaaaggctccaaggatcttaaggaattccacaagatgctaacagcaattctcgccaagcacccgtaacacaccctccagtcaccatctcatactcggctgaaaacatcctctcaatcaggctattacaaaaaacttaggagcaagggtaactgagcttcgcagacaagatgaccgagatctacgacttcgataaatcagcatgggatgtcaaagggtcaattgctcgcatagaacccaccatccaccatgacggtcgactgataccccaggtaagggtcatcgatcctggtctgggagacagaaaagatgagtgcttcatgtacctgtttctcctaggagtgattgaggataacgaccccctgtctcccccagttggaagaacctttggttctttacctctaggggtcggtaggtcaactgctaagccagaagaactactaagagaggccacagaactagatatagtggtgaggcgcactgcaggattaagtgagaaactggtattttacaacaacactccgctgtccttgttaacgccctggaagaaggttctgacaaccggaagtgtgtttagtgctaaccaggtttgcaatgcagtcaacctagtcccgcttgatactccccagagattcagggttgtgtacatgagcataactagattatcagacaatggttactatagtgtgcccagaagaatgctggagttccgctcagccaatgcagtagccttcaacatcttggttacactaagaattgaaaatggcacaagccctagaagatacatagttggctcatgggagaatccagaggtcacatttatggtacacgtgggcaactttagaagaaagaagaacgaagtatactcagctgattactgcaaaatgaagattgaaaagatgggtttagtttttgccctgggaggaataggtggaacaagtctccatattagaagcaccgggaaaatgagcaaaaccctccatgcacagctggggttcaagaaaatcttatgttaccccctaatggatgttaatgaggatcttaaccgatatctctggcgggcagagtgccgaatagtcaaaatccaagccgtcctacagccatcagtaccccaagaattccgtgtctacgatgatgtcatcatcaacgatgatcaaggcccattcaagatcccgtagttcaccctcaacatcatgacacgggtcgcaatcttgacatttctgtttcttttcccaaatgctgttgcgtgccagattcactggggcaatctatccaagatcgggattgtaggaacagggagtgccagctacaaggtgatgactaggccaagccaccaaactctggttataaagttaatgccaaatataacagccatcgacaattgtacaaagtcagagattgcagagtacaagagattgctgatcacagtgttaaagcctgtagaggatgctctgtcagtgataaccaagaatgtaagaccaattcaaactctgacacctgggcgtagaactcgccgctttgctggagctgttctggccggagtagcacttggagttgcgacagccgctcagataactgcaggagtcgcccttcatcaatcattgatgaactcccaagcaattgagagtttaaaaaccagtcttgagaagtcgaatcaggcaatagaggaaatcagacttgcaaataaggagaccatactagcagtacagggcgtccaggattatatcaacaatgagcttgtcccttctgtccatagaatgtcatgcgagctggtaggtcacaagctcggcctcaagctccttaggtactacaccgagatcctgtccatattcgggcccagccttcgagacccgatagctgccgaaatatcaatccaggcactcagttatgcattaggcggggacattaatagaatcctggacaagcttgggtatagcggtggggatttccttgccatcctagaaagcaagggaataaaggcccgggtaacatatgtggatacaagagattactttataattcttagcatcgcctacccaaccttatctgagatcaagggagtgatagttcacaagatagaagctataacatacaacattggggcacaggagtggtatactactatccctaaatatgtagccactcaggggtatctgatatcgaactttgatgagacgtcatgcgtattcactccggatgggacagtttgcagccagaatgcgttgtacccaatgagcccattgctccaggaatgtttccaggggtcaacaaaatcgtgcgccagaaccctagtttcagggaccataagtaatagatttatcctatcaaaagggaacctgattgcaaattgtgcgtcagttctgtgcaaatgctacacaacagagacagtcattagccaagatcccgacaaactactaactgttgtagcatccgacaagtgtcctgtagttgaggtggatggagtgacaatacaggtcggcagtcgagagtatccggattctgtatacctacacaaaatagacttaggtccagccatctccctagaaaaactggatgtaggcaccaatttaggcaatgcagtcacaagactggagaatgcaaaggagctcctagatgcatcagaccaaatactgaagactgttaaaggggtacctttcggtgggaatatgtacatagcactggcagcttgcattggggtatccctagggcttgtcacattaatatgctgctgcaaggggaggtgtaagaacaaggaagttcccatctccaaaatcaacccagggctcaaacccgacctgaccgggacctcaaagtcgtacgtgagatcactgtagtcagaatcacctgaatcattcggcataacacacatacatgtacgacacaagcagtcagaggacgcaggaaacccagcctccgatcacccacctgaccccaccccacactccaccacacattagtcatcaaacaaaacttaggacgaaaggtcaatcaccatgtccgcacaaagggaaaggatcaatgccttctacaaagacaatcctcacaataagaaccatagggtgatcctggatagagaacgcttggtcatcgaaagaccctacatcttgcttggagtcctgctggtaatgttcctgagtctaatcggactgctggccattgcagggatcaggcttcaccgggccaccgttggaacatcagagatccagagtcggctgaataccaatattgagttgaccgaatctattgatcaccaaactaaggatgtcttaactccccttttcaaaatcattggcgatgaagtcggcatcagaattccacagaaattcagtgatcttgtcaagttcatctccgataagattaaattcctcaaccctgatagagagtatgatttcagggatctccggtggtgcatgaatccccccgagagagtcaaaattaattttgatcagttttgtgagtacaaggctgcggttaagtcaattgaacatatatttgaatcaccactcaacaagtcaaaaaagctgcaatcattgactcttgggcccggaacaggctgtctaggcaggacagtaacaagagctcatttctcagaacttacactgaccttaatggacctggatctagagatgaagcacaacgtatcctcagtgtttaccgtagttgaagagggattattcggaagaacatataccgtctggagatccgatgccagggatccgagcaccgatctaggtattggccactttttaagagtcttcgagattggactggtaagagatctcgggctgggtccccctgtttttcatatgaccaactatctcacagtgaacatgagtgatgactatcggagatgtcttttagcggtaggggagttaaagttgacagccctatgcagctcatctgagactgtgacactgggcgagagaggagttccaaagagggagcctcttgtagttgtgatactcaacctagctgggcccactctagggggcgaactatacagtgtcttgcctacctctgatctcatggtggagaaactctatttatcttcacatagagggattatcaaagatgacgaggccaattgggtagtgccgtctaccgatgttcgtgatcttcaaaacaaaggagaatgtttggtggaggcatgcaagactcgacctccttcattttgcaatggcacaggatcaggcccgtggtcagaggggagaatccctgcttacggggtgatcagggtcagtcttgacttagctagtgacccgggtgtagttatcacttcagtgtttggcccactgatacctcacctatctggcatggatctttacaacaacccgttttctagagctgtatggttggctgtaccaccttatgagcagtcatttctaggaatgattaatacgattggattccctaacagagcagaggttatgccgcacattttgaccacagagatcagaggccctcggggtcgttgccatgttcccatagaattgtctcgcagggttgatgacgacatcaagattgggtccaacatggtcatattgccgacgatagacctgagatatattacagccacttatgatgtttctaggagcgagcatgctatcgtgtactatatctatgacacaggtcgctcatcatcttacttctacccagttcggctgaacttcaaaggcaatcctctctctctgaggatagagtgtttcccttggcgtcataaggtgtggtgctaccatgattgtcttatatacaacaccataacagatgaagaagtccatacgagagggctgaccggtatagaggtaacatgtaatccagtctgagcagagctacgaccgccactcaggcagtcccttgagtcgccaccgaatccaagcagcacagcctgggacactcaacagcacagcccagccaacaatgttataaaaaacttaggagccagggctgtaggggccatggactccctatcagtcaatcaggtcttgtaccctgaggtccatctagatagccctattgtcacaaacaaactagttgccatccttgagtactcgggtatcgaccataactatgttcttgaagaccagacccttgtcaagaatattaggtatagactggggtgcggtttttcaaatcaaatgatcatcaataacaggggggtgggtgaaacagtcaattctaaactcaaaagttacccccataatcgtcatatcatatacccggattgcaataaggagttgttttgtatcaaagatagctgcatatctaagaagctctcggagctattcaagaagggtaattccttgtactctaagataagtcaccaggtactggattgtcttaagagagtcaatgggaaattaggcctgggcacagatcttacctatggtctgaaggagggtatccttgacctagggttgcacatgcatagctctcaatggttcgagactttcctgttctggttcactatcaagacagagatgagatcaatgatcaaagaacagtcccatatatgccacaagaggaggtataacccgatttttgtgtcgggggatgcattcgaggtgcttgtatcacgagacctcgttgtgataattgataagaatacccagtatgtcttctacttaacatttgagctggtccttatgtattgtgatgtcatagagggcagacttatgactgagacagccatggccatagatcagagatattcagaacttctaagccgagtcagatacttgtgggatcttattgatgggttcttcccaacactaggcaacaccacataccaagttgttgccctgcttgaaccattatcgttggcttatcttcaacttcaggatgtcactctggagttaagaggtgcttttttagaccattgcttcaaagaactctatgagatattggagcattgcggcattgacacagaaggcacctacaattccatcactgaaggattggattacgtattcatcacccacgatatacatctaactggggagattttttcattctttcggagtttcggacacccccgtctagaagcagtcaccgctgcagaaaatgtcagaaaacatatgaaccaaccgaaggtaatcagttatgagactatgatgaaagggcacgcagtattttgcgggataatcataaatggttttagggaccgacatggcggcagctggccccctgttgcattgccagaacatgcttctgctgcgatccggaatgcgcaggcatcaggtgagggactgacccatgacctgtgtatagacaattggaagtcctttgttggattcaaatttggctgctttatgccactcagcctagatagtgatttgaccatgtacctcaaagacaaggcattggctgcactgaagaatgagtgggattcagtttacccgaaggaatacctccgttataacccacctagggggacagagtcaaggcgattggtggaggtgttcctgaatgactccagctttgatccttataacatgataatgtacgtggtgaatggctcctaccttaaagaccctgagtttaatctctcatacagcctaaaagagaaggagataaaggagacagggcggttgtttgccaaaatgacctataagatgcgggcctgtcaggtaatcgctgaaaatctgatatcgaatggtgttgggaagtatttccgagacaatgggatggcaaaggacgagcatgacctaacaaaagcccttcacactctggcagtctcaggtgttcccaagaacaacaaagataaccaccgaggtggccctcccagaagaacaacaaaccgagaggtgagatcaagccaagacaccaaaacacaaaatagagacaaggtccaaggggggcctgtgtacaactatttgcgatgccaaccaatcagccctgatcagggcgagtcatacgagactgttagtgcattcatcaccgctgaccttaagaagtattgcctgaattggagatacgagacaatcagcatatttgcacagaggctgaatgaaatatatggactaccatccttctttcaatggttacacaggattttggaaaaatccgtactctacgtcagtgacccacattgccctcccgacctagataatcatatccctctggacagtgtccctaatgcccaaatattcattaagtaccccatgggcggaatagaaggttattgccaaaaactatggacaatcagtactataccatacttgtatctggcagcctatgagagcggagtaagaatcgcctcactagtgcagggtgacaatcagacaatcgcagtgacaaaaagagttccaagttcttggccttattcactaaaaaagagggaggcatccaaagccgctcaaaattacttcgtagtcttaaggcaaagattgcacgatgtaggtcatcacttaaaggctaatgaaaccatagtatcttctcacttttttgtatattccaaggggatttattatgacggcctgctagtctcacaatcactaaagagcatcgccagatgtgtcttctggtccgagactatagtggatgaaaccagagcggcttgcagcaatattgcaacaactgtcgccaagagtatagagaggggttatgatagataccttgcatattctttgaatatcctcaagattttccaacagatccttatatcccttaacttcactattaacacaacaatgactcaggatgtcgtggcaccgatcatcgaaaacggggatttgctgataaggatggcactcttgccagcacccatcgggggtctcaattatcttaacatgagcaggttatttgtgagaaatatcggtgacccggtcacctcctctatagccgacctgaagaggatgatagatgctgggctaatgccagaagaaacattgcatcaagtgatgacccagaccccgggagaatcatcctacctcgattgggcaagtgacccttattctgccaacctaccctgcgtacagagtataactcgccttctcaaaaacatcactgcacgatatattttaatcagcagcccaaacccgatgctgaaaggattgtttcatgaggggagtagagatgaagacgaggagcttgcaagtttcttaatggatcggcatataattgttccaagagctgcacatgaaatcttagaccatagcataaccggggcaagagaagctatagccgggatgttggacaccaccaagggtctgattagaacaagtatgaaacggggtggcctcacccctcgagtcttagcccgcctttccaattatgattatgaacaattccgatccgggataacactattgacaaagaaagggcagtgttatctcattgacaaggactcgtgctcggtgcagctcgctatagctctgaggagccacatgtgggctaggttggctcgcgggagaccaatctatgggttggaggtgcccgatatattggaatcgatgaacggctaccttatcaaacgccacgagtcttgtgctatctgtgagacgggctcaagtcactacggatggttttttgtccctgcagggtgccagcttgacgatgtatctagagagacttcggctcttcgtgtaccttatgtcggatcaacaactgaggaaaggacagacatgaaacttgcttttgttaggtctccaagtcgatccctcaagtcagcagtcagaattgcaacagtttactcatgggcctacggggatgatgagaaatcatggggtgaagcctggatgctagctaggcagagagccaacatcaccttagatgaattgagaatgatcactccagtctctacatccaccaacctagcccaccggttgagggatcggagcacccaggtgaaatactcggggacatcccttgtgagggttgcaagatacacaaccatctccaatgataacctgtcatttgtgatatctgagaaaaaagtggataccaacttcatttatcagcaagggatgctgctcggtcttgggatccttgagaacctctttaggctagaggccaccaccggagtatccaacacagtgctacacctgcacgtggaaacagaatgttgtgttgtacctatggtggatcacccaaggataccgagtctccgtaatataaaagttacggatgagctatgcacaaaccctttgatctacgacaggtctcccatcatagagcacgatgcaacccgactatactcacagagccacaggagacatctggtggagtttgttacctggtcaacaagccagctttaccatatactggctaagtctacagcaatgtccatgattgagttgatcacgagattcgagaaagatcacatgaatgaaatagccgccctgattggcgatgacgacatcaacagcttcatcacagaatttttgctagtagagcctagattatttatagtttaccttggtcagtgtgctgccatcaattgggcctttgatatacattatcatcggccctcgggcaagcaccagatgggggaactcctctactctctgctctctcggatgagcaaaggagtatataagatcttcaccaatgctttgagccaccccaaagtttacaagaaattctggcgaagtggtgtcattgagccgattcatggcccatccctggatacacagaatttacatgtcactgtctgtgacatgatatatgggtcctatgtcacctatttggatcttttgctgaatgatgagctagatgattacccgtatttactctgcgagagtgatgaggacgtagtcacagacaggttcgacaacattcaagccaaacatctctgtgtattggccgatgtatattgcagctccaagaggtgtccttcgataatcgggatgtctcctatagaaaaatgtaccattctcacacattacatcaagggagaatcagtacaatccccgtcagggacctcatggaacactgatccccttgtagtagatcattactcatgctctctgacctaccttcgctgcggttccatcaaacaaatcaggttgagagtggatcctgggtttgtattcgaggcgttgacagacgtcgactttaaacagcctcgcaaggctaagttagatatatcggttgttgggttgactgatttctctcccccttgcgacaacgttggtgattttctagggactatcaacacattgaggcacaatctgcctgtcaccggaaccggggtctcgaactatgaagtccacgcttatcgtagaattggcctgaattcatcagcatgttataaagccgtagagatctccacgttaatcaggccatccttagaagtcggagagcatggattgttcctaggagaaggttccggttcaatgctggctgcgtacaaggaagttcttaaattagcaaattgttattacaacagcggggtaacagcggagggcagagccggacagagggaaatatctccctatccttcagagatgagcctagtagagaatcaaatggggatagagcggagtgttaaagtgctgttcaacggcaaacctgaggtaacttgggtggggaccaccgattgctacaagtacataatcagtaacatccaaacctctagtctgggtttcatacattcagatattgagacactcccaaccaaagacgctgttgagaaattggaagaatttgcctctatcctatccttatccttgattttgggaaaaatcggctctattacagttgtcaaagttatgcccattagcggggattttacccaaggtttcatagcctatgctatccaatattttagggagagcctgcttgcctatccgagatatagtaacttcatctcgactgagtgttaccttattatgataggattaaaggccaatcgattgataaacccagaagccattaagcaaagcataatcagagcgggggtcaggactgcaccaggacttgtgagccatatattatcagggaaacagaaaggttgtattcaatctttcttgggtgatccttatatccaaggagacttcaataagcaccttaaatctctaacccctattgagaaaatcctggtaaattgtggtctctcgatcaatggcacaaaaatctgtagagatctaatccaccatgatatcgcctccggtccagacggtcttatgagctctacaattattttatatagggagttggctcatttcaaagataatataaggagtcagcacggaatgttccacccctacccagtactggccaacagcaggcagcgtgaattaattcttcgaatagccaagaaattctgggggtatgtcttgctatattctgatgacccggcactaatcagacaaacaatcaagaacttgaaacggaatcatctaacctttgacttacatagtaacccgtttattaaagggctatccaaagctgagaaactgctggtgcggacgagttcactaagaagggaatggttgttcaccctcgatacgaaagaagtgaaagagtggttcaaattggtaggttacagtgcactcatcagaggctagttagctatacatctgcccccttttcctccgccataagaccccactgatgatccaaaagattaaagaaaactacatattggataagtatctattcccagctttgtctggt

>EU267274/Nigeria/Taraba/1976

accaaacaaagttgggtaaggatagatcttataataactatggactggcaaacttaggagtaaagatcctactgtcggggagaggaggaggagcaagatctttgaccatggcgactctccttaaaagcttagcattgttcaagaggaacaaagacaaagcgcctactgcgtcgggttcaggaggggccatccgggggattaagaatgttatcatagtccctatccccggggactcatccatcattacccgttcaagactgctcgacaggcttgtcagattggccggagatcctgacatcaacgggtcaaagctgaccggcgtgatgatcagcatgttatctttgttcgtggagtcacccgggcaattgatacagcggatcacagatgatccagatgtcagcatccgccttgttgaggtagttcaaagtactaggtcccagtccgggttgacctttgcatcacgtggtgctgacttggacaatgaggcagatatgtatttttcaactgaaggaccctcgagtggatgtaagaaaaggatcaactggtttgagaacagagaaataatagacatagaagtgcaagatgcagaagagttcaatatgttgttagcctccatcttagcacaagtttggatcctcctggccaaggcggttacggcaccagatacggcagccgactcagaaccgagaaggtgggttaaatacacacaacaaaggagagtgattggggaatttcgccttgacaaagggtggctggacgcagtccgcaacaggattgcagaagatctatcactccggcggttcatggtatctctcatacttgacatcaagaggacccccggtaacaagccaaggattgcagaaatgatctgcgacattgacaactatattgtcgaagccggactcgccagtttcattcttactatcaagtttggtattgaaaccatgtatcctgcactgggccttcacgagttcgccggggaattgtccactattgaatccttgatgaacttgtatcaacagctaggagaggttgcaccctacatggtaattctagagaactcaattcagaacaagtttagtgcaggagcctatcccctcctctggagctatgcgatgggtgtcggagtcgagttggagaactcaatggggggcttgaactttggcaggtcatattttgacccggcctattttcgtctcggacaggagatggtcagaagatctgcaggaaaggtcagctccgtaatcgcggctgagcttggcatcacagcagaggaagccaaactagtctcggaaatcgcctcgcagactggggaagaacgaaccgttagagggactgggcctcgtcaggcgcaggtttccttcctccagcataaaatggatgagggagagtcgcctacaccagcgaccaaagaagaagtcaaagctgcgattccaaatgggtctgaaggaagggacataaagcgaacacgctcagggaagcccagaggagaaactcccggccaactgcttccggagatcatgccagaggatgaagtctcgcgagagtccagtcaaaaccctcgtgaggctcaaagatcggctgaggcactcttcaggctgcaggccatggccaagattctggaggaccaggaggagggagaagacaacagtcagatccacaacgacaaggatctcctcagttgagcagatgcaccctccgtctgaatcagtgacaagacatcacctgccagtattataaaaaacttaggacccaggtccaagcaaccgcacatcgacaccccagtcaatcgagcggagaccaccgatggcagaagaacaagcataccatgtcaacaagggactggaatgtatcaagtctctcaaagcctctcccccggatctatccaccatcaaagatgcccttgagagctggagagaggggcctagccccccaggccgtgcaacaccgaaccctgatacgcccgagggagaccatcagaatatcaaccaatcatgctcaccagcaatcggatcagacaaagtcgacatgtctcctgaagataatctcggatttagagagatcacttgtactgacagtgaggccgggctcggaggagttcaggacaaacgatccgactctcaagtacagcgttactatgtttatagccacgggggtgaagagattgaaggactcgaggatgctgactctctcgtggttcaagcaaatcctccggttgctaacaccttcaatggaggagaggatggatctgacaacagcgatgtggactctggcccagatgatcccggcagagatcctctatatgaccggggacctgctgccggcaatgatgtctctaggtcaacagatgtcgaaaaattagaaggtgatgacattcaagaagttcttaactcccagaaaagtaaaggaggaagattccaaggcgggaaaatcttgcgagtcccggaaatacccgatgtcaagaactccagaccatcagcccaatcaattaaaaagggcacagacgggagctcagccttatctggaacggtgacagagtgttcatcgataagtggtgcaacccaagctgtgccagagtccagatgggagtcatcagagcgaaatgcgtctgtggggagtgtcctcaaatctgcgaggagtgcaaagacgatcccggggttaacacaagaatctggtaccatagcatcactgactcagcctaaagagaatgactccgagtatgagtatgaggatgacctattcactgagatgcaggacattcgtgcaagcattgctaagatccatgatgacaacaaaactatcctctctagacttgattctctactgttattgaaaggagaaatcgatactatcaagaaacaaatcagcaaacaaaatataagtatatctaccattgagggccatctatccagtataatgatagccatcccaggctttgggaaggacatcaaggacccaacatctgaggttgagttgaacccagatttgagacctataatcagccgtgattctggcagggctcttgcggaggtcctcaagaaacccgctgttgataggtctcagaaaagcggaatcaaagtcaactccggttcaaagggtcagctccttaaggatctccagctaaaacctgtcgacaaacaggcaagctctgcaatcgggtttgttccatccgaccatgaatcatccaggagtgtcatccgctccataatcaagtcgagcaagcttaacattgatcacaaggactatcttctagatttactgaatgatgtgaaaggctctaaggatcttaaggaattccacaagatgctaacagccattcttgcgaagcagccgtaacacatcccataatcaacatctcatactcggttgaaaacatcccctcaatcaggctattacaaaaaacttaggagcaagggcaactgagcttcacagacaagatgaccgagatctacgatttcgataaatcagcatgggatgtcaaagggtcaattgctcgcatagaacccaccacctatcacgacggccgactgataccccaggtgagggtcatcgaccctggtctgggagacagaaaagatgagtgcttcatgtacctgtttcttctaggagtgattgaggataacgaccccctgtctcccccagtcgggagaaccttcgggtctttacctctaggggtcggtaggtcaactgctaagccagaggaactactaagggaggccacagaactagatatagtggtgaggcgcacggcaggactaaatgagaaactggtattttacaacaacactccgctgtctttgttaacaccctggaggaaagtcttgacaaccggaagtgtgttcagcgccaaccaggtttgcaatgcagtcaacctagtcccacttgatactccccagagattcagggttgtgtacatgagtataactagattgtcagataatggttattatagtgttcccagaagaatgttggagttccgctcagccaatgcagtcgccttcaatatcttggttacactgagaattgaaaatggcacaaaccctaggagatacatagtcggctcatgggagaatccagaagtcacatttatggtacacgtgggcaactttagaagaaagaagaacgaagtatactctgctgattattgcaaaatgaagattgaaaagatgggtctagtttttgccttgggaggaataggtggaacaagtctccatattcgaagcacagggaaaatgagcaagaccctccatgcacagctggggttcaagaaaatcctatgctaccccctaatggatatcaatgaggatcttaaccgatatctctggcgggcagagtgccgaatagttaaaatccaggccgtcttacagccatcagtaccccaagaattccgtgtctatgatgatgtcatcatcaacgatgatcaaggcctgttcaagatcctgtaattcacctgcaacatcatgacacgggtcgcaaccttggtatttctgtttcttttcccaaacactgtcgcgtgccagattcactggggcaatctatccaagatcgggattgtaggaacagggagtgccagttacaaggtgatgactaggccaagccaccaaaccctggtcataaagttgatgccaaatataacagccattgacaattgtacgaaatcagagatttcagagtacaaaagattgctgatcacagtgttaaagcctgtagaggatgctctgtcagtgataaccaagaatgtaagaccaattcaaactctaacacctgggcgcaggacccgccgttttgccggagctgttctggccggagtagcacttggagtcgcgacggctgctcaaataactgccggagtcgcacttcatcagtcattgatgaattcccaagcaattgaaagtttaaaaaccagtcttgagaagtcgaatcaggcaatagaagaaatcagacttgcaaataaggagaccatactggcagtacagggcgtccaagattatatcaacaatgagcttgtcccctctgttcatagaatgtcatgtgagctggtaggtcacaaactcagtctcaagctccttaggtattataccgagatcctgtctatattcgggcctagccttcgagacccgatagctgctgaaatatcaatccaggcactcagctatgcactaggcggagacatcaataaaatcctggacaagcttgggtatagcggcggggatttccttgctattctagaaagcaaggggataaaggcccgggtgacatatgtggacacaagagattactttataattcttagcatagcctacccaaccttatctgagatcaagggggtgatagttcataagatagaagctatatcatacaacattggggcacaggaatggtatactactatccctaaatatgtagccactcagggatatctgatatcgaatttcgatgagacgtcatgcgtcttcactccagaggggacagtctgcagccagaatgcgttgtatccaatgagcccattgcttcaggaatgtttcagggggtcgacaaaatcgtgcgccagaaccctagtttcagggaccacaagtaatagatttatcctatcaaaagggaacttgattgcaaattgtgcgtcagttttgtgcaagtgttacacaacggagacagtcatcaaccaagatcctgataaactactaactgttatagcctccgataagtgtcccgtagttgaggtggatggagtgacaatacaggtcggcagtcgagagtacccagattctgtatacctacataaaatagacttaggcccagccatctctctggaaaaactggatgtaggcaccaatttaggcaatgcagtcacaagactggagaatgcaaaggagctcctagatgcatcagaccagatactgaagactgttaaaggggtacccttcagtggcaatatgtacatagcactggcagcttgcattggggtatccctagggcttgtcacattaatatgctgctgtaaggggagatgtaagaacaaggagattcctgcctccaaaatcaatccagggctcaaacccgacctgaccgggacttcaaagtcgtacgtgagatcactgtagtcagagtaacccgaatcatccagcatcacacgtatacatgtgcgacacaagcagtcagaggacacagaagacccaacttccgatcaccgatcagacccttctctacgccctactacacattggtcatcaaacaaaacttaggacgaaaggtcaatcaccatgtccgctcaaagggagaggatcaatgccttctacaaagacaatcctcacaataagaaccataggataatcctggatagggaacgcttaactattgaaagaccctacatcttacttggagtcctgctggtaatgttcctgagtctaatcgggctgctggccattgcagggatcaggcttcaccgggccaccgttggaactgcggagatccagagtcggctgaagaccaacattgagttgaccgaatccattgatcatcaaactaaggatgtcttaactcccctgtttaaaatcattggtgatgaagtcggcatcagaattccacagaagttcagtgatcttgtcaagttcatctccgataagattaagttcctcaaccctgacagggaatacgattttagggatctccggtggtgtatgaacccccctgagagagtcaaaattaactttgatcagttttgtgaatacaaagccgcggtcaaatcagttgaacatatatttgagtcatcactcaacaggtcagaaatgttgcgactattgactcttgggcccggaacaggctgtctcggcaggacagtaacaagagctcagttctcagagctcacgctgaccttgatggacctggatctcgagatgaagcacaacgtgtcctcagtgtttaccgtagtcgaagagggattattcggaagaacatatactgtctggagatccgacaccgggaaaccgagcaccagtccaggtattggccattttttaagagtcttcgagatcgggctggtgagagatctcgagctgggtgcccctattttccatatgaccaactacctcacagtgaacatgagtgatgactatcggagctgtcttttagcggtaggggagttgaagctgacagccctatgcaccccatctgagactgtgactctgagtgagagaggaattccaaagagagagcctcttgtggttgtgatactcaacctggctgggcctactctagggggcgaactatacagtgtcttgcctacctctgacctcatggtggagaaactctatttatcctcacatagagggattatcaaagataacgaggccaattgggtagtaccgtctaccgatgttcgtgatcttcaaaacaaaggagaatgtctggtggaagcatgcaagactcgacctccttcattttgcaatggcacaggaataggcccatggtcagaggggagaatccctgcctacggagtgatcagggtcagtcttgacttagctagtgacccaggtgtggttatcacttcagtgtttggcccattgatacctcacctatccggcatggatctttacaacaatccgttttcaagagctgcatggttagctgtaccaccttatgagcagtcatttctaggaatgataaatacaattggcttcccggacagagtagaggttatgccgcacattttgaccacagagatcagagggcctcgaggtcgttgtcatattcctatagagttgtcccgcaggattgatgatgatatcaagatcgggtccaacatggttgtattgccgacgaaggacctgaggtacataacagccacttatgatgtttccaggagcgagcatgcaatcgtgtactatatctatgacacgggtcgctcatcatcttacttctacccagttcgattgaatttcaggggcaatcctctctctctgaggatagagtgttttccctggtatcataaggtgtggtgctaccatgattgccttatatacaacaccataacaaacgaagaagtccatatgagagggctgaccggtatagaggtaacatgtaatccagtctgagtagagctacaaccatcgctcaagcaggcctccgagccatcccctagtccaagaagcatagcctgggacactcagcagcacaacccagccaacaatgttataaaaaacttaggagccaaggttgtaggagccatggactcactatcagtcaatcaggtcttgtaccctgaggtccatctagatagccctattgtcacaaacaaactagttgccatccttgaatactcggggatcgaccacaactatgttcttgaagaccagacccttatcaagaatattagatatagactggggtgcggtttttcaaatcaaatgatcatcaataataggggggtaggcgaaacagtcaattccaaacttaaaagttacccccgtaattgtcatatcatatacccagactgcaataaggatttgttttgtatcaaagatagctgcatatctagaaagctctcggagctattcaagaagggtaattccttgtactctaagataagtcaccaggtactggattgtcttaagagagtcaacgggaaattaggcctgggcacagatcttactcacggcctgaaggagggtatcatcgacttggggttgcacatgcatagctctcaatggttcgagacctttctgttctggttcactatcaagacagagatgagatcaatgattaaagaacagtcccatatatgccacaagaggaggtataacccaatttttgtgtcgggagatgcattcgaggtgctcgtatcacgagacctcgttgtgataattgataagaacacccagtatgtcttctacctgacatttgagctggtccttatgtattgtgatgtcatagagggcagacttatgacggagacagccatggctatagaccagagatattcagagcttctaaaccgggtcagatacttgtgggatctcattgacgggttcttcccaacactgggtaacaccacataccaagttgttgctctgcttgaaccactgtcgttagcttatcttcaacttcaggatgtcactctagagttaagaggtgcttttttggaccactgcttcaaagaactttatgagatactggagcattgtggcattgacacggaaggtacctacaattccatcactgaaggattggattacgtatttatcacccacgatatacacttaactggggagattttttcatttttccggagtttcggacacccccgcctcgaagcggtcaccgctgcagagaatgtcagaaaacatatgaaccaaccgaaggtaatcagttatgagactatgatgaaagggcatgcagtattttgcgggataatcataaatggttttagggaccggcacggcggcagctggccccctgttgcattgccagaacatgcttctgctgcgatccggaatgcgcaggcatccggtgaaggactgacccatgacctgtgtatagacaactggaagtcctttgttggattcaaatttggctgcttcatgccgctcagcctggatagtgatttgaccatgtacctcaaagacaaagcactggctgcactgaagaatgagtgggattcagtttacccgaaagaatacctccgttataatccacctagagggacagagtcaaggcgactggtggaggtgttcctgaatgactccagctttgatccttataacatgataatgtacgtggtgaatggctcctaccttaaagaccctgagtttaacctctcatacagcttaaaagagaaggagataaaagagacagggcggttgtttgccaaaatgacctacaagatgcgggcctgtcaggtaattgctgaaaatctaatatcaaatggtgtcgggaagtatttccgagacaatgggatggcaaaagacgagcatgacctaacaaaggcccttcacactctggcagtctcaggtgttcccaagaacaacaaagataaccaccgaggtgggcctcccagaaggaccgcaagccgagagatgagatcaagccaagccatcaacacacaaaatagagacaggatccaagggggccctatgtacaactacttgcgatgccaaccgaccagctctgatcagggtgagtcatacgagactgttagtgcattcatcaccgctgaccttaagaaatattgcctaaattggagatacgagacaatcagcatatttgcacagagactgaatgaaatatatgggttgccatccttctttcaatggttacacagggtattggaaaaatccgtgctctacgtcagtgacccgcattgccctcccgacttagatgatcatatccccctggacagtgtccctaatgcccaaatattcatcaagtacccaatgggcggaatagaaggttattgtcaaaaactatggacaatcagtactataccatacctgtatctggcagcctatgagagcggagtaagaattgcctcactagtgcaaggtgacaatcagacaattgcagtgacaaaaagagttccaagttcttggccttattcactaaaaaagagggaggcatctaaagcagctcaaaattacttcgtggtcttaaggcaaaggttgcacgatgtaggtcatcacttaaaggctaatgagaccatagtatcttctcacttttttgtatactctaaagggatttattatgacggcctgttagtctcacaatcactaaagagcatcgccagatgtgtcttctggtccgagactattgtagatgaaaccagagcggcctgcagcaatattgcaacaactatcgccaagagcatagagaggggttatgataggtacctcgcatactctttgaatatcctcaaaattttccaacagatccttatatcccttgacttcacgattaacacaactatgactcaagatgtcgtggcaccgatcatcgagaacggtgatttactaataaggatggcactcttgccagcacccattgggggtctcaattatcttaacatgagcaggttgtttgtgagaaatatcggtgacccggtcacttcctccatagccgacctgaagaggatgatagacgctgggctaatgccagaagaaacattgcatcaagtgatgacccagaccccgggagaatcatcttacctcgattgggcaagtgacccttattctgccaacctaacctgcgtacagagtataactcgccttctcaagaacatcactgcacggtacatcttaatcagcagcccaaatccgatgctgaaaggattgtttcatgaggggagtagagatgaagacgaggagcttgcgagtttcttgatggatcggcatataattgttccgagagccgcacatgaaatcttagaccacagcatcaccggagcaagagaagctatagccgggatgttggacaccaccaaaggtctgattagaacaagtatgaaacggggtggcctcacccctcgagtattagcccgcctttccaattatgattatgaacaattcagatccggaataacactattgacaaagaaagggcaatgttatctcattgacaaggactcgtgctcggtgcagctcgctatagctctgaggggccacatgtgggccaggttagctcgcgggagacctatctatgggttggaggtgcctgatgtactggaatcgatgaacggctaccttatcaaacgccacgagtcttgtgccatctgcgaaacgggctcaagtcactacgggtggtttttcgtccctgcagggtgccagcttgacgatgtctcaagagagacttcggctcttcgtgtaccttatgtcggatcaaccactgaggaaaggacagatatgaaacttgctttcgttagatctccaagccgatccctcaaatcagcagtcaggattgccacagtttactcatgggcctacggggatgatgagaaatcatggagtgaagcttggatgctagctaggcagagagctgatatcaccttagatgaactgagaatgatcactccagtctctacatccaccaacctagcccatcggttgagggatcggagcacccaggtgaaatattcggggacatcccttgtgagggttgcaagatacacaaccatctccaatgacaatttgtcatttgtgatatctgagaaaaaagtagataccaacttcatttaccagcaagggatgctgctcggtcttgggatccttgagaatctcttcaggttagaggccaccacaggggtatccaacacagtgctacacctgcacgtggaaacagaatgttgtgttgtacctatggttgatcacccaaggataccgagtctccgtaatattaaagttacgaatgagctatgcacaaaccctctgatctacgacaggtcccccatcatagaacacgatgcaactcgattatactcacaaagccacaggagacatttggtggagtttgttacctggtcaacaagccagctttatcatatactggccaaatctacagcaatgtccatgattgagttgatcacaagatttgagaaagatcacatgaatgaaatagccgccctgattggcgatgacgacatcaacagtttcatcacagaatttttgctcgtggagcccagactgtttatagtctaccttggccagtgtgctgccatcaattgggcttttgatatacattatcatcggccctcgggcaagtaccagatgggggaactcctctactctttactctctcggatgagcaaaggagtatataagatcttcactaatgctttgagccaccccaaagtttacaagaaattttggcgaagtggtgtaattgagccgattcatggcccatccctagatacacagaatttacatgtcactgtctgtgacatgatatacggatcatacgtcacctatctggatcttttgctgaatgatgagctagatgcttacccgtacttgctctgcgagagtgatgaggacgtggtcacagacaggttcgacaacattcaagccaaacatctctgtgtattggccgatgtatactgcagctccaagagatgtccctcgataatcgggatgtctcctatagaaaaatgtaccatcctcacacattacatcaagggagaatcggtacaatccccgtccgggatctcatggaacactgatccccttgtagtagatcattactcatgctctctgacctaccttcgccgcggttccatcaaacaaatcaggttgagagtggatcctgggtttgtattcgaggcgttgacagacatcgacttcaaacagcctcgcaaggctaagttggatgcatcgattgtggggttgactgatttttctcccccttgggataacgtcggtgattttctagggactatcaacacattgaggcacaatctgcccgtcaccggaaccggggtctcgaactatgaagtccacgcttatcgtagaattggtctgaattcatcagcatgttacaaagctgtagagatctccacgttaatcaagtcatccttagaagtcggagaggatggattgttcttaggagaaggttccggttctatgctggctgcgtacaaggaagttcttaaattggcaaactgttattacaacagcggggtaacagcggagggtagagccggacagagggaaatctctccctatccctcagaggtgagcctggtagagagtcagatgggggtagagagaagtgttaaagtgctgttcaatggcaaacctgaagtaacctgggtagggaccaccgattgctacaagtatataatcagtaacattcagacctctagtctgggtttcatacactcagatattgagacactcccaaccaaggatgccgttgagaagttagaagaatttgcctctatcctatccctatccctaattttgggaaaaatcggctctattacagttgtcaaaattatgcccattagcggagattttacccaaggcttcatagcctatgccattcaatattttagggagagcctgcttgcctatccgagatatagtaacttcatctcgactgagtgttaccttattatgataggattaaaggccaatcggttgataaacccagaagccattaagcaaagcataatcagagtggggactaggactgcaccagggcttgtgagccacatattatcagagaaacagaaaggttgtattcaatcttttctgggtgatccttatatccaaggagacttcaataagcaccttaaagctctaactcctattgagaaaatcctagtaaattgtggtctctcgatcaatggcacaaaaatctgtagggatctaatccaccatgatatcgcctccggtccagacggtctgatgagctccacaattattttatatagggaactggcccatttcaaagacaatataagaagtcagcacggtatgttccacccctacccagtattggccagtagcaggcaacgtgaattaatccttcgaatagccaagaaattctgggggtatgtcttgctatattctgatgacccggcactaatcaaacaaacgatcaagaacttgaagcggaatcacctaacctttgacttacacagtaatccgtttattaagggcttatccaaagctgagaaactgctagtgcggacaagttcactcagaagggaatggttgttcactctcgatacgaaagaagtgaaagagtggttcaaattggtgggttacagtgcactcgtcagaggttaattcgcgatacatctgcccccttctcctccaccatgagactctactggcaatctaaaagattaaagaaaactacatattggataagtttctattcccagctttgtctggt

>FJ905304/China/Ngari/2007/30

accaaacaaagttgggtaaggatagatcttataataactatggactggcaaacttaggagtaaagatcctactgtcggggggaggaggaggagcaagatctctgattatggcgactctccttaaaagcttagcattgttcaaaaggaacaaagacaaagcgccgacggcatcgggttcaggaggggccatccgagggattaagaatgttatcatagtcccgattcccggagactcgtccatcactacccgttcaagactgctcgacaggcttgtcaggttggccggagatcctgacatcaacgggtcaaagctgaccggcgtgatgatcagcatgctatccttgttcgtagagtcacccgggcaattgatacagcgaatcacagatgatccagatgttagtatccgccttgttgaggtagttcaaagtactaggtcccagtccgggttgacctttgcatcacgtggtgctgatttggataacgaggcagacatgtatttttcaactgaggggccctcgagtggaggtaagaaaaggatcaactggtttgagaacagagaaataatagacatagaggtgcaggatccagaagagttcaatatgttgttagcctccatactagcacaagtctggatccttctggccaaggctgttacggcaccggatacggcagctgactcagaactgagaaggtgggttaaatacacacaacaaaggagagtgattggggaatttcgccttgataaagggtggctggacgcagtccgcaacagaattgcagaagatctatcactccggcggttcatggtatctctcatacttgacatcaagaggacccccggcaacaagccaaggattgcagaaatgatctgcgacattgacaactatattgtcgaagcaggactcgccagtttcatccttactatcaaatttggtatagaaaccatgtatcctgcattagggctccacgagtttgccggggaattgtccactattgagtccttgatgaacttgtatcaacagctaggagaggttgcaccctacatggtaattctagagaactcaattcagaacaagtttagtgcaggagcttatcccctcctctggagctatgcgatgggtgtcggagttgagctggagaactcaatggggggcttgaactttggcaggtcatattttgaccctgcctattttcgtctcggacaggagatggtcagaagatctgcaggaaaggtcagctctgtgattgcggctgagctcggcatcacagcagaggaagctaaactagtctcggaaatagcctcgcaggctggggacgaaagaaccgctagagggactgggcctcgacaggcgcaggtctccttcctccagcaccaaacaggagggggagagtcgtccgcaccagcgaccagagaaggggtcaaagctgcgatcccaaacggatccgaagaaagggacagaaagcaaacacgcccaggaaggcccagaggagagacccccggccaactgctcctggaaatcatgccagaggatgaggtttcgcgagaatctggtcaaaaccctcgtgaggctcaaagatcggccgaggcactcttcaggctgcaggccatggccaagattctggaggaccaggaggagggagaagacaacaatcaggtctacaacgacaaggatctcctcggctaagcagacgcaccctctgtcgaaatcagtgacgagacatctcctaccagtattataaaaaacttaggacccaggtccaagcaagcacacatcgacactccaaccagtcgagcggaggccaccgatggcagaagaacaagcataccatgtcaacaaggggctggaatgtatcaagtccctcaaagcctctcccccggatctatccaccatcagagataccatcgagagctggagagaggggcttagcccatcgggccgtgcaacaccgaaccctgatatgtccgagggagatcatcagaatatcaaccaaccatgctcaccagcaatcggaccaaacaaagtctacttgtctcctgaagataatctcggatttagagagatcactagcaacgactatgaggctgagctcggaggagtccagggaaaaggatccaactctcaagtacagcgttactatgtttatagccacgggggtgaagagattgaaggactcgaggatgctgactctctcgtggttcaagcagatcctccagttgctaacatattcaatggaggagaggatggatctgacgacagcgatgtggactctggcccagatgatcccggcagagatactctatatgaccggggatctgttgccagcaatgatgtcgctaggtccacagatgtcgaaaaactagaaggtgctgatattcaagaagttctcaactcccagaaaggcagaggaggaagattccaaggcgggaaaaccttacgagtcccggaaatacccgatgtcaagcactccagaccatcagcccaatcaattaaaaagggcacagacgggaactcagtctcatctggaacggtgacagagtgtttatcgataagtggtgcaacccaagctgtgccagagtcaagatgggagtcatcagagcaaaatgcgtctgtggggagtgtcctcaagtctgcggggagtgcaaagacgatccaggggtcgacacaagaatctggtaccatagcatcactgactcagcctaaagagaatgactccgagtatgagtatgaggatgacctatttacagagattcaggacatccgtgcaagcattgccaagatccatgatgacaacaaggctatcctctcaaagcttgattctatactgttattgaaaggagaagtcgacactatcaagaaacaaatcagcaagcagaatataagtatatccaccattgagggccatctctccagtataatgatagccatcccgggctttgggaaggatatcaaggacccaacatccgaggttgagttgaacccagatttaagacctataataagccgtgactctggcagagctctcgcggaggtcctcaagaaacccgctgtggataggtctcagaaaattggaaccaaagccaactccagctcaaagggtcagcttcttaaggatctccagctaaaacctgtcgacaagcaggcaagctctgcaatcgggtttgtcccatccgaccatgaatcatccagaaatgtcatccgctccataatcaagtcgagcaagctcaacattgatcacaaggactatcttctagatttactgaatgatgtgaaaggctccaaggatcttaaggaattccacaagatgctaacagcaattctcgccaagcacccgtaacacatcctccagtcaccgccccatactcggctgaaaacatcctttcaatcaggctattacaaaaaacttaggagcaagggcaactgagcttcgcagacaagatgaccgagatctacgacttcgataaatcagcatgggatgtcaaagggtcaattgcccgcatagaacccaccacctaccacgacggccgactgataccccaggtaagggtcatcgatcctggtctgggagacagaaaagatgagtgctttatgtacctgtttctcctgggagtgattgaggataacgaccccctgtctccccccgtcggaagaacctttggctctttacctctaggggtcggtaggtcaactgctaagccagaggaactactaagggaggccacagaactagacatagtggtgaggcgcactgcaggagtaaatgagaaactggtattttacaacaacactccgctgtccttgttaacgccctggaagaaagttctgacaaccggaagtgtgtttagcgctaaccaggtttgcaatgcagtcaacctagtcccgcttgatactccccagaggttcagggttgtgtacatgagcataactagattatcagacaatggttactatagtgtgcccagaagaatgctggagttccgctcagccaatgcagtagccttcaacatcttggttacactaaaaattgaaaatggcacaaaccctagaagatacatagtcggctcatgggagaattcagaggtcacatttatggtacacgtgggcaactttagaagaaagaagaacgaagtatactctgctgattactgcaaaatgaagattgaaaagatgggtttagtttttgccctgggaggaataggtggaacaagtctccatattagaagcaccgggaaaatgagcaaaaccctccatgcacagctggggttcaagaaaatcttatgttaccccctaatggatgttaatgaggatcttaaccgatatctctggcgggcagagtgccgaatagtcaaaatccaagccgtcctacagccatcagtgccccaagaattccgtgtctacgatgatgtcatcatcaacgatgatcaaggcttgttcaagatcttgtagttcatttgcaacatcatgacacgggtcgcaatcttgacatttctgtttcttctcccaaatgttgttgcgtgccagattcactggggcaatctatccaagatcgggattgtaggaacagggagtgccagctacaaggtgatgactaggccaagccaccaaactctggttataaagttaatgccaaatataacagccatcaacaattgtacaaagtcagagattgcagagtacaagagattgctgatcacagtgttaaaacctgtagaggatgctctgtcggtgataaccaagaatgtaagaccaattcaaaccctgacacctgggcgtagaacccgtcgttttgctggagctgttctggccggagtagcacttggagttgcgacagccgctcagataactgcaggagtcgctcttcatcaatcattgatgaactctcaagcaattgagagtttaaaaaccagtcttgagaagtcgaatcaggcaatagaagaaatcagacttgcaaataaggagaccatactggcagtacagggcgtccaggattatatcaacaatgagcttgtcccttctgttcatagaatgtcatgcgagctggtaggccacaagctcggcctcaagctccttaggtactacaccgagatcctgtccatattcgggcccagccttcgagacccgatagctgccgaaatatcgatccaggcactcagttatgcattaggcggagacattaataaaatcctggacaagcttgggtatagcggtggggatttccttgccatcctagagagcaagggaataaaggcccgggttacatatgtggacacaagagattactttataatccttagcatcgcctacccaaccttatctgagatcaagggagtgatagttcacaagatagaagctataacatacaacattggggcacaggagtggtatactactatccctaaatatgtagccactcaggggtatctgatatcgaactttgatgagacgtcatgcgtattcactccagaggggacagtttgcagccagaatgcgttgtacccaatgagcccattgcttcaggaatgtttcaggggatcaacaaaatcgtgcgccagaaccctagtttcagggaccataagtaatagatttatcctatcaaaagggaacctgattgcaaattgtgcgtcagttttgtgcaaatgttacacaacggagacagtcatcagccaagatcctgacaaactactaactgttgtagcatccgacaagtgtcctgtagttgaggtggatggagtgacaatacaggtcggcagtcgagagtatccggattctgtatacttacacaaaatagacttaggtccagccatctccctagaaaaactggatgtaggcaccaatttaggcaatgcagtcacaagactggagaatgcaaaggagctcctagatgcatcagaccaaatactgaagactgtcaaaggggcacctttaggcgggaacatgtacatagcactggcagcttgcattggagtatccctagggcttgtcacattaatatgctgctgtaaggggaggtgtaagaacaaggaaatccctatctccaaaatcaacccagggctcaaacccgatctgaccgggacctcaaagtcgtacgtgagatcactgtagccagaatcacctgaatcatctggcatcacacacatacatgtacgacacaagcagtctgaggacgcaggaaacccagcctccgatcacctacctgaccctactccacgctccactacacattagtcatcaaacaaaacttaggacgaaaggtcaatcaccatgtccgcacaaagggaaaggatcaatgccttctacaaagacaatcctcacaataagaaccatagggtgatcctggatagagaacgcttggtcattgaaagaccctacatcttgcttggagtcctgctggtaatgttcctgagtctaatcggactgctggccattgcagggatcaggcttcaccgggccaccgttggaacttcagagatccagagtcggctgaataccaatattaagttggccgaatctattgatcaccagactaaggatgtcttaactcccctttttaaaatcattggcgatgaagtcggcatcagaattccacagaaattcagtgatcttgtcaagttcatctccgataagattaaattcctcaaccctgatagagagtatgatttcagggatctccggtggtgcatgaatccccccgagagagtcaaaattaattttgatcagttttgtgagtacaaagctgcggttaagtcaattgaacatatatttgagtcaccactcaacaagtcaaaaaagctgcaatcattgactctcgggcccggaacaggctgtctaggcaggacagtaacaaaagctcatttctcagaacttacactgaccttaatggacctggatctagagatgaagcacaacgtgtcctcagtgtttaccgtagttgaagaggggttattcggaagaacatataccgtctggagatccgatgccagggatccgagcaccgatctaggtatcggccattttttaagagtcttcgagattggactgataagagatctcgggctgggtccccctgtttttcatatgaccaactatctcacagtgaacatgagtgatgactatcggagatgtcttttagcggtaggggagttgaagttgacagccctatgcacctcatctgagactgtgacactgagtgagagaggagctccaaagagggagcctcttgtggttgtgatacttaatctggctggacccactctagggggcgaactatacagtgtcttgcctacctccgatctcatggtggagaaactctatttatcttcacatagagggatcatcaaggatgacgaggccaattgggtagtgccgtctaccgatgttcgtgatcttcagaacaaaggtgaatgtctggtggaagcatgcaagactcgacctccttcattttgcaatggcacaggatcaggcccgtggtcagaggggagaatccctgcttacggggtgatcagggtcagtcttgacttagctagtgacccggatgtagttatcacttcagtgtttggcccactgattcctcacctatccggcatggatctttacaacaacccgttttcaagagctatatggttggctgtaccaccttatgagcagtcatttctaggaatgataaatacaattggattccctaacagagcagaggttatgccgcacattttgaccacagagatcagaggccctcggggtcgttgccatgttcccatagaattgtcccgcagggttgatgacgatatcaagatcgggtccaacatggtcatattgccgacgatggacctgaggtatattacagccacttatgatgtttccaggagcgagcatgcaatcgtgtactatatctatgacacaggtcgctcatcatcttacttctacccagttcgactgaatttcaaaggcaatcctctctctctgaggatagagtgtttcccttggcgtcataaggtgtggtgctaccatgattgtcttatatacaacaccataacagatgaagaggtccatacgagagggctgaccggtatagaggtaacatgcaatccagtttgagcaaagctacgaccgtcactcaagcagtcccttgagtcgccaccgagtccaagcagcatagcctgggacactcaacagcacagcccagccaacaatgttataaaaaacttaggagccagggttgtaggggccatggactccctatcagtcaatcaggtcttgtaccctgaggttcatctagacagccctattgtcacaaacaaactagttgccatccttgagtactcgggtatcgaccataactatgttcttgaagaccagacccttgtcaagaatattaggtatagactggggtgcggtttttcaaatcaaatgatcatcaataacaggggggtgggtgaaacagtcaattctaaacttaaaagttatccccataatcgtcatatcatatacccggattgcaataaggagttgtttggtatcaaagatagctgcatatctaagaagctctcggagctattcaagaagggtaattccttgtactctaagataagtcaccaggtactggattgtcttaagagagtcaatgggaaattaggcctgggcacagatcttacccatggtctgaaggaaggtatccttgacctagggttgcacatgcatagctctcaatggttcgagactttcctgttctggttcactatcaagacagagatgagatcaatgatcaaagaacagtcccatatatgccacaagaggaggtataacccgatttttgtatcgggggatgcattcgaggtgctcgtatcacgagacctcgttgtgataattgataagaatacccagtatgtcttctacttaacatttgagctggtccttatgtattgtgatgtcatagagggcagacttatgacggagacagccatggccatagaccagagatattcagaacttctaagccgggttagatacttgtgggatcttattgatgggttcttcccaacactaggcaacaccacataccaaattgttgctctgcttgaaccattatcgttggcttatcttcaacttcaggatgtcacgctggagttaagaggtgcttttttagaccattgcttcaaagaactctatgagatactggagcattgcggcattgacacagaaggcacctacaattccatcactgaaggactggattacgtattcatcacccacgatatacatctaactggggagattttttcatttttccggagtttcggacacccccgtctagaagcagtcaccgctgcagaaaatgtcaggaaacatatgaaccaaccgaaggtaatcagttatgagactatgatgaaaggacatgcggtattttgcgggataatcataaatggttttagggaccgacatggcggcagctggccccctgttgcattgccagaacatgcttctgctgcgatccggaatgcgcaggcatcaggtgagggactgacccatgacctgtgtatagacaactggaaatcctttgttggattcaaatttggctgctttatgccactcagcctagatagtgatttgaccatgtatctcaaagacaaggcattggctgcactgaagaatgagtgggattcagtttacccgaaagaatacctccgttataacccacctagagggacagagtcaaggcgactggtggaggtgttcctgaatgactccagctttgatccttataacatgataatgtacgtggtgaatggctcctaccttaaagaccctgagtttaatctctcatacagcctaaaagagaaggagataaaggagacagggcggttgtttgccaaaatgacttataagatgcgggcctgtcaggtaattgctgaaaatctgatatcgaatggtgttgggaagtatttccgagacaatgggatggcaaaggacgagcatgacctaacaaaagcccttcacaccctggcagtctcaggtgttcccaagaacaacaaagataaccaccgaggtgggcctcccagaaggacaacaagccgagaggtgagatcaagccaagacaccaaaacacaaaatagagacaaggtccaaggaggacctatgtacaactatttgcgatgccaaccaatcagccctgatcagggtgagtcatacgagactgttagtgcattcatcaccgctgaccttaagaagtattgcctgaattggagatacgagacaatcagcatatttgcacagaggctgaatgaaatatatggactaccatccttctttcaatggttacacaggatattggaaaaatccgtactctacgtcagtgacccacattgccctcccgatctagataatcatatccctctggacagtgtccctaatgcccaaatattcattaagtacccaatgggcggaatagaaggttattgccaaaaactatggacaatcagtactataccatacttgtatctggcagcctatgagagcggagtaagaatcgcctcactggtgcagggtgacaatcagacaatcgcagtgacaaaaagagttccaagctcttggccttattcactaaaaaagaaggaggcatccagagccgctcaaaattacttcgtagtcttaaggcaaagattgcacgatgtaggtcatcacttaaaggctaatgaaaccatagtatcttctcacttttttgtatattccaaagggatttattatgacggcctgctagtctcacaatcactgaagagcatcgccagatgtgtcttctggtccgagactatagtggatgaaaccagagcggcttgcagcaatattgcaacaactgtcgctaagagtatagagaggggttatgataggtaccttgcatattctttgaatatcctcaagattttccaacagatccttatatcccttaacttcactattaacacaacaatgactcaggatgtcgtggcaccgatcatcgagaacggtgatttgctgataaggatggcactcttgccagcacccatcggaggtctaaattatcttaacatgagcaggttatttgtgagaaatatcggtgacccggtcacctcctctatagccgacctgaagaggatgatagatgccgggctaatgccagaagaaacattgcatcaagtgatgacccagaccccgggagaatcatcctacctcgattgggcaagtgacccttattctgccaacctaccctgcgtacagagtataactcgccttctcaaaaacatcactgcacgatatattttaatcagcagcccaaacccgatgctgaggggattgtttcatgaggggagtagagatgaagacgaggagcttgcaagtttcctaatggatcggcatataattgttccaagagctgcacatgaaatcttagaccatagtataaccggggcaagagaagctatagccgggatgttggacaccaccaaaggtctgattagaacaagtatgaaacggggtggcctcacccctcgagtcttagcccgcctttccaattatgattatgaacaattccgatccgggataacactattgacaaagaaagggcagtgttatctcattgacaaggattcgtgctcggtgcagctcgctatagctctgaggagccacatgtgggctaggttggctcgcgggagaccaatctatgggttggaggtgcccgatatattagaatcgatgaacggctaccttatcaaacgccacgagtcttgtgctatctgtgaaacgggctcaagtcactacggatggtttttcgtccctgcagggtgccagcttgacgatgtatctagagagacttcggctcttcgtgtaccttatgtcggatcaacaactgaggaaaggacagacatgaaacttgcatttgttagatctccaagtagatccctcaaatcagcagttagaattgcaacagtttactcatgggcctacggggatgatgagaagtcatggagtgaagcctggatgctagctaggcagagagccaatatcaccttagatgaattgagaatgataactccagtctctacatccaccaacctagcccaccggttgagggatcggagcacccaggtgaaatactcggggacatcccttgtgagggttgcaagatacacaaccatctccaatgataacttgtcatttgtgatatctgagaaaaaagtggataccaacttcatttatcagcaagggatgttgcttggtcttgggatccttgagaacctctttaggctagaggccaccaccggagtatccaacacagtgctacacctgcacgtggaaacagaatgttgtgttgtacctatggtggatcacccaaggataccgagtctccgtaatataaaagttacggatgagctatgcacaaatcctttgatctacgacaggtctcccatcatagagcacgatgcaacccgactatactcacagagccacaggagacatttggtggagtttgttacctggtccacaagccagctttaccatatactggctaagtctacagcaatgtccatgattgaattgatcacgagattcgagaaagatcacatgaatgaaatagccgccctgattggcgatgacgacatcaacagcttcatcacagaatttttgctagtggagcctagattatttatagtttaccttggtcagtgtgctgccatcaattgggcctttgatatacattatcatcggccctcgggcaagtaccagatgggggaactcctctactctttgctctctcggatgagcaaaggagtatataagatcttcaccaatgctttgagccaccccaaagtttacaagaaattttggcgaagtggtgtcattgagccgattcatggcccatccctggatacacagaatttacatgtcactgtctgtgacatgatatatgggtcctatgtcacctatttggatcttttgctgaatgatgagctagatgattacccgtatttgctctgcgagagtgatgaggacgtggtcacagacaggttcgacaacattcaagccaaacatctctgtgtattagccgatgtatattgcagctccaagaggtgtccttcaataatcgggatgtctcctatagaaaaatgtaccattctcacacattacatcaagggagaatcagtacaatccccgtctgggacctcatggaacactgatccccttgtagtagatcattactcatgctctctgacctaccttcgccgcggttccatcaaacaaatcaggttgagggtggatcctgggtttgtattcgaggcgttgacagacgtcgaccttaaacaacctcgcaaagctaagttagatatatcggtcgttgggttgactgatttctctcccccttgcgacaacgtcggtgattttctagggactatcaacacattgaggcacaatctgcctgtcaccggaaccggggtctcgaactatgaagtccacgcttatcgtagaattggcctaaattcatcagcatgttacaaagccgtagagatctccacgttaatcaagccatccctagaagtcggagagcatggattgttcttaggagaaggttccggttcaatgctggctgcgtacaaggaagttcttaaattagcaaattgttattacaacagcggggtaacaacggagggcagagccggacagagggaaatatctccctatccttcagagatgagcctagtagagaatcaaatggggatagagcggagtgttaaagtgctgttcaacggcaaacctgaagtaacttgggtggggaccaccgattgctacaagtacataatcagtaacatccaaacctctagtctgggtttcatacattcagatattgagacgctcccaaccaaagatgctgttgagaaattagaagaatttgcctctatcctatccttatccttgattttggggaaaatcggctctattacagttgtcaaaattatgcccattagcggggattttacccaaggctttatagcctatgccatccaatattttagggagagcctgcttgcctatccgagatatagtaacttcatctcgactgagtgttaccttattatgataggattaaaggccaatcgactgataaacccagaagccattaagcaaagcataatcagagcgggggtcaggactgcaccaggacttgtgagccatatattatcagagaaacagaaaggttgtattcaatctttcttgggtgatccttatatccaaggagacttcaataagcaccttaaatctctgacccctattgagaaaatcctggtaaattgtggtctctcgatcaatggcacaaaaatctgtagagatctaatccaccatgatatcgcctccggtccagacggtcttatgagctctacaattattttatatagggaattggctcatttcaaagataatataaggagtcagcacggaatgttccacccctacccagtactggccaacagccggcaacgtgaattaatccttcgaatagccaagaaattctgggggtatgtcttgctatattctgatgacccggcactaatcagacaaacaatcaagaacttgaaacggaatcatctaacctttgacttacatagtaacccgtttattaaagggctatccaaagctgagaaactgctggtgcggacgagtccactaagaagggaatggttgttcactctcgatacgaaagaagtgaaagagtggttcaaattggtaggttacagtgcactcatcagaggctagttagctatacatctgcccccttctcctccgccatgagaccccactgacgatccagaagattaaagaaaactacatattggataagtatctattcccagctttgtctggt

>JF939201/China/Ngari/2007

accaaacaaagttgggtaaggatagatcttataataactatggactggcaaacttaggagtaaagatcctactgtcggggggaggaggaggagcaagatctctgattatggcgactctccttaaaagcttagcattgttcaaaaggaacaaagacaaagcgccgacggcatcgggttcaggaggggccatccgagggattaagaatgttatcatagtcccgattcccggagactcgtccatcactacccgttcaagactgctcgacaggcttgtcaggttggccggagatcctgacatcaacgggtcaaagctgaccggcgtgatgatcagcatgctatccttgttcgtagagtcacccgggcaattgatacagcgaatcacagatgatccagatgttagtatccgccttgttgaggtagttcaaagtactaggtcccagtccgggttgacctttgcatcacgtggtgctgatttggataacgaggcagacatgtatttttcaactgaggggccctcgagtggaggtaagaaaaggatcaactggtttgagaacagagaaataatagacatagaggtgcaggatccagaagagttcaatatgttgttagcctccatactagcacaagtctggatccttctggccaaggctgttacggcaccggatacggcagctgactcagaactgagaaggtgggttaaatacacacaacaaaggagagtgattggggaatttcgccttgacaaagggtggctggacgcagtccgcaacagaattgcagaagatctatcactccggcggttcatggtatctctcatacttgacatcaagaggacccccggcaacaagccaaggattgcagaaatgatctgcgacattgacaactatattgtcgaagcaggactcgccagtttcatccttactatcaaatttggtatagaaaccatgtatcctgcattagggctccacgagtttgccggggaattgtccactattgagtccttgatgaacttgtatcaacagctaggagaggttgcaccctacatggtaattctagagaactcaattcagaacaagtttagtgcaggagcttatcccctcctctggagctatgcgatgggtgtcggagttgagctggagaactcaatggggggcttgaactttggcaggtcatattttgaccctgcctattttcgtctcggacaggagatggtcagaagatctgcaggaaaggtcagctctgtgattgcggctgagctcggcatcacagcagaggaagctaaactagtctcggaaatagcctcgcaggctggggacgaaagaaccgctagagggactgggcctcgacaggcgcaggtctccttcctccagcaccaaacaggagggggagagtcgtccgcaccagcgaccagagaaggggtcaaagctgcgatcccaaacggatccgaagaaagggacagaaagcaaacacgcccaggaaggcccagaggagagacccccggccaactgctcctggaaatcatgccagaggatgaggtttcgcgagaatctggtcaaaaccctcgtgaggctcaaagatcggccgaggcactcttcaggctgcaggccatggccaagattctggaggaccaggaggagggagaagacaacaatcaggtccacaacgacaaggatctcctcggctaagcagacgcaccctctgtcgaaatcagtgacgagacatctcctaccagtattataaaaaacttaggacccaggtccaagcaagcacacatcgacactccaaccagtcgagcggaggccaccgatggcagaagaacaagcataccatgtcaacaaggggctggaatgtatcaagtccctcaaagcctctcccccggatctatccaccatcagagataccatcgagagctggagagaggggcttagcccatcgggccgtgcaacaccgaaccctgatatgtccgagggagatcatcagaatatcaaccaaccatgctcaccagcaatcggaccaaacaaagtctacttgtctcctgaagataatctcggatttagagagatcactagcaacgactatgaggctgagctcggaggagtccagggaaaaggatccaactctcaagtacagcgttactatgtttatagccacgggggtgaagagattgaaggactcgaggatgctgactctctcgtggttcaagcagatcctccagttgctaacatattcaatggaggagaggatggatctgacgacagcgatgtggactctggcccagatgatcccggcagagatactctatatgaccggggatctgttgccagcaatgatgtcgctaggtccacagatgtcgaaaaactagaaggtgctgatattcaagaagttctcaactcccagaaaggcagaggaggaagattccaaggcgggaaaaccttacgagtcccggaaatacccgatgtcaagcactccagaccatcagcccaatcaattaaaaagggcacagacgggaactcagtctcatctggaacggtgacagagtgtttatcgataagtggtgcaacccaagctgtgccagagtcaagatgggagtcatcagagcaaaatgcgtctgtggggagtgtcctcaagtctgcggggagtgcaaagacgatccaggggtcgacacaagaatctggtaccatagcatcactgactcagcctaaagagaatgactccgagtatgagtatgaggatgacctatttacagagattcaggacatccgtgcaagcattgccaagatccatgatgacaacaaggctatcctctcaaagcttgattctatactgttattgaaaggagaagtcgacactatcaagaaacaaatcagcaagcagaatataagtatatccaccattgagggccatctctccagtataatgatagccatcccgggctttgggaaggatatcaaggacccaacatccgaggttgagttgaacccagatttaagacctataataagccgtgactctggcagagctctcgcggaggtcctcaagaaacccgctgtggataggtctcagaaaattggaaccaaagccaactccagctcaaagggtcagcttcttaaggatctccagctaaaacctgtcgacaagcaggcaagctctgcaatcgggtttgtcccatccgaccatgaatcatccagaaatgtcatccgctccataatcaagtcgagcaagctcaacattgatcacaaggactatcttctagatttactgaatgatgtgaaaggctccaaggatcttaaggaattccacaagatgctaacagcaattctcgccaagcacccgtaacacatcctccagtcaccaccccatactcggctgaaaacatcctttcaatcaggctattacaaaaaacttaggagcaagggcaactgagcttcgcagacaagatgaccgagatctacgacttcgataaatcagcatgggatgtcaaagggtcaattgcccgcatagaacccaccacctaccacgacggccgactgataccccaggtaagggtcatcgatcctggtctgggagacagaaaagatgagtgctttatgtacctgtttctcctgggagtgattgaggataacgaccccctgtctccccccgtcggaagaacctttggctctttacctctaggggtcggtaggtcaactgctaagccagaggaactactaagggaggccacagaactagacatagtggtgaggcgcactgcaggagtaaatgagaaactggtattttacaacaacactccgctgtccttgttaacgccctggaagaaagttctgacaaccggaagtgtgtttagcgctaaccaggtttgcaatgcagtcaacctagtcccgcttgatactccccagaggttcagggttgtgtacatgagcataactagattatcagacaatggttactatagtgtgcccagaagaatgctggagttccgctcagccaatgcagtagccttcaacatcttggttacactaaaaattgaaaatggcacaaaccctagaagatacatagtcggctcatgggagaattcagaggtcacatttatggtacacgtgggcaactttagaagaaagaagaacgaagtatactctgctgattactgcaaaatgaagattgaaaagatgggtttagtttttgccctgggaggaataggtggaacaagtctccatattagaagcaccgggaaaatgagcaaaaccctccatgcacagctggggttcaagaaaatcttatgttaccccctaatggatgttaatgaggatcttaaccgatatctctggcgggcagagtgccgaatagtcaaaatccaagccgtcctacagccatcagtgccccaagaattccgtgtctacgatgatgtcatcatcaacgatgatcaaggcttgttcaagatcttgtagttcatttgcaacatcatgacacgggtcgcaatcttgacatttctgtttcttctcccaaatgttgttgcgtgccagattcactggggcaatctatccaagatcgggattgtaggaacagggagtgccagctacaaggtgatgactaggccaagccaccaaactctggttataaagttaatgccaaatataacagccatcaacaattgtacaaagtcagagattgcagagtacaagagattgctgatcacagtgttaaaacctgtagaggatgctctgtcggtgataaccaagaatgtaagaccaattcaaaccctgacacctgggcgtagaacccgtcgttttgctggagctgttctggccggagtagcacttggagttgcgacagccgctcagataactgcaggagtcgctcttcatcaatcattgatgaactctcaagcaattgagagtttaaaaaccagtcttgagaagtcgaatcaggcaatagaagaaatcagacttgcaaataaggagaccatactggcagtacagggcgtccaggattatatcaacaatgagcttgtcccttctgttcatagaatgtcatgcgagctggtaggccacaagctcggcctcaagctccttaggtactacaccgagatcctgtccatattcgggcccagccttcgagacccgatagctgccgaaatatcgatccaggcactcagttatgcattaggcggagacattaataaaatcctggacaagcttgggtatagcggtggggatttccttgccatcctagagagcaagggaataaaggcccgggttacatatgtggacacaagagattactttataatccttagcatcgcctacccaaccttatctgagatcaagggagtgatagttcacaagatagaagctataacatacaacattggggcacaggagtggtatactactatccctaaatatgtagccactcaggggtatctgatatcgaactttgatgagacgtcatgcgtattcactccagaggggacagtttgcagccagaatgcgttgtacccaatgagcccattgcttcaggaatgtttcaggggatcaacaaaatcgtgcgccagaaccctagtttcagggaccataagtaatagatttatcctatcaaaagggaacctgattgcaaattgtgcgtcagttttgtgcaaatgttacacaacggagacagtcatcagccaagatcctgacaaactactaactgttgtagcatccgacaagtgtcctgtagttgaggtggatggagtgacaatacaggtcggcagtcgagagtatccggattctgtatacttacacaaaatagacttaggtccagccatctccctagaaaaactggatgtaggcaccaatttaggcaatgcagtcacaagactggagaatgcaaaggagctcctagatgcatcagaccaaatactgaagactgtcaaaggggcacctttcggcgggaacatgtacatagcactggcagcttgcattggagtatccctagggcttgtcacattaatatgctgctgtaaggggaggtgtaagaacaaggaaatccctatctccaaaatcaacccagggctcaaacccgatctgaccgggacctcaaagtcgtacgtgagatcactgtagccagaatcacctgaatcatctggcatcacacacatacatgtacgacacaagcagtctgaggacgcaggaaacccagcctccgatcacctacctgaccctactccacgctccactacacattagtcatcaaacaaaacttaggacgaaaggtcaatcaccatgtccgcacaaagggaaaggatcaatgccttctacaaagacaatcctcacaataagaaccatagggtgatcctggatagagaacgcttggtcattgaaagaccctacatcttgcttggagtcctgctggtaatgttcctgagtctaatcggactgctggccattgcagggatcaggcttcaccgggccaccgttggaacttcagagatccagagtcggctgaataccaatattaagttggccgaatctattgatcaccagactaaggatgtcttaactcccctttttaaaatcattggcgatgaagtcggcatcagaattccacagaaattcagtgatcttgtcaagttcatctccgataagattaaattcctcaaccctgatagagagtatgatttcagggatctccggtggtgcatgaatccccccgagagagtcaaaattaattttgatcagttttgtgagtacaaagctgcggttaagtcaattgaacatatatttgagtcaccactcaacaagtcaaaaaagctgcaatcattgactctcgggcccggaacaggctgtctaggcaggacagtaacaaaagctcatttctcagaacttacactgaccttaatggacctggatctagagatgaagcacaacgtgtcctcagtgtttaccgtagttgaagaggggttattcggaagaacatataccgtctggagatccgatgccagggatccgagcaccgatctaggtatcggccattttttaagagtcttcgagattggactgataagagatctcgggctgggtccccctgtttttcatatgaccaactatctcacagtgaacatgagtgatgactatcggagatgtcttttagcggtaggggagttgaagttgacagccctatgcacctcatctgagactgtgacactgagtgagagaggagctccaaagagggagcctcttgtggttgtgatacttaatctggctggacccactctagggggcgaactatacagtgtcttgcctacctccgatctcatggtggagaaactctatttatcttcacatagagggatcatcaaggatgacgaggccaattgggtagtgccgtctaccgatgttcgtgatcttcagaacaaaggtgaatgtctggtggaagcatgcaagactcgacctccttcattttgcaatggcacaggatcaggcccgtggtcagaggggagaatccctgcttacggggtgatcagggtcagtcttgacttagctagtgacccggatgtagttatcacttcagtgtttggcccactgattcctcacctatccggcatggatctttacaacaacccgttttcaagagctatatggttggctgtaccaccttatgagcagtcatttctaggaatgataaatacaattggattccctaacagagcagaggttatgccgcacattttgaccacagagatcagaggccctcggggtcgttgccatgttcccatagaattgtcccgcagggttgatgacgatatcaagatcgggtccaacatggtcatattgccgacgatggacctgaggtatattacagccacttatgatgtttccaggagcgagcatgcaatcgtgtactatatctatgacacaggtcgctcatcatcttacttctacccagttcgactgaatttcaaaggcaatcctctctctctgaggatagagtgtttcccttggcgtcataaggtgtggtgctaccatgattgtcttatatacaacaccataacagatgaagaggtccatacgagagggctgaccggtatagaggtaacatgcaatccagtttgagcaaagctacgaccgtcactcaagcagtcccttgagtcgccaccgagtccaagcagcatagcctgggacactcaacagcacagcccagccaacaatgttataaaaaacttaggagccagggttgtaggggccatggactccctatcagtcaatcaggtcttgtaccctgaggttcatctagacagccctattgtcacaaacaaactagttgccatccttgagtactcgggtatcgaccataactatgttcttgaagaccagacccttgtcaagaatattaggtatagactggggtgcggtttttcaaatcaaatgatcatcaataacaggggggtgggtgaaacagtcaattctaaacttaaaagttatccccataatcgtcatatcatatacccggattgcaataaggagttgtttggtatcaaagatagctgcatatctaagaagctctcggagctattcaagaagggtaattccttgtactctaagataagtcaccaggtactggattgtcttaagagagtcaatgggaaattaggcctgggcacagatcttacccatggtctgaaggaaggtatccttgacctagggttgcacatgcatagctctcaatggttcgagactttcctgttctggttcactatcaagacagagatgagatcaatgatcaaagaacagtcccatatatgccacaagaggaggtataacccgatttttgtatcgggggatgcattcgaggtgctcgtatcacgagacctcgttgtgataattgataagaatacccagtatgtcttctacttaacatttgagctggtccttatgtattgtgatgtcatagagggcagacttatgacggagacagccatggccatagaccagagatattcagaacttctaagccgggttagatacttgtgggatcttattgatgggttcttcccaacactaggcaacaccacataccaaattgttgctctgcttgaaccattatcgttggcttatcttcaacttcaggatgtcacgctggagttaagaggtgcttttttagaccattgcttcaaagaactctatgagatactggagcattgcggcattgacacagaaggcacctacaattccatcactgaaggactggattacgtattcatcacccacgatatacatctaactggggagattttttcatttttccggagtttcggacacccccgtctagaagcagtcaccgctgcagaaaatgtcaggaaacatatgaaccaaccgaaggtaatcagttatgagactatgatgaaaggacatgcggtattttgcgggataatcataaatggttttagggaccgacatggcggcagctggccccctgttgcattgccagaacatgcttctgctgcgatccggaatgcgcaggcatcaggtgagggactgacccatgacctgtgtatagacaactggaaatcctttgttggattcaaatttggctgctttatgccactcagcctagatagtgatttgaccatgtatctcaaagacaaggcattggctgcactgaagaatgagtgggattcagtttacccgaaagaatacctccgttataacccacctagagggacagagtcaaggcgactggtggaggtgttcctgaatgactccagctttgatccttataacatgataatgtacgtggtgaatggctcctaccttaaagaccctgagtttaatctctcatacagcctaaaagagaaggagataaaggagacagggcggttgtttgccaaaatgacttataagatgcgggcctgtcaggtaattgctgaaaatctgatatcgaatggtgttgggaagtatttccgagacaatgggatggcaaaggacgagcatgacctaacaaaagcccttcacaccctggcagtctcaggtgttcccaagaacaacaaagataaccaccgaggtgggcctcccagaaggacaacaagccgagaggtgagatcaagccaagacaccaaaacacaaaatagagacaaggtccaaggaggacctatgtacaactatttgcgatgccaaccaatcagccctgatcagggtgagtcatacgagactgttagtgcattcatcaccgctgaccttaagaagtattgcctgaattggagatacgagacaatcagcatatttgcacagaggctgaatgaaatatatggactaccatccttctttcaatggttacacaggatattggaaaaatccgtactctacgtcagtgacccacattgccctcccgatctagataatcatatccctctggacagtgtccctaatgcccaaatattcattaagtacccaatgggcggaatagaaggttattgccaaaaactatggacaatcagtactataccatacttgtatctggcagcctatgagagcggagtaagaatcgcctcactggtgcagggtgacaatcagacaatcgcagtgacaaaaagagttccaagctcttggccttattcactaaaaaagaaggaggcatccagagccgctcaaaattacttcgtagtcttaaggcaaagattgcacgatgtaggtcatcacttaaaggctaatgaaaccatagtatcttctcacttttttgtatattccaaagggatttattatgacggcctgctagtctcacaatcactgaagagcatcgccagatgtgtcttctggtccgagactatagtggatgaaaccagagcggcttgcagcaatattgcaacaactgtcgctaagagtatagagaggggttatgataggtaccttgcatattctttgaatatcctcaagattttccaacagatccttatatcccttaacttcactattaacacaacaatgactcaggatgtcgtggcaccgatcatcgagaacggtgatttgctgataaggatggcactcttgccagcacccatcggaggtctaaattatcttaacatgagcaggttatttgtgagaaatatcggtgacccggtcacctcctctatagccgacctgaagaggatgatagatgccgggctaatgccagaagaaacattgcatcaagtgatgacccagaccccgggagaatcatcctacctcgattgggcaagtgacccttattctgccaacctaccctgcgtacagagtataactcgccttctcaaaaacatcactgcacgatatattttaatcagcagcccaaacccgatgctgaggggattgtttcatgaggggagtagagatgaagacgaggagcttgcaagtttcctaatggatcggcatataattgttccaagagctgcacatgaaatcttagaccatagtataaccggggcaagagaagctatagccgggatgttggacaccaccaaaggtctgattagaacaagtatgaaacggggtggcctcacccctcgagtcttagcccgcctttccaattatgattatgaacaattccgatccgggataacactattgacaaagaaagggcagtgttatctcattgacaaggattcgtgctcggtgcagctcgctatagctctgaggagccacatgtgggctaggttggctcgcgggagaccaatctatgggttggaggtgcccgatatattagaatcgatgaacggctaccttatcaaacgccacgagtcttgtgctatctgtgaaacgggctcaagtcactacggatggtttttcgtccctgcagggtgccagcttgacgatgtatctagagagacttcggctcttcgtgtaccttatgtcggatcaacaactgaggaaaggacagacatgaaacttgcatttgttagatctccaagtagatccctcaaatcagcagttagaattgcaacagtttactcatgggcctacggggatgatgagaagtcatggagtgaagcctggatgctagctaggcagagagccaatatcaccttagatgaattgagaatgataactccagtctctacatccaccaacctagcccaccggttgagggatcggagcacccaggtgaaatactcggggacatcccttgtgagggttgcaagatacacaaccatctccaatgataacttgtcatttgtgatatctgagaaaaaagtggataccaacttcatttatcagcaagggatgttgcttggtcttgggatccttgagaacctctttaggctagaggccaccaccggagtatccaacacagtgctacacctgcacgtggaaacagaatgttgtgttgtacctatggtggatcacccaaggataccgagtctccgtaatataaaagttacggatgagctatgcacaaaccctttgatctacgacaggtctcccatcatagagcacgatgcaacccgactatactcacagagccacaggagacatttggtggagtttgttacctggtccacaagccagctttaccatatactggctaagtctacagcaatgtccatgattgaattgatcacgagattcgagaaagatcacatgaatgaaatagccgccctgattggcgatgacgacatcaacagcttcatcacagaatttttgctagtggagcctagattatttatagtttaccttggtcagtgtgctgccatcaattgggcctttgatatacattatcatcggccctcgggcaagtaccagatgggggaactcctctactctttgctctctcggatgagcaaaggagtatataagatcttcaccaatgctttgagccaccccaaagtttacaagaaattttggcgaagtggtgtcattgagccgattcatggcccatccctggatacacagaatttacatgtcactgtctgtgacatgatatatgggtcctatgtcacctatttggatcttttgctgaatgatgagctagatgattacccgtatttgctctgcgagagtgatgaggacgtggtcacagacaggttcgacaacattcaagccaaacatctctgtgtattagccgatgtatattgcagctccaagaggtgtccttcaataatcgggatgtctcctatagaaaaatgtaccattctcacacattacatcaagggagaatcagtacaatccccgtctgggacctcatggaacactgatccccttgtagtagatcattactcatgctctctgacctaccttcgccgcggttccatcaaacaaatcaggttgagggtggatcctgggtttgtattcgaggcgttgacagacgtcgaccttaaacaacctcgcaaagctaagttagatgtatcggtcgttgggttgactgatttctctcccccttgcgacaacgtcggtgattttctagggactatcaacacattgaggcacaatctgcctgtcaccggaaccggggtctcgaactatgaagtccacgcttatcgtagaattggcctaaattcatcagcatgttacaaagccgtagagatctccacgttaatcaagccatccctagaagtcggagagcatggattgttcttaggagaaggttccggttcaatgctggctgcgtacaaggaagttcttaaattagcaaattgttattacaacagcggggtaacaacggagggcagagccggacagagggaaatatctccctatccttcagagatgagcctagtagagaatcaaatggggatagagcggagtgttaaagtgctgttcaacggcaaacctgaagtaacttgggtggggaccaccgattgctacaagtacataatcagtaacatccaaacctctagtctgggtttcatacattcagatattgagacgctcccaaccaaagatgctgttgagaaattagaagaatttgcctctatcctatccttatccttgattttggggaaaatcggctctattacagttgtcaaaattatgcccattagcggggattttacccaaggctttatagcctatgccatccaatattttagggagagcctgcttgcctatccgagatatagtaacttcatctcgactgagtgttaccttattatgataggattaaaggccaatcgactgataaacccagaagccattaagcaaagcataatcagagcgggggtcaggactgcaccaggacttgtgagccatatattatcagagaaacagaaaggttgtattcaatctttcttgggtgatccttatatccaaggagacttcaataagcaccttaaatctctgacccctattgagaaaatcctggtaaattgtggtctctcgatcaatggcacaaaaatctgtagagatctaatccaccatgatatcgcctccggtccagacggtcttatgagctctacaattattttatatagggaattggctcatttcaaagataatataaggagtcagcacggaatgttccacccctacccagtactggccaacagccggcaacgtgaattaatccttcgaatagccaagaaattctgggggtatgtcttgctatattctgatgacccggcactaatcagacaaacaatcaagaacttgaaacggaatcatctaacctttgacttacatagtaacccgtttattaaagggctatccaaagctgagaaactgctggtgcggacgagtccactaagaagggaatggttgttcgctctcgatacgaaagaagtgaaagagtggttcaaattggtaggttacagtgcactcatcagaggctagttagctatacatctgcccccttctcctccgccatgagaccccactgacgatccagaagattaaagaaaactacatattggataagtatctattcccagctttgtctggt

>JX217850/China/Tibet/2008/bharal

accaaacaaagttgggtaaggatagatcttataataactatggactggcaaacttaggagtaaagatcctactgtcggggggaggaggaggagcaagatctctgattatggcgactctccttaaaagcttagcattgttcaaaaggaacaaagacaaagcgccgacggcatcgggttcaggaggggccatccgagggattaagaatgttatcatagtcccgattcccggagactcgtccatcactacccgttcaagactgctcgacaggcttgtcaggttggccggagatcctgacatcaacgggtcaaagctgaccggcgtgatgatcagcatgctatccttgttcgtagagtcacccgggcaattgatacagcgaatcacagatgatccagatgttagtatccgccttgttgaggtagttcaaagtactaggtcccagtccgggttgacctttgcatcacgtggtgctgatttggataacgaggcagacatgtatttttcaactgaggggccctcgagtggaggtaagaaaaggatcaactggtttgagaacagagaaataatagacatagaggtgcaggatccagaagagttcaatatgttgttagcctccatactagcacaagtctggatccttctggccaaggctgttacggcaccggatacggcagctgactcagaactgagaaggtgggttaaatacacacaacaaaggagagtgattggggaatttcgccttgacaaagggtggctggacgcagtccgcaacagaattgcagaagatctatcactccggcggttcatggtatctctcatacttgacatcaagaggacccccggcaacaagccaaggattgcagaaatgatctgcgacattgacaactatattgtcgaagcaggactcgccagtttcatccttactatcaaatttggtatagaaaccatgtatcctgcattagggctccacgagtttgccggggaattgtccactattgagtccttgatgaacttgtatcaacagctaggagaggttgcaccctacatggtaattctagagaactcaattcagaacaagtttagtgcaggagcttatcccctcctctggagctatgcgatgggtgtcggagttgagctggagaactcaatggggggcttgaactttggcaggtcatattttgaccctgcctattttcgtctcggacaggagatggtcagaagatctgcaggaaaggtcagctctgtgattgcggctgagctcggcatcacagcagaggaagctaaactagtctcggaaatagcctcgcaggctggggacgaaagaaccgctagagggactgggcctcgacaggcgcaggtctccttcctccagcaccaaacaggagggggagagtcgtccgcaccagcgaccagagaaggggtcaaagctgtgatcccaaacggatccgaagaaagggacagaaagcaaacacgcccaggaaggcccagaggagagacccccggccaactgctcctggaaatcatgccagaggatgaggtttcgcgagaatctggtcaaaaccctcgtgaggctcaaagatcggccgaggcactcttcaggctgcaggccatggccaagattctggaggaccaggaggagggagaagacaacaatcaggtctacaacgacaaggatctcctcggctaagcagacgcaccctctgtcgaaatcagtgacgagacatctcctaccagtattataaaaaacttaggacccaggtccaagcaagcacacatcgacactccaaccagtcgagcggaggccaccgatggcagaagaacaagcataccatgtcaacaaggggctggaatgtatcaagtccctcaaagcctctcccccggatctatccaccatcagagataccatcgagagctggagagaggggcttagcccatcgggccgtgcaacaccgaaccctggtatgtccgagggagatcatcagaatatcaaccaaccatgctcaccagcaatcggaccaaacaaagtctacttgtctcctgaagataatctcggatttagagagatcactagcaacgactatgaggctgagctcggaggagtccagggaagaggatccaactctcaagtacagcgttactatgtttatagccacgggggtgaagagattgaaggactcgaggatgctgactctctcgtggttcaagcagatcctccagttgctaacatattcaatggaggagaggatggatctgacgacagcgatgtggactctggcccagatgatcccggcagagatactctatatgaccggggatctgttgccagcaatgatgtcgctaggtccacagatgtcgaaaaactagaaggtgctgatattcaagaagttctcaactcccagaaaggcagaggaggaagattccaaggcgggaaaaccttacgagtcccggaaatacccgatgtcaagcactccagaccatcagcccaatcaattaaaaagggcacagacgggaactcagtctcatctggaacggtgacagagtgtttatcgataagtggtgcaacccaagctgtgccagagtcaagatgggagtcatcagagcaaaatgcgtctgtggggagtgtcctcaagtctgcggggagtgcaaagacgatccaggggtcgacacaagaatctggtaccatagcatcactgactcagcctaaagagaatgactccgagtatgagtatgaggatgacctatttacagagattcaggacatccgtgcaagcattgccaagatccatgatgacaacaaggctatcctctcaaagcttgattctatactgttattgaaaggagaagtcgacactatcaagaaacaaatcagcaagcagaatataagtatatccaccattgagggccatctctccagtataatgatagccatcccgggctttgggaaggatatcaaggacccaacatccgaggttgagttgaacccagatttaagacctataataagccgtgactctggcagagctctcgcggaggtcctcaagaaacccgctgtggataggtctcagaaaattggaaccaaagccaactccagctcaaagggtcagcttcttaaggatctccagctaaaacctgtcgacaagcaggcaagctctgcaatcgggtttgtcccatccgaccatgaatcatccagaaatgtcatccgctccataatcaagtcgagcaagctcaacattgatcacaaggactatcttctagatttactgaatgatgtgaaaggctccaaggatcttaaggaattccacaagatgctaacagcaattctcgccaagcacccgtaacacatcctccagtcaccaccccatactcggctgaaaacatcctttcaatcaggctattacaaaaaacttaggagcaagggcaactgagcttcgcagacaagatgaccgagatctacgacttcgataaatcagcatgggatgtcaaagggtcaattgcccgcatagaacccaccacctaccacgacggccgactgataccccaggtaagggtcatcgatcctggtctgggagacagaaaagatgagtgctttatgtacctgtttctcctgggagtgattgaggataacgaccccctgtctccccccgtcggaagaacctttggctctttacctctaggggtcggtaggtcaactgctaagccagaggaactactaagggaggccacagaactagacatagtggtgaggcgcactgcaggagtaaatgagaaactggtattttacaacaacactccgctgtccttgttaacgccctggaagaaagttctgacaaccggaagtgtgtttagcgctaaccaggtttgcaatgcagtcaacctagtcccgcttgatactccccagaggttcagggttgtgtacatgagcataactagattatcagacaatggttactatagtgtgcccagaagaatgctggaattccgctcagccaatgcagtagccttcaacatcttggttacactaaaaattgaaaatggcacaaaccctagaagatacatagtcggctcatgggagaattcagaggtcacatttatggtacacgtgggcaactttagaagaaagaagaacgaagtatactctgctgattactgcaaaatgaagattgaaaagatgggtttagtttttgccctgggaggaataggtggaacaagtctccatattagaagcaccgggaaaatgagcaaaaccctccatgcacagctggggttcaagaaaatcttatgttaccccctaatggatgttaatgaggatcttaaccgatatctctggcgggcagagtgccgaatagtcaaaatccaagccgtcctacagccatcagtgccccaagaattccgtgtctacgatgatgtcatcatcaacgatgatcaaggcttgttcaagatcttgtagttcatttgcaacatcatgacacgggtcgcaatcttgacatttctgtttcttctcccaaatgttgttgcgtgccagattcactggggcaatctatccaagatcgggattgtaggaacagggagtgccagctacaaggtgatgactaggccaagccaccaaactctggttataaagttaatgccaaatataacagccatcaacaattgtacaaagtcagagattgcagagtacaagagattgctgatcacagtgttaaaacctgtagaggatgctctgtcggtgataaccaagaatgtaagaccaattcaaaccctgacacctgggcgtagaacccgtcgttttgctggagctgttctggccggagtagcacttggagttgcgacagccgctcagataactgcaggagtcgctcttcatcaatcattgatgaactctcaagcaattgagagtttaaaaaccagtcttgagaagtcgaatcaggcaatagaagaaatcagacttgcaaataaggagaccatactggcagtacagggcgtccaggattatatcaacaatgagcttgtcccttctgttcatagaatgtcatgcgagctggtaggccacaagctcggcctcaagctccttaggtactacaccgagatcctgtccatattcgggcccagccttcgagacccgatagctgccgaaatatcgatccaggcactcagttatgcattaggcggggacattaataaaatcctggacaagcttgggtatagcggtggggatttccttgccatcctagagagcaagggaataaaggcccgggttacatatgtggacacaagagattactttataatccttagcatcgcctacccaaccttatctgagatcaagggagtgatagttcacaagatagaagctataacatacaacattggggcacaggagtggtatactactatccctaaatatgtagccactcaggggtatctgatatcgaactttgatgagacgtcatgcgtattcactccagaggggacagtttgcagccagaatgcgttgtacccaatgagcccattgcttcaggaatgtttcaggggatcaacaaaatcgtgcgccagaaccctagtttcagggaccataagtaatagatttatcctatcaaaagggaacctgattgcaaattgtgcgtcagttttgtgcaaatgttacacaacggagacagtcatcagccaagatcctgacaaactactaactgttgtagcatccgacaagtgtcctgtagttgaggtggatggagtgacaatacaggtcggcagtcgagagtatccggattctgtatacttacacaaaatagacttaggtccagccatctccctagaaaaactggatgtaggcaccaatttaggcaatgcagtcacaagactggagaatgcaaaggagctcctagatgcatcagaccaaatactgaagactgtcaaaggggcacctttcggcgggaacatgtacatagcactggcagcttgcattggagtatccctagggcttgtcacattaatatgctgctgtaaggggaggtgtaagaacaaggaaatccctatctccaaaatcaacccagggctcaaacccgatctgaccgggacctcaaagtcgtacgtgagatcactgtagccagaatcacctgaatcatctggcatcacacacatacatgtacgacacaagcagtctgaggacgcaggaaacccagcctccgatcacctacctgaccctactccacgctccactacacattagtcatcaaacaaaacttaggacgaaaggtcaatcaccatgtccgcacaaagggaaaggatcaatgccttctacaaagacaatcctcacaataagaaccatagggtgatcctggatagagaacgcttggtcattgaaagaccctacatcttgcttggggtcctgctggtaatgttcctgagtctaatcggactgctggccattgcagggatcaggcttcaccgggccaccgttggaacttcagagatccagagtcggctgaataccaatattaagttgaccgaatctattgatcaccagactaaggatgtcttaactcccctttttaaaatcattggcgatgaagtcggcatcagaattccacagaaattcagtgatcttgttaagttcatctccgataagattaaattcctcaaccctgatagagagtatgatttcagggatctccggtggtgcatgaatccccccgagagagtcaaaattaattttgatcagttttgtgagtacaaagctgcggttaagtcaattgaacatatatttgagtcaccactcaacaagtcaaaagagctgcaatcattgactctcgggcccggaacaggctgtctaggcaggacagtaacaaaagctcatttctcagaacttacactgaccttaatggacctggatctagagatgaagcacaacgtgtcctcagtgtttaccgtagttgaagaggggttattcggaagaacatataccgtctggagatctgatgccagggatccgagcaccgatctaggtatcggccattttttaagagtcttcgagattggactgataagagatctcgggctgggtccccctgtttttcatatgaccaactatctcacagtgaacatgagtgatgactatcggagatgtcttttagcggtaggggagttgaagttgacagccctatgcacctcatctgagactgtgacactgagtgagagaggagctccaaagagggagcctcttgtggttgtgatacttaatctggctggacccactctagggggcgaactatacagtgtcttgcctacctccgatctcatggtggagaaactctatttatcttcacatagagggatcatcaaggatgacgaggccaattgggtagtgccgtctaccgatgttcgtgatcttcagaacaaaggtgaatgtctggtggaagcatgcaagactcgacctccttcattttgcaatggcacaggatcaggcccgtggtcagaggggagaatccctgcttacggggtgatcagggtcagtcttgacttagctagtgacccggatgtagttatcacttcagtgtttggcccactgattcctcacctatccggcatggatctttacaacaacccgttttcaagagctatatggttggctgtaccaccttatgagcagtcatttctaggaatgataaatacaattggattccctaacagagcagaggttatgccgcacattttgaccacagagatcagaggccctcggggtcgttgccatgttcccatagaattgtcccgcagggttgatgacgatatcaagatcgggtccaacatggtcatattgccgacgatggacctgaggtatattacagccacttatgatgtttccaggagcgagcatgcaatcgtgtactatatctatgacacaggtcgctcatcatcttacttctacccagttcgactgaatttcaaaggcaatcctctctctctgaggatagagtgtttcccttggcgtcataaggtgtggtgctaccatgattgtcttatatacaacaccataacagatgaagaggtccatacgagagggctgaccggtatagaggtaacatgcaatccagtttgagcaaagctacgaccgtcactcaagcagtcccttgagtcgccaccgagtccaagcagcatagcctgggacactcaacagcacagcccagccaacaatgttataaaaaacttaggagccagggttgtaagggccatggactccctatcagtcaatcaggtcttgtaccctgaggttcatctagacagccctattgtcacaaacaaactagttgccatccttgagtactcgggtatcgaccataactatgttcttgaagaccagacccttgtcaagaatattaggtatagactggggtgcggtttttcaaatcaaatgatcatcaataacaggggggtgggtgaaacagtcaattctaaacttaaaagttatccccataatcgtcatatcatatacccggattgcaataaggagttgtttggtatcaaagatagctgcatatctaagaagctctcggagctattcaagaagggtaattccttgtactctaagataagtcaccaggtactggattgtcttaagagagtcaataggaaattaggcctgggcacagatcttacccatggtctgaaggaaggtatccttgacctagggttgcacatgcatagctctcaatggttcgagactttcctgttctggttcactatcaagacagagatgagatcaatgatcaaagaacagtcccatatatgccacaagaggaggtataacccgatttttgtatcgggggatgcattcgaggtgctcgtatcacgagacctcgttgtgataattgataagaatacccagtatgtcttctacttaacatttgagctggtccttatgtattgtgatgtcatagagggcagacttatgacggagacagccatggccatagaccagagatattcagaacttctaagccgggttagatacttgtgggatcttattgatgggttcttcccaacactaggcaacaccacataccaaattgttgctctgcttgaaccattatcgttggcttatcttcaacttcaggatgtcacgctggagttaagaggtgcttttttagaccattgcttcaaagaactctatgagatactggagcattgcggcattgacacagaaggcacctacaattccatcactgaaggactggattacgtattcatcacccacgatatacatctaactggggagattttttcatttttccggagtttcggacacccccgtctagaagcagtcaccgctgcagaaaatgtcaggaaacatatgaaccaaccgaaggtaatcagttatgagactatgatgaaaggacatgcggtattttgcgggataatcataaatggttttagggaccgacatggcggcagctggccccctgttgcattgccagaacatgcttctgctgcgatccggaatgcgcaggcatcaggtgagggactgacccatgacctgtgtatagacaactggaaatcctttgttggattcaaatttggctgctttatgccactcagcctagatagtgatttgaccatgtatctcaaagacaaggcattggctgcactgaagaatgagtgggattcagtttacccgaaagaatacctccgttataacccacctagagggacagagtcaaggcgactggtggaggtgttcctgaatgactccagctttgatccttataacatgataatgtacgtggtgaatggctcctaccttaaagaccctgagtttaatctctcatacagcctaaaagagaaggagataaaggagacagggcggttgtttgccaaaatgacttataagatgcgggcctgtcaggtaattgctgaaaatctgatatcgaatggtgttgggaagtatttccgagacaatgggatggcaaaggacgagcatgacctaacaaaagcccttcacaccctggcagtctcaggtgttcccaagaacaacaaagataaccaccgaggtgggcctcccagaaggacaacaagccgagaggtgagatcaagccaagacaccaaaacacaaaatagagacaaggtccaaggaggacctatgtacaactatttgcgatgccaaccaatcagccctgatcagggtgagtcatacgagactgttagtgcattcatcaccgctgaccttaagaagtattgcctgaattggagatacgagacaatcagcatatttgcacagaggctgaatgaaatatatggactaccatccttctttcaatggttacacaggatattggaaaaatccgtactctacgtcagtgacccacattgccctcccgatctagataatcatatccctctggacagtgtccctaatgctcaaatattcattaagtacccaatgggcggaatagaaggttattgccaaaaactatggacaatcagtactataccatacttgtatctggcagcctatgagagcggagtaagaatcgcctcactggtgcagggtgacaatcagacaatcgcagtgacaaaaagagttccaagctcttggccttattcactaaaaaagaaggaggcatccagagccgctcaaaactacttcgtggtcttaaggcaaagattgcacgatgtaggtcatcacttaaaggctaatgaaaccatagtatcttctcacttttttgtatattccaaagggatttattatgacggcctgctagtctcacaatcactgaagagcatcgccagatgtgtcttctggtccgagactatagtggatgaaaccagagcggcttgcagtaatattgcaacaactgtcgctaagagtatagagaggggttatgataggtaccttgcatattctttgaatatcctcaagattttccaacagatccttatatcccttaacttcactattaacacaacaatgactcaggatgttgtggcaccgatcatcgagaacggtgatttgctgataaggatggcactcttgccagcacccatcggaggtctaaattatcttaacatgagcaggttatttgtgagaaatatcggtgacccggtcacctcctctatagccgacctgaagaggatgatagatgccgggctaatgccagaagaaacattgcatcaagtgatgacccagaccccgggagaatcatcctacctcgattgggcaagtgacccttattctgccaacctaccctgcgtacagagtataactcgccttctcaaaaacatcactgcacgatatattttgatcagcagcccaaacccgatgctgagaggattgtttcatgaggggagtagagatgaagacgaggagcttgcaagtttcctaatggatcggcatataattgttccaagagctgcacatgaaatcttagaccatagtataaccggggcaagagaagctatagccgggatgttggacaccaccaaaggtctgattagaacaagtatgaaacggggtggcctcacccctcgagtcttagcccgcctttccaattatgattatgaacaattccgatccgggataacactattgacaaagaaagggcagtgttatctcattgacaaggattcgtgctcggtgcagctcgctatagctctgaggagccacatgtgggctaggttggctcgcgggagaccaatctatgggttggaggtgcccgatatattagaatcgatgaacggctaccttatcaaacgccacgagtcttgtgctatctgtgaaacgggctcaagtcactacggatggtttttcgtccctgcagggtgccagcttgacgatatatctagagagacttcggctcttcgtgtaccttatgtcggatcaacaactgaggaaaggacagacatgaaacttgcatttgttagatctccaagtagatccctcaaatcagcagttagaattgcaacagtttactcatgggcctacggggatgatgagaagtcatggagtgaagcctggatgctagctaggcagagagccaatatcaccttagatgaattgagaatgataactccagtctctacatccaccaacctagcccaccggttgagggatcggagcacccaggtgaaatactcggggacatcccttgtgagggttgcaagatacacaaccatctccaatgataacttgtcatttgtgatatctgagaaaaaagtggataccaacttcatttatcagcaagggatgttgcttggtcttgggatccttgagaacctctttaggctagaggccaccaccggagtatccaacacagtgctacacctgcacgtggaaacagaatgttgtgttgtacctatggtggatcacccaaggataccgagtctccgtaatataaaagttacggatgagctatgcacaaaccctttgatctacgacaggtctcccatcatagagcacgatgcaacccgactatactcacagagccacaggagacatttggtggagtttgttacctggtccacaagccagctttaccatatactggctaagtctacagcaatgtccatgattgaattgatcacgagattcgagaaagatcacatgaatgaaatagccgccctgattggcgatgacgacatcaacagcttcatcacagaatttttgctagtggagcctagattacttatagtttaccttggtcagtgtgctgccatcaattgggcctttgatatacattatcatcggccctcgggcaagtaccagatgggggaactcctctactctttgctctctcggatgagcaaaggagtatataagatcttcaccaatgctttgagccaccccaaagtttacaagaaattttggcgaagtggtgtcattgagccgattcatggcccatccctggatacacagaatttacatgtcactgtctgtgacatgatatatgggtcctatgtcacctatttggatcttttgctgaatgatgagctagatgattacccgtatttgctctgcgagagtgatgaggacgtggtcacagacaggttcgacaacattcaagccaaacatctctgtgtattagccgatgtatattgcagctccaagaggtgtccttcaataatcgggatgtctcctatagaaaaatgtaccattctcacacattacatcaagggagaatcagtacaatccccgtctgggacctcatggaacactgatccccttgtagtagatcattactcatgctctctgacctaccttcgccgcggttccatcaaacaaatcaggttgagggtggatcctgggtttgtattcgaggcgttgacagacgtcgaccttaaacaacctcgcaaagctaagttagatatatcggtcgttgggttgactgatttctctcccccttgcgacaacgtcggtgattttctagggactatcaacacattgaggcacaatctgcctgtcaccggaaccggggtctcgaactatgaagtccacgcttatcgtagaattggcctaaattcatcagcatgttacaaagccgtagagatctccacgttaatcaagccatccctagaagtcggagagcatggattgttcttaggagaaggttccggttcaatgctggctgcgtacaaggaagttcttaaattagcaaattgttattacaacagcggggtaacaacggagggcagagccggacagagggaaatatctccctatccttcagagatgagcctagtagagaatcaaatggggatagagcggagtgttaaagtgctgttcaacggcaaacctgaagtaacttgggtggggaccaccgattgctacaagtacataatcagtaacatccaaacctctagtctgggtttcatacattcagatattgagacgctcccaaccaaagatgctgttgagaaattagaagaatttgcctctatcctatccttatccttgattttggggaaaatcggctctattacagttgtcaaaattatgcccattagcggggattttacccaaggctttatagcctatgccatccaatattttagggagagcctgcttgcctatccgagatatagtaacttcatctcgactgagtgttaccttattatgataggattaaaggccaatcgactgataaacccagaagccattaagcaaagcataatcagagcgggggtcaggactgcaccaggacttgtgagccatatattatcagagaaacagaaaggttgtattcaatctttcttgggtgatccttatatccaaggagacttcaataagcaccttaaatctctgacccctattgagaaaatcctggtaaattgtggtctctcgatcaatggcacaaaaatctgtagagatctaatccaccatgatatcgcctccggtccagacggtcttatgagctctacaattattttatatagggaattggctcatttcaaagataatataaggagtcagcacggaatgttccacccctacccagtactggccaacagccggcaacgtgaattaatccttcgaatagccaagaaattctgggggtatgtcttgctatattctgatgacccggcactaatcagacaaacaatcaagaacttgaaacggaatcatctaacctttgacttacatagtaacccgtttattaaagggctatccaaagctgagaaactgctggtgcggacgagtccactaagaagggaatggttgttcactctcgatacgaaagaagtgaaagagtggttcaaattggtaggttacagtgcactcatcagaggctagttagctatacatctgcccccttctcctccgccatgagaccccactgacgatccagaagattaaagaaaactacatattggataagtatctattcccagctttgtctggt

>KJ466104/Ghana/Accra/2010

accaaacaaagttgggtaaggatagatcttacaatgactatagactagcaaacttaggagtaaagatcctactgtcggggagaggaggaggagcaagatctttgaccatggcgactctccttaaaagcttagcactgttcaagaggaacaaagacaaagcgcctactgcgtcaggttcaggaggggccatccgggggattaagaatgttatcatagtccctatccccggggactcatccatcattacccgttcaagactgctcgacaggcttgtcagactggccggagatcctgacatcaacgggtcaaagctgaccggcgtgatgatcagcatgttatctttgttcgtggagtcacccgggcaattgatacagcggatcacagatgatccagatgttagcatccgtcttgttgaggtagttcaaagtaccaggtcccagtcaggattaacctttgcatcacgtggtgctgatttggacaatgaggcagatatgtatttttcaactgaaggaccctcgagtggaagtaagaaaaggatcaactggtttgagaacagagaaataatagatatagaagtgcaagatgcagaagagttcaatatgttgttagcctccatcttagcacaagtttggatcctcctggccaaggcggtcacggcaccagatacagcagccgactcagaactgagaaggtgggttaaatacacacaacaaaggagagtgattggggaatttcgctttgacaaagggtggctggacgcaatccgcaacaggattgcagaagatctatcactccggcggttcatggtatccctgatacttgacatcaagaggacccccggcaacaagccaaggattgcagaaatgatctgcgacattgacaactatattgtcgaagccggacttgcaagtttcattctcactatcaagtttggtattgaaaccatgtatcctgcattaggccttcacgagttcgccggggaattgtccactattgaatccttgatgaacttgtatcaacagctaggagaagttgcaccctatatggtaattctagagaactcaattcagaacaagtttagtgcaggagcctatcctctcctctggagctatgcgatgggtgtcggagtcgagttggagaactcaatggggggcttgaactttggcaggtcatattttgacccggcctattttcgtctcggacaggagatggtcagaagatctgcaggaaaggtcagctctgtaattgcggctgagcttggcatcacagcagaggaagccaaactagtctcggaaatcgcctcgcagactggggaagaacgaaccgttagagggactgggcctcgacaggcgcaggtttccttcctccagcacaaaattggtgagggagagtcgcctacaccagcgaccaaagaagaagttaaagctgcgatcccaaacggatctgaaggaagggacataaagcgaacacgcccaaggaagcccagaggagaaactcccgggcaactgcttctggagatcatgccagaggatgaagcctcgcgagagtctggtcaaaaccctcgtgaggctcaaagatcggctgaggcactcttcaggctgcaggccatggccaagattctggaggaccaggaggagggagaagacaacagtcagatctacaacgacaaggatctcctcagctgagcagatacaccctctgtccaaatcagtgacaagacatcacctaccagtattataaaaaacttaggacccaggtccaaccaaccgcacatcgacaccctagtcaatcgagcggagaccaccgatggcagaagaacaagcataccatgtcaacaaggggctggaatgtatcaagtccctcaaagcctctcccccggatctatccaccatccaagatgcccttgagagctggagagaggggttcaacccctcaggccgtgcaacaccgaaccatgatacgtccgagggggaccatcagaatatcaaccaatcatgctcttcagcaatcggatcagacaaagtcgacatgtctcctgaaggtaatctcggatttagagagatcacttgtgatgacaatgagactgggctcggaggagttcaggacaaaggatccgactctcaagtacagcgttactatgtttataaccacgggggtgaagagattgaaggactcgaggatgctgactctctcgtggttcaagcaaatcctccggttgctaacaccttcgatggaggagaggatggatctgacaacagcgatgtggactctggcccagatgataccggcagagatcctctatatgaccggggacctgctgccggcaatgatgtctctaggtcaacagatgtcgaaaaattagaaggtgatgacattcaagaagttcttaactcccagaagagtaaaggaggaagattccagggcgggaaaatcctgcgagtcccggaaatacccgatgtcaagaactccagaccatcggcccaatcaattaaaaagggcacagacgggagctcagtcttatctggaatggtgacagagtgttcatcgataagtggtgcaacccaagctgtgctagagtccagatgggagtcatcagagcgaaatgcatctgtggggagtgtccccaaatctgcgaggagtgcaaagacgatccaggggttaacacaagaatctggtaccatagcatcactgactcagcctaaagaaaatgactccgagtatgagtatgaggatgatctattcactgagatgcaggacattcgtgcaagcattgctaagatccatgatgacaacaaaactatcctctcaaaacttgattctctactgttattgaaaggagaaatcgatactatcaagaaacaaatcagcaaacaaaatataagtatatctaccattgagggccatctatccagtataatgatagccatcccaggttttgggaaggacatcaaggatccaacatctgaggttgagttgaacccagatttgagacctataatcagccgcgattctggcagggctcttgcggaggtccttaagaaacccgctgttgataggtctcagaagagcggaatcaaagtcaactccggttcaaagggtcagctcctcaaggatctccagctaaaacccgtcgacaagcaggcaagctctgcaatcgggtttgttccatccgaccatgaatcatccagaagtgtcatccgctccataatcaagtcgagcaagcttaacattgatcacaaagactatcttctagatttactgaatgatgtgaaaggctctaaggatctcaaggaattccacaagatgctaacagccattcttgccaagcagccgtaacacatcccataatctacatctcatactcggttgaaagcatcctctcaatcaggatattacaaaaaacttaggagcaagggcaactgagcttcacagacaggatgaccgagatttacgatttcgataaatcagcatgggatgtcaaagggtcaattgctcgcatagaacccaccacctatcacgacggccgactggtaccccaggtgagggtcatcgaccctggtctgggagacagaaaagatgagtgcttcatgtacctgtttcttctaggagtgattgaggataacgaccccctttctcccccagtcgggagaaccttcgggtctttacctctaggtgtcggtaagtcaactgctaagccagaggaactactaagggaggccacagaactagatatagtggtgaggcgcacggcaggattaaatgagaaactggtattttacaacaacactccgctatctttgttaacaccctggaggaaagtcttgacgaccggaagtgtattcagcgccaaccaggtttgcaatgcggtcaacctagtcccactcgatactccccagagattcagggttgtgtacatgagtataactagattgtcagataatggttattatagtgtccccagaagaatgttggagttccgctcagccaatgcagtcgcttttaatatcttggttacactgagaattgagaatggcacaaaccctagaagatacatagtcggctcatgggagaatccagaagtcacatttatggtacacgtgggcaactttagaagaaagaagaacgaagtatactctgctgattattgcaaaatgaagattgaaaagatgggtctagtttttgccttgggaggaataggtggaacaagtctccatattcgaagcacagggaaaatgagcaagaccctccatgcacagctggggttcaagaaaattctatgctaccctctgatggatatcaatgaggatcttaaccgatatctctggcgggcagagtgccgaatagtcaaaatccaggccgtcttacagccatcagtaccccaagaattccgtgtctatgatgatgtcatcatcaacgatgaccaaggcctgttcaagatcctgtaagtcacctgcaacatcatgacacgggtcgcaaccttggtattgctgcttctcttcccaaacaccgtcgcgtgccagattcactggggcaatctatccaagatagggattgtcggaacagggagtgccagttacaaggtgatgaccaggccaagccaccaaactctggttataaagttgatgccaaatataacagccatcgacaattgtacgaaatcagagatttcagagtacaaaagattgctaatcacagtgttaaagcctgtagaggatgctctgtcagtaataaccaagaatgtaagaccaattcaagctctaacacctgggcgcaggacccgccgtttcgccggagctgttctggccggagtagcacttggagtcgcgacggctgctcaaataactgccggagtcgcactccatcagtcattgatgaattcccaagcaattgaaagtttaaaaaccagtcttgagaagtcaaatcaggcaatagaagaaatcagacttgcaaataaggagaccatactggcagtacagggcgtccaagattatatcaacaacgagcttgtcccctctgttcatagaatgtcatgtgagctaataggtcacaaactcagtctcaaactccttaggtattataccgagatcctgtctatattcgggcctagccttcgagacccgatagcagctgaaatatcaatccaggcactcagctatgcactaggcggagacatcaataaaattctggacaagcttgggtatagcggcggggatttccttgctattctagaaagcaaggggataaaggctcgggtcacatatgtggacacaagagattactttataattcttagcatagcctacccgaccttatctgagatcaaaggggtgatagttcataagatagaagctatatcatacaacattggggcacaggaatggtatactactatccctaaatatgtagccactcagggatatttgatatcgaatttcgatgagacgtcatgcgtcttcactccagaggggacagtctgcagccagaatgcgttgtatccaatgagcccattgcttcaggaatgtttcagggggtcgaccaaatcgtgtgccagaaccctagtttcagggaccacaagtaatagatttatcctgtcaaaagggaacttgattgcaaattgtgcgtcagttttgtgcaagtgttacacaacggagacagtcatcaaccaagatcctgataaactactaactgttatagcctcagataagtgtcccgtagttgaggtggatggagtgacaatacaagtcggcagtcgagagtacccagattctgtatacctacataaaatagacttaggcccagccatctctctggaaaaactggatgtgggcaccaatttaggcaatgcagtcacgagactggagaatgcaaaggagctcctagacgcatcagaccagatactgaagactgtgaaaggggtacccttcagtggcaatatgtacatagcactggcagcttgcattggggtatccctagggcttgtcacattaatatgctgctgtaaggggagatgtaggaacaaggagattcctgcctccaaaatcaacccagggctcaaacccgacctgaccgggacttcaaagtcttacgtgagatcactgtagtcagaacaacccgaatcatccggcatcacgcgtatacatgtgcgacacaagcagtcagaggacgcagaagagtcaacctccgatcaccgaccagaccccactctacgccctactacacattggtcatcaaacaaaacttaggacgaaaggtcaatcaccatgtccgcacaaagggagaggatcaatgccttctacaaagacaaccctcacaataaaaaccataggataatcctggatagggaacgcttaactattgaaagaccctacatcttacttggagtcctgctggtaatgttcctgagtctaatcgggctgctagccattgcagggatcaggcttcaccgggccaccgttggaactgcggagatccagagtcggctgaataccaacattgagttgaccgaatccattgatcatcaaactaaggatgtcttaacacccctgtttaaaatcattggtgatgaagtcggtatcagaattccacagaagttcagtgatcttgtcaagttcatctccgataagattaagttcctcaaccctgacagagaatacgattttagggatctccggtggtgtatgaacccccctgagagagtcaaaattaactttgatcaattctgtgaatacaaagccgcggtcaagtcagttgaacatatatttgagtcatcattcaacaggtcagaaagattgcgactattgactcttgggcccggaacaggctgtctcggcaggacagtaacaagagctcagttctcagaacttacgctgaccctgatggacctggatctcgagatgaagcacaacgtgtcctcagtgtttaccgtagttgaagagggattattcggaagaacatatattgtctggagatctgacaccgggaaaccgagcaccagtctagatattggccagtttttaagagtctttgagatcgggttggtgagggatctcgagctgggtgcccccattttccatatgaccaactacctcacggtgaacatgagtgatgactatcggagttgccttttagcggtaggggagttgaagctaacagccctatgcaccccatctgagactgtgactctgagtgagagaggagttccaaagagagagcctcttgtggttgtgatactcaacctagttgggcctactctagggggcgaactatacagtgtcttgcctacctctgacctcatggtagagaaactccatctatcctcacacagagggatcatcaaagacaacgaggccaattgggtagtaccgtctaccgatgttcgtgatctccaaaacaagggagaatgtctggtggaagcatgcaaaactcgacctccttcattttgcaatggcacaggaataggcccatggtcagaggggagaatccctgcctacggagtgatcagggtcagtcttgacttagctagtgacccaggtgtggttatcacttcagtgtttggcccactgatacctcacctatctggcatggatctttacaacaatccgttttcaagagctgcatggctggctgtaccaccctacgagcagtcatttctaggaatgataaatacaattggcttcccggacagagcagaggtcatgccgcacattttgaccacagagatcaaagggcctcgaggtcgttgtcatgttcctatagagttgtcccgcaggattgatgatgatatcaagatcgggtccaatatggttgtattgccgacgagggatctgaggtacataacagccgcttatgatgtttccaggagcgagcatgcaatcgtgtactatatctatgacacgggtcgctcatcatcttacttctacccagttcgattgaatttcaagggcaatcctctctctctgaggatagagtgtttcccctggtctcataaggtgtggtgctaccatgattgtcttatatacaacaccatgacaaacgaagaagtccatacgagagggctgaccggtatagaggtaacatgcaatccagtctgagtcgagctgaaaccatcgctcaagcaggcttccgagccatcccctagttcaagcagcatagtctgggacactcagcagcacaacccagccaacaatgttataaaaaacttaggagccaaggttgtaggagccatggactcactatcagtcaatcaggttttgtaccctgaggtccatctagatagccctattgtcacaaacaaactagttgctatccttgaatactcggggatcaaccacaactatgttcttgaagaccagactcttatcaagaatatcagatatagactggggtgcggtttttcaaatcaaatgatcatcaataataggggggtaggtgaaacagtcaattccaaacttaaaagttacccccgtaactgtcatatcatatacccagactgcaataaggagttgttttgtatcaaagatagctgcatatctagaaagctctcggagctattcaagaagggtaattccttgtactctaaggtaagtcaccaggtactggattgtcttaagagagtcaacgggaaattagggctgggcacagatcttaatcacggcctgaaggatggtatcctcgacttggggttgcacatgcatagctctcaatggttcgagacctttctgttctggttcactatcaagacagagatgagatcaatgatcaaagaacagtcccatatatgccacaagaggaggtataacccaatttttgtgtcaggggatgcattcgaggtgcttgtatcacgagacctcgtagtgataattgataagaacacccagtatgtcttctacctgacgtttgagctggtccttatgtattgtgatgtcatagagggtagacttatgacggagacagccatggctatagaccagagatattcagagcttctaaaccgggtcagatacttgtgggaccttatcgatgggttcttcccaacactgggtaacaccacataccaagttgttgctctgcttgaaccgctgtcgttggcttatcttcaacttcaggatgtcactctagagttaagaggtgcctttttggaccactgcttcaaagaactttatgagatactggagcattgtggcattgacacggaaggtacctacaattccatcactgagggattggattacgtatttatcactcacgatatacacttaactggggagattttttcattttttcggagtttcggacacccccgcctcgaagcggtcaccgctgcagagaatgtcagaaaacatatgaaccagccgaaggtaatcagttatgagactatgatgaaagggcatgcagtattttgcgggataatcataaatggttttagggaccggcacggcggcagctggccccctgttgcattgccagaacatgcttctgctgcgatccggaatgcgcaggcatccggcgaaggactgacccacgacctgtgtatagacaactggaagtcttttgtaggattcagatttggctgcttcatgccgctcagcctagatagtgatttgaccatgtacctcaaagacaaagcattggctgcacttaagaatgagtgggattcagtttacccgaaagaatacctccgttataatccacctagagggacagagtcaaggcgactagtagaggtgttcctgaatgactccagctttgatccttataacatgataatgtacgtggtgaatggctcctaccttaaagaccctgagtttaatctctcatacagcttaaaagaaaaggagataaaagagacagggcggttgtttgccaagatgacctacaagatgcgggcctgtcaggtgattgctgaaaatctgatatcaaatggtgttgggaagtatttccgagacaatgggatggcgaaagacgagcatgacctaacaaaagcccttcacactctggcagtctcaggcgttcccaagaacaacaaagacaaccaccgaggtgggccccccagaaggaacgcaagccgagagatgagatcaagccaagccgtcaacacacaaaatagagacaagatccaggggggccctatgtacaactacttgcgatgccaaccaaccagccctgatcagggtgagtcatacgagactgttagtgcattcatcaccgctgaccttaagaagtattgcctaaattggagatacgagacaatcagcatatttgcacagagactgaatgaaatatatgggttgccatccttctttcaatggttacataaggtattggagaaatccgtgctctacgtcagtgatccgcattgccctcccgacttagatgatcacatccctctggacagtgtccctaatgcccaaatattcatcaagtacccgatgggcggaatagaaggttattgtcaaaaactatggacaatcagtactataccatatttgtatctagcagcctatgagagtggagtaagaatcgcctcactagtgcaaggtgacaatcagacaattgcagtgacaaaaagagttccaagttcttggccttattcactaaaaaagagggaggcatctaaagcagctcaaaattactttgtggtcttaaggcagaggttgcacgatgtaggtcatcacttaaaggctaatgagaccatagtatcttctcacttttttgtatactctaaagggatctattatgacggcctgttagtttcacaatcactaaaaagcatcgccagatgtgtcttctggtccgagaccatcgtggatgaaaccagagcggcctgcagcaacattgcaacaactatcgccaagagtatagagaggggttacgataggtacctcgcatactctttgaatatcctaaaaatttttcaacagatccttgtatcccttgacttcacgattaatacaacaatgactcaagatgtcgtggcaccgatcatcgagaacggcgatttactgataaggatggcactcttgccagcacccattgggggtctcaattatctcaacatgagcaggttgtttgtgagaaatatcggtgacccggtcacttcctccatagccgacctgaagaggatgatagacgctgggctaatgccagaagaaacattgcatcaagtgatgacccagaccccgggagaatcatcctaccttgattgggcaagtgatccttattctgccaacctaccctgcgtacagagtataacccgccttctcaagaacatcactgcacggtatattttaatcagcagcccaaatccgatgctgaaagggttgtttcacgaggggagtagagatgaagacgaggagcttgcgagtttcttaatggatcggcatataattgttccgagagctgcacatgaaatcttagaccacagcataaccggggcaagagaagctatagccgggatgttggacaccaccaagggtctgattagaacaagtatgaaacggggtggcctcacccctcgagtattagcccgcctttccaattatgattatgaacaattcagatccgggatcacactattgacaaagaaagggcagtgttatctcattgacaaggactcgtgctcggtgcagctcgctatagctctgaggagccatatgtgggcaaggttggctcgcgggagacctatctatggcttggaggtgcctgatgtactggaatcgatgaacggctaccttatcaaacgtcacgagtcttgtgccatctgtgaaacgggctcaagtcactacgggtggtttttcgtccctgcagggtgtcagcttgacgatgtctcaagagagacctcagctcttcgtgtgccttatgtcggatcaaccactgaggaaaggacagatatgaaacttgctttcgttagatctccaagccgatccctcaaatcagcagtcaggattgccacagtctactcatgggcctacggggatgatgagaaatcatggagtgaagcttggatgctagctaggcagagagccgatatcaccttagatgaactgagaatgatcactccagtctctacatccaccaacctagctcatcggttaagggatcggagcactcaggtgaaatattcggggacatcccttgtgagggttgcaaggtacacaaccatctccaatgacaatttgtcatttgtgatatctgagaaaaaagtagataccaacttcatttaccagcaagggatgctgctcggtcttgggatccttgagaatctcttcaggttagaggccaccacaggggtatccaacacagtgctacacctgcacgtggaaacagaatgttgcgttgtacctatggttgatcacccaaggataccgagtctccgcaatatcaaagttacgaacgagctatgcacaaaccctcttatctacgacaagtcccccatcatagaacacgatgcaactcgattatactcacaaagccacaggagacatttggtggagtttgttacctggtcaacaagccagctctatcatatactggccaaatctacagcaatgtccatgattgagctgatcacaagatttgagaaagatcacatgaatgaaatagccgctctgattggcgatgacgacatcaacagtttcatcacagaatttttgcttgtggagcccagactgtttatagtttacctcggccagtgtgctgccattaattgggcttttgatatacattatcatcggccctcgggcaagtaccagatgggggaactcctctactctttactctctcggatgagcaagggagtatataagatcttcaccaatgctctgagccaccccaaagtttacaagaaattttggcgaagtggtataattgagccggttcatggcccatccctagatacacagaatttacatgtcactgtctgtgacatgatatacggatcatacgtcacctatctggatcttttgctgaatgatgagctggatgcttacccgtatttgctctgcgagagtgatgaggacgtggtcacagacaggttcgacaacattcaagccaaacatctctgtgtactggccgatgtatactgcagctccaagaggtgtccctcgataatcgggatgtccccgatagaaaaatgtaccatcctcacacattacatcaagggagaatcggtacaatccccgtccgggatctcatggaacactgatccccttgtagtagatcattactcatgctctctgacctaccttcgccgtggttccatcaaacaaatcagattgagagtggatcctgggtttgtgttcgaggcgttgacagacatcgacttcaaacagcctcgcaaggctaagttggatgtatcgattgtgggattgactgatttttctcccccttgggataacgtcggtgattttctagggactatcaacacattgaggcacaatctgcccgtcaccgggaccggggtctcgaactatgaagtccacgcttatcgtagaattggtctgaattcatcagcatgttataaagctgtagagatctccacgttaataaagtcatccttagaagccggggagaatggattgttcttaggagaaggctccggctcgatgctggctgcgtacaaggaagttcttaaattagcaaactgttattacaacagtggggtaacagcagagggtagggccggacagagggaaatctctccctatccctcagaggtgagcctggtagagagtcagatggggatagagagaagtgttaaagtcttgttcaatggcaaacctgaagtaacctgggtagggaccaccgattgctacaagtatataatcagtaacattcagacctctagtctgggtttcatacactcagatatcgagacactcccaaccaaggatgccgttgagaagttggaggaatttgcctctattctatctctatccctaattttgggaaaaatcggctctattacagttgtcaaaattatgcccattagcggagattttacccaaggcttcatagcctatgccattcaatatttcagggagagcctgcttgcctatccgagatatagtaacttcatctcgactgagtgttaccttattatgataggattaaaggccaatcggttgataaacccagaagccattaagcaaagcataatcagggtggggactaggactgcaccaggacttgtgagccacatattatcagagaaacagaaaggttgtattcaatcttttctgggtgatccttatgtccaaggagacttcaataagcaccttaaagctctaactcctattgagaaaatccttgtaaattgtggtctctcgatcaatggtacaaaaatctgtagggatctaatccaccatgatattgcctccggtccagacggtctgatgagctccacaattattttatacagggaactggcccatttcaaagacaatataagaagtcagcacggcatgttccacccctacccagtattggccagtagcaggcaacgtgaattgatccttcgaatagccaagaagttttgggggtatgtcttgctatattctgatgacccggcactaatcaaacaaacgatcaagagcttgaagcggaatcacctaacctttgacttacacagcaatccgtttattaagggcttatctaaagctgagaaactgctagtgcggacaagttcactcagaagggaatggttgttcactctcgatacgaaagaagtgaaagagtggttcaaattggtgggttacagtgcactcgtcagaggttaattcgcgatacatctgcccccttctcctccaccatgagactctactggcaatccaaaagattaaagaaaactacatattggataagtatctattcccagctttgtctggt

>KJ867540/Ethiopia/1994

accagacaaagctgggtaaggatagatcttataataactatgaattgccacgcttaggattaaagatcctactgccggggggaggaggaggagcaagatccctgactatggcgactctcctcaaaagcttggcattgttcaagaagaacaaagacaaagcgccgacggcatcaggttcaggaggggccatccgggggattaagaatgttatcatagtcccgattcccggagactcatccatcattacccgttcaagattgctcgacaggcttgtcagattggccggagatccagatatcaacgggtcaaagctgaccggcgtgatgatcagcatgctttctttgttcgtagagtcacccgggcaactgatccagcggatcacagatgatccagatgtcagtatccgccttgttgaggtggtccaaagtactagatctcagtccgggttgacctttgcatcacgtggtgctgatttggacaatgaggcagacatgtatttctcaactgaagggtcctcgagtgggagcaagaaaaggatcaactggtttgagaacagggaaataatagacatagaagtgcaggatgcggaagagttcaatatgttattagcctccatactggcacaagtttggattctcctggccaaagcggttacggcaccggacactgcagctgactcagaattgagaaggtgggttaaatacacacaacaaagaagagtgattggggaatttcgccttgacaaagggtggctagatgcggtccgcaacaggattgcagaagatctatcactccggcggttcatggtatctcttatacttgacatcaaaaggacccctggcaacaagccaaggattgcagaaatgatctgtgacattgataactatattgtcgaagccgggctcgccagtttcatccttaccatcaagtttggtattgaaaccatgtatcctgcactaggtcttcacgagtttgccggggagttgtccactatagaatccttgatgaatctgtatcaacagctaggcgaagttgcaccctacatggtaattctagagaactcagttcagaacaagtttagtgcaggagcctatcctcttctctggagctatgcgatgggtgttggagtcgagctggagaactcaatgggggggttgaactttggtagatcatattttgacccggcttattttcgtctcggacaggagatggtcagaagatccgcaggaaaggtcagctctgtaatcgcagctgagctcggcatcacagcagaggaagctaaactagtctcggaaatcgcctctcagactggggacgaaaggaccgctagagggaccgggcccagacaggcgcaggtttccttcctccagcataaaataggagagggagagtcacatgcatcggcgaccagggaagaagtcaaagctgcgaccccaaatgggcccgacgaaaaggacaaaaaacgagcacgctcaggaaggccaagaggaggaacccccgaccaactgctcctggaaatcatgcctgaagacgaggtcccgcgagggtctggacaaaaccctcgtgaggctcaacgatcggccgaggcactctttagactgcaggccatggccaagattctagagggccaggaggagggagaagacaacagtcagatatataacgacaaggatctcctcagctgagcagaagcgccatccatcgaaaccagcgacaagacaccgcccatcagtattataaaaaacttaggacccaggtccaagcggtcacaccccggcaccccagccgatctagcggagaccaccgatggcagaggaactagcttaccatgtcgtggagggactggcatgtctcaagtccctctttgcctctcccccggatctttcccccatccgagatacccttgaaaactggagagaggggcttgacccatcccaccgtggaaccccgaaccctgatatgtccgaggggaaccatcagaatatcaaccaatcatgcccaccagccatcggatcaggcgaaatcgacttgtctactgaaggtaatctcggatatagagagatcaattacgatgacagtgaggctggactcagaggagttcaggtctacggatcccaccctcaaatacagcgttaccatgtttatagccacgggcgtgaagagattgaaggactccaggatgctgactctctcatgggtcaagcagatccaccacttgctaacaccttcagtacaggagaggatggatctgacgacagcgatgtggactctggcccagatgatcccgacagacatcctctatatgaccggggatctgttgccgacaatgatgacgttaagtccacagatgtcgaaaagttagaaggtgacgacattcaagaagttcttaactctcagaagagtaaaagaggaagattccaaggcgggaaaaccttgcgggtccctgaaacacccgacgtcaagcaccccagaccatcagcccaatcaattaaaaagggcacagacgggaactcagtcttatctggaacggtgacagagtgttcatcgataagtggtgcaacccaagctgtgccagagtcaagatgcgagtcatcagagagaaatgcatttgtggagagtgtccccaaatctgcgaggagtgcaaagacgatccaggagttgacacaagaatctggtaccatagcatcaccgactcagcctaaagaaaatgactccgagtatgagtatgaggatgacctatttacagaaatgcaggacattcgtgcaagcattgccaagatccatgaagacaacaaaactatcctctcaaagcttgactctatactattattgaaaggagaaatcgatactatcaagaagcaaatcagcaaacagaatataagtatatctaccattgagggccatctttccagtataatgatagccatcccgggttttggaaaggaggtcaaggacccaacatccgaggttgagttgaacccggatttaagacctataatcagccgtgattctggcagggctctcgcggaggtcctcaagaaacctgctgttgataggtctcccaaaaccggattcaaagtcaactctggttcaaagggtcagctcctcaaggatctccaactaaaacccgttgacaaacaggcaagctctgccatagggtttgtcccgtctgaccatgaatcatccaaaagtgtcatccgctctataattaagtcaagcaagctcaacattgaacacaaagactatcttttagatttggtgaatgatgtgaaaggctccaaggatcgtaaagaattccacaagatgctaacagctattcttgccaagcaaccgtaacaccgcccctaatcaacttctcatgctcggctgaaaacagccccacaaccaggctattacaaaaaacttaggagcaagggcaaccgagcttcgcagacaagatgaccgagatctatgactttgacaaatcagcatgggacgtcaaaggatcaatagctcccatagagcctaccacttatcacgatggtcgactgataccccaggtgagagtcatcgatcctgggctaggagataggaaagatgagtgcttcatgtacttgtttctcctaggagtgatggaggataatgaccccctgtctcccccagtgggaagaacctttggttctttacctctaggggtcggtaggtcaactgctaagccagaagaactattaagggaggccactgaacttgacatagtggtgagacgcacggctggactaaacgagaagctggtattttacaacaacactccgctgtctttgttaacaccttggaggagagtcctgacaaccggaagtgtgtttagcgctaaccaggtttgtaacgcagtcaatctagtcccactcgatactccccagagattcagggttgtgtacatgaccatcactagattgtctgacaatggttattatagtgtccccgggagaatgttagagttccgctcagccaatgccgtcgccttcaatatcttggttacactgagaattgagaatgggacaaaccctagaaggtacatagtcggctcatgggagaatccagaggtcacattcatgatacatgtgggcaactttagaagaaagaagaacgacgtatattctgctgattactgcaaaatgaagattgagaagatgggtctggtttttgctttaggaggaatagggggaacaagtcttcacattagaagcaccgggaaaatgagcaagaccctccatgcacagctggggttcaagaaaatcttatgctaccccttaatggatattaatgaggatctcaatcgatatctttggcgggcagagtgccgaatagtcaaaattcaagctgtcttacagccttcagtaccccaagaattccgtgtctacgatgatgtcatcattaacgatgaccaaggcttgttcaagatcctgtaattcacttacaacatcatgacaaaggtcgcaatcttgacatctctgtttctcttaccaaacactgtcgcatgccagattcactggggcaacctatccaagatcgggattgtgggaacaggaagtgccagttataaggtgatgactaggccgagccaacaaaccctggttataaagctaatgccaaatataacagccatcgacaattgtacgaaatcagagatttcgaaatacaaaagattgctgatcacagtgttaaaacctgtagaggatgccctgtcagtgataaccaagaatgtaagaccaattcaagctctaacacccggtcgcaggactcgccgttttgccggggctgtcctggccggagtggcacttggagtcgcaacggctgctcaaataactgccggagtcgcactccatcaatctttgatgaactcccaagcaattgaaagtttaaaagccagtcttgagaagtcgaaccaggcaatagaagaaatcagacttgcaaataaggaaaccatactggcagtacagggtgtccaagattatatcaacaatgagcttatcccttctgttcataagatgtcatgtgagcttgtaggtcacacactcagtctcaagcttcttaggtattataccgagatcttgtctatattcgggcccagccttcgagacccgatagctgctgaaatatcaatccaggcactcagttatgcactaggtggagatatcaataagatcctggataagcttgggtatagcggcgaggacttccttgctatcctagaaagcaaggggataaaggcccgagtcacctatgtggatacaagagattactttataattcttagcatagcttacccgaccttatctgagatcaagggggtgatagttcataagatggaagctgtgtcatacaacatcggggcacaagaatggtacactactatccctaaatatgtagccactcagggatatttgatatcaaattttgacgagacgtcatgcgtgttcactccaggggggacagtctgcagtcagaatgctttatacccaatgagcccgttgcttcaggaatgtttcagggggtctacaaaatcgtgcgctagaaccctagtttcagggaccataagtaatcgatttatcctatcaaaagggaacttgattgccaattgtgcatcagtcctgtgcaaatgttacacaacggagacaattatcagccaagatcctgataaattactaactgttatagcctctgataaatgtcctgtagttgaagtggatggagtgacgatacaggtcggcagtagggagtacccagattctgtgtacctacataaaatagacttgggcccagccatctctctagaaaaactggatgtaggcaccaatttaggcaatgcaattacaagactggagaatgcaaaggagctcctagatgcatcagatcagatattgaagactgttaagggggtacccttcagcggcaatttgtacataggactggcagcttgcattggggtatctctagggcttgtcacattaatatgctgctgtagggggaggtgtaggaacaaggagattcctacccctaaaatcaacccagggctcaaaccagacctgaccgggacctcaaagtcgtacgtgagatcactgtagtcagaataacccgaatcatccagcgtcacacacagacatgtgcaacacaagcagtcaggggacgtagaagattcaacttccgatcatcaacctggtcttactctacgccccattgcacattggttatcaaacaaaacttaggacgaaaggttagtcaccatgtccgcacaaagagagaggatcaatgctttctacaaagacactcctcacaacaagaaccatagggtgaccctggataaggaacgtttgactattgaaagaccctatatcctgctcggggtcctactggtaatgtttctgagtctaattgggttgctggccattgcagggattagacttcaccgagccaccgtcggaactgcagagatccagggccggcttaataccaatattgagttaactgaatctattgatcatcaaactaaggatgtcctaactcccctgtttaaaatcatcggtgatgaagtcggcataaggattccacagaggttcagtgaccttgtcaagttcatctccgataagattaaattcctcaaccctgacagagaatatgatttcagggatctccggtggtgtatgaatccccccgagagagttaaaatagattttgatcagttttgtgaatacaaagccgcggataagtcgattgaacatatatttgagtcaccactcaacaggtcaaaaaagttgcgatcgttgactcttgggcctggagcgggctgtcccggcagggcagtaacaagggcccagttctcagagcttactctgaccctgttggatctggatctcaatatgaagcacaacgtgacctcagtgtttaccatagttgaagagggattattcggaagaacatacaccgtctggagatcagatgccggaaatctgagcacccgaccaggtatcggccattttctaagagtcttcgagatcggactggtaagagatttcgggctaagtgcccctgtttcccatatgactaactatctcacagtgaacatgagtgatgactatcggagctgtctattagcagtgggggagttgaagctgacagctctatgcacccattctgagactgtgactctgagtgagagaggagttcctaagagagagcctcttgcggttgtaatacttaatctagctgggcctactctagggggcgaactatacagtattttgcctgcctctgacctcatggtagagaaactctatttctcatcacatagagggattatcaaagacaacgaggccaattgggtagtaccgtctaccgatgtacgtgatcttcaaaacaaaggagaatgcttggtggaagcatgcaaaactcgacctccttcattttgcaatggtacagggttaggcccatggtcagaggggagaatccctgcctacggggtgatcagagtcagtcttgacgtagctagtgatccagatgtggttattacctcagtatttggccccttgatacctcatctatctggcatggatctttacaacaacccattctcaagagccgtgtggttggctgtaccaccttatgagcagtcatttctgggaatgataaatacaattgcattcccaaatagagcagaggttatgccgcacattctgaccacagagatcagagggcttcggggtcgttgtcatgttcccatagagttgtcccgcaggattgatgatgatattaagattggctccaacatggttgtactaccgacgaaggacctgaggtacattacagccacttatgatgtttccaggagcgatcatgcaatcgtgtactatatctatgacacaggtcgctcatcatcttacttcttccccgctcgattaaagctcaaaggcaaccctctctctctgaggatagagtgtttcccctggtatcataaggtgtggtgctaccatgactgcctcatatacaacaccaccacaaacgaagaagtccatacgagagggctaaccggcatagaggtaacatgtaatccagtctgagcagagctgcgaccatcaatcaagcaagcttccgagccattcccaagccaaagcagcacagctaggggcactcaacagcacaactcagccgacagtgttataaaaaacttaggagccagggctataggagccatggactccctgtcagtcaatcaagtcctataccctgaggtccatttagacagccccattgtcacaaacaaactagttgccatccttgaatattcgggcatcgaccacaactatgttcttgaggaccagactcttatcacgaatattagatatagactggggtgcggtttttcaaatcaaatgatcatcaataacaggggggtaggtgaaacagtcaattccaaacttaaaagttacccccgtagttgtcatgtcatatatccggattgcaataaagatctgttttgtatcagggacggctgcatatctaggaagctctctgagctattcaagaagggtaattccctgtactctaagataagtcaccaggtactagattgtctcagaagagtcaacggcaaaatgggcctgggcacagatcttagccatggcctgaaggagggtatcctcgatttagggttgcacatgcatagctctcagtggtttgagaccttcctattctggttcactatcaagactgaaatgagatcaatgatcaaagaacaatcccatatatgccacaggaggaggtataacccaacctttgtctcaggagatgcattcgaggtgcttgtatcacgagacctcgttgtgataattgacaagaatactcagtgtgtctattacctgacattcgagctggtccttatgtattgtgatgtcatagagggcaggcttatgacggagaccgctatggctatagaccagaggtattcagaactgctaagccgggtcaaatacttgtgggatcttattgatgggttcttcccaacactaggtaacgccacataccaagtcgttgctctgcttgaaccattatcgttggcttaccttcaacttcaggatgtcactctggagttaagaggtgcattcttggatcactgcttcaaagaactctatgagatactggaacattgtggcgttgacacggaaagcacctacgattccatcatcgaaggattggattacgtatttatcacccacgatatccacttaactggggagattttctcctttttccggagtttcggacacccccgcctcgaagcggtcaccgctgcagagaatgtcagaaaacatatgaatcaacctaaggtaatcagttatgagactatgatgaaagggcacgcagtgttttgcggaatcatcataaatgggtttagggaccgccatggcggcagctggccccccgttgtgctgccagaacatgcctctgctgcaattcggaatgcgcaggcatccggtgagggactgacccatgacctgtgtatagacaactggaagtccttcgttggattcaggtttggctgttttatgccgctcagcctagatagtgacttgaccatgtacctcaaagacaaagcactagctgcactgaaaaatgagtgggattcagtttatccgaaagaatacctccgctataacccacctagagggacagagtcaaggcgactggtagaggtgttcctgaatgactccagctttgatccttataacatgataatgtacgtggtgaatggatcctaccttgaagaccatgagtttaatctctcatacagcttgaaagagaaagagataaaagagacagggcgattgtttgcaaaaatgacttacaaaatgcgggcctgccaggtcattgcggaaaatctgatatcaaatggagtagggaagtatttccgagacaatggaatggcgaaagatgagcatgacctaacaaaggcccttcataccttggcagtctcaggcgttcccaaaaacaacaaagataatcaccggggtgggcctcctagaaggaccacaagccaaaagatgaggtcagatcgaggcatcaccagacaagatagggacaaggtacaggaagagcccatgtataactatctgcgatgccaaccagtcagccctgatcagggtgagtcatacgagactgttagtgcattcatcaccgctgaccttaagaaatattgcctgaactggagatacgagacaatcagcatatttgcacagagactgaatgagatttacggactaccatctttctttcagtggttacacagggtattagaaaaatctgtgctgtacgtcagtgacccacattgccctcccgacttagatgatcatatccctctggacagtgtccccaatgcccaaatattcattaagtacccaatgggcggaatagaaggttattgtcaaaaattatggacaatcagtactataccatacttgtatctagcagcccatgagagcggagtaagaatcgcctctctagtgcaaggtgacaatcaaacaattgcagtgacaaaaagagtcccgagttcttggccttattcactaaaaaagagggaggcatccagagcagctcaaaattacttcgtggttttaagacagaggttgcacgatgtaggtcatcacttaaaggccaatgaaactatagtatcttcccacttttttgtatactctaaagggatttattatgatggcctgttagtctcacaatcactaaagagcattgccagatgtgtcttctggtccgagactattgtggatgaaaccagagccgcctgcagcaatattgcaacaactgtcgccaagagtatagagaggggttatgataggtaccttgcttactccttgaatatactcaaaattctccaacagatccttatatcccttaacttcactatcaacacgacaatgactcaagatgtcgtggcaccgatcatcgagaacggtgatctactaacaaggatggcacttttgccagcacccatcgggggccttaactaccttaacatgagcaggttatttgtgagaaatatcggtgacccagtcacttcctctatagccgacctgaagaggatgatagacgctgggctaatgccagaagaaacattgcaccaggtgatgacccaaaccccgggagaatcatcttaccttgattgggcaagtgacccttattctgccaacctaacctgtgtacagagtataactcgccttctcaagaacatcactgcaaggtacattttaaccagcagcccaaatccgatgctaaaagggttattccatgaggggagcagagatgaagatgaagagcttgcgagtttcttgatggatcggcatataattgttcccagagctgcacatgaaatcttagaccatagcataactggagcaagagaagctatcgccggtatgttagataccaccaagggtctgattagaacaagtatgaaacgaggtggccttaccccacgggtcttagctcgcctttccaattatgattatgaacaattcagatccggtataacattattgacgaagaagggacagtgttatctcattgacaaagattcgtgctcggtgcagcttgctatagccctgaggagccatatgtgggctaggttggcccgcgggagacctatctatgggttggaagtgcctgatgtactggaatcgatgaatggctatcttatcaaacgccatgaggcttgtgccatttgcgagacgggctcaagtcattacgggtggttcttcgtccctgcagggtgccagcttgacgatgtctcgaaagagacttcagctcttcgtgtgccttatgtcgggtcaaccacagaggaaaggacagatatgaaacttgccttcgttagatctccaagtcgatccctcaaatcagcagtcagaattgctaccgtttattcatgggcctatggggatgatgaaaaatcatggagtgaagcctggatgctggcaaggcagagagccgatatcaccctagatgagttgagaatgatcactccagtctctacatcgaccaacctagcccaccggttgagggatcggagcacccaggtgaaatattcggggacatcccttgtgagggttgcaagatacacaaccatttccaatgataatttgtcatttgtgatatctgagaagaaagtagataccaacttcatttaccagcaagggatgctcctcggtcttgggatccttgagaatcttttcaggttggaggccaccacaggggtatccaacacagtgctacatctgcacgtggagacagaatgttgtgttgtacctatggttgatcacccaagaataccgagtctccgtaacattaaggttacgaatgagctatgcacaaaccccttgatctacgacaagtctcctatcatagaacacgatgcaacccgattatactcacaaagccacagaagacatctggtggagtttgttacctggtcaacaagtcagctttatcatatactggctaagtctacagcaatgtccatgattgagttgattacaagattcgagaaggatcacatgaatgaaataaccgccttgattggcgatgacgacatcaacagttttatcacagaattcctgctagtagaacctagactgtttatagtctaccttggccagtgtgctgccatcaattgggcttttgatatacattatcatcgcccatcgggcaaataccagatgggagagcttctctactctttactctctaggatgagcaaaggagtatataagatctttaccaatgctttgagccaccccaaagtttacaagaaattttggcgaagtggtgtcatcgagccgattcatggcccgtccttagatacacagaatctacatgtcactgtctgtgatatgatatatggatcctacgtcacctatctggatcttttgctgaatgatgagctagatgattatacgtatctgctctgcgagagtgatgaggacgtggtcacagacaggttcgataacattcaagccaaacatctctgtgtattagctgatgtgtattgcagctccaaaaggtgtccctcgataatcgggatgtctcctatagaaaaatgtaccatcctcacacattacatcaagggagaatcaatacaatctccgtctgggacctcatggaacactgatcctcttgtagtagatcattactcatgctctctgacataccttcgtcgcggttccatcaaacaaatcaggttgagagtagatcctgggtttgtatttgaggcattgacagacatcgacatcaaacagcctcgcaaggctaatactgatgtatcggttgtggggttgactgatttctctcctccttgggataacattggtgattttctaaggactatcaacaccttgagacacaatctgcctgtcaccgggaccggggtctctaactatgaggtccacgcttatcgtagaattggtttgaattcatcagcatgttataaagctgtagagatctccacgttaatcaagccatccttagaagtcggagagcatgggttgttcttaggggaaggttccggttctatgctggctgcgtacaaggaagttcttaaattagccaactgttattacaacagcggagtaatagctgagggcagagccggacaaagagaaatatccccttacccctcagagatgagcttagtagagaatcaaatggggttagaaaagagtgttaaagtgctgttcaacggcaaacctgaagtaacctgggtagggactaccgattgctatgaatatataatcagtaacatccaaacctctagtctgggtttcatacactcagatattgagacactcccaaccaaagatgctgttgagaaattagaagaatttgcctcgatcttatccttatccctaattctgggaaaagttggctctattacagttatcaaagttatgcctattagcggagattttactcagagcttcataagctatgccatccaatattttagggagagcctgcttgtctatccgagatatagtaatttcatttcaactgagtgttaccttattatgataggattgaaagccaatcggttgataaacccagaagccattaagcaaagcataatcagagcagggactagaactgcaccaggactagtgagccatatattatcagagaaacaaaaagggtgtatccaatcttatctgggtgatccttatatccaaggagacttcaataaacaccttaaatctctaacccctattgagaaaatcctaattaattgtggtctctcggtcaatggcacaaaaatctgtagggatctaatccaccatgatatcgcctctggtccagacggtctgatgagttccacaattattttatacagggagctggctcatttcaaagacaacataagaagtcagcacgggatgttccacccctacccagtactggccagtagcaggcaacgtgaattaattcttcgaatcgccaagaaattctggggttatgtcttgctatattctgatgatccggcactgatcaggcaaacaattaagaacttgaagaggaaccacctaacctttgacttacacaataacccgtttattaagggtctgtccagagctgagaaattgctagtgcggacaagttcactcagaagagagtggttgttcactctcgaaacgaaggaagtgaaagaatggttcaaactggtgggttacagtgctctcgtcagaggttaattagctatacatctgcccccttctcctccaccatgaaattctactggcaatctaatagattaaagaaaactacagatcggataagtatctattccgagttttttctggt

>KJ867544/Oman/Ibri/1983

accaaacaaagttgggtaaggatagatcttataataactatgaattgtcacgcttaggattaaagatcctactgccggggggaggagtaggagcaagatccttgactatggcgactctcctcaaaagcttggcattgttcaagaagaacaaagacaaagcgccgacggcatcaggttcaggtggggccatccgggggattaagaatgttatcatagtcccgattcccggagactcatccatcattacccgttcaagattgctcgacaggcttgtcaaattggccggagatccggatatcaacgggtcaaagctgaccggcgtgatgatcagcatgctgtctttgttcgtagagtcacccgggcaactgatccagcggatcacagatgatccagatgtcagtatccgccttgttgaggtggtccaaagtactagatctcagtccgggttgacctttgcatcacgtggtgctgatttggataatgaggcagacatgtatttttcaactgaggggtccccgagtgggagcaaaaaaaggatcaactggtttgagaacagggaaataatagacatagaagtgcaggatgcagaagagttcaatatgttattagcctccatactggcacaagtttggattctcctggccaaagcggtcacggcaccggacactgcagctgactcagaattgagaaggtgggttaaatacacacaacaaagaagagtgattggggaatttcgccttgacaaagggtggctagatgcggtccgcaataggattgcagaagatctatcactccggcggttcatggtatctcttatacttgacatcaaaaggacccctggcaataagccaaggattgcagagatggtctgtgacattgataactatattgtcgaagccgggctcgcaagtttcatccttaccatcaagtttggtattgaaaccatgtatcctgcactaggtcttcacgagtttgccggggagttgtccactatagaatccttgatgaatctgtatcaacagctaggcgaagttgcaccctacatggttattctagagaactcagttcagaataagtttagtgcaggagcctatcctcttctctggagctatgcgatgggtgttggagtcgagctggagaactcgatgggggggctgaactttggtagatcatattttgacccggcttattttcgtctcggacaggagatggtcagaagatcagcaggaaaggtcagctctgtaatcgcagctgagctcggcatcacagcagaggaggccaaactagtctcggaaatcgcctctcagactggggacggaaggaccactagagggactgggcccagacaggcgcaggtttccttcctccagcataaaacaggagagggagagtcacatgcatcggtgaccagggaagaagtcacagctgagaccccaaatgggcccgacgagaaggacaagaaacgagcacgcccaggaaggccaagaggaggaacccccgaccaactgctcctggagatcatgcctgaagacgaggtcccgcgagggcctggacaaacccctcgtgaggctcaacgatcggcggaggcactctttagactacaggccatggccaagattctagagggccaggaggagggagaagacaacagtcagatatacaacgacaaggatctcctcagctgagcagaagcgccctccatcgaaaccagtgacaagacaccgcccatcagtattataaaaaacttaggacccaggtccaagcggtcacaccccggcaccccagccgaccgagcggagaccaccgatggcagaggaacaagcataccatgtcaacaagggactggaatgtatcaagtccctcaaagcctctcccccggatctatccgccatcagagatacccttgaaaactggagagaggggcttgacccatcggaccgtgcaacaccgaaccctaatatgtccgaggggaaccatcagaatatcaaccaatcatgctcaccagcaatcggatcaggcaagatcgacgtgtctactgaaggtaatctcagatatagagagatcaattacgatgacagtgaggctggactcagaggagttcaagacagaggatccgactctcaagtacagcgttaccatgtttatagccacgggggtgaagagattgaaggactcgaggatgctgactctctcgtggttcaagcagatcctccacttgctaacaccttcagtagaggagaggatggatctgacgacagcgatgtggactctggcccagatgatcccgacagagatcctctatatgaccggggatctgttgccggcaatgatgtcgttaagtccaccgatgtcgaaaaattagaaggtgacaacattcaagaagttcttaactctcagaagagtaaaagaggaagattccaaggcgggaaaactttgcgagtccctgaaacacccgatgtcaaccaccccagaccatcggcccaatcaattaaaaagggcacagacgggaactcagtcttatctggaacggtgacagagtgttcatcgataagtggtgcaacccaagctgtgccagagtcaagatgggagtcatcagagcgaaatgcatttgtggagagtgtccccaaatctgcgaggagtgcaaagacgacccaggggttgacacaagaatctggtaccatagcaccaccgactcagcctaaagagaatgactccgagtatgagtatgaggatgatctatttacagaaatgcaggacattcgtgcaagcattgccaagatccatgatgacaacaaaactatcctctcaaagcttgactctatactattactgaaaggagaaatcgatactatcaagaagcaaatcagcaaacagaatataagtatatctaccattgagggtcatctttccagtataatgatagccatcccgggttttggaaaggaggtcaaggacccaacatctgaggttgagttgaacccggatttaagacctataatcagccgtgattctggcagggctcttgcggaggtcctcaagaaacctgctgttgataggtctcccaaaaccggaatcaaggtcaactctggttcaaagggtcagctcctcaaggatctccaactaaaacccgttgacaaacaggcaagctctgccatagggtttgtcccgtctgaccatgaatcatccagaagtgtcatccgctctataatcaagtcaagcaagctcaacattgatcacaaagactatcttttagacttactgaatgatgtgaaaggctccaaggatcttaaagaattccacaagatgctaacagctattcttgccaagcaaccgtaatgcaccgcccaatcaacttcccatgctcggctgaaaacagctcctcaaccaggctattacaaaaaacttaggagcaagggcaaccgagcttcgcagacaagatgaccgagatctatgacttcgacaaatcagcatgggacgtcaaaggatcaatagctcccatagagcccaccacttatcacgatggtcgactggtaccccaggtgagagtcatcgatcccgggctaggagataggaaagatgagtgcttcatgtacctgttcctcctaggggtgattgaggataatgaccccctgtctcccctagtgggaagaacctttggttctttacctctgggggtcggcaggtcaactgctaagccagaagaactattaagggaggccactgaacttgacatagtggtgagacgcacggctggactaaacgagaagctggtattttacaacaacactccgctgtccttgttaacaccttggaggagagtcctgacaaccggaagtgtgttcagcgctaaccaggtttgtaacgcagtcaatctagtcccactcgatactccccagagatttagggttgtgtacatgaccatcactagattgtcggacaacggttattatagtgttcccaggagaatgttagagtttcgctcagccaatgccgtcgccttcaatatcttggttacactgagaattgagaatgggacaaaccctagaaggtacatagtcggctcatgggagaatccagaggtcacattcatgatacacgtgggcaactttagaagaaagaagaacgacgtatattctgctgattactgcaaaatgaagattgagaagatgggtctggtttttgctttgggaggaatagggggaacaagtcttcacattagaagcaccgggaaaatgagcaagaccctccatgcacagctggggttcaagaaaatcttatgctaccccttaatggatattaatgaggatctcaatcgatatctttggcgggcagagtgccgaatagtcaaaattcaagctgtcttacagccttcagtaccccaagaattccgtgtctacgatgatgtcatcattaacgatgaccaaggcttgtttaagatcctgtaattcactcacaacatcatgacaagagtcgcaatcttgacatctctgtttctcctcaccaacactgtcgcatcccagattcactggggcaacctatccaagatcgggattgtgggaacaggaagtgccagttataaggtgatgactaggccgagccaacaaaccctggttataaagctaatgccaaatgtaacagccatagacaattgtacgaaatcagagatttcggaatacaaaagattgctgatcacagtgttaaaacctgtcgaagatgctctgtcagtgataaccaagaatgtaagaccaattcaagctctaacacccggtcgcaggacccgccgttttgccggggctgtcctggccggagtggcacttggagtcgcaactgctgctcagataactgccggagtcgcacttcatcaatctttgatgaactcccaagcaattgaagggttaaaagctagtcttgagaagtcaaatcaggcaatagaagaaatcagacttgcaaataaggaaaccatactggcagtacagggtgtccaagattatatcaacaatgagctagtcccttctgttcataaaatgtcatgtgagttggtaggtcacaaactcagcctcaagcttcttaggtattacaccgagatcttgtctatattcgggcccagccttcgagacccgatagctgctgaaatatcaatccaagcactcagttatgcactaggtggagatatcaataagatcctggataagcttgggtatagcggcggggacttccttgctatcctagaaagtaaggggataaaggcccgagtcacctatgtggatacaagagattactttataattcttagcatagcttacccgaccttatctgagatcaagggggtgatagttcataaaatggaagctgtgtcatataacatcggggcacaagaatggtacactactatccctaaatatgtagccactcagggatacctgatatcaaattttgatgagacgtcatgcgtgttcactccaggagggacagtctgcagtcagaatgctttatacccaatgagcccgttgcttcaggaatgtttcagggggtctacaaaatcgtgcgctagaaccctagtttcagggaccataagcaatcgatttatcctatcaaaagggaacttgattgccaattgtgcatcagtcctgtgcaaatgttacacaacggagacaattatcagccaagatcctgataaactactaactgttatagcctccgataaatgtcctgtagttgaagtggatggagtgacgatacaggtcggcagtagagagtacccagattctgtgtacctacataaaatagacttgggcccagccatctctctagaaaaactggacgtaggcaccaatttaggcaatgcagttacaagactggagaatgcaaaggagctcctagatgcatcagatcagatattgaagactgttaagggggtacccctcagcggcaacttgtacataggactggcagcttgcattggggtatctctagggcttgtcacattaatatgctgctgtaaggggaggtgtaggaacaaggagattcctacccctaaaatcaacccagggctcaaaccagacctgacagggacctcaaagtcgtacgtgagatcactgtagtcagaataacccgaatcatccatcgtcacacacagacatgtgcaacacaagcagtcaagggtcgtagaagattcaacttccgatcatcaacctggccttactctacgccccattgcacattggttatcaaacaaaacttaggacgaaaggttagtcaccatgtccgcacaaagagagaggatcaatgctttctacaaagacactcctcacaacaagaaccatagggtgaccctggataaggaacgtttgactattgaaagaccctatatcctgctcggggtcctactggtaatgtttctgagcctaattgggctgctggccattgcagggattagacttcaccgagccaccgtcggaactgcagagatccagagccggcttaataccaatattgagttaactgaatccattgatcatcaaactaaggatgtcctaactcccctgtttaaaatcatcggtgatgaagtcggcataaggattccacagaggttcagtgaccttgtcaagttcatctccgataagattaaattcctcaaccctgacaaagaatatgatttcagggatctccggtggtgtatgaatccccccgagagagttaaaatagattttgatcagttttgtgaatacaaagccgcgggtaagtcgattgaacatatatttgagtcaccactcaacaggtcaaaaaggttgcgatcgttaactctagggcctggaacgggctgtcctggcaaggcagtaacaagggcccagttctcagagcttactctgaccctgttggatctggatctcaatatgaagcacaacgtgtcctcagtgtttaccataattgaagagggattattcggaagaacatacaccgtctggagatcagatgccggaaatctgagcaccggctcaggtattggcaattttctgagagtcttcgagatcggactggtaagagatttcgggctaagtgcccctgttttccatatgactaactatctcacagtgaacatgagtgatgactatcggagctgtctattagcagtaggggagttgaagctggcagctctatgcactcgttctgagactgtgactctgagtgatagaggagttcctaagagagagcctcttgtggttgtaatacttaatctggccgggcctactctagggggcgaactatatagtattttgcctgcctctgacctcatggtagagaaactctatttgtcatcacatagagggattatcaaagacaacgaggccaattgggtagtaccgtctaccgatgttcgtgatcttcaaaccaaaggagaatgcttggtggaagcatgcaaaactcgacctccttcattttgcaatggtacagggttaggcccatggtcagaggggagaatccccgcctacggggtgatcagagtcagtcttgacgtagctagtgatccagatgtggttattacctcagtatttggccccttgatacctcatctatctggcatggatctttacaacaacccattctcaagggctgtgtggttggctgtaccaccttatgagcagtcatttctgggaatgataaatacaattgccttcccagatagagcagaggttacgccgcacattctgaccacagagatcagagggcttcggggtcgttgtcatgttcctatagaattgtcccgcaggattgatgatgatattaagattggctccaacatggttgtactaccgacgaaggacctgaggtacattacagccacttatgatgtttccaggagcgatcacgccattgtgtactatatctatgacacaggtcgctcatcatcttacttcttccccgcccgattaaagatcaaaggcaaccctctctccctgagaatagagtgtttcccctggtatcataaggtgtggtgctaccatgactgcctcatatacaacaccatcacaaacgaagaagtccatacgagagggctaaccggcatagaggtaacatgtaatccagtctgagcagagctgcgaccatcaatcaagcaggcttccgagccattcccaagccaaagtagcacagctaggggcacacaacagcacaactcagccgacagtgttataaaaaacttaggagccagggctataggagccatggactccctgtcagtcaatcaggtcctataccctgaggtccatttagacagccccattgtcacaaacaagctagttgccatccttgagtattcgggcatcgaccacaactatgttcttgaagaccagacccttatcaagaatattagatatagactggggtgcggtttttcaaatcaaatgatcatcaataacaggggggtaggtgaaacagtcaattccaagcttaaaagctacccccgtagttgtcatgtcatatatccggattgcaataaggatctgttttgtatcaaggacggctgcatatctagaaagctctctgagctattcaagaagggtaattccctgtactccaagataagtcaccaggtactagattgtctcaggagagtcaacggcaaaatgggcctgggcacagatcttaaccatggcctgaaggagggtatcctcgatttagggttgcacatgcatagctctcagtggtttgagactttcctgttctggttcactatcaagactgaaatgagatccatgatcaaagaacaatcccatatatgccacaagaggaggtataacccaacctttgtctcaggagatgcattcgaggtgcttgtatcacgagacctcgttgtgataattgacaagaatactcagtgtgtctattacctgacattcgagctggtccttatgtattgtgatgtcatagagggcagacttatgacggagacagctatggctatagaccagaggtattcagaactgttaagccgggtcaaatacttgtgggatcttattgatgggttcttcccaacactaggtaacgccacataccaagtcgttgctctgcttgaaccattatcgttggcttaccttcaacttcaggatgtcactctggagttaagaggcgcatttttggatcactgcttcaaagagctctatgagatactggaacattgtggcgttgacacggaaagcacctacgattccatcatcgaaggattggattacgtatttatcacccacgatatccacttaactggggagattttctcctttttccggagtttcggacacccccgcctcgaagcggtcaccgctgcagagaatgtcagaaaacacatgaatcaacctaaggtaatcagttatgagactatgatgaaagggcacgcagtattttgcggaatcatcataaatgggtttagggaccgccatggcggcagctggccccccgttgtgctgccagaacatgcctctgctgcaattcggaatgcgcaggcatccggtgaggggctgacccatgacctgtgtatagacaactggaagtccttcgttggattcagatttggctgttttatgccgctcagcctagatagtgacctgaccatgtacctcaaagacaaagcactagctgcactgaaaaatgagtgggattcagtctacccgaaagaatacctccgctataacccacctagagggacagagtcaaggcgactggtggaggtgttcctgaatgactccagctttgatccttataacatgataatgtacgtggtgaacggttcctaccttgaagaccacgagtttaatctctcatacagcttgaaagagaaagagataaaagagacagggcgattgtttgcaaaaatgacttacaaaatgcgggcctgccaggtcattgcggaaaatctgatatcaaatggggtagggaagtatttccgagacaatggaatggcgaaagatgagcatgacctaacaaaggctcttcataccctggcagtctcaggcgttcccaaaaacaacaaagataatcaccggggtgggcctcccagaaggaccacaagccaaaagatgaggtcagatcgaggcatcaccagacaagatagggacaaggtacagggagagcccatgtataactatctgcgatgccaaccagtcagccctgatcaaggtgagtcatacgagactgttagtgcattcatcaccgctgatcttaagaaatattgcctgaactggagatacgagacaatcagcatatttgcacagagactgaatgaaatttacggactaccatctttctttcagtggttacacagggtattagaaaaatctgtgctgtacgtcagtgacccgcattgccctcccgacttagatgatcacatccctctggacagtgtccccaatgcccaaatattcatcaagtacccaatgggcggaatagaaggttattgccaaaaattatggacaatcagtactataccgtacttgtatctagcagcccatgagagcggagtaagaatcgcctctctagtgcaaggtgacaatcaaacaattgcagtgacaaaaagagtcccgagttcttggccttattcactaaaaaagagggaggcatccagagcagctcaaaattacttcgtggttttaaggcagagattgcacgatgtaggtcatcacttaaaggccaatgaaaccattgtatcttcccacttttttgtatactccaaagggatttattatgatggcctgttagtctcacagtcactaaagagcattgccagatgtgtcttctggtccgagactattgtggatgaaaccagggccgcctgcagcaatattgcaacaactgtcgccaagagtatagagaggggttatgataggtaccttgcttactctttgaatatactcaaaattctccaacagatccttatatcccttaacttcactatcaatacaacaatgactcaagatgtcgtggcaccgatcatcgagaacggtgatctactaacaaggatggcacttttgccagcacccatcgggggccttaattaccttaacatgagcaggttatttgtgagaaatatcggtgacccagtcacttcctctatagccgacctgaagaggatgatagacgctgggctaatgccagaagaaacattgcaccaggtgatgacccaaaccccgggagaatcatcttaccttgattgggcaagtgatccttattctgccaacctaacttgtgtacagagtataactcgccttctcaagaacatcactgcaaggtatattttaaccagcagcccaaatccgatgctaagagggttattccatgaagggagcagagatgaagatgaagagcttgcgagtttcttgatggatcggcatataattgttcccagagctgcacatgaaatcttagaccatagcataactggagcaagagaagctattgccggtatgttagataccaccaagggtctgattagaacaagtatgaaacgaggtggccttaccccccgggtcttagctcgcctttccaattatgattatgaacaattcagatccggtataacattattgacgaagaagggacagtgttatctcattgacaaagattcgtgctcggtgcagcttgctatagccctgaggagccatatgtgggctaggttggcccgcgggagacctatctatgggttggaggtgcctgatgtactagaatcgatgaatggctatcttatcaaacgccacgaagcttgtgccatttgcgaaacaggctcaagtcattacgggtggttcttcgtccctgcagggtgccagcttgacgatgtctcgaaagagacttcagctcttcgtgtgccttatgtcgggtcaaccacagaggaaaggacagatatgaaacttgccttcgttcgatctccgagtcgatccctcaaatcagcagtcagaattgctaccgtttattcatgggcctacggggatgatgaaaaatcatggagtgaagcctggatgctggctaggcagagagccgatatcaccctcgatgagttgagaatgatcactccaatctctacatcgaccaacctagcccatcggttgagggatcggagcacccaggtgaaatactcggggacatcccttgtgagggttgcgagatacacaaccatttccaatgataatttgtcatttgtgatatctgagaagaaggtagataccaacttcatttaccagcaagggatgctcctcggtcttgggatccttgagaatcttttcaggttggaggctaccacaggggtatccaacacagtgctacatctgcacgtggagacagaatgctgcgttgtacctatggttgaccacccaaggataccgagtctccgtaacattacggttacgaatgagctatgcacaaaccccttgatctacgacaagtctcctatcatagaacacgatgcaacccgattatactcacaaagccacagaagacatctggtggagtttgttacctggtcaacaagtcagctttaccatatactggctaagtctacagcgatgtccatgattgagttgattacaagattcgagaaggatcacatgaatgaaataacagccttgattggcgatgacgacatcaacagtttcatcacagaattcctgttagtagaacctagactgtttatagtctaccttggccagtgtgctgccatcaattgggcttttgatatacattatcatcgcccatcgggcaaataccagatgggagagcttctctactctttgctctctaggatgagcaaaggagtatacaagatctttaccaatgctttgagccaccccaaggtttacaagaaattttggagaagtggtgtcatcgagccgattcatggcccgtccttagatacacagaatctacatgtcactgtctgtgacatgatatatggatcctacgtcacctatctggatcttttactgaatgatgagctagatgattatacgtatctgctctgcgagagtgatgaggacgtggtcacagacaggttcgataacattcaagccaaacatctctgtgtattagccgatgtttattgcagctccaaaaagtgtccctcgataatcgggatgtctcctatagaaaaatgtaccatcctcacacattacatcaagggagaatcaatacaatccccgtctgggacctcatggaacactgaacctcttgtagtagatcattactcatgctctctgacataccttcgtcgcggttccatcaaacaaatcaggttgagagtagatcctggatttgtattcgaggcactgacagacatcgacaccaaactgcctcgaaaggctaatactgatgtatcggttgtagggttgactgatttctcccctccttgggataacattggtgattttctaaggactatcaacaccttgagacacaatctgcctgtcaccgggaccggggtctctaactatgaggtccacgcttatcgtaggattggtttgaattcatcagcatgttataaagctgtagaaatctccacgttaatcaaaccatccttagaagtcggagagcatgggttgttcttaggggaaggttccggttctatgctggctgcgtataaggaagttcttaaattagccaactgttattacaacagcggagtaatagctgaggggagagccggacagagagaaatatccccttacccttcagagatgagcctagtagagaaccaaatggggttagaaaggagtgttaaagtgctgttcaacggcaaacctgaagtaacttgggtcgggaccaccgattgctacgaatatatcatcagtaacatccaaacctctagtctgggtttcatacactcagatattgagacactcccaaccaaagatgctgttgagaaattagaagaatttgcctctatcttatccttatccctaattttgggaaaaattggctctattaccgttatcaaagttatgcctattagcggagattttactcaaggcttcataagttatgccatccaatattttagggagagcctgcttgtctatccgagatatagtaatttcatttcaactgagtgttaccttattatgataggattgaaagccaatcggttgataaacccagaagccattaagcaaaacataatcagagcggggactagaactgcaccaggactagtgagccatatattatcagagaaacaaaaagggtgtatccaatcttatctgggtgatccttatatccaaggagacttcaataaacaccttaaatctctaacccctattgagaaaatcctagttaattgtggtctctcgatcaatggcacaaaaatctgtagggatctaatccaccatgatatcgcctctggtccagacggtctgatgagctccacaattattttgtatagggagctggcacatttcaaagacaacataagaagtcagcacgggatgttccacccctacccagtactggctagtagcaggcaacgtgaattaatccttcgaatcgccaagaaattctggggttatgtcttgctatattctgatgatccggcactgatcaggcaaacaattaagaacttgaagagaaaccacctaacctttgacttacacagtaacccgtttattaagggtctgtccagagctgagaaaatgctagtgcggacaagttcactcagaagagagtggttgttcactctcgaaacgaaggaagtgaaagagtggttcaaattggtgggttacagtgctctcgtcagaggttaattagctatacatctgcccccttctcctccaccatgaaattctactggcaatctaatagattaaagaaaactacagatcggataagtatctattcccagctttgtctggt

>KJ867545/United_Arab_Emirates/1986/Dorcas_gazelle

accaaacaaagttgggtaaggatagttcttataataactatgaattgccacgcttaggattaaagatcctactgccggggggaggagtaggagcaagatccttgactatggcgactctcctcaaaagcttggcactgttcaagaagaacaaagacaaagcgccgacggcatcaggttcaggtggggccatccgggggattaagaatgttatcatagtcccgattcccggagactcatccatcattacccgttcaagattgctcgacaggcttgtcagattggccggagatccggatatcaacgggtcaaagctgaccggcgtgatgatcagcatgctgtctttgttcgtagagtcacccgggcaactgatccagcggatcacagatgatccagatgtcagtatccgccttgttgaggtggtccaaagtactagatctcagtccgggttgacctttgcatcacgtggtgctgatttggataatgaggcagacatgtatttttcaactgaggggtccccgagtgggagcaaaaaaaggatcaactggtttgagaacagggaaataatagacatagaagtgcaggatgcagaagagttcaatatgttattagcctccatactggcacaagtttggattctcctggccaaagcggtcacggcaccggacactgcagctgactcagaattgagaaggtgggttaaatacacacaacaaagaagagtgattggggaatttcgccttgacaaagggtggctagatgcggtccgcaataggattgcagaagatctatcactccggcggttcatggtatctcttatacttgacatcaaaaggacccctggcaacaagccaaggattgcagagatggtctgtgacattgataactatattgtcgaagccgggctcgccagtttcatccttaccatcaagtttggtattgaaaccatgtatcctgcactaggtcttcacgagtttgccggggagttgtccactatagaatccttgatgaatctgtatcaacagctaggcgaagttgcaccctacatggttattctagagaactcagttcagaataagtttagtgcaggagcctatcctcttctctggagctatgcgatgggtgttggagtcgagctggagaactcgatgggggggctgaactttggtagatcatattttgacccggcttattttcgtctcggacaggagatggtcagaagatcagcaggaaaggtcagctctgtaatcgcagctgagctcggcatcacagcagaggaggccaaactagtctcggaaatcgcctctcagactggggacggaaggaccactagagggactgggcccagacaggcgcaggtttccttcctccagcataaaacaggagagggagagtcacatgcatcggtgaccagggaagaagtcacagctgagaccccaaatgggcccgacgagaaggacaagaaacgagcacgcccaggaaggccaagaggaggaacccccgaccaactgctcctggagatcatgcctgaagacgaggtcccgcgagggcctggacaaacccctcgtgaggctcaacgatcggcagaggcactctttagactacaggccatggccaagattctagagggccaggaggagggagaagacaacagtcagatatacaacgacaaggatctcctcagctgagcagaagcgccctccatcgaaaccagtgacaagacaccgcccatcagtattataaaaaacttaggacccaggtccaagcggtcacaccccggcaccccagccgaccgagcggagaccaccgatggcagaggaacaagcataccatgtcaacaagggactggaatgtatcaagtccctcaaagcctctcccccggatctatccgccatcagagatacccttgaaaactggagagaggggcttgacccatcggaccgtgcaacaccgaaccctgatatgtccgaggggaaccatcagaatatcaaccaatcatgctcaccagcaatcggatcaggcaagatcgacgtgtctactgaaggtaatctcagatatagagagatcaattacgatgacagtgaggctggactcagaggagttcaagacagaggatccgactctcaagtacagcgttaccatgtttatagccacgggggtgaagagattgaaggactcgaggatgctgactctctcgtggttcaagcagatcctccacttgctaacaccttcagtagaggagaggatggatctgacgacagcgatgtggactctggcccagatgatcccgacagagatcctctatatgaccggggatctgttgccggcaatgatgtcgttaagtccaccgatgtcgaaaaattagaaggtgacaacattcaagaagttcttaactctcagaagagtaaaagaggaagattccaaggcgggaaaactttgcgagtccctgaaacacccgatgtcaaccaccccagaccatcggcccaatcaattaaaaagggcacagacgggaactcagtcttatctggaacggtgacagagtgttcatcgataagtggtgcaacccaagctgtgccagagtcaagatgggagtcatcagagcgaaatgcatttgtggagagtgtccccaaatctgcgaggagtgcaaagacgacccaggggttgacacaagaatctggtaccataacaccaccgactcagcctaaagagaatgactccgagtatgagtatgaggatgatctatttacagaaatgcaggacattcgtgcaagcattgccaagatccatgatgacaacaaaactatcctctcaaagcttgactctatactattactgaaaggagaaatcgatactatcaagaagcaaatcagcaaacagaatataagtatatctaccattgagggtcatctttccagtataatgatagccatcccgggttttggaaaggaggtcaaggacccaacatctgaggttgagttgaacccggatttaagacctataatcagccgtgattctggcagggctcttgcggaggtcctcaagaaacctgctgttgataggtctcccaaaaccggaatcaaggtcaactctggttcaaagggtcagctcctcaaggatctccaactaaaacccgttgacaaacaggcaagctctgccatagggtttgtcccgtctgaccatgaatcatccagaagtgtcatccgctctataatcaagtcaagcaagctcaacattgatcacaaagactatcttttagacttactgaatgatgtgaaaggctccaaggatcttaaagaattccacaagatgctaacagctattcttgccaagcaaccgtaatgcaccgcccaatcaacttcccatgctcggctgaaaacagctcctcaaccaggctattacaaaaaacttaggagcaagggcaaccgagcttcgcagacaagatgaccgagatctatgacttcgacaaatcagcatgggacgtcaaaggatcaatagctcccatagagcccaccacttatcacgatggtcgactggtaccccaggtgagagtcatcgatcccgggctaggagataggaaagatgagtgcttcatgtacctgttcctcctaggggtgattgaggataatgaccccctgtctcccccagtgggaagaacctttggttctttacctctgggggtcggcaggtcaactgctaagccagaagaactattaagggaggccactgaacttgacatagtggtgagacgcacggctggactaaacgagaagctggtattttacaacaacactccgctgtccttgttaacaccttggaggagagtcctgacaaccggaagtgtgttcagcgctaaccaggtttgtaacgcagtcaatctagtcccactcgatactccccagagatttagggttgtgtacatgaccatcactagattgtcggacaacggttattatagtgttcccaggagaatgttagagtttcgctcagccaatgccgtcgccttcaatatctcggttacactgagaattgagaatgggacaaaccctagaaggtacatagtcggctcatgggagaatccagaggtcacattcatgatacacgtgggcaactttagaagaaagaagaacgacgtatattctgctgattactgcaaaatgaagattgagaagatgggtctggtttttgctttgggaggaatagggggaacaagtcttcacattagaagcaccgggaaaatgagcaagaccctccatgcacagctggggttcaagaaaatcttatgctaccccttaatggatatcaatgaggatctcaatcgatatctttggcgggcagagtgccgaatagtcaaaattcaagctgtcttacagccttcagtaccccaagaattccgtgtctacgatgatgttatcattaacgatgaccaaggcttgtttaagatcctgtaattcactcacaacatcatgacacgggtcgcaatcttgacatctctgtttctcctcaccaacactgtcgcatcccagattcactggggcaacctatccaagatcgggattgtgggaacaggaagtgccagttataaggtgatgactaggccgagccaacaaaccctggttataaagctaatgccaaatgtaacagccatagacaattgtacgaaatcagagatttcggaatacaaaagattgctgatcacagtgttaaaacctgtcgaagatgctctgtcagtgataaccaagaatgtaagaccaattcaagctctaacacccggtcgcaggacccgccgttttgccggggctgtcctggccggagtggcacttggagtcgcaactgctgctcagataactgccggagtcgcacttcatcaatctttgatgaactcccaagcaattgaagggttaaaagctagtcttgagaagtcaaatcaggcaatagaagaaatcagacttgcaaataaggaaaccatactggcagtacagggtgtccaagattatatcaacaatgagctagtcccttctgttcataaaatgtcatgtgagttggtaggtcacaaactcagcctcaagcttcttaggtattacaccgagatcttgtctatattcgggcccagccttcgagacccgatagctgctgaaatatcaatccaagcactcagttatgcactaggtggagatatcaataagatcctggataagcttgggtatagcggcggggacttccttgctatcctagaaagtaaggggataaaggcccgagtcacctatgtggatacaagagattactttataattcttagcatagcttacccgaccttatctgagatcaagggggtgatagttcataaaatggaagctgtgtcatataacatcggggcacaagaatggtacactactatccctaaatatgtagccactcagggatacctgatatcaaattttgatgagacgtcatgcgtgttcactccaggagggacagtctgcagtcagaatgctttatacccaatgagcccgttgcttcaggaatgtttcagggggtctacaaaatcgtgcgctagaaccctagtttcagggaccataagcaatcgatttatcctatcaaaagggaacttgattgccaattgtgcatcagtcctgtgcaaatgttacacaacggagacaattatcaaccaagatcctgataaactactaactgttatagcctccgataaatgtcctgtagttgaagtggatggagtgacgatacaggtcggcagtagagagtacccagattctgtgtacctacataaaatagacttgggcccagccatctctctagaaaaactggacgtaggcaccaatttaggcaatgcagttacaagactggagaatgcaaaggagctcctagatgcatcagatcagatattgaagactgttaagggggtacccctcagcggcaacttgtacataggactggcagcttgcattggggtatctctagggcttgtcacattaatatgctgctgtaaggggaggtgtaggaacaaggagattcctacccctaaaatcaacccagggctcaaaccagacctgacagggacctcaaagtcgtacgtgagatcactgtagtcagaataacccgaatcatctatcgtcacacacagacatgtgcaacacaagcagtcaagggtcgtagaagattcaacttccgatcatcaacctggccttactctacgccccattgcacattggttatcaaacaaaacttaggacgaaaggttagtcaccatgtccgcacaaagagagaggatcaatgctttctacaaagacactcctcacaacaagaaccatagggtgaccctggataaggaacgtttgactattgaaagaccctatatcctgctcggggtcctactggtaatgtttctgagcctaattgggctgctggccattgcagggattagacttcaccgagccaccgtcggaactgcagagatccagagccggcttaataccaatattgagttaactgaatccattgatcatcaaactaaggatgtcctaactcccctgtttaaaatcatcggtgatgaagtcggcataaggattccacagaggttcagtgaccttgtcaagttcatctccgataagattaaattcctcaaccctgacaaagaatatgatttcagggatctccggtggtgtatgaatccccccgagagagttaaaatagattttgatcagttttgtgaatacaaagccgcgggtaagtcgattgaacatatatttgagtcaccactcaacaggtcaaaaaggttgcgatcgttaactctagggcctggaacgggctgtcctggcaaggcagtaacaagggcccagttctcagagcttactctgaccctgttggatctggatctcaatatgaagcacaacgtgtcctcagtgtttaccatagttgaagagggattattcggaagaacatacaccgtctggagatcagatgccggaaatctgagcaccggctcaggtattggcaattttctgagagtcttcgagatcggactggtaagagatttcgggctaagtgcccctgttttccatatgactaactatctcacagtgaacatgagtgatgactatcggagctgtctattagcagtaggggagttgaagctggcagctctatgcactcgttctgagactgtgactctgagtgatagaggagttcctaagagagagcctcttgtggttgtaatacttaatctggccgggcctactctagggggcgaactatatagtattttgcctgcctctgacctcatggtagagaaactctatttgtcatcacatagagggattatcaaagacaacgaggccaattgggtagtaccgtctaccgatgttcgtgatcttcaaaccaaaggagaatgcttggtggaagcatgcaaaactcgacctccttcattttgcaatggtacagggttaggcccatggtcagaggggagaatccccgcctacggggtgatcagggtcagtcttgacgtagctagtgatccagatgtggttattacctcagtatttggccccttgatacctcatctatctggcatggatctttacaacaacccattctcaagggctgtgtggttggctgtaccaccttatgagcagtcatttctgggaatgataaatacaattgccttcccagatagagcagaggttacgccgcacattctgaccacagagatcagagggcttcggggtcgttgtcatgttcctatagaattgtcccgcaggattgatgatgatattaagattggctccaacatggttgtactaccgacgaaggacctgaggtacattacagccacttatgatgtttccaggagcgatcatgccattgtgtactatatctatgacacaggtcgctcatcatcttacttcttccccgcccgattaaagatcaaaggcaaccctctctccctgagaatagagtgtttcccctggtatcataaggtgtggtgctaccatgactgcctcatatacaacaccatcacaaacgaagaagcccatacgagagggctaaccggcatagaggtaacatgtaatccagtctgagcagagctgcgaccatcaatcaagcaggcttccgagccattcccaagccaaagtagcacagctaggggcacccaacagcacaactcagccgacagtgttataaaaaacttaggagccagggctataggagccatggactccctgtcagtcaatcaggtcctataccctgaggtccatttagacagccccattgtcacaaacaagctagttgccatccttgagtattcgggcatcgaccacaactatgttcttgaagaccagacccttatcaagaatattagatatagactggggtgcggtttttcaaatcaaatgatcatcaataacaggggggtaggtgaaacagtcaattccaagcttaaaagttacccccgtagttgtcatgtcatatatccggattgcaataaggatctgttttgtatcagggacggctgcatatctagaaagctctctgagctattcaagaagggtaattccctgtactccaagataagtcaccaggtactagattgtctcaggagagtcaacggcaaaatgggcctgggcacagatcttaaccatggcctgaaggagggtatcctcgatttagggttgcacatgcatagctctcagtggtttgagactttcctgttctggttcactatcaagactgaaatgagatccatgatcaaagaacaatcccatatatgccacaagaggaggtataacccaacctttgtctcaggagatgcattcgaggtgcttgtatcacgagacctcgttgtgataattgacaagaatactcagtgtgtctattacctgacattcgagctggtccttatgtattgtgatgtcatagagggcagacttatgacggagacagctatggctatagaccagaggtattcagaactgttaagccgggtcaaatacttgtgggatcttattgatgggttcttcccaacactaggtaacgccacataccaagtcgttgctctgcttgaaccattatcgttggcttaccttcaacttcaggatgtcactctggagttaagaggcgcatttttggatcactgcttcaaagagctctatgagatactggaacattgtggcgttgacacggaaagcacctacgattccatcatcgaaggattggattacgtatttatcacccacgatatccacttaactggggagattttctcctttttccggagtttcggacacccccgcctcgaagcggtcaccgctgcagagaatgtcagaaaacacatgaatcaacctaaggtaatcagttatgagactatgatgaaagggcacgcagtattttgcggaatcatcataaatgggtttagggaccgccatggcggcagctggccccccgttgtgctgccagaacatgcctctgctgcaattcggaatgcgcaggcatccggtgaggggctgacccatgacctgtgtatagacaactggaagtccttcgttggattcagatttggctgttttatgccgctcagcctagatagtgacctgaccatgtacctcaaagacaaagcactagctgcactgaaaaatgagtgggattcagtctacccgaaagaatacctccgctataacccacctagagggacagagtcaaggcgactggtggaggtgttcctgaatgactccagctttgatccttataacatgataatgtacgtggtgaacggttcctaccttgaagaccacgagtttaatctctcatacagcttgaaagagaaagagataaaagagacagggcgattgtttgcaaaaatgacttacaaaatgcgggcctgccaggtcattgcggaaaatctgatatcaaatggggtagggaagtatttccgagacaatggaatggcgaaagatgagcatgacctaacaaaggctcttcataccctggcagtctcaggcgttcccaaaaacaacaaagataatcaccggggtgggcctcccagaaggaccacaagccaaaagatgaggtcagatcgaggcatcaccagacaagatagggacaaggtacagggagagcccatgtataactatctgcgatgccaaccagtcagccctgatcaaggtgagtcatacgagactgttagtgcattcatcaccgctgatcttaagaaatattgcctgaactggagatacgagacaatcagcatatttgcacagagactgaatgaaatttacggactaccatctttctttcagtggttacacagggtattagaaaaatctgtgctgtacgtcagtgacccgcattgccctcccgacttagatgatcacatccctctggacagtgtccccaatgcccaaatattcatcaagtacccaatgggcggaatagaaggttattgccaaaaattatggacaatcagtactataccgtacttgtatctagcagcccatgagagcggagtaagaatcgcctctctagtgcaaggtgacaatcaaacaattgcagtgacaaaaagagtcccgagttcttggccttattcactaaaaaagagggaggcatccagagcagctcaaaattacttcgtggttttaaggcagagattgcacgatgtaggtcatcacttaaaggccaatgaaaccattgtatcttcccacttttttgtatactccaaagggatttattatgatggcctgttagtctcacagtcactaaagagcattgccagatgtgtcttctggtccgagactattgtggatgaaaccagggccgcctgcagcaatattgcaacaactgtcgccaagagtatagagaggggttatgataggtaccttgcttactctttgaatatactcaaaattctccaacagatccttatatcccttaacttcactatcaatacaacaatgactcaagatgtcgtggcaccgatcatcgagaacggtgatctactaacaaggatggcacttttgccagcacccatcgggggccttaattaccttaacatgagcaggttatttgtgagaaatatcggtgacccagtcacttcctctatagccgacctgaagaggatgatagacgctgggctaatgccagaagaaacattgcaccaggtgatgacccaaaccccgggagaatcatcttaccttgattgggcaagtgatccttattctgccaacctaacttgtgtacagagtataactcgccttctcaagaacatcactgcaaggtatattttaaccagcagcccaaatccgatgctaagagggttattccatgaagggagcagagatgaagatgaagagcttgcgagtttcttgatggatcggcatataattgttcccagagctgcacatgaaatcttagaccatagcataactggagcaagagaagctattgccggtatgttagataccaccaagggtctgattagaacaagtatgaaacgaggtggccttaccccccgggtcttagctcgcctttccaattatgattatgaacaattcagatccggtataacattattgacgaagaagggacagtgttatctcattgacaaagattcgtgctcggtgcagcttgctatagccctgaggagccatatgtgggctaggttggcccgcgggagacctatctatgggttggaggtgcctgatgtactagaatcgatgaatggctatcttatcaaacgccacgaagcttgtgccatttgcgaaacaggctcaagtcattacgggtggttcttcgtccctgcagggtgccagcttgacgatgtctcgaaagagacttcagctcttcgtgtgccttatgtcgggtcaaccacagaggaaaggacagatatgaaacttgccttcgttcgatctccgagtcgatccctcaaatcagcagtcagaattgctaccgtttattcatgggcctacggggatgatgaaaaatcatggagtgaagcctggatgctggctaggcagagagccgatatcaccctcgatgagttgagaatgatcactccaatctctacatcgaccaacctagcccatcggttgagggatcggagcacccaggtgaaatactcggggacatcccttgtgagggttgcgagatacacaaccatttccaatgataatttgtcatttgtgatatctgagaagaaggtagataccaacttcatttaccagcaagggatgctcctcggtcttgggatccttgagaatcttttcaggttggaggctaccacaggggtatccaacacagtgctacatctgcacgtggagacagaatgctgtgttgtacctatggttgaccacccaaggataccgagtctccgtaacattacggttacgaatgagctatgcacaaaccccttgatctacgacaagtctcctatcatagaacacgatgcaacccgattatactcacaaagccacagaagacatctggtggagtttgttacctggtcaacaagtcagctttaccatatactggctaagtctacagcgatgtccatgattgagttgattacaagattcgagaaggatcacatgaatgaaataacagccttgattggcgatgacgacatcaacagtttcatcacagaattcctgttagtagaacctagactgtttatagtctaccttggccagtgtgctgccatcaattgggcttttgatatacattatcatcgcccatcgggcaaataccagatgggagagcttctctactctttgctctctaggatgagcaaaggagtatacaagatctttaccaatgctttgagccaccccaaggtttacaagaaattttggagaagtggtgtcatcgagccgattcatggcccgtccttagatacacagaatctacatgtcactgtctgtgacatgatatatggatcctacgtcacctatctggatcttttactgaatgatgagctagatgattatacgtatctgctctgcgagagtgatgaggacgtggtcacagacaggttcgataacattcaagccaaacatctctgtgtattagccgatgtttattgcagctccaaaaagtgtccctcgataatcgggatgtctcctatagaaaaatgtaccatcctcacacattacatcaagggagaatcaatacaatccccgtctgggacctcatggaacactgaacctcttgtagtagatcattactcatgctctctgacataccttcgtcgcggttccatcaaacaaatcaggttgagagtagatcctggatttgtattcgaggcactgacagacatcgacaccaaactgcctcgaaaggctaatactgatgtatcggttgtagggttgactgatttctcccctccttgggataacattggtgattttctaaggactatcaacaccttgagacacaatctgcctgtcaccgggaccggggtctctaactatgaggtccacgcttatcgtaggattggtttgaattcatcagcatgttataaagctgtagaaatctccacgttaatcaaaccatccttagaagtcggagagcatgggttgttcttaggggaaggttccggttctatgctggctgcgtataaggaagttcttaaattagccaactgttattacaacagcggagtaatagctgaggggagagccggacagagagaaatatccccttacccttcagagatgagcctagtagagaaccaaatggggttagaaaggagtgttaaagtgctgttcaacggcaaacctgaagtaacttgggtcgggaccaccgattgctacgaatatatcatcagtaacatccaaacctctagtctgggtttcatacactcagatattgagacactcccaaccaaagatgctgttgagaaattagaagaatttgcctctatcttatccttatccctaattttgggaaaaattggctctattaccgttatcaaagttatgcctattagcggagattttactcaaggcttcataagttatgccatccaatattttagggagagcctgcttgtctatccgagatatagtaatttcatttcaactgagtgttaccttattatgataggattgaaagccaatcggttgataaacccagaagccattaagcaaaacataatcagagcggggactagaactgcaccaggactagtgagccatatattatcagagaaacaaaaagggtgtatccaatcttatctgggtgatccttatatccaaggagacttcaataaacaccttaaatctctaacccctattgagaaaatcctagttaattgtggtctctcgatcaatggcacaaaaatctgtagggatctaatccaccatgatatcgcctctggtccagacggtctgatgagctccacaattattttgtatagggagctggcacatttcaaagacaacataagaagtcagcacgggatgttccacccctacccagtactggctagtagcaggcaacgtgaattaatccttcgaatcgccaagaaattctggggttatgtcttgctatattctgatgatccggcactgatcaggcaaacaattaagaacttgaagagaaaccacctaacctttgacttacacagtaacccgtttattaagggtctgtccagagctgagaaaatgctagtgcggacaagttcactcagaagagagtggttgttcactctcgaaacgaaggaagtgaaagagtggttcaaattggtgggttacagtgctctcgtcagaggttaattagctatacatctgcccccttctcctccaccatgaaattctactggcaatctaatagattaaagaaaactacagatcggataagtatctattcccagctttgtctggt

>KM089830/China/Nanyang/2014

accaaacaaagttgggtaaggatagatcttataataactatggactggcaaacttaggagtaaagatcctactgtcggggggaggaggaggagcaagatctttgattatggcgactctccttaaaagcttagcattgttcaaaaggaacaaagacaaagcgccgacagcatcaggttcaggaggggccatccgggggattaagaatgttatcatagtcccgattcccggagactcgtccatcactacccgttcaagactgctcgacaggcttgtcagattggccggagatcctgacatcaacgggtcaaagctgaccggggtgatgatcagcatgttatccttgttcgtagagtcacccgggcaattgatacagcgaatcacagatgatccggatgttagtatccgccttgttgaggtagttcaaagtactaggtcccagtccgggttgacctttgcatcacgtggtgctgatttagacaacgaggcagacatgtatttttcaactgaggggccctcgagtggaggtaagaaaaggatcaactggtttgagaacagagaaataatagacatagaggtgcaggatccagaagagttcaatatgttgttagcctccatactagcacaagtctggatccttctggccaaggctgttacggcaccagatacggcagctgactcagaactgagaaggtgggttaaatacacacaacaaaggagagtgattggggaatttcgccttgacaaggggtggctggacgcagtccgcaacagaattgcagaagatctatcactccggcggttcatggtatctctcatacttgacatcaagaggaccccaggcaacaagccaaggattgcagaaatgatctgcgacattgacaactatattgtcgaagcaggactcgccagtttcatccttactatcaaatttggtatcgaaaccatgtatcctgcattagggctccacgagtttgccggagaattgtccactattgagtccttgatgaacttgtatcaacagctaggagaggttgcaccctacatggtaattctagagaattcaattcagaacaagtttagtgcaggagcttatcccctcctctggagctatgcgatgggtgtcggagtcgagctggagaactcaatggggggcttgaactttggcaggtcatattttgaccctgcctattttcgtctcggacaggagatggtcagaagatctgcaggaaaggtcagctctgtgattgcggctgagctcggcatcacagcagaggaagctaaacttgtctcggaaatcgcctcgcaggctggggacgaaagaaccgccagagggactgggcctcgacaggcgcaggtttccttccttcagcaccaaacaggagggggagagtcgtccgcaccagcgaccagagaaggggtcaaagctgcgatcccaaacggagctgaagaaagggacagaaagcaaacacgcccaggaaggcccagaggagagacctccggccaactgctcctggacatcatgccagaggatgagatcttgcgagagtctggtcaaaaccctcgtgaggctcaaagatcggccgaggcactcttcaggctgcaggccatggccaagattctggaggaccaggaggagggagaagacaacagtcaggtctacaacgacaaggatctcctcggctaagcagacgcaccctctgtcgaaatcagtgacaagacatctcttaccagtattataaaaaacttaggacccaggtccaagcaagcacacatcgacactccaaccagtcgagcggagaccaccgatggcagaagaacaagcataccatgtcaacaaggggctggaatgtatcaagtctctcaaagcctcacccccggatctatccaccatcagagataccatcgagagctggagagaggggcttagcccctcgggccgtgcaacaccgaaccctgatacgtccgagggagatcatcagaatatcaaccaatcatgctcaccagcaatcggaccaaacaaagtctacttgtctcctgaagataatctcggatttagagagatcactggcaacgactgtgaggctgggctcggaggggtccagagagaaggatccaactctcaagtacagcgttaccatgtttatagtcacgggggtgaagagattgaaggactcgaggatgctgactctctcgtggttcaagcagatcctccggttgctaacgtcttcaatggaggagaggacggatctgacgacagcgatgtggactctggcccagatgatcccggcagagatactctatatgaccggggatctgttgccggcaatggtatcgctaggtccacagatgtcgaaaaactagaaggtgctgatattcaagaagttcttaactcccagaaaggcaaaggaggaagattccagggcgggaaaaccttgcgagtcccggaaatacccgatgtcaagcactccagaccatcagcccaatcaattaaaaagggcacagacgggaactcagtctcatctggaacggtgatagagtgtttatcgataagtggtgcaacccaaactgtgccagagtcaagatgggaatcatcagagcaaaatgcgtctgtggggagtgtcctcaagtctgcgaggagtgcaaagacgatccaggggtcgacacaagaatctggtaccatagcatcactgactcagcctaaagagaatgactccgagtatgagtatgaggatgacctatttacagagattcaggacatccgtgcaagcattgccaagatccatgatgacaataaaactatcctctcaaagcttgattctatactgttattgaaaggggaagtcgacactatcaagaaacaaatcagcaagcagaatataagtatatccaccattgagggccatctctccagtataatgatagccatccctggctttgggaaggacatcaaggacccaacatccgaagtcgagttgaacccagatctaagacctataataagccgtgactctggcagagctctcgcggaggtcctcaagaaacccgctgtggataggtctcagaaaattggaaccaaagccaactccagctcaaagggtcagcttcttaaggatctccagctaaaacctgtcgacaagcaggcaagctctgcaatcgggtttgtcccgtccgaccatgaatcatccagaaatgtcatccgctccataatcaagtcgagcaagctaaatattgatcacaaggactatcttctagatttactaaatgatgtgaaaggctccaaggatcttaaggaatttcacaagatgctaacagcaattctcgccaagcacccgtaacacatcctccagtcatcatctcatactcgactaaaaacatcctttcaatcaggctattacaaaaaacttaggagcaagggcaactgagcttcgcagacaggatgaccgagatctacgacttcgataaatcagcatgggatgtcaaagggtcaattgcccgcatagaacccaccacctaccacgacggccgactgataccccaggtgagggtcatcgatcctggtctgggagacagaaaagatgagtgctttatgtacctgtttctcctaggagtgattgaggataacgaccccctgtctccccccgtcggaagaacctttggctctctacctctaggggttggtaggtcaactgccaagccagaagaactactaagggaggccacagaattagatatagtggtgaggcgcactgcaggagtaaatgagaaactggtattttacaacaacactccgctgtccttgttaacgccctggaagaaagttctgacaaccggaagtgtgtttagcgctaaccaggtttgcaatgcagtcaacctagtcccgcttgatactccccagagattcagggttgtgtacatgagcataactagattatcagacaatggttactatagtgtgcccagaagaatgctggagttccgctcggccaatgcagtagccttcaacatcttggttacactaagaattgaaaatggcacaaaccctagaagatacatagtcggctcatgggagaattcagaggtcacatttatggtacacgtgggcaactttaggagaaagaagaacgaagtatactctgctgattactgcaaaatgaagattgaaaagatgggtttagtttttgccctgggtggaataggcggaacaagtctccatattagaagcaccgggaaaatgagcaaaaccctccatgcacagctggggttcaagaaaatcttatgttaccccctaatggatgtcaatgaggatcttaaccgatatctctggcgggcagagtgccgaatagtcaaaatccaagctgtcttacagccatcagtaccccaagagttccgtgtctacgatgatgtcatcatcaacgatgatcaaggcttgttcaagatcttgtagttcatttgcaacatcatgacgcgggtcgcaattttgacatttctgtttcttttcccaaatgttgttgcgtgtcagattcactggggcaatctatccaagatcgggattgtaggaacagggagtgccagctacaaggtgatgactaggccaagccaccagactctggttataaagttaatgccaaatataacggccatcgacaattgtacaaagtcagagattgcagagtacaagagattgctgatcacagtgttaaagcctgtagaggatgctctgtcggtgataaccaagaatgtaagaccaattcaaactctaacacctgggcgtagaacccgccgttttgctggagctgttctggccggggtagcacttggagttgcgacagccgctcagataactgcaggagtcgcccttcatcaatcattgatgaactcccaagcaattgagagtttaaaaaccagtcttgagatgtcgaatcaggcaatagaagaaatcagacttgcaaataaggagaccatactggcagtacagggcgtccaggattatatcaacaatgagctcgtcccttctgttcatagaatgtcatgcgagctggtaggtcacaagctcggcctcaagctccttaggtactacaccgagatcctgtccatattcgggcccagtcttcgagacccgatatctgccgaaatatcaatccaggcacttagttatgcattaggcggagacattaataaaatcctggacaagcttgggtatagcggtggggatttccttgccatcctagaaagcaagggaataaaggcccgggttacatatgtggacgcaagagattactttataatccttagcatcgcctacccaaccttatctgagatcaagggagtgatagttcacaagatagaagctataacatacaacattggggcacaggagtggtatactactatccctaaatatgtagccactcaggggtatctgatatcgaactttgatgagacgtcatgcgtattcactccagaggggacagtttgcagccagaatgcgttgtacccaatgagcccattgcttcaggaatgtttcagggggtcaacaaaatcgtgcgccagaaccttagtttcagggaccataagtaatagatttatcctatcaaaagggaacctgattgcaaattgtgcgtcagttttgtgcaaatgttacacaacagagacagttatcagccaagaccctgacaaactactaactgttgtagcatccgacaagtgtcctgtagttgaggtggatggagtgacaatacaggtcggcagtcgagagtatccggattctgtatacttacacaaaatagacttaggtccagccatctccctagaaaaactggatgtaggcaccaatttaggcaatgcagtcacaagactggagaatgcaaaggagctcctagatgcatcagaccaaatactgaagactgttaaaggggtacctttcagtgggaatatgtacatagcactggcagcttgcataggagtatccctgggccttgtcacattaatatgctgctgtaaggggaggtgtaagaacaaggaaatccctatctccaaaatcaacccagggctcaaacccgacctgaccgggacctcaaagtcgtacgtaagatcactgtagtcagaatcacctggatcatctggcatcgcacacatacatgcacgacacaggcagtccgaggacgcaagaaacccagcctccggtcacccacccgaccccactccacgctccaccacacattagtcatcaaacaaaacttaggacgaaaggtcaatcaccatgtccgcacaaagggagaggatcaatgccttctacaaagacaatcctcacaataagaaccatagggtgatcctggatagagaacgcttggtcattgaaagaccctacatcttgcttggagtcctgctggtaatgttcctgagtctaatcggactgctggccattgcagggatcaggcttcaccgggccaccgttggaacttcagagatccagagtcggctgaataccaatattaagttgaccgaatctattgatcaccagactaaggatgtcttaactcccctttttaaaatcattggcgatgaagtcggcatcagaattccacagaaattcagtgatcttgtcaagttcatctccgataagattaaattcctcaaccctgatagagagtatgatttcagagatctccggtggtgtatgaatccccccgagagagtcaaaattaattttgatcagttttgtgagtacaaggctgcggttaagtcaattgaacatatatttgagtcaccactcaacaagtcaaaaaagctgcaatctttgactctcgggcccggaacaggctgtctaggcaggacagtaacaagagcccatttctcagaacttacaatgaccttaatggacctggatctagagatgaagcacaacgtgtcctcagtgtttaccgtagttgaagagggattattcggaagaacatataccgtctggagatccgatgccagggatccgagcaccgatctaggtatcggccattttttaagagtcttcgagattggactggtaagagatcttgggctgggtccccctgtttttcatatgaccaactatctcacggtgaacatgagtgatgactatcggagatgtcttttagcggtaggggagttgaagttgacagccctatgcacctcatctgagactgtgacactgagtgagagaggagttccaaggagggaacctcttgtggttgtgatacttaatctagctggacccactctagggggagagctatacagtgtcttgcctacctctgatctcatggtggagaaactctatttgtcttcacatagagggatcatcaaagatgatgaggccaattgggtagtgccgtctaccgatgttcgtgatcttcagaacaaaggtgaatgtctggtggaagcatgcaagactcgacctccttcatttagcaatggcacaggatcaggcccgtggtcagaagggagaatccctgcctgcggggtgatcagggtcagtcttgacttagcgagtgacccggatgtagttatcacttcagtgtttggcccactgatacctcacctatccggcatggatctttacaacaacccgttttcaaaagctgtatggttggctgtaccaccttatgagcagtcatttctaggaatgataaatacaattggattccctaacagagcagaggttatgccgcacattttgaccacagagatcagaggccctcggggtcgttgccatgttcccatagaattgtcccgccgggttgatgacgatatcaagatcgggtcccacatggtcatattgccgacgatggacctgaggtatattacagccacttatgatgtttccaggagcgagcatgcaatcgtgtactatatctatgacacgagtcgctcatcatcttacttctacccagttcgactgaatttcaaaggcaatcctctctctttgaggatagagtgtttcccctggcgtcataaggtgtggtgctaccatgattgtcttatatacaacaccataacaggtgaagaggtccatacgagagggctgaccggcatagaggtaacatgcaatccagtctgagcagagctacgaccgtcactcaggcagtcccttgagtcgccaccgagtccaagcagcacagcctgggacaatcaacggcacagcccagccaacaatgttataaaaaacttaggagccagggttgtaggggccatggactctctatcagtcaatcaggtcttgtaccctgaggttcatctagacagccctattgtcacaaacaaactagttgccatccttgagtactcgggtatcgaccataactatgttcttgaagaccagactcttgtcaagaatattaggtataggctggggtgcggtttttcaaatcaaatgatcatcaataacaggggggtgggtgaaacagtcaattctaaacttaaaagttacccccataatcgtcatatcatatacccgggttgcaataaggagttgttttgtatcaaggatagctgcatatctaagaagctctcggagctattcaagaagggtaattccttgtactctaagataggtcaccaggtactggattgtcttaagagagtcaatgggaaattaggtctgggcacagatcttacccatggtctgaaggagggtatccttgacctagggttgcacatgcatagctctcaatggttcgagactttcctgttctggttcactatcaagacagagatgagatcaatgatcaaagaacagtcccatatatgccacaagaggaggtataacccgacttttgtgtcgggggatgcattcgaggtgctcgtatcgcgagacctcgttgtgataattgataagaatacccagtatgtcttctacttaacatttgagctggtccttatgtattgtgatgtcatagagggtagacttatgacggagacagccatggccatagaccagagatattcagaacttctaagccgggtcagatacttgtgggatcttattgatgggttcttcccaacactaggcaacaccacataccaaattgttgctctgcttgaaccattatcgttggcttatcttcaacttcaggatgtcactctggggttaagaggtgcttttttagaccattgcttcaaagaactctatgagatactggaacattgcggcattgacacagcaggcacttacaattccatcactgaaggattggattacgtattcatcacccacgatatacatctaactggggagattttttcatttttccggagtttcggacacccccgtctagaagcagtcaccgctgcggaaaatgtcagaaaacatatgaaccaaccgaaggtaatcagttatgagactatgatgaaaggccatgcggtattttgcgggataatcataaatggttttcgggaccgacatggcggcagctggccccctgttgcactgccagaacatgcctccgctgcgatccggaatgcgcaggcatcaggcgagggactgacccatgacctgtgtatagacaactggaaatcctttgttggattcaaatttggctgctttatgccactcagcctagatagtgatttgaccatgtatctcaaagacaaggcattggctgcactgaagaatgagtgggattcagtttacccgaaagaatacttccgttataacccacctagagggacagagtcaaggcgattggtagaggtattcctgaatgactccagctttgatccttataacatgataatgtacgtggtgaatggctcctaccttaaagaccctgagtttaacctctcatacagcctaaaagagaaggagataaaggagacagggcggttgtttgccaaaatggcctataagatgcgggcctgtcaggtaatcgctgaaaatctgatatcgaatggtgttgggaagtatttccgagacaatgggatggcaaaggacgagcatgacctaacaaaagcccttcacaccctggcagtctcaggtgttcccaaaaacaacaaagataaccaccgaggtgggcctcccagaaggacaacaaaccgaggggtgagatcaagccaaggcaccaaaacacaagatagagacaaggttcaagggggacctatgtacaactatttgcgatgccaaccgatcagccctgatcagggtgagtcatacgagactgttagtgcattcatcaccgctgaccttaagaagtattgcctgaattggagatacgagacaatcagcatatttgcacagaggctgaatgaaatatatggactaccatccttctttcaatggttacacaggatattggaaaaatccgtactctacgtcagtgacccacattgtcctcccgatctagataatcatatccctctggacagtgtccccaatgcccaaatattcatcaagtacccaatgggcggaatagaaggttattgccaaaaactatggacaatcagcactataccatacttgtatctggcagcctatgagagcggagtaagaatcgcctcactggtgcagggtgacaatcagacaatcgcagtgacaaaaagagttccaagttcttggccttattcactaaaaaagagggaggcctccaaggccgctcaaaattacttcgtagtcttaaggcaaagattgcacgatgtaggtcatcacttaaaggctaatgagaccatagtatcttctcacttttttgtatattccaaagggatttattatgacggcctgctagtctcacaatcactaaagagcatcgccagatgtgtcttctggtccgagactatagtggatgaaaccagagcggcgtgcagtaatattgcaacaactgtcgctaagagtatagagaggggttatgataggtaccttgcatattctttgaatatcctcaagattttccaacagatccttatatcccttaacttcactattaacacaacaatgactcaggatgtcgtggcaccgatcatcgagaacggtgatttgctgataaggatggcactcttgccagcacccatcgggggtctcaattatcttaacatgagcaggttatttgtgagaaatatcggtgacccggtcacctcctctatagccgacctgaagaggatgatagatgccgggctaatgccagaagaaacattgcatcaagtgatgacccagaccccgggagaatcatcctacctagattgggcaagtgacccttattctgccaacctaccctgcgtacagagtatcactcgccttctcaaaaacatcactgcacgatatatttcaatcagcagcccaaacccgatgctgaaagggttatttcatgaggggagtagggatgaagacgaggagcttgcaagtttcctaatggatcggcatataattgttccaagagctgcacatgaaatcttagaccatagtataaccggagcaagagaagctatagccgggatgttggacaccaccaagggtctgattagaacaagtatgaaacggggcggcctcacccctcgagtcttagcccgcctttccaattatgattatgaacaattccgatccgggataacactattaacaaagaaagggcagtgttatctcattgacaaggactcgtgctcggtgcagctcgctatagctctgaggggccacatgtgggctaagttggctcgcgggagaccaatctatgggttggaggtgcctgatgtattagaatcgatgatcggctaccttatcaaacgccacgagtcgtgtgctatctgtgaaacgggctcaagtcactacggatggtttttcgtccctgcagggtgccagcatgacgatgtatctagagagacttcggctcttcgtgtaccttatgtcggatcaacaactgaggaaaggacagacatgaaacttgcatttgttaggtctccaagtagatccctcaaatcagcagttagaattgcaacagtttactcatgggcttacggggatgatgggaaatcatggagtgaagcctggatgctagctaggcagagagccaatatcaccttagatgaattgagaatgatcactccagtctccacatccaccaacttagcccaccggttgagggatcggagtacccaggtgaaatactcggggacatcccttgtgagggttgcaagatacacaaccatctccaatgataacttgtcgtttgtgatatctgagaaaaaagtggataccaacttcatttatcagcaagggatgttgcttggtcttgggatccttgagaacctctttaggctagaggccaccaccggagtatccaacacagtgctacacctgcacgtggaaacagaatgttgtgttgtacctatggtggatcacccaaggataccgagtctccgtaatataaaagttacggatgagctatgcacaaaccctttgatctacgacaggtcccccatcatagagcacgatgcaacccggctatattcacagagccacaggagacatttggtggagtttgttacctggtcaacaagccagctttaccatatactggctaagtctacagcaatgtccatgattgaattgatcacgagattcgcgaaagatcacatgaatgaaatagccgccctgattggcgatgacgacatcaacagcttcatcacagaatttttgctagtggagcctagattatttatagtttaccttggtcagtgtgctgccatcaattgggcctttgatatacattatcatcggccctcgggcaagtaccagatgggggaacttctctactctctgctctctcggatgagcaaaggagtatataagatctttactaatgctttgagccaccccaaagtttacaagaaattttggcgaagtggcatcattgagccgattcatggcccatccttggatacacagaatttacatgtcactgtctgtgacatgatatatgggtcctatgtcacctatttggatcttttgctgaatgatgagctagatgattacccgtatttgctctgcgagagtgatgaggacgtggtcacagacaggttcgacaacattcaagccaaacatctctgtgtattggccgatgtatattgcagctccaagaggtgtccttcaataatcgggatgtctcctatagaaaaatgtaccattctcacacattacatcaagggagaatcagtacaatccccgtctgggacctcatggaacactgatccccttgtagtagatcattactcatgctctctgacctaccttcgccgcggttccatcaaacaaatcaggttgagggtggatcctgggtttgtattcgaggcgttgacagacgtcgactttaaacaacctcgcaaagctaagttagatatatcggtcgttgggttgactgatttctctcccccttacgacaacgtcggtgattttttagggactatcaacacattgaggcacgatctgcctgtcaccggaaccggggtctcgaactatgaagtccacgcttatcgtagaattggcctgaattcatcagcatgttataaagccgtagagatctccacgttaatcaagccatccttagaagtcggagagcatggattgttcttaggagaaggttccggttcaatgctggctgcgtacaaagaagttcttaaattagcaaattgttactacaacagcggagtaacagcggagggcagagccggacagagggaaatatctccctatccttcagagatgagcctagtagagaatcaaatggggatagagcggagtgttaaagtgctgttcaacgggaaacctgaagtaacttgggtggggaccacagattgctacaagtacataatcagtaacatccaaacctctagtctgggtttcatacattcagatattgagacgctcccaaccaaagatgctgttgagaaattggaggaatttgcctctatcctatccttatccttgattttggggaaaatcggctctattacagttgtcaaaattatgcccattagcggggattttacccaaggcttcatagcctatgccatccaatattttagggagagcctgcttgcctacccgagatatagtaacttcatctcgactgagtgttaccttattatgataggattaaaggccaatcgattgataaacccagaagccattaagcaaagcataatcagagcgggggtcaggactgcaccaggacttgtgtgccatgtattatcagagaaacaaaaaggttgtattcaatctctcttgggtgatccttatatcccaggagacttcaataagcaccttaaatctctaacctctattgagaaaatcctggtaaattgtggtctctcgatcaatggcataaaaatctgtagagatctaatccaccatgatatcgcctccggtccagacggccttatgagctctacaattattttatatagggaattggctcatttcaaagagaatataaggagtcagcacggaatgttccacccctacccggtactggccgacagcaggcaacgtgaattaatccttcgaatagccaagaaattctgggggtatgtcttgctatattctgatgacccggcactaatcagacaaacaatcaagaacttgaaacggaatcatctaacctttgacttacatagtaacccatttattaaagggctatccaaggctgagaaactgctggtgcggacgagttcactaagaagagaatggttgttcactctcgaaacgaaagaagtaaaagagtggttcaaattggtaggttacagtgcactcatcagaggctagtcagctatgcatctgcccccttctcctccgccatgagaccccactggcgatccagaagattaaagaaaactacatattggataagtatctattcccagctttgtctggt

>KM089831/China/Zhoukou/2014

accaaacaaagttgggtaaggatagatcttataataactatggactggcaaacttaggagtaaagatcctactgtcggggggaggaggaggagcaagatccttgatcatggcgactctccttaaaagcttagcattgttcaaaaggaacaaagacaaagcgccgacagcatcaggttcaggaggggccatccgggggattaagaatgttatcatagtcccgattcccggagactcgtccatcactacccgttcaagactgctcgacaggcttgtcagattggccggagatcctgacatcaacgggtcaaagctgaccggggtgatgatcagcatgttatccttgttcgtagagtcacccgggcaattgatacagcgaatcacagatgatccggatgttagtatccgccttgttgaggtagttcaaagtactaggtcccagtccgggttgacctttgcatcacgtggtgctgatttagacaacgaggcagacatgtatttttcaactgaggggccctcgagtggaggtaagaaaaggatcaactggtttgagaacagagaaataatagacatagaggtgcaggatccagaagagttcaatatgttgttagcctccatactagcacaagtctggatccttctggccaaggctgttacggcaccagatacggcagctgactcagaactgagaaggtgggttaaatacacacaacaaaggagagtgattggggaatttcgccttgacaaggggtggctggacgcagtccgcaacagaattgcagaagatctatcactccggcggttcatggtatctctcatacttgacatcaagaggaccccaggcaacaagccaaggattgcagaaatgatctgcgacattgacaactatattgtcgaagcaggactcaccagtttcatccttactatcaaatttggtatcgaaaccatgtatcctgcattagggctccacgagtttgccggagaattgtccactattgagtccttgatgaacttgtatcaacagctaggagaggttgcaccctacatggtaattctagagaattcaattcagaacaagtttagtgcaggagcttatcccctcctctggagctatgcgatgggtgtcggagtcgagctggagaactcaatggggggcttgaactttggcaggtcatattttgaccctgcctattttcgtctcggacaggagatggtcagaagatctgcaggaaaggtcagctctgtgattgcggctgagctcggcatcacagcagaggaagctaaactagtctcggaaatcgcctcgcaggctggggacgaaagaaccgccagagggactgggcctcgacaggcgcaggtttccttccttcagcaccaaacaggagggggagagtcgtccgcaccagcgaccagagaaggggtcaaagctgcgatcccaaacggagctgaagaaagggacagaaagcaaacacgcccaggaaggcccagaggagagacctccggccaactgctcctggacatcatgccagaggatgagatcttgcgagagtctggtcaaaaccctcgtgaggctcaaagatcggccgaggcactcttcaggctgcaggccatggccaagattctggaggaccaggaggagggagaagacaacagtcaggtctacaacgacaaggatctcctcggctaagcagacgcaccctctgtcgaaatcagtgacaagacatctcctaccagtattataaaaaacttaggacccaggtccaagcaagcacacatcgacactccaaccagtcgagcggagaccaccgatggcagaagaacaagcataccatgtcaacaaggggctggaatgtatcaagtctctcaaagcctcacccccggatctatccaccatcagagataccatcgagagctggagagaggggcttagcccctcgggccgtgcaacaccgaaccctgatacgtccgagggagatcatcagaatatcaaccaatcatgctcaccagcaatcggaccaaacaaagtctacttgtctcctgaagataatctcggatttagagagatcactggcaacgactgtgaggctgggctcggaggggtccagagagaaggatccaactctcaagtacagcgttaccatgtttatagtcacgggggtgaagagattgaaggactcgaggatgctgactctctcgtggttcaagcagatcctccatttgctaacgtcttcaatggaggagaggatggatctgacgacagcgatgtggactctggcccagatgatcccggcagaggtactctatatgaccggggatctgttgccggcaatggtatcgctgggtccacagatgtcgaaaaactagaaggtgctgatattcaagaagttcttaactcccagaaaggcaaaggaggaagattccagggcgggaaaaccttgcgagtcccggaaatacccgatgtcaagcactccagaccatcagcccaatcaattaaaaagggcacagaagggaactcagtctcatctggaacggtgatagagtgtttatcgataagtggtgcaacccaaactgtgccagagtcaagatgggagtcatcagagcaaaatgcgtctgtggggagtgtcctcaagtctgcgaggagtgcaaagacgatccaggggtcgacacaagaatctggtaccatagcatcactgactcagcctaaagagaatgactccgagtatgagtatgaggatgacctatttacagagattcaggacgtccgtgcaagcattgccaagatccatgatgacaataaaactatcctctcaaagcttgattctatactgttattgaaaggggaagtcgacactatcaagaaacaaatcagcaagcagaatataagtatatccaccattgagggccatctctccagtataatgatagccatccctggctttgggaaggacatcaaggacccaacatccgaagtcgagttgaacccagatctaagacctataataagccgtgactctggcagagctctcgcggaggtcctcaagaaacccgctgtggataggtctcagaaaattggaaccaaagccaactccagctcaaagggtcagcttcttaaggatctccagctaaaacctgtcgacaagcaggcaagctctgcaatcgggtttgtcccgtccgaccatgaatcatccagaaatgtcatccgctccataatcaagtcgagcaagctaaatattgatcacaaggactatcttctagatttactaaatgatgtgaaaggctccaaggatcttaaggaatttcacaagatgctaacagcaattctcgccaagcacccgtaacacatcctccagtcatcatctcatactcgactaaaaacatcctttcaatcaggctattacaaaaaacttaggagcaagggcaactgagcttcgcagacaggatgaccgagatctacgacttcgataaatcagcatgggatgtcaaagggtcaattgcccgcatagaacccaccacctaccacgacggccgactgataccccaggtgagggtcatcgatcctggtctgggagacagaaaagatgagtgctttatgtacctgtttctcctaggagtgattgaggataacgaccccctgtctccccccgtcggaagaacctttggctctctacctctaggggttggtaggtcaactgccaagccagaagaactactaagggaggccacagaattagatatagtggtgaggcgcactgcaggagtaaatgagaaactggtattttacaacaacactccgctgtccttgttaacgccctggaagaaagttctgacaaccggaagtgtgtttagcgctaaccaggtttgcaatgcagtcaacctagtcccgcttgatactccccagagattcagggttgtgtacatgagcataactagattatcagacaatggttactatagtgtgcccagaagaatgctggagttccgctcggccaatgcagtagccttcaacatcttggttacactaagaattgaaaatggcacaaaccctagaagatacatagtcggctcatgggagaattcagaggtcacatttatggtacacgtgggcaactttaggagaaagaagaacgaagtatactctgctgattactgcaaaatgaagattgaaaagatgggtttagtttttgccctgggtggaataggcggaacaagtctccatattagaagcaccgggaaaatgagcaaaaccctccatgcacagctggggttcaagaaaatcttatgttaccccctaatggatgtcaatgaggatcttaaccgatatctctggcgggcagagtgccgaatagtcaaaatccaagctgtcttacagccatcagtaccccaagagttccgtgtctacgatgatgtcatcatcaacgatgatcaaggcttgttcaagatcttgtagttcatttgcaacatcatgacgcgggtcgcaattttgacatttctgtttcttttcccaaatgttgttgcgtgtcagattcactggggcaatctatccaagatcgggattgtaggaacagggagtgccagctacaaggtgatgactaggccaagccaccagactctggttataaagttaatgccaaatataacggccatcgacaattgtacaaagtcagagattgcagagtacaagagattgctgatcacagtgttaaagcctgtagaggatgctctgtcggtgataaccaagaatgtaagaccaattcaaactctaacacctgggcgtagaacccgccgttttgctggagctgttctggccggggtagcacttggagttgcgacagccgctcagataactgcaggagtcgcccttcatcaatcattgatgaactcccaagcaattgagagtttaaaaaccagtcttgagatgtcgaatcaggcaatagaagaaatcagacttgcaaataaggagaccatactggcagtacagggcgtccaggattatatcaacaatgagctcgtcccttctgttcatagaatgtcatgcgagctggtaggtcacaagctcggcctcaagctccttaggtactacaccgagatcctgtccatattcgggcccagtcttcgagacccgatatctgccgaaatatcaatccaggcacttagttatgcattaggcggagacattaataaaatcctggacaagcttgggtatagcggtggggatttccttgccatcctagaaagcaagggaataaaggcccgggttacatatgtggacacaagagattactttataatccttagcatcgcctacccaaccttatctgagatcaagggagtgatagttcacaagatagaagctataacatacaacattggggcacaggagtggtatactactatccctaaatatgtagccactcaggggtatctgatatcgaactttgatgagacgtcatgcgtattcactccagaggggacagtttgcagccagaatgcgttgtacccaatgagcccattgcttcaggaatgtttcagggggtcaacaaaatcgtgcgccagaaccttagtttcagggaccataagtaatagatttatcctatcaaaagggaacctgattgcaaattgtgcgtcagttttgtgcaaatgttacacaacagaaacagttatcagccaagatcctgacaaactactaactgttgtagcatccgacaagtgtcctgtagttgaggtggatggagtgacaatacaggtcggcagtcgagagtatccggattctgtatacttacacaaaatagacttaggtccagccatctccctagaaaaactggatgtaggcaccaatttaggcaatgcagtcacaagactggagaatgcaaaggagctcctagatgcatcagaccaaatactgaagactgttaaaggggtacctttcagtgggaatatgtacatagcactggcagcttgcataggagtatccctgggccttgtcacattaatatgctgctgtaaggggaggtgtaagaacaaggaaatccctatctccaaaatcaacccagggctcaaacccgacctgaccgggacctcaaagtcgtacgtaagatcactgtagtcagaatcacctggatcatctggcatcacacacatacatgcacgacacaggcagtccgaggacgcaagaaacccagcctccggtcacccacccgaccccactccacgctccaccacacattagtcatcaaacaaaacttaggacgaaaggtcaatcaccatgtccgcacaaagggagaggatcaatgccttctacaaagacaatcctcacaataagaaccatagggtgatcctggatagagaacgcttggtcattgaaagaccctacatcttgcttggagtcctgctggtaatgttcctgagtctaatcggactgctggccattgcagggatcaggcttcaccgggccaccgttggaacttcagagatccagagtcggctgaataccaatattaagttgaccgaatctattgatcaccagactaaggatgtcttaactcccctttttaaaatcattggcgatgaagtcggcatcagaattccacagaaattcagtgatcttgtcaagttcatctccgataagattaaattcctcaaccctgatagagagtatgatttcagagatctccggtggtgtatgaatccccccgagagagtcaaaattaattttgatcagttttgtgagtacaaggctgcggttaagtcaattgaacatatatttgagtcaccactcaacaagtcaaaaaagctgcaatctttgactctcgggcccggaacaggctgtctaggcaggacagtaacaagagcccatttctcagaacttacaatgaccttaatggacctggatctagagatgaagcacaacgtgtcctcagtgtttaccgtagttgaagagggattattcggaagaacatataccgtctggagatccgatgccagggatccgagcaccgatctaggtatcggccattttttaagagtcttcgagattggactggtaagagatcttgggctgggtccccctgtttttcatatgaccaactatctcacggtgaacatgagtgatgactatcggagatgtcttttagcggtaggggagttgaagttgacagccctatgcacctcatctgagactgtgacactgagtgagagaggagttccaaggagggaacctcttgtggttgtgatacttaatctagctggacccactctagggggagagctatacagtgtcttgcctacctctgatctcatggtggagaaactctatttgtcttcacatagagggatcatcaaagatgatgaggccaattgggtagtgccgtctaccgatgttcgtgatcttcagaataaaggtgaatgtctggtggaagcatgcaagactcgacctccttcattttgcaatggcacaggatcaggcccgtggtcagaagggagaatccctgcctacggggtgatcagggtcagtcttgacttagcgagtgacccggatgtagttatcacttcagtgtttggcccactgatacctcacctatccgacatggatctttacaacaacccgttttcaaaagctgtatggttggctgtaccaccttatgagcagtcatttctaggaatgataaatacaattggattccctaatagagcagaggttatgccgcacattttgaccacagagatcagaggccctcggggtcgttgccatgttcccatagaattgtcccgccgggttgatgacgatatcaagatcgggtccaacatggtcatattgccgacgatggacctgaggtatattacagccacttatgatgtttccaggagcgagcatgcaatcgtgtactatatctatgacacgagtcgctcatcatcttacttctacccagttcgactgaatttcaaaggcaatcctctctctttgaggatagagtgtttcccctggcgtcgtaaggtgtggtgctaccatgattgtcttatatacaacaccataacaggtgaagaggtccatacgagagggctgaccggcatagaggtaacatgcaatccagtctgagcagagctacgaccgtcactcaggcagtcccttgagtcgccaccgagtccaagcagcacagcctgggacaatcaacggcacagcccagccaacaatgttataaaaaacttaggagccagggttgtaggggccatggactctctatcagtcaatcaggtcttgtaccctgaggttcatctagacagccctattgtcacaaacaaactagttgccatccttgagtactcgggtatcgaccataactatgttcttgaagaccagactcttgtcaagaatattaggtataggctggggtgcggtttttcaaatcaaatgatcatcaataacaggggggtgggtgaaacagtcaattctaaacttaaaagttacccccataatcgtcatatcatatacccggattgcaataaggagttgttttgtatcaaggatagctgcatatctaagaagctctcggagctattcaagaagggtaattccttgtactctaagataagtcaccaggtactggattgtcttaagagagtcaatgggaaattaggtctgggcacagatcttacccatggtctgaaggagggtatccttgacccagggttgcacatgcatagctctcaatggttcgagactttcctgttctggttcactatcaagacagagatgagatcaatgatcaaagaacagtcccatatatgccacaagaggaggtataacccgacttttgtgtcgggggatgcattcgaggtgctcgtatcgcgagacctcgttgtgataattgataagaatacccagtatgtcttctacttaacatttgagctggtccttatgtattgtgatgtcatagagggtagacttatgacggagacagccatggccatagaccagagatattcagaacttctaagccgggtcagatacttgtgggatcttattgatgggttcttcccaacactaggcaacaccacataccaaattgttgctctgcttgaaccattatcgttggcttatcttcaacttcaggatgtcactctggagttaagaggtgcttttttagaccattgcttcaaagaactctatgagatactggaacattgcggcattgacacagcaggcacttacaattccatcactgaaggattggattacgtattcatcacccacgatatacatctaactggggagattttttcatttttccggagtttcggacacccccgtctagaagcagtcaccgctgcggaaaatgtcagaaaacatatgaaccaaccgaaggtaatcagttatgagactatgatgaaaggccatgcggtattttgcgggataatcataaatggttttcgggaccgacatggcggcagctggccccctgttgcactgccagaacatgcctctgctgcgatccggaatgcgcaggcatcaggtgagggactgacccatgacctgtgtatagacaactggaaatcctttgttggattcaaatttggctgctttatgccactcagcctagatagtgatttgaccatgtatctcaaagacaaggcattggctgcactgaagaatgagtgggattcagtttacccgaaagaatacctccgttataacccacctagagggacagagtcaaggcgattggtagaggtattcctgaatgactccagctttgatccttataacatgataatgtacgtggtgaatggctcctaccttaaagaccctgagtttaacctctcatacagcctaaaagagaaggagataaaggagacagggcggttgtttgccaaaatgacctataagatgcgggcctgtcaggtaatcgctgaaaatctgatatcgaatggtgttgggaagtatttccgagacaatgggatggcaaaggacgagcatgacctaacaaaagcccttcacaccctggcagtctcaggtgttcccaaaaacaacaaagataaccaccgaggtgggcctcccagaaggacaacaaaccgaggggtgagatcaagccaaggcaccaaaacacaagatagagacaaggttcaagggggacctatgtacaactatttgcgatgccaaccgatcagccctgatcagggtgagtcatacgagactgttagtgcattcatcaccgctgaccttaagaagtattgcctgaattggagatacgagacaatcagcatatttgcacagaggctgaatgaaatatatggactaccatccttctttcaatggttacacaggatattggaaaaatccgtactctacgccagtgacccacattgtcctcccgatctagataatcatatccctctggacagtgtccccaatgcccaaatattcatcaagtacccaatgggcggaatagaaggttattgccaaaaactatggacaatcagcactataccatacttgtatctggcagcctatgagagcggagtaagaatcgcctcactggtgcagggtgacaatcagacaatcgcagtgacaaaaagagttccaagttcttggccttattcactaaaaaagagggaggcctccaaggccgctcaaaattacttcgtagtcttaaggcaaagattgcacgatgtaggtcatcacttaaaggctaatgagaccatagtatcctctcacttttttgtatattccaaagggatttattatgacggcctgctagtctcacaatcactaaagagcatcgccagatgtgtcttctggtccgagactatagtggatgaaaccagagcggcgtgcagtaatattgcaacaactgtcgctaagagtatagagaggggttatgataggtaccttgcatattctttgaatatcctcaagattttccaacagatccttatatcccttaacttcactattaacacaacaatgactcaggatgtcgtggcaccgatcatcgagaacggtgatttgctgataaggatggcactcttgccagcacccatcgggggtctcaattatcttaacatgagcaggttatttgtgagaaatatcggtgacccggtcacctcctctatagccgacctgaagaggatgatagatgccgggctaatgccagaagaaacattgcatcaagtgatgacccagaccccgggagaatcatcctacctagattgggcaagtgacccttactctgccaacctaccctgcgtacagagtatcactcgccttctcaaaaacatcactgcacgatatattttaatcagcagcccaaacccgatgctgaaagggttatttcatgaggggagtagagatgaagacgaggagcttgcaagtttcctaatggatcggcatataattgttccaagagctgcacatgaaatcttagaccatagtataaccggagcaagagaagctatagccgggatgttggacaccaccaagggtctgattagaacaagtatgaaacggggcggcctcacccctcgagtcttagcccgcctttccaattatgattatgaacaattccgatccgggataacactattaacaaaggaagggcagtgttatctcattgacaaggactcgtgctcggtgcagctcgctatagctctgaggggccacatgtgggctaagttggctcgcgggagaccaatctatgggttggaggtgcctgatgtattagaatcgatgaacggctaccttatcaaacgccacgagtcgtgtgctatctgtgaaacgggctcaagtcactacggatggtttttcgtccctgcagggtgccagcttgacgatgtatctagagagacttcggctcttcgtgtaccttatgtcggatcaacaactgaggaaaggacagacatgaaacttgcatttgttaggtctccaagtagatccctcaaatcagcagttagaattgcaacagtttactcatgggcttacggggatgatgagaaatcatggagtgaagcctggatgctagctaggcagagagccaatatcaccttagatgaattgagaatgatcactccagtctccacatccaccaacttagcccaccggttgagggatcggagtacccaggtgaaatactcggggacatcccttgtgagggttgcaagatacacaaccatctccaatgataacttgtcgtttgtgatatctgagaaaaaagtggataccaacttcatttatcagcaagggatgttgcttggtcttgggatccttgagaacctctttaggctagaggccaccaccggagtatccaacacagtgctacacctgcacgtggaaacagaatgttgtgttgtacctatggtggatcacccaaggataccgagtctccgtaatataaaagttacggatgagctatgcacaaaccctttgatctacgacaggtcccccatcatagagcacgatgcaacccggctatattcacagagccacaggagacatttggtggagtttgttacctggtcaacaagccagctttaccatatactggctaagtctacagcaatgtccatgattgaattgatcacgagattcgagaaagatcacatgaatgaaatagccgccctgattggcgatgacgacatcaacagcttcatcacagaatttttgctagtggagcctagattatttatagtttaccttggtcagtgtgctgccatcaattgggcctttgatatacattatcatcggccctcgggcaagtaccagatgggggaacttctctactctctgctctctcggatgagcaaaggagtatataagatctttactaatgctttgagccaccccaaagtttacaagaaattttggcgaagtggcatcattgagccgattcatggcccatccttggatacacagaatttacatgtcactgtctgtgacatgatatatgggtcctatgtcacctatttggatcttttgctgaatgatgagctagatgattacccgtatttgctctgcgagagtgatgaggacgtggtcacagacaggttcgacaacattcaagccaaacatctctgtgtattggccgatgtatattgcagctccaagaggtgtccttcaataatcgggatgtctcctatagaaaaatgtaccattctcacacattacatcaagggagaatcagtacaatccccgtctgggacctcatggaacactgatccccttgtagtagatcattactcatgctctctgatctaccttcgccgcggttccatcaaacaaatcaggttgagggtggatcctgggtttgtattcgaggcgttgacagacgtcgactttaaacaacctcgcaaagctaagttagatatatcggtcgttgggttgactgatttctctcccccttacgacaacgtcggtgattttttagggactatcaacacattgaggcacgatctgcctgtcaccggaaccggggtctcgaactatgaagtccacgcttatcgtagaattggcctgaattcatcagcatgttataaagccgtagagatctccacgttaatcaagccatccttagaagtcggagagcatggattgttcttaggagaaggttccggttcaatgctggctgcgtacaaagaagttcttaaattagcaaattgttactacaacagcggagtaacagcggagggcagagccggacagagggaaatatctccctatccttcagagatgagcctagtagagaatcaaatggggatagagcggagtgttaaagtgctgttcaacgggaaacctgaagtaacttgggtggggaccacagattgctacaagtacataatcagtaacatccaaacctctagtctgggtttcatacattcagatattgagacgctcccaaccaaagatgctgttgagaaattggaggaatttgcctctatcctatccttatccttgattttggggaaaatcggctctattacagttgtcaaaattatgcccattagcggggattttacccaaggcttcatagcctatgccatccaatattttagggagagcctgcttgcctatccgagatatagtaacttcatctcgactgagtgttaccttattatgataggattaaaggccaatcgattgataaacccagaagccattaagcaaagcataatcagagcgggggtcaggactgcaccaggacttgtgagccatgtattatcagagaaacaaaaaggttgtattcaatctttcttgggtgatccttatatccaaggagacttcaataagcaccttaaatctctaacctctattgagaaaatcctggtaaattgtggtctctcgatcaatggcataaaaatctgtagagatctaatccaccatgatatcgcctccggtccagatggccttatgagctctacaattattttatatagggaattggctcatttcaaagataatataaggagtcagcacggaatgttccacccctacccggtactggccaacagcaggcaacgtgaattaatccttcgaatagccaagaaattctgggggtatgtcttgctatattctgatgacccggcactaatcagacaaacaatcaagaacttgaaacggaatcatctaacctttgacttacatagtaacccatttattaaagggctatccaaggctgagaaactgctggtgcggacgagttcactaagaagagaatggttgttcactctcgaaacgaaagaagtaaaagagtggttcaaattggtaggttacagtgcactcatcagaggctagtcagctatgcatctgcccccttctcctccgccatgagaccccactggcgatccagaagattaaagaaaactacatattggataagtatctattcccagctttgtctggt

>KM089832/China/Zhumadian/2014

accaaacaaagttgggtaaggatagatcttataataactatggactggcaaacttaggagtaaagatcctactgtcggggggaggaggaggagcaagatctttgattatggcgactctccttaaaagcttagcattgttcaaaaggaacaaagacaaagcgccgacagcatcaggttcaggaggggccatccgggggattaagaatgttatcatagtcccgattcccggagactcgtccatcactacccgttcaagactgctcgacagacttgtcagattggccggagatcctgacatcaacgggtcaaagctgaccggggtgatgatcagcatgttatccttgttcgtagagtcacccgggcaattgatacagcgaatcacagatgatccggatgttagtatccgccttgttgaggtagttcaaagtactaggtcccagtccgggttgacctttgcatcacgtggtgctgatttagacaacgaggcagacatgtatttttcaactgaggggccctcgagtggaggtaagaaaaggatcaactggtttgagaacagagaaataatagacatagaggtgcaggatccagaagagttcaatatgttgttagcctccatactagcacaagtctggatccttctggccaaggctgttacggcaccagatacggcagctgactcagaactgagaaggtgggttaaatacacacaacaaaggagagtgattggggaatttcgccttgataaggggtggctggacgcagtccgcaacagaattgcagaagatctatcactccggcggttcatggtatctctcatacttgacatcaagaggaccccaggcaacaagccaaggattgcagaaatgatctgcgacattgacaactatattgtcgaagcaggactcgccagtttcatccttactatcaaatttggtatcgaaaccatgtatcctgcattagggctccacgagtttgccggagaattgtccactattgagtccttgatgaacttgtatcaacagctaggagaggttgcaccctacatggtaattctagagaattcaattcagaacaagtttagtgcaggagcttatcccctcctctggagctatgcgatgggtgtcggagtcgagctggagaactcaatggggggcttgaactttggcaggtcatattttgaccctgcctattttcgtctcggacaggagatggtcagaagatctgcaggaaaggtcagctctgtgattgcggctgagctcggcatcacagcagaggaagctaaactagtctcggaaatcgcctcgcaggctggggacgaaagaaccgccagagggactgggcctcgacaggcgcaggtttccttccttcagcaccaaacaggagggggagagtcgtccgcaccagcgaccagagaaggggtcaaagctgcgatcccaaacggagctgaagaaagggacagaaagcaaacacgcccaggaaggcccagaggagagacctccggccaactgctcctggacatcatgccagaggatgagatcttgcgagagtctggtcaaaaccctcgtgaggctcaaagatcggccgaggcactcttcaggctgcaggccatggccaagattctggaggaccaggaggagggagaagacaacagtcaggtctacaacgacaaggatctcctcggctaagcagacgcaccctctgtcgaaatcagtgacaagacatctcttaccagtattataaaaaacttaggacccaggtccaagcaagcacacatcgacactccaaccagtcgagcggagaccaccgatggcagaagaacaagcataccatgtcaacaaggggctggaatgtatcaagtctctcaaagcctcacccccggatctatccaccatcagagataccatcgagagctggagagaggggcttagcccctcgggccgtgcaacaccgaaccctgatacgtccgagggagatcatcagaatatcaaccaatcatgctcaccagcaatcggaccaaacaaagtctacttgtctcctgaagataatctcggatttagagagatcactggcaacgactgtgaggctgggctcggaggggtccagagagaaggatccaactctcaagtacagcgttaccatgtttatagtcacgggggtgaagagattgaaggactcgaggatgctgactctctcgtggttcaagcagatcctccagttgctaacgtcttcaatggaggagaggatggatctgacgacagcgatgtggactctggcccagatgatcccggcagagatactctatatgaccggggatctgttgccggcaatggtatcgctaggtccacagatgtcgaaaaactagaaggtgctgatattcaagaagttcttaactcccagaaaggcaaaggaggaagattccagggcgggaaaaccttgcgagtcccggaaatacccgatgtcaagcactccagaccatcagcccaatcaattaaaaagggcacagacgggaactcagtctcatctggaacggtgatagagtgtttatcgataagtggtgcaacccaaactgtgccagagtcaagatgggagtcatcagagcaaaatgtgtctgtggggagtgtcctcaagtctgcgaggagtgcaaagacgatccaggggtcgacacaagaatctggtaccatagcatcactgactcagcctaaagagaatgactccgagtatgagtatgaggatgacctatttacagagattcaggacatccgcgcaagcattgccaagatccatgatgacaataaaactatcctctcaaagcttgattctatactgttattgaaaggggaagtcgacactatcaagaaacaaatcagcaagcagaatataagtatatccaccattgagggccatctctccagtataatgatagccatccctggctttgggaaggacatcaaggacccaacatccgaagtcgagttgaacccagatctaagacctataataagccgtgactctggcagagctctcgcggaggtcctcaagaaacccgctgtggataggtctcagaaaattggaaccaaagccaactccagctcaaagggtcagcttcttaaggatctccagctaaaacctgtcgacaagcaggcaagctctgcaatcgggtttgtcccgtccgaccatgaatcatccagaaatgtcatccgctccataatcaagtcgagcaagctaaatattgatcacaaggactatcttctagatttactaaatgatgtgaaaggctccaaggatcttaaggaatttcacaagatgctaacagcaattctcgccaagcacccgtaacacatcctccagtcatcatctcatactcgactaaaaacatcctttcaatcaggctattacaaaaaacttaggagcaagggcaactgagcttcgcagacaggatgaccgagatctacgacttcgataaatcagcatgggatgtcaaagggtcaattgcccgcatagaacccaccacctaccacgacggccgactgataccccaggtgagggtcatcgatcctggtctgggagacagaaaagatgagtgctttatgtacctgtttctcctaggagtgattgaggataacgaccccctgtctccccccgtcggaagaacctttggctctctacctctaggggttggtaggtcaactgccaagccggaagaactactaagggaggccacagaattagatatagtggtgaggcgcactgcaggagtaaatgagaaactggtattctacaacaacactccgctgtccttgttaacgccctggaagaaagttctgacaaccggaagtgtgtttagcgctaaccaggtttgcaatgcagtcaacctagtcccgcttgatactccccagagattcagggttgtgtacatgagcataactagattatcagacaatggttactatagtgtgcccagaagaatgctggagttccgctcggccaatgcagtagccttcaacatcttggttacactaagaattgaaaatggcacaaaccctagaagatacatagtcggctcatgggagaattcagaggtcacatttatggtacacgtgggcaactttaggagaaagaagaacgaagtatactctgctgattactgcaaaatgaagattgaaaagatgggtttagtttttgccctgggtggaataggcggaacaagtctccatattagaagcaccgggaaaatgagcaaaaccctccatgcacagctggggttcaagaaaatcttatgttaccccctaatggatgtcaatgaggatcttaaccgatatctctggcgggcagagtgccgaatagtcaaaatccaagctgtcttacagccatcagtaccccaagagttccgtgtctacgatgatgtcatcatcaacgatgatcaaggcttgttcaagatcttgtagttcatttgcaacatcatgacgcgggtcgcaattttgacatttctgtttcttttcccaaatgttgttgcgtgtcagattcactggggcaatctatccaagatcgggattgtaggaacagggagtgccagctacaaggtgatgactaggccaagccaccagactctggttataaagttaatgccaaatataacggccatcgacaattgtacaaagtcagagattgcagagtacaagagattgctgatcacagtgttaaagcctgtagaggatgctctgtcggtgataaccaagaatgtaagaccaattcaaactctaacacctgggcgtagaacccgccgttttgctggagctgttctggccggggtagcacttggagttgcgacagccgctcagataactgcaggggtcgcccttcatcaatcattgatgaactcccaagcaattgagagtttaaaaaccagtcttgagatgtcgaatcaggcaatagaagaaatcagacttgcaaataaggagaccatactggcagtacagggcgtccaggattatatcaacaatgagctcgtcccttctgttcataggatgtcatgcgagctggtaggtcacaagctcggcctcaagctccttaggtactacaccgagatcctgtccatattcgggcccagtcttcgagacccgatatctgccgaaatatcaatccaggcacttagttatgcattaggcggagacattaataaaatcctggacaagcttgggtatagcggtggggatttccttgccatcctagaaagcaagggaataaaggcccgggttacatatgtggacacaagagattactttataatccttagcatcgcctacccaaccttatctgagatcaagggagtgatagttcacaagatagaagctataacatacaacattggggcacaggagtggtatactactatccctaaatatgtagccactcaggggtatctgatatcgaactttgatgagacgtcatgcgtattcactccagaggggacagtttgcagccagaatgcgttgtacccaatgagcccattgcttcaggaatgtttcagggggtcaacaaaatcgtgcgccagaaccttagtttcagggaccataagtaatagatttatcctatcaaaagggaacctgattgcaaattgtgcgtcagttttgtgcaaatgttacacaacagagacagttatcagccaagatcctgacaaactactaactgttgtagcacccgacaagtgtcctgtagttgaggtggatggagtgacaatacaggtcggcagtcgagagtatccggattctgtatacttacacaaaatagacttaggtccagccatctccctagaaaaactggatgtaggcaccaatttaggcaatgcagtcacaagactggagaatgcaaaggagctcctagatgcatcagaccaaatactgaagactgttaaaggggtacctttcagtgggaatatgtacatagcactggcagcttgcataggagtatccttgggccttgtcacattaatatgctgctgtaaggggaggtgtaagaacaaggaaatccctatctccaaaatcaacccagggctcaaacccgacctgaccgggacctcaaagtcgtacgtaagatcactgtagtcagaatcacctggatcatctggcatcgcacacatacatgcacgacacaggcagtccgaggacgcaagaaacccagcctccggtcacccacccgaccccactccacgctccaccacacattagtcatcaaacaaaacttaggacgaaaggtcaatcaccatgtccgcacaaagggagaggatcaatgccttctacaaagacaatcctcacaataagaaccatagggtgatcctggatagggaacgcttggtcattgaaagaccctacatcttgcttggagtcctgctggtaatgttcctgagtctaatcggactgctggccattgcagggatcaggcttcaccgggccaccgttggaacttcagagatccagagtcggctgaataccaatattaagttgaccgaatctattgatcaccagactaaggatgtcttaactcccctttttaaaatcattggcgatgaagtcggcatcagaattccacagaaattcagtgatcttgtcaagttcatctccgataagattaaattcctcaaccctgatagagagtatgatttcagagatctccggtggtgtatgaatccccccgagagagtcaaaattaattttgatcagttttgtgagtacaaggctgcggttaagtcaattgaacatatatttgagtcaccactcaacaagtcaaaaaagctgcaatctttgactctcgggcccggaacaggctgtctaggcaggacagtaacaagagcccatttctcagaacttacaatgaccttaatggacctggatctagagatgaagcacaacgtgtcctcagtgtttaccgtagttgaagagggattattcggaagaacatataccgtctggagatccgatgccagggatccgagcaccgatctaggtatcggccattttttaagagtcttcgagattggactggtaagagatcttgggctgggtccccctgtttttcatatgaccaactatctcacggtgaacatgagtgatgactatcggagatgtcttttagcggtaggggagctgaagttgacagccctatgcacctcatctgagactgtgacactgagtgagagaggagttccaaggagggaacctcttgtggttgtgatacttaatctagctggacccactctagggggagagctatacagtgtcttgcctacctctgatctcatggtggagaaactctatttgtcttcacatagagggatcatcaaagatgatgaggccaattgggtagtgccgtctaccgatgttcgtgatcttcagaacaaaggtgaatgtctggtggaagcatgcaagactcgacctccttcattttgcaatggcacaggatcaggcccgtggtcagaagggagaatccctgcctacggggtgatcagggtcagtcttgacttagcgagtgacccggatgtagttatcacttcagtgtttggcccactgatacctcacctatccggcatggatctttacaacaacccgttttcaaaagctgtatggttggctgtaccaccctatgagcagtcatttctaggaatgataaatacaattggattccctaacagagcagaggttatgccgcacattttgaccacagagatcagaggccctcggggtcgttgccatgttcccatagaattgtcccgccgggttgatgacgatatcaagatcgggtccaacatggtcatattgccgacgatggacctgaggtatattacagccacttatgatgtttccaggagcgagcatgcaatcgtgtactatatctatgacacgagtcgctcatcatcttacttctacccagttcgactgaatttcaaaggcaatcctctctctttgaggatagagtgtttcccctggcgtcataaggtgtggtgctaccatgattgtcttatatacaacaccataacaggtgaagaggtccatacgagagggctgaccggcatagaggtaacatgcaatccagtctgagcagagctacgaccgtcactcaggcagtcccttgagtcgccaccgagtccaagcagcacagcctgggacaatcaacggcacagcccagccaacaatgttataaaaaacttaggagccagggttgtaggggccatggactctctatcagtcaatcaggtcttgtaccctgaggttcatctagacagccctattgtcacaaacaaactagttgccatccttgagtactcgggtatcgaccataactatgttcttgaagaccagactcttgtcaagaatattaggtataggctggggtgcggtttttcaaatcaaatgatcatcaataacaggggggtgggtgaaacagtcaattctaaacttaaaagttacccccataatcgtcatatcatatacccggattgcaataaggagttgttttgtatcaaggatagctgcatatctaagaagctctcggagctattcaagaagggtaattccttgtactctaagataagtcaccaggtactggattgtcttaagagagtcaatgggaaattaggtctgggcacagatcttacccatggtctgaaggagggtatccttgacctagggttgcacatgcatagctctcaatggttcgagactttcctgttctggttcactatcaagacagagatgagatcaatgatcaaagaacagtcccatatatgccacaagaggaggtataacccgacttttgtgtcgggggatgcattcgaggtgctcgtatcgcgagacctcgttgtgataattgataagaatacccagtatgtcttctacttaacatttgagctggtccttatgtattgtgatgtcatagagggtagacttatgacggagacagccatggccatagaccagagatattcagaacttctaagccgggtcagatacttgtgggatcttattgatgggttcttcccaacactaggcaacaccacataccaaattgttgctctgcttgaaccattatcgttggcttatcttcaacttcaggatgtcactctggagttaagaggtgcttttttagaccattgcttcaaagaactctatgagatactggaacattgcggcattgacacagcaggcacttacaattccatcactgaaggattggattacgtattcatcacccacgatatacatctaactggggagattttttcatttttccggagtttcggacacccccgtctagaagcagtcaccgctgcggaaaatgtcagaaaacatatgaaccaaccgaaggtaatcagttatgagactatgatgaaaggccatgcggtattttgcgggataatcataaatggttttcgggaccgacatggcggcagctggccctctgttgcactgccagaacatgcctctgctgcgatccggaatgcgcaggcatcaggtgagggactgacccatgacctgtgtatagacaactggaaatcctttgttggattcaaatttggctgctttatgccactcagcctagatagtgatttgaccatgtatctcaaagacaaggcattggctgcactgaagaatgagtgggattcagtttacccgaaagaatacctccgttataacccacctagagggacagagtcaaggcgattggtagaggtattcctgaatgactccagctttgatccttataacatgataatgtacgtggtgaatggctcctaccttaaagaccctgagtttaacctctcatacagcctaaaagagaaggagataaaggagacagggcggttgtttgccaaaatgacctataagatgcgggcctgtcaggtaatcgctgaaaatctgatatcgaatggtgttgggaagtatttccgggacaatgggatggcaaaggacgagcatgacctaacaaaagcccttcacaccctggcagtctcaggtgttcccaaaaacaacaaagataaccaccgaggtgggcctcccagaaggacaacaaaccgaggggtgagatcaagccaaggcaccaaaacacaagatagagacaaggttcaagggggacctatgtacaactatttgcgatgccaaccgatcagccttgatcagggtgagtcatacgagactgttagtgcattcatcaccgctgaccttaagaagtattgcctgaattggagatacgagacaatcagcatatttgcacagaggctgaatgaaatatatggactaccatccttctttcaatggttacacaggatattggagaaatccgtactctacgtcagtgacccacattgtcctcccgatctagataatcatatccctctggacagtgtccccaatgcccaaatattcatcaagtacccaatgggcggaatagaaggttattgccaaaaactatggacaatcagcactataccatacttgtatctggcagcctatgagagcggagtaagaatcgcctcactggtgcagggtgacaatcagacaatcgcagtgacaaaaagagttccaagttcttggccttattcactaaaaaagagggaggcctccaaggccgctcaaaattacttcgtagtcttaaggcaaagattgcacgatgtaggtcatcacttaaaggctaatgagaccatagtatctcctcacttttttgtatattccaaagggatttattatgacggcctgctagtctcacaatcactaaagagcatcgccagatgtgtcttctggtccgagactatagtggatgaaaccagagcggcgtgcagtaatattgcaacaactgtcgctaagagtatagagaggggttatgataggtaccttgcatattctttgaatatcctcaagattttccaacagatccttatatccctcaacttcactattaacacaacaatgactcaggatgtcgtggcaccgatcatcgagaacggtgatttgctgataaggatggcactcttgccagcacccatcgggggtctcaattatcttaacatgagcaggttatttgtgagaaatatcggtgacccggtcacctcctctatagccgacctgaagaggatgatagatgccgggctaatgccagaagaaacattgcatcaagtgatgacccagaccccgggagaatcatcctacctagattgggcaagtgacccttattctgccaacctaccctgcgtacagagtatcactcgccttctcaaaaacatcactgcacgatatattttaatcagcagcccaaacccgatgctgaaagggttatttcatgaggggagtagagatgaagacgaggagcttgcaagtttcctaatggatcggcatataattgttccaagagctgcacatgaaatcttagaccatagtataaccggagcaagagaagctatagccgggatgttggacaccaccaagggtctgattagaacaagtatgaaacggggcggcctcacccctcgagtcttagcccgcctttccaattatgattatgaacaattccgatccgggataacactattaacaaagaaagggcagtgttatctcattgacaaggactcatgctcggtgcagctcgctatagctctgaggggccacatgtgggctaagttggctcgcgggagaccaatctatgggttggaggtgcctgatgtattagaatcgatgaacggctaccttatcaaacgccacgagtcgtgtgctatctgtgaaacgggctcaagtcactacggatggtttttcgtccctgcagggtgccagcttgacgatgtatctagagagacttcggctcttcgtgtaccttatgtcggatcaacaactgaggaaaggacagacatgaaacttgcatttgttaggtctccaagtagatccctcaaatcagcagttagaattgcaacagtttactcatgggcttacggggatgatgagaaatcatggagtgaagcctggatgctagctaggcagagagccaatatcaccttagatgaattgagaatgatcactccagtctccacatccaccaacttagcccaccggttgagggatcggagtacccaggtgaaatactcggggacatcccttgtgagggttgcaagatacacaaccatctccaatgataacttgtcgtttgtgatatctgagaaaaaagtggataccaacttcatttatcagcaagggatgttgcttggtcttgggatccttgagaacctctttaggctagaggccaccaccggagtatccaacacagtgctacacctgcacgtggaaacagaatgttgtgttgtacctatggtggatcacccaaggataccgagtctccgtaatataaaagttacggatgagctatgcacaaaccctttgatctacgacaggtcccccatcatagagcacgatgcaacccggctatattcacagagccacaggagacatttggtggagtttgttacctggtcaacaagccagctttaccatatactggctaagtctacagcaatgtccatgattgaattgatcacgggattcgagaaagatcacatgaatgaaatagccgccctgattggcgatgacgacatcaacagcttcatcacagaatttttgctagtggagcctagattatttatagtttaccttggtcagtgtgctgccatcaattgggcctttgatatacattatcatcggccctcgggcaagtaccagatgggggaacttctctactctctgctctctcggatgagcaaaggagtatataagatctttactaatgctttgagccaccccaaagtttacaagaaattttggcgaagtggcatcattgagccgattcatggcccatccttggatacacagaatttacatgtcactgtctgtgacatgatatatgggtcctatgtcacctatttggatcttttgctgaatgatgagctagatgattacccgtatttgctctgcgagagtgatgaggacgtggtcacagacaggttcgacaacattcaagccaaacatctctgtgtattggccgatgtatattgcagcttcaagaggtgtccttcaataatcgggatgtctcctatagaaaaatgtaccattctcacacattacatcaagggagaatcagtacaatccccgtctgggacctcatggaacactgatccccttgtagtagatcattactcatgctctctgacctaccttcgccgcggttccatcaaacaaatcaggttgagggtggatcctgggtttgtactcgaggcgttgacagacgtcgactttaaacaacctcgcaaagctaagttagatatatcggtcgttgggtcgactgatttctctcccccttacgacaacgtcggtgattttttagggactatcaacacattgaggcacgatctgcctgtcaccggaaccggggtctcgaactatgaagtccacgcttatcgtagaattggcctgaattcatcagcatgttataaagccgtagagatctccacgttaatcaagccatccttagaagtcggagagcatggattgttcttaggagaaggttccggttcaatgctggctgcgtacaaagaagttcttaaattagcaaattgttactacaacagcggagtaacagcggagggcagagccggacagagggaaatatctccctatccttcagagatgagcctagtagagaatcaaatggggatagagcggagtgttaaagtgctgttcaacgggaaacctgaagtaacttgggtggggaccacagattgctacaagtacataatcagtaacatccaaacctctagtctgggtttcatacattcagatattgagacgctcccaaccaaagatgctgttgagaaattggaggaatttgcctctatcctatccttatccttgattttggggaaaatcggctctattacagttgtcaaaattatgcccattagcggggattttacccaaggcttcatagcctatgccatccaatattttagggagagcctgcttgcctacccgagatatagtaacttcatctcgactgagtgttaccttattatgataggattaaaggccaatcgattgataaacccagaagccattaagcaaagcataatcagagcgggggtcaggactgcaccaggacttgtgagccatgtattatcagagaaacaaaaaggttgtattcaatctttcttgggtgatccttatatccaaggagacttcaataagcaccttaaatctctaacctctattgagaaaatcctggtaaattgtggtctctcgatcaatggcataaaaatctgtagagatctaatccaccatgatatcgcctccggtccagacggccttatgagctctacaattattttatatagggaattggctcatttcaaagataatataaggagtcagcacggaatgttccacccctacccggtactggccaacagcaggcaacgtgaattaatccttcgaatagccaagaaattctgggggtatgtcttgctatattctgatgacccggcactaatcagacaaacaatcaagaacttgaaacggaatcatctaacctttgacttacatagtaacccatttattaaagggctatccaaggctgagaaactgctggtgcggacgagttcactaagaagagaatggttgttcactctcgaaacgaaagaagtaaaagagtggttcaaattggtaggttacagtgcactcatcagaggctagtcagctatgcatctgcccccttctcctccgccatgagaccccactggcgatccagaagattaaagaaaactacatattggataagtatctattcccagctttgtctggt

>KM091959/China/Ili/2013

accaaacaaagttgggtaaggatcgatcttataataactatggactggcaaacttaggagtaaagatcctactgtcggggggaggaggaggagcaagatctttgattatggcgactctccttaaaagcttagcattgttcaaaaggaacaaagacaaagcgccgacagcatcaggttcaggaggggccatccgggggattaagaatgttatcatagtcccgattcccggagactcgtccatcactacccgttcaagactgctcgacaggcttgtcagattggccggagatcctgacatcaacgggtcaaagctgaccggggtgatgatcagcatgttatccttgttcgtagagtcacccgggcaattgatacagcgaatcacagatgatccggatgttagtatccgccttgttgaggtagttcaaagtactaggtcccagtccgggttgacctttgcatcacgtggtgctgatttagacaacgaggcagacatgtatttttcaactgaggggccctcgagtggaggtaagaaaaggatcaactggtttgagaacagagaaataatagacatagaggtgcaggatccagaagagttcaatatgttgttagcctccatactagcacaagtctggatccttctggccaaggctgttacggcaccagatacggcagctgactcagaactgagaaggtgggttaaatacacacaacaaaggagagtgattggggaatttcgccttgacaaggggtggctggacgcagtccgcaacagaattgcagaagatctatcactccggcggttcatggtatctctcatacttgacatcaagaggaccccaggcaacaagccaaggattgcagaaatgatctgcgacattgacaactatattgtcgaagcaggactcgccagtttcatccttactatcaaatttggtatcgaaaccatgtatcctgcattagggctccacgagtttgccggagaattgtccactattgagtccttgatgaacttgtatcaacagctaggagaggttgcaccctacatggtaattctagagaattcaattcagaacaagtttagtgcaggagcttatcccctcctctggagctatgcgatgggtgtcggagtcgagctggagaactcaatggggggcttgaactttggcaggtcatattttgaccctgcctattttcgtctcggacaggagatggtcagaagatctgcaggaaaggtcagctctgtgattgcggctgagctcggcatcacagcagaggaagctaaactagtctcggaaatcgcctcgcaggctggggacgaaagaaccgccagagggactgggcctcgacaggcgcaggtttccttccttcagcaccaaacaggagggggagagtcgtccgcaccagcgaccagagaaggggtcaaagctgcgatcccaaacggagctgaagaaagggacagaaagcaaacacgcccaggaaggcccagaggagagacctccggccaactgctcctggacatcatgccagaggatgagatcttgcgagagtctggtcaaaaccctcgtgaggctcaaagatcggccgaggcactcttcaggctgcaggccatggccaagattctggaggaccaggaggagggagaagacaacagtcaggtctacaacgacaaggatctcctcggctaagcagacgcaccctctgtcgaaatcagtgacaagacatctcctaccagtattataaaaaacttaggacccaggtccaagcaagcacacatcgacactccaaccagtcgagcggagaccaccgatggcagaagaacaagcataccatgtcaacaaggggctggaatgtatcaagtctctcaaagcctcacccccggatctatccaccatcagagataccatcgagagctggagagaggggcttagcccctcgggccgtgcaacaccgaaccctgatacgtccgagggagatcatcagaatatcaaccaatcatgctcaccagcaatcggaccaaacaaagtctacttgtctcctgaagataatctcggatttagagagatcactggcaacgactgtgaggctgggctcggaggggtccagagagaaggatccaactctcaagtacagcgttaccatgtttatagtcacgggggtgaagagattgaaggactcgaggatgctgactctctcgtggttcaagcagatcctccagttgctaacgtcttcaatggaggagaggatggatctgacgacagcgatgtggactctggcccagatgatcccggcagagatactctatatgaccggggatctgttgccggcaatggtatcgctaggtccacagatgtcgaaaaactagaaggtgctgatattcaagaagttcttaactcccagaaaggcaaaggaggaagattccagggcgggaaaaccttgcgagtcccggaaatacccgatgtcaagcactccagaccatcagcccaatcaattaaaaagggcacagacgggaactcagtctcatctggaacggtgatagagtgtttatcgataagtggtgcaacccaaactgtgccagagtcaagatgggagtcatcagagcaaaatgcgtctgtggggagtgtcctcaagtctgcgaggagtgcaaagacgatccaggggtcgacacaagaatctggtaccatagcatcactgactcagcctaaagagaatgactccgagtatgagtatgaggatgacctatttacagagattcaggacatccgtgcaagcattgccaagatccatgatgacaataaaactatcctctcaaagcttgattctatactgttattgaaaggggaagtcgacactatcaagaaacaaatcagcaagcagaatataagtatatccaccattgagggccatctctccagtataatgatagccatccctggctttgggaaggacatcaaggacccaacatccgaagtcgagttgaacccagatctaagacctataataagccgtgactctggcagagctctcgcggaggtcctcaagaaacccgctgtggataggtctcagaaaattggaaccaaagccaactccagctcaaagggtcagcttcttaaggatctccagctaaaacctgtcgacaagcaggcaagctctgcaatcgggtttgtcccgtccgaccatgaatcatccagaaatgtcatccgctccataatcaagtcgagcaagctaaatattgatcacaaggactatcttctagatttactaaatgatgtgaaaggctccaaggatcttaaggaatttcacaagatgctaacagcaattctcgccaagcacccgtaacacatcctccagtcatcatctcatactcgactaaaaacatcctttcaatcaggctattacaaaaaacttaggagcaagggcaactgagcttcgcagacaggatgaccgagatctacgacttcgataaatcagcatgggatgtcaaagggtcaattgcccgcatagaacccaccacctaccacgacggccgactgataccccaggtgagggtcatcgatcctggtctgggagacagaaaagatgagtgctttatgtacctgtttctcctaggagtgattgaggataacgaccccctgtctccccccgtcggaagaacctttggctctctacctctaggggttggtaggtcaactgccaagccagaagaactactaagggaggccacagaattagatatagtggtgaggcgcactgcaggagtaaatgagaaactggtattttacaacaacactccgctgtccttgttaacgccctggaagaaagttctgacaaccggaagtgtgtttagcgctaaccaggtttgcaatgcagtcaacctagtcccgcttgatactccccagagattcagggttgtgtacatgagcataactagattatcagacaatggttactatagtgtgcccagaagaatgctggagttccgctcggccaatgcagtagccttcaacatcttggttacactaagaattgaaaatggcacaaaccctagaagatacatagtcggctcatgggagaattcagaggtcacatttatggtacacgtgggcaactttaggagaaagaagaacgaagtatactctgctgattactgcaaaatgaagattgaaaagatgggtttagtttttgccctgggtggaataggcggaacaagtctccatattagaagcaccgggaaaatgagcaaaaccctccatgcacagctggggttcaagaaaatcttatgttaccccctaatggatgtcaatgaggatcttaaccgatatctctggcgggcagagtgccgaatagtcaaaatccaagctgtcttacagccatcagtaccccaagagttccgtgtctacgatgatgtcatcatcaacgatgatcaaggcttgttcaagatcttgtagttcatttgcaacatcatgacgcgggtcgcaattttgacatttctgtttcttttcccaaatgttgttgcgtgtcagattcactggggcaatctatccaagatcgggattgtaggaacagggagtgccagctacaaggtgatgactaggccaagccaccagactctggttataaagttaatgccaaatataacggccatcgacaattgtacaaagtcagagattgcagagtacaagagattgctgatcacagtgttaaagcctgtagaggatgctctgtcggtgataaccaagaatgtaagaccaattcaaactctaacacctgggcgtagaacccgccgttttgctggagctgttctggccggggtagcacttggagttgcgacagccgctcagataactgcaggagtcgcccttcatcaatcattgatgaactcccaagcaattgagagtttaaaaaccagtcttgagatgtcgaatcaggcaatagaagaaatcagacttgcaaataaggagaccatactggcagtacagggcgtccaggattatatcaacaatgagctcgtcccttctgttcatagaatgtcatgcgagctggtaggtcacaagctcggcctcaagctccttaggtactacaccgagatcctgtccatattcgggcccagtcttcgagacccgatatctgccgaaatatcaatccaggcacttagttatgcattaggcggagacattaataaaatcctggacaagcttgggtatagcggtggggatttccttgccatcctagaaagcaagggaataaaggcccgggttacatatgtggacacaagagattactttataatccttagcatcgcctacccaaccttatctgagatcaagggagtgatagttcacaagatagaagctataacatacaacattggggcacaggagtggtatactactatccctaaatatgtagccactcaggggtatctgatatcgaactttgatgagacgtcatgcgtattcactccagaggggacagtttgcagccagaatgcgttgtacccaatgagcccattgcttcaggaatgtttcagggggtcaacaaaatcgtgcgccagaaccttagtttcagggaccataagtaatagatttatcctatcaaaagggaacctgattgcaaattgtgcgtcagttttgtgcaaatgttacacaacagagacagttatcagccaagatcctgacaaactactaactgttgtagcatccgacaagtgtcctgtagttgaggtggatggagtgacaatacaggtcggcagtcgagagtatccggattctgtatacttacacaaaatagacttaggtccagccatctccctagaaaaactggatgtaggcaccaatttaggcaatgcagtcacaagactggagaatgcaaaggagctcctagatgcatcagaccaaatactgaagactgttaaaggggtacctttcagtgggaatatgtacatagcactggcagcttgcataggagtatccctgggccttgtcacattaatatgctgctgtaaggggaggtgtaagaacaaggaaatccctatctccaaaatcaacccagggctcaaacccgacctgaccgggacctcaaagtcgtacgtaagatcactgtagtcagaatcacctggatcatctggcatcacacacatacatgcacgacacaggcagtccgaggacgcaagaaacccagcctccggtcacccacccgaccccactccacgctccaccacacattagtcatcaaacaaaacttaggacgaaaggtcaatcaccatgtccgcacaaagggagaggatcaatgccttctacaaagacaatcctcacaataagaaccatagggtgatcctggatagagaacgcttggtcattgaaagaccctacatcttgcttggagtcctgctggtaatgttcctgagtctaatcggactgctggccattgcagggatcaggcttcaccgggccaccgttggaacttcagagatccagagtcggctgaataccaatattaagttgaccgaatctattgatcaccagactaaggatgtcttaactcccctttttaaaatcattggcgatgaagtcggcatcagaattccacagaaattcagtgatcttgtcaagttcatctccgataagattaaattcctcaaccctgatagagagtatgatttcagagatctccggtggtgtatgaatccccccgagagagtcaaaattaattttgatcagttttgtgagtacaaggctgcggttaagtcaattgaacatatatttgagtcaccactcaacaagtcaaaaaagctgcaatctttgactctcgggcccggaacaggctgtctaggcaggacagtaacaagagcccatttctcagaacttacaatgaccttaatggacctggatctagagatgaagcacaacgtgtcctcagtgtttaccgtagttgaagagggattattcggaagaacatataccgtctggagatccgatgccagggatccgagcaccgatctaggtatcggccattttttaagagtcttcgagattggactggtaagagatcttgggctgggtccccctgtttttcatatgaccaactatctcacggtgaacatgagtgatgactatcggagatgtcttttagcggtaggggagttgaagttgacagccctatgcacctcatctgagactgtgacactgagtgagagaggagttccaaggagggaacctcttgtggttgtgatacttaatctagctggacccactctagggggagagctatacagtgtcttgcctacctctgatctcatggtggagaaactctatttgtcttcacatagagggatcatcaaagatgatgaggccaattgggtagtgccgtctaccgatgttcgtgatcttcagaacaaaggtgaatgtctggtggaagcatgcaagactcgacctccttcattttgcaatggcacaggatcaggcccgtggtcagaagggagaatccctgcctacggggtgatcagggtcagtcttgacttagcgagtgacccggatgtagttatcacttcagtgtttggcccactgatacctcacccatccggcatggatctttacaacaacccgttttcaaaagctgtatggttggctgtaccaccttatgagcagtcatttctaggaatgataaatacaattggattccctaacagagcagaggttatgccgcacattttgaccacagagatcagaggccctcggggtcgttgccatgttcccatagaattgtcccgccgggttgatgacgatatcaagatcgggtccaacatggtcatattgccgacgatggacctgaggtatattacagccacttatgatgtttccaggagcgagcatgcaatcgtgtactatatctatgacacgagtcgctcatcatcttacttctacccagttcgactgaatttcaaaggcaatcctctctctttgaggatagagtgtttcccctggcgtcataaggtgtggtgctaccatgattgtcttatatacaacaccataacaggtgaagaggtccatacgagagggctgaccggcatagaggtaacatgcaatccagtctgagcagagctacgaccgtcactcaggcagtcccttgagtcgccaccgagtccaagcagcacagcctgggacaatcaacggcacagcccagccaacaatgttataaaaaacttaggagccagggttgtaggggccatggactctctatcagtcaatcaggtcttgtaccctgaggttcatctagacagccctattgtcacaaacaaactagttgccatccttgagtactcgggtatcgaccataactatgttcttgaagaccagactcttgtcaagaatattaggtataggctggggtgcggtttttcaaatcaaatgatcatcaataacaggggggtgggtgaaacagtcaattctaaacttaaaagttacccccataatcgtcatatcatatacccggattgcaataaggagttgttttgtatcaaggatagctgcatatctaagaagctctcggagctattcaagaagggtaattccttgtactctaagataagtcaccaggtactggattgtcttaagagagtcaatgggaaattaggtctgggcacagatcttacccatggtctgaaggagggtatccttgacctagggttgcacatgcatagctctcaatggttcgagactttcctgttctggttcactatcaagacagagatgagatcaatgatcaaagaacagtcccatatatgccacaagaggaggtataacccgacttttgtgtcgggggatgcattcgaggtgctcgtatcgcgagacctcgttgtgataattgataagaatacccagtatgtcttctacttaacatttgagctggtccttatgtattgtgatgtcatagagggtagacttatgacggagacagccatggccatagaccagagatattcagaacttctaagccgggtcagatacttgtgggatcttattgatgggttcttcccaacactaggcaacaccacataccaaattgttgctctgcttgaaccattatcgttggcttatcttcaacttcaggatgtcactctggagttaagaggtgcttttttagaccattgcttcaaagaactctatgagatactggaacattgcggcattgacacagcaggcacttacaattccatcactgaaggattggattacgtattcatcacccacgatatacatctaactggggagattttttcatttttccggagtttcggacacccccgtctagaagcagtcaccgctgcggaaaatgtcagaaaacatatgaaccaaccgaaggtaatcagttatgagactatgatgaaaggccatgcggtattttgcgggataatcataaatggttttcgggaccgacatggcggcagctggccccctgttgcactgccagaacatgcctctgctgcgatccggaatgcgcaggcatcaggtgagggactgacccatgacctgtgtatagacaactggaaatcctttgttggattcaaatttggctgctttatgccactcagcctagatagtgatttgaccatgtatctcaaagacaaggcattggctgcactgaagaatgagtgggattcagtttacccgaaagaatacctccgttataacccacctagagggacagagtcaaggcgattggtcgaggtattcctgaatgactccagctttgatccttataacatgataatgtacgtggtgaatggctcctaccttaaagaccctgagtttaacctctcatacagcctaaaagagaaggagataaaggagacagggcggttgtttgccaaaatgacctataagatgcgggcctgtcaggtaatcgctgaaaatctgatatcgaatggtgttgggaagtatttccgagacaatgggatggcaaaggacgagcatgacctaacaaaagcccttcacaccctggcagtctcaggtgttcccaaaaacaacaaagataaccaccgaggtgggcctcccagaaggacaacaaaccgaggggtgagatcaagccaaggcaccaaaacacaagatagagacaaggttcaagggggacctatgtacaactatttgcgatgccaaccgatcagccctgatcagggtgagtcatacgagactgttagtgcattcatcaccgctgaccttaagaagtattgcctgaattggagatacgagacaatcagcatatttgcacagaggctgaatgaaatatatggactaccatccttctttcaatggttacacaggatattggaaaaatccgtactctacgtcagtgacccacattgtcctcccgatctagataatcatatccctctggacagtgtccctaatgcccaaatattcatcaagtacccaatgggcggaatagaaggttattgccaaaaactatggacaatcagcactataccatacttgtatctggcagcctatgagagcggagtaagaatcgcctcactggtgcagggtgacaatcagacaatcgcagtgacaaaaagagttccaagttcttggccttattcactaaaaaagagggaggcctccaaggccgctcaaaattacttcgtagtcttaaggcaaagattgcacgatgtaggtcatcacttaaaggctaatgagaccatagtatcttctcacttttttgtatattccaaagggatttattatgacggcctgctagtctcacaatcactaaagagcatcgccagatgtgtcttctggtccgagactatagtggatgaaaccagagcggcgtgcagtaatattgcaacaactgtcgctaagagtatagagaggggttatgataggtaccttgcatattctttgaatatcctcaagattttccaacagatccttatatcccttaacttcactattaacacaacaatgactcaggatgtcgtggcaccgatcatcgagaacggtgatttgctgataaggatggcactcttgccagcacccatcgggggtctcaattatcttaacatgagcaggttatttgtgagaaatatcggtgacccggtcacctcctctatagccgacctgaagaggatgatagatgccgggctaatgccagaagaaacattgcatcaagtgatgacccagaccccgggagaatcatcctacctagattgggcaagtgacccttattctgccaacctaccctgcgtacagagtatcactcgccttctcaaaaacatcactgcacgatatattttaatcagcagcccaaacccgatgctgaaagggttatttcatgaggggagtagagatgaagacgaggagcttgcaagtttcctaatggatcggcatataattgttccaagagctgcacatgaaatcttagaccatagtataaccggagcaagagaagctatagccgggatgttggacaccaccaagggtctgattagaacaagtatgaaacggggcggcctcacccctcgagtcttagcccgcctttccaattatgattatgaacaattccgatccgggataacactattaacaaagaaagggcagtgttatctcattgacaaggactcgtgctcggtgcagctcgctatagctctgaggggccacatgtgggctaagttggctcgcgggagaccaatctatgggttggaggtgcctgatgtattagaatcgatgaacggctaccttatcaaacgccacgagtcgtgtgctatctgtgaaacgggctcaagtcactacggatggtttttcgtccctgcagggtgccagcttgacgatgtatctagagagacttcggctcttcgtgtaccttatgtcggatcaacaactgaggaaaggacagacatgaaacttgcatttgttaggtctccaagtagatccctcaaatcagcagttagaattgcaacagtttactcatgggcttacggggatgatgagaaatcatggagtgaagcctggatgctagctaggcagagagccaatatcaccttagatgaattgagaatgatcactccagtctccacatccaccaacttagcccaccggttgagggatcggagtacccaggtgaaatactcggggacatcccttgtgagggttgcaagatacacaaccatctccaatgataacttgtcgtttgtgatatctgagaaaaaagtggataccaacttcatttatcagcaagggatgttgcttggtcttgggatccttgagaacctctttaggctagaggccaccaccggagtatccaacacagtgctacacctgcacgtggaaacagaatgttgtgttgtacctatggtggatcacccaaggataccgagtctccgtaatataaaagttacggatgagctatgcacaaaccctttgatctacgacaggtcccccatcatagagcacgatgcaacccggctatattcacagagccacaggagacatttggtggagtttgttacctggtcaacaagccagctttaccatatactggctaagtctacagcaatgtccatgattgaattgatcacgagattcgagaaagatcacatgaatgaaatagccgccctgattggcgatgacgacatcaacagcttcatcacagaatttttgctagtggagcctagattatttatagtttaccttggtcagtgtgctgccatcaattgggcctttgatatacattatcatcggccctcgggcaagtaccagatgggggaacttctctactctctgctctctcggatgagcaaaggagtatataagatctttactaatgctttgagccaccccaaagtttacaagaaattttggcgaagtggcatcattgagccgattcatggcccatccttggatacacagaatttacatgtcactgtctgtgacatgatatatgggtcctatgtcacctatttggatcttttgctgaatgatgagctagatgattacccgtatttgctctgcgagagtgatgaggacgtggtcacagacaggttcgacaacattcaagccaaacatctctgtgtattggccgatgtatattgcagctccaagaggtgtccttcaataatcgggatgtctcctatagaaaaatgtaccattctcacacattacatcaagggagaatcagtacaatccccgtctgggacctcatggaacactgatccccttgtagtagatcattactcatgctctctgacctaccttcgccgcggttccatcaaacaaatcaggttgagggtggatcctgggtttgtattcgaggcgttgacagacgtcgactttaaacaacctcgcaaagctaagttagatatatcggtcgttgggttgactgatttctctcccccttacgacaacgtcggtgattttttagggactatcaacacattgaggcacgatctgcctgtcaccggaaccggggtctcgaactatgaagtccacgcttatcgtagaattggcctgaattcatcagcatgttataaagccgtagagatctccacgttaatcaagccatccttagaagtcggagagcatggattgttcttaggagaaggttccggttcaatgctggctgcgtacaaagaagttcttaaattagcaaattgttactacaacagcggagtaacagcggagggcagagccggacagagggaaatatctccctatccttcagagatgagcctagtagagaatcaaatggggatagagcggagtgttaaagtgctgttcaacgggaaacctgaagtaacttgggtggggaccacagattgctacaagtacataatcagtaacatccaaacctctagtctgggtttcatacattcagatattgagacgctcccaaccaaagatgctgttgagaaattggaggaatttgcctctatcctatccttatccttgattttggggaaaatcggctctattacagttgtcaaaattatgcccattagcggggattttacccaaggcttcatagcctatgccatccaatattttagggagagcctgcttgcctatccgagatatagtaacttcatctcgactgagtgttaccttattatgataggattaaaggccaatcgattgataaacccagaagccattaagcaaagcataatcagagcgggggtcaggactgcaccaggacttgtgagccatgtattatcagagaaacaaaaaggttgtattcaatctttcttgggtgatccttatatccaaggagacttcaataagcaccttaaatctctaacctctattgagaaaatcctggtaaattgtggtctctcgatcaatggcataaaaatctgtagagatctaatccaccatgatatcgcctccggtccagacggccttatgagctctacaattattttatatagggaattggctcatttcaaagataatataaggagtcagcacggaatgttccacccctacccggtactggccaacagcaggcaacgtgaattaatccttcgaatagccaagaaattctgggggtatgtcttgctatattctgatgacccggcactaatcagacaaacaatcaagaacttgaaacggaatcatctaacctttgacttacatagtaacccatttattaaagggctatccaaggctgagaaactgctggtgcggacgagttcactaagaagagaatggttgttcactctcgaaacgaaagaagtaaaagagtggttcaaattggtaggttacagtgcactcatcagaggctagtcagctatgcatctgcccccttctcctccgccatgagaccccactggcgatccagaagattaaagaaaactacatattggataagtatctattcccagctttgtctggt

>KM212177/Senegal/Dakar/2013/11

accaaacaaagttgggtaaggatagatcttacaatgactatagactagcaaacttaggagtaaagatcctactgtcggggagaggaggaggagcaagatctttgaccatggcgactctccttaaaagcttagcactgttcaagaggaacaaagacaaagcgcctactgcgtcaggttcaggaggggccatccgggggattaagaatgttatcatagtccctatccccggggactcatccatcattacccgctcaagactgctcgacaggcttgtcagactggccggagatcctgacatcaacgggtcaaagctgaccggcgtgatgatcagcatgttatctttgttcgtggagtcacccgggcaattgatacagcggatcacagatgatccagatgttagcatccgtcttgttgaggtagttcaaagtaccaggtcccagtccggattgacctttgcatcacgtggtgctgatttggacaatgaggcagatatgtatttttcaactgaaggaccctcgagtggaagtaagaaaaggatcaactggtttgagaacagagaaataatagacatagaagtgcaagatgcagaagagttcaatatgttgttagcctccatcttagcacaagtttggatcctcctggccaaggcggttacggcaccagatacagcagccgactcagaactgagaaggtgggttaaatacacacaacaaaggagagtgattggggaatttcgctttgacaaagggtggttggacgcagtccgcaacaggattgcagaagatctatcactccggcggttcatggtatccctgatacttgacatcaagaggacccccggcaacaagccaaggattgcagaaatgatctgcgacattgacaactatattgtcgaggccggactcgcaagtttcattcttactatcaagtttggtattgaaaccatgtatcctgcactaggccttcacgagttcgctggggaattgtccactattgaatccttgatgaacctgtatcaacagctaggagaagttgcaccctatatggtaattctagagaactcaattcagaacaagtttagtgcaggagcctatcctctcctctggagctatgcgatgggtgtcggagtcgagttggagaactcaatggggggcttgaactttggcaggtcatattttgacccggcctattttcgtctcggacaggagatggtcagaagatctgcaggaaaggtcagctctgtaattgcggctgagcttggcatcacagcagaggaagccaaactagtctcggaaatcgcctcgcagactggggaagaacggaccgttagagggactgggcctcgacaggcgcaggtttccttcctccagcacaaaataggtgagggagagtcgcctacaccagcgaccaaagaagaagtcaaagctgcgatcccaaacggatccgaaggaagagacataaagcgaacacgcccagggaagcccagaggagaaactcccgggcaactgcttctggagatcatgccagaggatggagtctcgcgagagtctggtcaaaaccctcgtgaggctcaaagatcggctgaggcactcttcaggctgcaggccatggccaaaattctggaggaccaggaggagggagaagacaacagtcagatctacaacgacaaggatctcctcagctgagcagacacaccctccgtccaaatcagcgacaagacatcacctgtcagtattataaaaaacttaggacccaggtccaaccaaccgcacatcgacaccccagtcaatcgagcggagaccaccgatggcagaagaacaagcataccatgtcaacaaggggctggaatgtatcaagtccctcaaagcctctcccccggatctatccaccatccaagatgcccttgagagctggagagaggggttcaacccctcaggccgtgcaacaccgaaccatgatacgtccgagggggaccatcagaatatcaaccaatcatgctcatcagcaatcggatcagacaaagtcgacatgtctcctgaaggtaatctcggatttagagaaatcacttgtgatgacaatgaggctgggctcggaggagttcaggacaaaagatccgactctcaagtacagcgttattatgtttataaccacgggggtgaagagattgaaggactcgaggatgctgactctctcgtggttcaagcaaatcctccgattgctaacaccttcgatggaggagaggatggatctgacaacagcgatgtggactctggcccagatgatcccggcagagatcctctatatgaccggggacctgctgccggcaatgatgtctctaggtcaacagatgtcgaaaaattagaaggtgatgacattcaagaagttcttaactcccagaagagtaaaggaggaagattccagggcgggaaaatattgcgagtcccggaaatacccgatgtcaagaactccagaccatcggcccaatcaattaaaaagggcacagacgggagctcagtcttatctggaatggtgacagagtgttcatcgataagtggtgcaacccaagctgtgctagagtccagatgggagtcatcagagcgaaatgcatctgtggggagtgtccccaaatctgtgaggagtgcaaagacgatccaggggttaacacaagaatctggtaccatagcatcactgactcagcctaaagaaaatgactccgagtatgagtatgaggatgatctattcactgagatgcaggacattcgtgcaagcattgctaagatccacgatgacaacaaaactctcctctcaaaacttgattctctactgttattgaaaggagaaatcgatactatcaagaaacaaatcagcaaacaaaatataagtatatctaccattgagggccatctatccagtataatgatagccatcccaggttttgggaaggacatcaaggacccaacatctgaggttgagctgaacccagatttgagacctataatcagccgcgattctggcagggctcttgcggaggtccttaagaaacccgctgttgataggtctcagaagagcggaatcaaagtcaactccggttcaaagggtcagctcctcaaggatctccagctaaaacccgtcgacaagcaggcaagctctgcaatcgggtttgttccatccgaccatgaatcatccagaagtgtcatccgctccataatcaagtcgagcaagcttaacattgatcacaaagactatcttctagatttactgaatgatgtgaaaggctctaaggatctcaaggaattccacaagatgctgacagccattcttgccaagcagccgtaacacatcccataatctacatctcatactcggttgaaagcatcctctcaatcaggctattacaaaaaacttaggagcaagggcaactgagcttcacagacaggatgaccgagatttacgatttcgacaaatcagcatgggatgtcaaagggtcaattgctcgcatagaacccaccacctatcacgacggccgactggtaccccaggtgagggtcatcgaccctggtctgggagacagaaaagatgagtgcttcatgtacctgttccttctaggagtgattgaggataacgaccccctttctcccccagtcgggagaaccttcgggtctttacctctaggtgtcggcaggtcaactgctaagccagaggaactactaagggaggccacagaactagatatagtggtgaggcgcacggcaggactaaatgagaaattggtgttttacaacaacactccgctatctttgttaacaccatggaggaaagtcttgacgaccggaagtgtgttcagcgccaaccaggtttgcaatgcggtcaacctagtcccacttgatactccccagagattcagggttgtgtacatgagtataactagattgtcagataatggttattatagtgtccccagaagaatgttggagttccgctcagccaatgcagtcgctttcaatatcttggttacactgagaattgaaaatggcacaaaccctagaagatacatagtcggctcatgggagaatccagaagtcacatttatggtacacgtgggcaactttagaagaaagaagaacgaagtatactctgctgattattgcaaaatgaagattgaaaagatgggtctagtttttgccttgggaggaataggtggaacaagtctccatattcgaagcacagggaaaatgagcaagaccctccatgcacagctggggttcaagaaaattctatgctaccctctgatggatatcaatgaggatcttaaccgatatctctggcgggcagagtgccgaatagtcaaaatccaggccgtcttacagccatcagtaccccaagaattccgtgtctatgatgatgtcatcatcaacgatgaccaaggcctgttcaagatcctgtaagtcacctgcgacatcatgacacgggtcgcaaccttggtattgctggttcttttccctaacactgtcgcgtgccagattcactggggcaatctatccaagatagggattgtcggaacagggagtgccagttacaaggtgatgaccagaccaagccaccaaactctggttataaagttgatgccaaatataacagccatcgacaattgtacaaaatcagagatttcagagtacaaaagattgctaatcacagtgttaaagcctgtagaggatgctctgtcagtaataaccaagaatgtaagaccaattcaagctctaacacctgggcgcaggacccgccgtttcgccggagctgttctggccggagtagcacttggagtcgcgacggctgctcaaataactgccggagtcgcactccatcagtcattgatgaattcccaagcaattgaaagtttaaaaaccagtcttgagaagtcaaatcaggcaatagaagaaatcagacttgcaaataaggagaccatactggcagtacagggcgtccaagattatatcaacaacgagcttgtcccctctgttcatagaatgtcatgtgagctaataggtcacaaactcagtctcaaactccttaggtattataccgagatcctgtctatattcgggcctagccttcgagacccgatagctgctgaaatatcaatccaggcactcagctatgcactaggcggagacatcaataaaattctggacaagcttgggtatagcggcggggatttccttgctattctagaaagcaaggggataaaggcccgggtcacatatgtggacacaagagattactttataattcttagcatagcctacccaaccttatctgagatcaaaggggtgatagttcataagatagaagctatatcatacaacattggggcacaggaatggtatactactatccctaaatatgtagccactcagggatatttgatatcaaatttcgatgagacatcatgtgtcttcactccagaggggacagtctgcagccagaatgcgctgtatccaatgagcccattgcttcaggaatgtttcagggggtcgacaaaatcgtgtgccagaaccctagtttcagggaccacaagtaatagatttatcctatcaaaagggaacttgattgcaaattgtgcgtcagttttgtgcaagtgttacacaacggagacagtcatcaaccaagatcctgataaactactaactgttatagcctcagataagtgtcccgtagttgaggtggatggagtgacaatacaagtcggcagtcgagagtacccagattctgtatacctacataaaatagacttaggcccagccatctctctggaaaaactggatgtaggcaccaatttaggcaatgcagtcacaagactggagaatgcaaaggagctcctagacgcatcagaccagatactgaagactgtcaaaggggtacccttcagtggcaatatgtacatagcactggcagcttgcattggggtatccctagggcttgtcacattaatatgctgctgtaaggggagatgtaggaacaaggagattcctgcctccaaaatcaacccagggctcaaacccgacctgaccgggacttcaaagtcttacgtgagatcactgtagtcagaataacccgaatcacccggcatcacgcgtatacatgtgcgacacaagcagtcagaggacgcagaagactcaacctccgatcaccgaccagaccccactctacgccctactgcacattggtcatcaaacaaaacttaggacgaaaggtcaatcaccatgtccgcacaaagggagaggatcaatgccttctacaaagacaaccctcacaataaaaaccataggataatcctggatagggaacgcttaactattgaaagaccctacatcttacttggagtcctgctggtaatgttcttgagtctaatcgggctgctagccattgcagggatcaggcttcaccgggccaccgttggaactgcggagatccagagtcggctgaataccaacattgagttgaccgaatccattgatcatcaaactaaggatgtcttaacacccctgttcaaaatcattggtgatgaagtcggtatcaggattccacagaagttcagtgatcttgtcaagttcatctccgataagattaagttcctcaaccctgacagagaatacgattttagggatctccggtggtgtatgaacccccctgagagagtcaaaattaactttgatcaattctgtgaatacaaagccgcggtcaagtcagttgaacatatatttgagtcatcattcaacaggtcagaaagattgcgactattgactcttgggcccggaacaggctgtctcggcaggacagtaacaagagctcagttctcagaacttacgatgaccctgatggacctggatctcgagacgaagcacaacgtgtcctcagtgtttaccgtagttgaagagggattattcggaagaacatatattgtctggagatctgacaccgggaaaccgagcaccagtctagatattggccagtttttaagagtcttcgagatcgggttggtgagggatctcgagctgggtgcccccattttccatatgaccaactacctcacagtgaacatgagtgatgactatcggaattgccttttagcggtaggggagttgaagctgacagccctatgcaccccatctgagactgtgactctgagtgagagaggagttccaaagagagagcctcttgtggttgtgatactcaacctagttgggcctactctagggggcgaactatacagtgtcttgcctacctctgacctcatggtggagaaactccatctatcctcacacagagggatcatcaaagacaacgaggccaattgggtagtaccgtctactgatgttcgtgatctccaaaacaaaggagaatgtctggtggaagcatgcaaaacacgacctccttcattttgcaatggcacaggaataggcccatggtcagaggggagaatccctgcctacggagtgatcagggtcagtcttgacttagctagtgacccaggtgtggttatcacctcagtgtttggcccactgatacctcacctatctggcatggatctttacaacaatccgttttcaagagctgcatggctggctgtaccaccctacgagcagtcatttctaggaatgataaatacaattggcttcccggacagagcagaggtcatgccgcacatcttgaccacagagatcaaagggcctcgaggtcgttgtcatgttcctatagagttgtcccgcaggattgatgatgatatcaagatcgggtccaatatggttgtattgccgacgagggatctgaggtacataacagccacttatgatgtttccaggagcgagcatgcaatcgtgtactatatctatgacacgggtcgctcatcatcttacttctacccagttcgattgaatttcaagggcaatcctctctctctgaggatagagtgtttcccctggtctcataaggtgtggtgctaccatgattgtcttatatacaacaccatgacaaacgaagaagtccatacgagagggctgaccggtatagaggtaacatgcaatccagtctgagtcgagctgaaaccatcgctcaagcaggctttcgagccatcccctagttcaagcagcatagtctgggacactcagcagcacaacccagccaacaatgttataaaaaacttaggagccaaggttgtaggagccatggactcactatcagtcaatcaggttttgtaccctgaggtccatctagatagccctattgtcacaaacaaactagttgctatccttgaatactcggggatcaaccacaactatgttcttgaggaccagactcttatcaagaatattagatatagattggggtgcggtttctcaaatcaaatgatcatcaataataggggggtaggtgaaacagtcaattccaagcttaaaagttacccccgtaactgtcatatcatatacccagactgcaataaggagttgttttgtatcaaagatagctgcatatctagaaagctctcggagctattcaagaagggtaattccttgtactctaaggtaagtcaccaggtactggattgtcttaagagagtcaacgggaaattagggctgggcacagatcttaatcacggcctgaaggatggtatcctcgacttggggttgcacatgcatagctctcagtggttcgagacctttctgttctggttcactatcaagacagagatgagatcaatgatcaaagaacagtcccatatatgccacaagaggaggtataacccaatttttgtgtcgggggatgcattcgaggtgcttgtatcacgagacctcgtagtgataattgataagaacacccagtatgtcttctacctgacgtttgagctggtccttatgtattgtgatgtcatagagggtagacttatgacggagacagccatggctatagaccagagatattcagagcttctaaaccgggtcagatacttgtgggaccttatcgatgggttcttcccaacactgggtaacaccacataccaagttgttgctctgcttgaaccactgtcgttggcttatcttcaacttcaggatgtcactctagagttaagaggtgcctttttggaccactgctttaaagaactttatgagatactggagcattgtggcattgacacggaaggtacctacaattccatcactgagggattggattacgtatttatcactcacgatatacacttaactggggagattttttcatttttccggagtttcggacacccccgcctcgaagcggtcaccgctgcagagaatgtcagaaaacatatgaaccaaccgaaggtaatcagctatgagactatgatgaaagggcatgcagtattttgcgggataatcataaatggttttagggaccggcacggcggcagctggccccctgttgcattgccagaacatgcttctgctgcgatccggaatgcgcaggcatccggcgaaggactgacccacgatctgtgtatagacaactggaagtcctttgtaggattcagatttggctgcttcatgccgctcagcctagatagtgatttgaccatgtacctcaaagacaaagcattggctgcattgaagaatgagtgggattcagtttacccgaaagaatacctccgttataatccacctagagggacagagtcaaggcgactagtagaggtgttcctgaatgactccagctttgatccttataacatgataatgtacgtggtgaatggctcctaccttaaagaccctgagtttaatctctcatacagcttaaaagaaaaggagataaaagagacagggcggttgtttgccaagatgacctacaagatgcgggcctgtcaggtgattgctgaaaatctgatatcaaatggtgttgggaagtatttccgagacaatgggatggcaaaagacgagcatgacctaacaaaagcccttcacactctggcagtctcaggcgttcccaagaacaacaaagacaaccaccgaggtgggccccccagaaggaacgcaagccgagagatgagatcaagccaagccgccaacacacaaaatagggacaagatccaagggggccccatgtacaactacttgcgatgccaaccaaccagccctgatcagggtgagtcatacgagactgttagtgcattcatcaccgctgaccttaagaagtattgcctaaattggagatacgagacaatcagcatatttgcacagagactgaatgaaatatatgggttgccatccttctttcaatggttacataaggtattggaaaaatccgtgctctacgtcagtgatccgcattgccctcccgacttagatgatcatatccctctggacagtgtccctaatgcccaaatattcatcaagtacccaatgggcggaatagaaggttattgtcaaaaactatggacaatcagtactataccatacttgtatctagcagcctatgagagtggagtaagaatcgcctcactagtgcaaggtgacaatcagacaattgcagtgacaaaaagagttccaagttcttggccttattcactaaaaaagagggaggcatctaaagcagctcaaaattactttgtggtcttaaggcaaaggttgcacgatgtaggtcatcacttaaaggctaatgagaccatagtatcttctcacttttttgtatactctaaagggatctattatgacggcctgttagtttcacaatcactaaagagcatcgccagatgtgtcttctggtccgagactatcgtggatgaaactagagcggcctgcagcaatattgcaacaactatcgctaagagtatagagaggggttatgataggtacctcgcatactctttgaatatcctcaaaattttccaacagatccttgtatcccttgacttcacgattaatacaacaatgactcaagatgtcgtggcaccgatcatcgagaacggtgatttactgataaggatggcactcttgccagcacccattgggggtctcaattatctcaacatgagcaggttgtttgtgagaaatatcggtgacccggtcacttcctccatagccgacctgaagaggatgatagacgctgggctaatgccagaagaaacattgcatcaagtgatgacccagaccccgggagaatcatcctaccttgattgggcaagtgatccttattctgccaacctaccctgcgtacagagtataactcgacttctcaagaacatcactgcacggtatattttaatcagcagcccaaatccgatgctgaaagggttgttccacgaggggagcagagatgaagacgaggagcttgcgagtttcttaatggatcggcatataattgttccgagagctgcacatgaaatcttagaccacagcataaccggggcaagagaagctatagccgggatgttggacaccaccaagggtctgattagaacaagtatgaaacggggtggcctcacccctcgagtattagcccgcctttccaattatgattatgaacaattcagatccgggataacactattgacaaagaaagggcagtgttatctcattgacaaggactcgtgctcggtgcagctcgctatagctctgaggagccatatgtgggcaaggttggctcgcgggagacctatctatggcttggaggtgcctgatgtactggaatcgatgaacggctaccttatcaaacgtcacgagtcttgtgccatctgtgaaacgggctcaagtcactacgggtggtttttcgtccctgcagggtgtcagcttgacgatgtctcaagagagacttcagctcttcgtgtgccttatgtcggatcaaccactgaggaaaggacagatatgaaacttgcttttgttagatctccaagccgatccctcaaatcagcagtcaggattgccacagtctactcatgggcctacggggatgatgagaaatcatggagtgaagcttggatgctagctaggcagagagccgatatcaccttagatgaactgagaatgatcactccagtctctacatccaccaacctagctcatcggttaagggatcggagcactcaggtgaaatattcggggacatcccttgtgagggttgcaaggtacacaaccatctccaatgacaatttgtcatttgtaatatctgagaaaaaagtagataccaacttcatttaccagcaagggatgctgctcggtcttgggatccttgagaatctattcaggttagaggccaccacaggggtatccaacacagtgctacacctgcacgtggaaacagaatgttgtgttgtacctatggttgatcacccaaggataccgagtctccgcaatatcaaagttacgaacgagctatgcacaaaccctcttatctacgacaagtcccctatcatagaacacgatgcaactcgattatactcacaaagccacaggagacatttggtggagtttgttacctggtcaacaagccagctctatcatatactggccaaatctacagcaatgtccatgattgagctgatcacaagatttgagaaagatcacatgaatgaaatagccgctctgattggcgatgacgacatcaacagtttcatcacagaatttttgcttgtggagcccagactgtttatagtttacctcggccagtgtgctgccatcaattgggcttttgatatacattatcatcggccctcgggcaagtaccagatgggggaactcctctactctttactctctcggatgagcaagggagtatataagatcttcactaatgctctgagccaccccaaagtttacaagaaattttggcgaagtggtataattgagccggttcatggcccatccctagatacacagaatttacatgtcactgtctgtgacatgatatacggatcatacgtcacctatctggatcttttgctgaatgatgagctagatgcttacccgtatttgctctgcgagagtgatgaggacgtggtcacagataggttcgacaacattcaagccaaacatctctgtgtactggccgatgtatactgcagctccaagaggtgtccctcgataatcgggatgtcccctatagaaaaatgtaccatcctcacacattacatcaagggagaatcggtacaatccccgtccgggatctcatggaacactgatccccttgtagtagatcattactcatgctctctgacctaccttcgccgtggttccatcaaacaaatcagattgagagtggatcctgggtttgtgttcgaggcgttgacagacatcgacttcaaacagcctcgcaaggctaagttggacgtatcgattgtgggattgactgatttttctcccccttgggataacgtcggtgattttctagggactatcaacacattgaggcacaatctgcccgtcaccgggaccggggtctcgaactatgaagtccacgcttatcgtagagttggtctgaattcatcagcatgttataaagctgtagagatctccacgttaataaaggcatccttagaagccggggagaatggattgttcttaggagaaggctccggctcgatgctggctgcgtacaaggaagttcttaaattggcaaactgttattacaacagtggggtaacagcagagggtagagccggacagagggaaatctctccctatccctcagaggtgagcctggtagagagtcagatggggatagagagaagtgttaaagtcctgttcaatggcaaacctgaagtaacctgggtagggaccaccgattgctacaagtatataatcagtaacattcagacctcaagtctgggtttcatacactcagatatcgagacactcccaaccaaggatgctgttgagaagttggaggaatttgcctctattctatctctatccctaattttgggaaaaatcggctctattacagttgtcaaaattatgcccattagcggagattttacccaaggcttcatagcctatgccattcaatatttcagagagagcctgcttgcctatccgagatatagtaacttcatctcgactgagtgttaccttattatgataggattaaaggccaatcggttgataaacccagaagccattaagcaaagcataatcagggtggggactaggactgcaccaggacttgtgagccacatattatcagagaaacagaaaggttgtattcaatcttttctgggtgatccttatgtccaaggagacttcaataagcaccttaaagctctaactcctattgagaaaatccttgtaaattgtggtctctcgatcaatggtacaaaaatctgtagggatctaatccaccatgatattgcctccggtccagacggtctgatgagctccacaattattctatacagggaactggcccatttcaaagacaatataagaagtcagcacggcatgttccacccctacccagtattggccagtagcaggcaacgtgaattgatccttcgaatagccaagaagttttgggggtatgtcttgctatattctgatgacccggcactaatcaaacaaacgatcaagaacttgaagcggaatcacctaacctttgacttacacagtaatccgtttattaagggcttatccaaagctgagaaactgctagtgcggacaagttcactcagaagggaatggttgttcactctcgatacgaaagaagtgaaagagtggttcaaattggtgggttacagtgcactcgtcagaggttaattagcgatacatctgcccccttctcctccaccatgagactctactggcaatccaaaagattaaagaaaactacatattggataagtatctattcccagctttgtctggt

>KM463083/Kenya/Kakuma/2011/5

accaaacaaagttgggtaaggatagatcttataataactatgaattgccacgcttaggattaaagatcctactgccggggggaggagtaggagcaagatccctgactatggcgactctcctcaagagcttggcattgttcaagaagaacagggacaaagcgccgacggcatcaggttcaggaggagccatccgggggattaagaatgttatcatagtcccgattcccggagactcatccatcattacccgttcaagattgctcgacaggcttgtcagattggccggagatccagatatcaacgggtcaaagctgaccggcgtgatgatcagcatgctgtctttgttcgtagagtcacccgggcaactgatccagcggatcacagatgatccagatgtcagtatccgccttgttgaggtggtccaaagtactagatcccagtccggattgacctttgcatcacgtggtgctgatttggacaatgaggcagacatgtattttgcaactgaagggccctcgagtgggagcaggaaaaggatcaactggtttgagaacagggaaataatagatatagaagtacaggatgcagaagagttcaatatgttattagcctccatactggcacaagtttggattctcctggccaaggcagttacagcaccagacactgcagctgactcagaattgagaaggtgggttaagtacacacaacaaagaagggtgattggggaattccgctttgacaaagggtggctagatgcggtccggaacaggattgcagaagatctatcactccggcggttcatggtatctcttatacttgacatcaaaaggacccctgggaacaagccaaggattgcagaaatgatctgtgacattgacaactatattgtcgaagccgggctcgccagtttcatccttactatcaagtttggtattgaaaccatgtatcctgcgctaggtcttcacgagtttgccggagagttgtccactatagaatccttgatgaatctatatcaacagctaggcgaagttgcaccctacatggtgattctggagaactcagttcagaacaagtttagtgcaggagcctatccacttctctggagctatgcaatgggtgttggagtcgagctggagaactcaatgggagggctgaactttggtagatcatattttgacccagcttattttcgtctcggacaggagatggtcagaagatccgcaggaaaggtcagctctgcaatcgcagctgagctcggtatcacagcagaggaagctaaactagtctcggaaattgcctctcagactggggacgaaaggaccgctagagggaccgggcccagacaggcgcaggtttccttcctccagcataaaataggagagggagagtcacatgcatcggcgaccggggaagaagtcaaagctgcgaccccaaatgggcccgacgaaaaggacaaaactcgggcgcgctcaggaaagccaagaggaggaacccctgaccaactgctcctggaaatcatgcctgaagacgaggtcccgcgagggtctggacaaaaccctcgtgaggctcaaagatcggccgaggcactctttagactgcaggccatggccaagatcctagagggccaagaggagggggaagacaacagccagacatacaacgacaaggacctccttagctgagaggaagagccctccatcaaaaccagcaacaagacaccgcccttcagtattataaaaaacttaggacccaggtccaagcagtcacactccggcaccccagccgaccgagcggagaccaccgatggcagaggaacaagcataccatgtcaacaagggactggaatgtatcaagtccctcaaagcctctcccccggatctctccaccatcagagatacccttgaaagctggagggaggggcttgacacatcggaccgtgcagcaccgaaccctgatatatccaagggggaccatcagaatatcaaccaatcatgcccaccagcaatcggatcaggcgaaatcgacgtgtctactgaaagtaatctcggatatagagagatcaatcacgatgacagtgaggctggactcagaggagttcaaagcagaggacccaaccctcaaatacagcgttaccatgtttatagccacgggggtgaagagattgaaggactcgaggatgctgactctctcatggttcaagcagatcctccacttgctaacaccttcagtagaggagagggtggatctgacgacagcgatgtggactctggcccagatgatcccgacagagatcctctatatgaccggggatctgttgccgacaatgatgtcgttaagtccacagatgtcgagaaattagaaggtgacaacattcaagaagttcttaactctcagaagagcaagaggggaaaattccaaggcgggaaaaccttgcgagtccctgaaacgcccgacgtcaagcaccccagaccatcagcccaatcaattaaaaagggcacagacgggaactcagtcttatctggaacggtgacagagtgttcatcgataaatggtgcaacccaagctgtgccagagtcaagatgggagtcatcagagcgaaatgcatttgtggagagtgcccccaaatctgcgaggagtgcaaagacgatccaggagttgccacaagaatctggtaccatagcatcaccgactcagcctaaagaaaatgactccgagtatgagtatgaggatgacctatttatagagatgcaggacattcgtgcaagcattgccaagatccatgatgacaacaaaaccatcctctcaaagcttgactctatactattattgaaaggagaaatcgatactatcaagaaacaaatcagcaaacagaatataagtatatctaccattgagggccatctttccagtataatgatagccatcccgggtttcgggaaggaggtcaaggacccaacatccgaggttgagttaaacccggatttaagacctataatcagccgtgattctggcagggctctcgcggaggtcctcaagaaacctgctgttgataggtctcctaaaaccggattcaaagtcaactccggttcaaagggtcagctcctcaaggatctccaactgaaacccgttgacaaacaggcaagttctgccatagggtttgtcccgtctgaccatgaatcatccagaagtgtcatccgctctataatcaagtcaagcaagctcaacattgatcacaaagactatcttttagatttactgaatgatgtgaaaggctccaaggatctcaaagaattccacaagatgctaacagctattcttgctaagcaaccgtaatgcaccgcctaaccaatttctcatgctcagctgaaaacagccccacaaccaggctattacaaaaaacttaggagcaagggcaaccgagcttcgcagacaagatgaccgagatctatgacttcgacaaatcagcatgggacgtcaaaggatcaatagctcccatagagcccaccacttatcacgatggtcgactgataccccaggtgagagtcatcgatcctgggctaggagacaggaaagatgaatgcttcatgtacttgtttctcctaggggtgattgaggataatgaccccctgtctcccccagtaggaagaacctttggttccttacctctaggtgtcggtaggtcaactgctaagccagaagaactattaagggaggccactgaacttgacatagtggtgagacgcacagccggactaaacgagaagctggtattttacaacaacactccgctgtctttgttaacaccttggaggagagtcctgacaaccggaagtgtgttcagcgctaaccaggtttgtaacgcagtcaatctaatcccactcgatactccccagagattcagggttgtgtacatgaccatcactagattgtctgacaatggttattatagtgtccccaagagaatgttagagttccgctcagccaatgccgtcgccttcaatatcctggtgacactgagaattgagaatgggacaaaccctagacgatacatagtcggctcatgggagaacccagaggtcacattcatgatacatgtgggcaactttagaagaaagaagaatgacgtatattccgctgattactgcaaaatgaagattgagaagatgggtctggtttttgctttgggaggaatagggggaacaagtcttcacattagaagtaccgggaaaatgagcaagaccctccatgcacagctggggttcaagaaaatcttatgctaccccttaatggatattaatgaggatctcaatcgatatctttggcgggcagagtgccgaatagtcaaaattcaagctgtcttacagccttcagtaccccaagaattccgtgtctacgatgatgtcatcattaatgatgaccaaggcttgttcaagatcctgtaactcactcacaacatcatgacaaaagtcgcaatcttgacatccctgtttctcctcaccagcactgtcgtatcccagattcactggggcaacctatccaagatcgggattgtggggacaggaagtgccagttataaggtgatgactaggccgagccaacagaccctggttataaagctaatgccaaatataacagccatcgacaattgtacgaaatcagagatttcggaatacaaaagactgctgatcacggtgttaaaacctgtcgaggatgctctgtcagtgataaccaagaatgtaagaccaattcaagctctaacacccggtcgcaggactcgccgttttgccggggctgtcctggccggagtggcacttggggtcgcaacggctgctcagataactgccggagtcgcactccatcaatctttgatgaactcccaagcaattgaaagtctaaaagccagtcttgagaagtcgaatcaagcaatagaagaaatcagacttgcaaataaggaaaccatactggcagtacagggtgtccaagattacatcaacaacgagctaatcccttctgttcataagatgtcatgtgagttggtaggtcacaaactcagcctcaagctccttaggtactacaccgaaatcttgtctgtattcgggcccagccttcgagacccgatagctgctgaaatatcaatccaggcactcagttatgcactaggtggggatatcaataagatcctggataagcttggatatagcggcgaggacttccttgctatcttggaaagcaaggggataaaggcccgagtcacctatgtggatacaagagattactttataattcttagcatagcctacccgaccttatctgagatcaaaggggtgatagttcataaaatggaagctgtgtcatacaacatcggggcacaagaatggtacactactatccctaaatatgtagccactcagggatacttgatatcaaattttgacgagacgtcatgcgtgttcactccaggagggacagtctgcagtcagaatgctttatacccaatgagcccgttgcttcaggaatgtttcagggggtctacaaaatcgtgcgccagaaccctagtttcagggactataagtaatcgatttatcctgtcaaaagggaacctgattgccaattgtgcatcagtcctgtgcaaatgttacacaacggagacgattatcagccaagatcctgataaattactaactgttatagcctctgataaatgtcctgtagttgaagtggatggagtgacgatacaggtcggcagtagggagtacccagattctgtgtacctacataaaatagacttgggcccagccatctctctagaaaaactggatgtaggcaccaatttaggcaatgcagttactaggctggagaatgcaaaggaactcctagatgcatcagatcagatattgaagactgttaagggggtacccttcagcggcaatttgtacataggactggcagcttgcatcggggtatctctagggcttgtcacattaatatgctgctgtagggggcggtgtaggaacaaggagattcctacccctaagatcaacccagggcttaaaccagacctgaccgggacctcaaagtcgtacgtaagatcactgtaatcagaatagcccgaatcatccagcgtcacacacagacatgtgcaacacaagcagtcaggggactcagagggttcaacttccgatcctcaacctgatcccattccaagccccattgcacatcggttattaaataaaacttaggacgaaaggttagtcaccatgtccgcacaaagagagaggatcaatgccttctacaaagacactcctcacaacaagaaccatagggtgaccctggataaggagcgtctgactattgaaagaccctatatcctgcttggggtcctactggtaatgtttctgagtctgattgggttgctggccattgcagggattagacttcaccgagccaccgtcggaactgcagagatccagggccggcttaataccaacattgagttgactgaatctattgatcatcaaactaaggatgtcctaactcccctatttaaaatcatcggtgatgaagtcggcataaggattccacagagattcagtgaccttgtcaagttcatctccgataagattaaattcttcaaccctgacagagaatatgatttcagggacctccggtggtgtatgaatcctccagagagagttaaaatagattttgatcagttttgtgaatacaaagccgcggataagtcgattgaacatatatttgagttaccactcaacaggtcaaaagagttgcgatcgttgactcttgggcctggaacgggctgtcccggcagggcagtaacaagggcccaattctcagagcttactctgactctgttggatctggatctcaatatgaagcacaacgtgtcctcagtgtttaccatagttggagagggattattcggaagaacatataccgtctggagatcatatgccggaaatctgagtaccgcctcaggtctcggccattttttaagagtcttcgagatcggactggtaagagattttgggctaagtgcccctgttttccatatgactaactatctcacagtgaacatgagtgatgactaccggagctgtctattagcagtgggggaattgaagctgacagctctatgcacccattctgagactgtgactctgagtgagagaggagttcctaagagagagcctcttgcggttgtaatacttaatctagccgggcctactctagggggcgaactatacagtattttgccttcctctgacctcatggtagaaaaactctatttctcatcaaatagggggattatcaaagacaacgaggccaattgggtagtaccatctaccgatgtacgtgatcttcaaaataaaggagaatgcctggtggaggcatgcaaaactcgacctccttcattttgcaatggtacagggttaggcccatggtcagaggggagaatccctgcctacggggtgatcagagtcagtcttgacgtagctagtgatccagatgtggttattacctcagtgtttggccccttgatacctcatctatctggcatggatctttacaacaacccattctccagagctgtgtggttggctgtaccaccttataagcagtcatttctgggaatgataaatacaattgccttcccaagtagagcagaggttatgccgcatattttgaccacagagatcagagggcttcggggtcgttgtcatgttcccatagaattgtcccgcaagactgatgatgatattaagattggctccaacatggttgtattaccgacgaaggacctgaggtacattacagccacttacgatgtttccaggagcgatcatgcaatcgtgtattatatctatgacacaggtcgctcgtcatcttacttcttccccgctcgattaaagctcaaaggtaaccctctctctatgaggatagagtgtttcccctggcatcataaggtatggtgctaccatgactgtctcatatacaacaccaccacaaacgaagaagttcatacgagagggctaactggcatagaggtaacatgtaatccagtctgagcagagctgcgaccatcaatcaagcaagcttccgagccattcccaagccaaagcatcacacctaggggcactcaacagcacaactcagccgacagtgttataaaaaacttaggagccagggctattggagccatggactccctgtcagtcaaccaggtcctataccctgaggtccatttagacagccccattgtcacaaacaagctagttgccatccttgaatactcgggcatcgaccacaactatgttcttgaggaccagactcttatcacgaatattagatataggttgggatgcggtttttcaaatcaaatgatcatcaataacaggggggtaggtgaaacagtcaattccaaacttaaaagttacccccgtaattgtcatgtcatatatccagattgcaataaggatctgttttatatcagggacggctgcatatctaggaagctctctgagctattcaagaaaggtaattccctgtactctaagataagtcaccaggtactagattgtctcagaagagtcaacggcaaaatgggcctgggcacagatcttagccatggcctgaaggagggtatcctcgatttagggttgcacatgcatagctctcagtggtttgagaccttcctgttctggttcactatcaagactgaaatgaggtcaatgatcaaagaacaatcccatatatgccacaagaggaggtataacccaacctttgtctcaggagatgcactcgaggtgcttgtatcacgggacctcgttgtgataattgataaaaccactcagtgtgtctattacctgacattcgagctggtccttatgtattgcgatgtcatagagggcagacttatgacggagactgccatggctatagaccagaggtattcagaactgctaagccgggtcaaatacttatgggatcttattgatgggttcttcccaacactaggtaacgccacataccaagttgttgctctgcttgaaccgttatcgttggcttaccttcaacttcaggatgtcactctggagttaagaggtgcattcttggatcactgcttcaaagaactctacgagatactggaacattgtggcgttgacacggaaagcacctacgattccatcatcgaaggattggattacgtatttatcacccacgatatacacttaactggggagattttctcctttttccggagtttcggacatccccgccttgaggcggtcactgccgcagagaatgtcaggaaacatatgaaccaacctaaggtaatcagttatgagactatgatgaaagggcacgcagtgttttgcgggatcatcataaatgggtttagggaccgccatggcggcagctggccccccgttgtgctgccagaacatgcctctgctgcaattcggaatgcgcaggcatccggtgaagggttgacccatgacctgtgtatagacaactggaagtccttcgttggattcaggtttggctgttttatgccgctcagcttagacagtgacctgaccatgtatctcaaagacaaagcactagctgcactgaaaaatgagtgggattcagtttatccgaaagaatacctccgctataacccacccagagggacggagtcaaggcgactggtggaggtgttcctgaatgactccagctttgatccttataacttgataatgtacgttgtgaatggctcctaccttgaagaccatgagtttaatctctcatacagcttgaaagagaaagagataaaagagacagggcgactgtttgcaaaaatgacttacaaaatgcgggcctgccaggtcattgcggagaatctgatatcaaatggggtagggaagtatttccgagacaatggaatggcgaaagatgagcacgacctaacaaaggcccttcataccttggcagtctcaggtgttcctaaaaacaacaaggataatcaccggggtgggcctcctagaaggaccacaagccaaaagatgaggtcagatcggggcatcaccagtcaagatagggacaaaatacagagagagcccatgtataactatctgcgatgccaaccagtcagccctgatcagggtgagtcatacgagactgttagtgcattcatcaccgctgacctgaagaaatattgcctgaactggagatacgagacaatcagcatatttgcacagagactaaatgagatttatggactaccatctttcttccaatggttacacagggtattagaaaaatctgtgctgtacgtcagtgacccacattgccctcccgacttggacgatcatatccctctggacagtgtccccaatgcccaaatattcatcaagtacccgatgggcgggatagaaggttattgtcaaaaattatggacaatcagtactataccatacttgtatctagcagcccatgagagcggagtaagaatcgcctctctagtgcaaggtgataatcaaacaattgcagtgacaaagagagtcccgagttcctggccttattcactaaaaaagagggaggcatctagagcagctcagaattacttcgtggttttaagacagaggttgcatgatgtaggtcatcacttaaaggccaatgaaactatagtatcttctcatttttttgtatactcaaaagggatttattatgatggcctgttagtatcacaatcactaaagagcattgccagatgtgtcttctggtccgagactattgtggatgaaaccagagccgcctgcagcaatattgcaacaactgtcgccaagagtatagagaggggttatgacaggtaccttgcttactccttgaacatactcaaaattctccaacagatccttatatcccttgacttcactatcaacacaacaatgactcaagatgtcgtggcaccgatcatcgagaacggtgacctgctaacaaggatggcacttttgccagcacccatcgggggccttaactacctcaacatgagcaggttatttgtgagaaatatcggtgacccagtcacttcctctatagccgacctgaagaggatgatagacgctggactaatgccagaggaaacattgcaccaggtgatgacccagaccccgggagaatcatcttaccttgattgggcaagtgacccttattctgccaacctaacctgtgtacagagtataactcgtcttctcaagaacatcactgcaaggtacattttaaccagcagcccaaatccgatgctaaaagggttattccatgaagggagcagagatgaggatgaagagcttgcgagtttcctgatggatcggcatataattgttcccagagctgcacatgaaatcttagaccatagtataactggggcaagagaagctattgccggtatgttagataccaccaagggtctgattagaacaagtatgaaacgaggtggccttacgccacgggtcttagctcgcctttccaattatgattatgaacaatttagatccggtataacattgctgacaaagaagggacagtgttatctcattgacaaagattcatgctcggtacagcttgctatagccctgaggagtcatatgtgggctaggttggcccgcgggagacctatctatgggttggaagtgcctgatgtactggaatcgatgaatggctatcttatcaaacgccatgaggcatgtgctatttgcgagacgggctcaggtcattacgggtggttcttcgtccctgcagggtgccagcttgacgatgtctcgaaagagacttcagctcttcgtgtgccttatgttggatcaaccacagaggaaaggacagatatgaaacttgccttcgttagatctccgagtcgatccctcaaatcagcagtcagaattgctaccgtttattcatgggcctatggggatgatgaaaaatcatggagtgaagcctggacgttggcaaggcagagagccgacatcaccctagatgagttgaggatgatcaccccagtctctacgtcgaccaacctagcccatcggttgagggatcggagcacccaggtgaaatactcggggacatcccttgtgagggttgcgagatacacaaccatttccaatgataatttgtcatttgtgatatctgagaagaaagtagataccaacttcatttaccagcaagggatgctcctcggtcttgggatccttgagaatcttttcaggttggagaccaccacgggggtatccaacacagtgctacatctgcacgtggagacagaatgttgtgttgtacctatgattgatcacccaagaataccaagtctccgtaacattaaggttacgaatgagctatgtacaaaccccttgatctacgacaagtctcctatcatagaacacgatgcaactcgattatactcacaaagccacagaagacatctggtggaatttgttacctggtcaacaagtcagctttaccatatactagctaagtctacagcaatgtccatgattgagttgattacaagattcgagaaggatcacatgaatgaaataaccgccttgattggcgatgacgacatcaacagttttataacagagttcctgctagtagaacctagactgtttatagtctaccttggccagtgtgctgccatcaattgggcttttgatatacattatcatcgcccatcgggcaaataccagatgggggaacttctctactctttactctctaggatgagcaaaggagtatataagatctttaccaatgctctgagccaccccaaagtttacaagaaattttggcgaagcggtgtcatcgagccgattcatggcccgtccttagacacacagaatctacatgtcactgtctgtgatatgatatatggatcctacgtcacatatctggatcttttgctgaatgatgagctagatgattatacgtatctgctctgcgaaagtgatgaggacgtggtcacagacaggtttgataacatccaagccaaacatctctgtgtattggccgatgtgtattgcagctccaaaaggtgtccctcaataatcgggatgtctcctatagaaaaatgtaccatcctcacacattacataaaaggggaatcaatacagtctccgtctggggcctcatggaacactgatcctcttgtagtagatcattactcatgctctctgacataccttcgtcgcggttccatcaaacaaatcaggttgagagtagatcctgggtttgtatttgaggcattgacagacatcgacgtcaaacagcctcgcaaggctaacactggtgtatcggttgtggggttgactgatttctctcctccttgggataacattggtgtttttctaaggactatcaacaccttgagacacaatctgccaatcaccgggaccggagtcttaaactatgaggtccacgcttatcgtagaattggtttgaattcatcagcatgttataaagctgtagagatctccacgttaatcaagccatccttagaagtcggagagcatgggttgttcttaggggaaggttccggttctatgctggctgcgtacaaggaaattcttaaattagccaactgttattacaacagcggagtaatagctgagggcagagccggacaaagagaaatatccccttacccatcagagatgagcctagtagagaatcaaatggggttagaaaagagtgttaaagtgctgttcaacggcaaacctgaagtaacctgggtagggactaccgattgttatgaatatataatcagtaacgtccatacctctagtctgggtttcatacactcggatattgagactctcccaaccaaagatgctgttgagaaattagaagaatttgcctctatcctttccttatccctagttttaggaaaaatcggctctattacagttatcaaagttatgcctattagcggagattttactcagggcttcataagctatgccatccagtattttagggagagcctgcttgtctatccgagatatagtaatttcatttcaactgagtgttaccttattatgataggattgaaagctaatcggttgataaacccagaagccattaagcaaagcataatcagagcagggactagaactgcaccaggactagtgagccatatattatcagagaaacaaaaagggtgtatccaatcttatctgggtgatccttacatccaaggagacttcaataaacaccttaaatctctaacccctatcgagaaaatcctaattaattgtggtctctcggtcaatggcacaaaaatctgtagggatctaatccaccatgatatcgcctctggtccggacggtctgatgagctccacaataatcttatacagggagctggctcatttcaaagacaacgtaagaagtcagcacgggatgttccacccctacccagtactggccagtagcaggcaacgcgaattaattcttcgaatcgccaagaaattctggggttatgtcttgctatattctgatgatccggcactgatcaggcaaacaatcaagaatttgaaaaggaaccacttaacctttgacctacacaataacccgtttattaagggcctgtccagagctgagaaactgctagtgcgtacgagttcactcagaagagagtggttgttcactctcgaaacgaaggaagtgaaagaatggtttaaactggtgggctacagtgcactcgtcagaggttaattagctatacatctgtccccttctcctccaccatgaaattccactggcaatctaatagattaaagaaaactacagatcagataagtatctattcccagctttgtctggt

>KM816619/China/Jilin/2014/GZL

accaaacaaagttgggtaaggatcgatcttataataactatggactggcaaacttaggagtaaagatcctactgtcggggggaggaggaggagcaagatccttgatcatggcgactctccttaaaagcttagcattgttcaaaaggaacaaagacaaagcgccgacagcatcaggttcaggaggggccatccgggggattaagaatgttatcatagtcccgattcccggagactcgtccatcactacccgttcaagactgctcgacaggcttgtcagattggccggagatcctgacatcaacgggtcaaagctgaccggggtgatgatcagcatgttatccttgttcgtagagtcacccgggcaattgatacagcgaatcacagatgatccggatgttagtatccgccttgttgaggtagttcaaagtactaggtcccagtccgggttgacctttgcatcacgtggtgctgatttagacaacgaggcagacatgtatttttcaactgaggggccctcgagtggaggtaagaaaaggatcaactggtttgagaacagagaaataatagacatagaggtgcaggatccagaagagttcaatatgttgttagcctccatactagcacaagtctggatccttctggccaaggctgttacggcaccagatacggcagctgactcagaactgagaaggtgggttaaatacacacaacaaaggagagtgattggggaatttcgccttgacaaggggtggctggacgcagtccgcaacagaattgcagaagatctatcactccggcggttcatggtatctctcatacttgacatcaagaggaccccaggcaacaagccaaggattgcagaaatgatctgcgacattgacaactatattgtcgaagcaggactcgccagtttcatccttactatcaaatttggtatcgaaaccatgtatcctgcattagggctccacgagtttgccggagaattgtccactattgagtccttgatgaacttgtatcaacagctaggagaggttgcaccctacatggtaattctagagaattcaattcagaacaagtttagtgcaggagcttatcccctcctctggagctatgcgatgggtgtcggagtcgagctggagaactcaatggggggcttgaactttggcaggtcatattttgaccctgcctattttcgtctcggacaggagatggtcagaagatctgcaggaaaggtcagctctgtgattgcggctgagctcggcatcacagcagaggaagctaaactagtctcggaaatcgcctcgcaggctggggacgaaagaaccgccagagggactgggcctcgacaggcgcaggtttccttccttcagcaccaaacaggagggggagagtcgtccgcaccagcgaccagagaaggggtcaaagctgcgatcccaaacggagctgaagaaagggacagaaagcaaacacgcccaggaaggcccagaggagagacctccggccaactgctcctggacatcatgccagaggatgagatcttgcgagagtctggtcaaaaccctcgtgaggctcaaagatcggccgaggcactcttcaggctgcaggccatggccaagattctggaggaccaggaggagggagaagacaacagtcaggtctacaacgacaaggatctcctcggctaagcagacgcaccctctgtcgaaatcagtgacaagacatctcctaccagtattataaaaaacttaggacccaggtccaagcaagcacacatcgacactccaaccagtcgagcggagaccaccgatggcagaagaacaagcataccatgtcaacaaggggctggaatgtatcaagtctctcaaagcctcacccccggatctatccaccatcagagataccatcgagagctggagagaggggcttagcccctcgggccgtgcaacaccgaaccctgatacgtccgagggagatcatcagaatatcaaccaatcatgctcaccagcaatcggaccaaacaaagtctacttgtctcctgaagataatctcggatttagagagatcactggcaacgactgtgaggctgggctcggaggggtccagagagaaggatccaactctcaagtacagcgttaccatgtttatagtcacgggggtgaagagattgaaggactcgaggatgctgactctctcgtggttcaagcagatcctccagttgctaacgtcttcaatggaggagaggatggatctgacgacagcgatgtggactctggcccagatgatcccggcagagatactctatatgaccggggatctgttgccggcaatggtctcgctaggtccacagatgtcgaaaaactagaaggtgctgatattcaagaagttcttaactcccagaaaggcaaaggaggaagattccagggcgggaaaaccttgcgagtcccggaaatacccgatgtcaagcactccagaccatcagcccaatcaattaaaaagggcacagacgggaactcagtctcatctggaacggtgatagagtgtttatcgataagtggtgcaacccaaactgtgccagagtcaagatgggagtcatcagagcaaaatgcgtctgtggggagtgtcctcaagtctgcgaggagtgcaaagacgatccaggggtcgacacaagaatctggtaccatagcatcactgactcagcctaaagagaatgactccgagtatgagtatgaggatgacctatttacagagattcaggacatccgtgcaagcattgccaagatccatgatgacaataaaactatcctctcaaagcttgattctatactgttattgaaaggggaagtcgacactatcaagaaacaaatcagtaagcagaatataagtatatccaccattgagggccatctctccagtataatgatagccatccctggctttgggaaggacatcaaggacccaacatccgaagtcgagttgaacccagatctaagacctataataagccgtgactctggcagagctctcgcggaggtcctcaagaaacccgctgtggataggtctcagaaaattggaaccaaagccaactccagctcaaagggtcagcttcttaaggatctccagctaaaacctgtcgacaagcaggcaagctctgcaatcgggtttgtcccgtccgaccatgaatcatccagaaatgtcatccgctccataatcaagtcgagcaagctaaatattgatcacaaggactatcttctagatttactaaatgatgtgaaaggctccaaggatcttaaggaatttcacaagatgctaacagcaattctcgccaagcacccgtaacacatcctccagtcatcatctcatactcgactaaaaacatcctttcaatcaggctattacaaaaaacttaggagcaagggcaactgagcttcgcagacaggatgaccgagatctacgacttcgataaatcagcatgggatgtcaaagggtcaattgcccgcatagaacccaccacctaccacgacggccgactgataccccaggtgagggtcatcgatcctggtctgggagacagaaaagatgagtgctttatgtacctgtttctcctaggagtgattgaggataacgaccccctgtctccccccgtcggaagaacctttggctctctacctctaggggttggtaggtcaactgccaagccagaagaactactaagggaggccacagaattagatatagtggtgaggcgcactgcaggagtaaatgagaaactggtattttacaacaacactccgctgtccttgttaacgccctggaagaaagttctgacaaccggaagtgtgtttagcgctaaccaggtttgcaatgcagtcaacctagtcccgcttgatactccccagagattcagggttgtgtacatgagcataactagattatcagacaatggttactacagtgtgcccagaagaatgctggagttccgctcggccaatgcagtagccttcaacatcttggttacactaagaattgaaaatggcacaaaccctagaagatacatagtcggctcatgggagaattcagaggtcacatttatggtacacgtgggcaactttaggagaaagaagaacgaagtatactctgctgattactgcaaaatgaagattgaaaagatgggtttagtttttgccctgggtggaataggcggaacaagtctccatattagaagcaccgggaaaatgagcaaaaccctccatgcacagctggggttcaagaaaatcttatgttaccccctaatggatgtcaatgaggatcttaaccgatatctctggcgggcagagtgccgaatagtcaaaatccaagctgtcttacagccatcagtaccccaagagttccgtgtctacgatgatgtcatcatcaacgatgatcaaggcttgttcaagatcttgtagttcatttgcaacatcatgacgcgggtcgcaattttgacatttctgtttcttttcccaaatgttgttgcgtgtcagattcactggggcaatctatccaagatcgggattgtaggaacagggagtgccagctacaaggtgatgactaggccaagccaccagactctggttataaagttaatgccaaatataacggccatcgacaattgtacaaagtcagagattgcagagtacaagagattgctgatcacagtgttaaagcctgtagaggatgctctgtcggtgataaccaagaatgtaagaccaattcaaactctaacacctgggcgtagaacccgccgttttgctggagctgttctggccggggtagcacttggagttgcgacagccgctcagataactgcaggagtcgcccttcatcaatcattgatgaactcccaagcaattgagagtttaaaaaccagtcttgagatgtcgaatcaggcaatagaagaaatcagacttgcaaataaggagaccatactggcagtacagggcgtccaggattatatcaacaatgagctcgtcccttctgttcatagaatgtcatgcgagctggtaggtcacaagctcggcctcaagctccttaggtactacaccgagatcctgtccatattcgggcccagtcttcgagacccgatatctgccgaaatatcaatccaggcacttagttatgcattaggcggagacattaataaaatcctggacaagcttgggtatagcggtggggatttccttgccatcctagaaagcaagggaataaaggcccgggttacatatgtggacacaagagattactttataatccttagcatcgcctacccaaccttatctgagatcaagggagtgatagttcacaagatagaagctataacatacaacattggggcacaggagtggtatactactatccctaaatatgtagccactcaggggtatctgatatcgaactttgatgagacgtcatgcgtattcactccagaggggacagtttgcagccagaatgcgttgtacccaatgagcccattgcttcaggaatgtttcagggggtcaacaaaatcgtgcgccagaaccttagtttcagggaccataagtaatagatttatcctatcaaaagggaacctgattgcaaattgtgcgtcagttttgtgcaaatgttacacaacagagacagttatcagccaagatcctgacaaactactaactgttgtagcatccgacaagtgtcctgtagttgaggtggatggagtgacaatacaggtcggcagtcgagagtatccggattctgtatacttacacaaaatagacttaggtccagccatctccctagaaaaactggatgtaggcaccaatttaggcaatgcagtcacaagactggagaatgcaaaggagctcctagatgcatcagaccaaatactgaagactgttaaaggggtacctttcagtgggaatatgtacatagcactggcagcttgcataggagtatccctgggccttgtcacattaatatgctgctgtaaggggaggtgtaagaacaaggaaatccctatctccaaaatcaacccagggctcaaacccgacctgaccgggacctcaaagtcgtacgtaagatcactgtagtcagaatcacctggatcatctggcatcacacacatacatgcacgacacaggcagtccgaggacgcaagaaacccagcctccggtcacccacccgaccccactccacgctccaccacacattagtcatcaaacaaaacttaggacgaaaggtcaatcaccatgtccgcacaaagggagaggatcaatgccttctacaaagacaatcctcacaataagaaccatagggtgatcctggatagagaacgcttggtcattgaaagaccctacatcttgcttggagtcctgctggtaatgttcctgagtctaatcggactgctggccattgcagggatcaggcttcaccgggccaccgttggaacttcagagatccagagtcggctgaataccaatattaagttgaccgaatctattgatcaccagactaaggatgtcttaactcccctttttaaaatcattggcgatgaagtcggcatcagaattccacagaaattcagtgatcttgtcaagttcatctccgataagattaaattcctcaaccctgatagagagtatgatttcagagatctccggtggtgtatgaatccccccgagagagtcaaaattaattttgatcagttttgtgagtacaaggctgcggttaagtcaattgaacatatatttgagtcaccactcaacaagtcaaaaaagctgcaatctttgactctcgggcccggaacaggctgtctaggcaggacagtaacaagagcccatttctcagaacttacaatgaccttaatggacctggatctagagatgaagcacaacgtgtcctcagtgtttaccgtagttgaagagggattattcggaagaacatataccgtctggagatccgatgccagggatccgagcaccgatctaggtatcggccattttttaagagtcttcgagattggactggtaagagatcttgggctgggtccccctgtttttcatatgaccaactatctcacggtgaacatgagtgatgactatcggagatgtcttttagcggtaggggagttgaagttgacagccctatgcacctcatctgagactgtgacactgagtgagagaggagttccaaggagggaacctcttgtggttgtgatacttaatctagctggacccactctagggggagagctatacagtgtctcgcctacctctgatctcatggtggagaaactctatctgtcttcacatagagggatcatcaaagatgatgaggccaattgggtagtgccgtctaccgatgttcgtgatcttcagaataaaggtgaatgtctggtggaagcatgcaagactcgacctccttcattttgcaatggcacaggatcaggcccgtggtcagaagggagaatccctgcctacggggtgatcagggtcagtcttgacttagcgagtgacccggatgtagttatcacttcagtgtttggcccactgatacctcacctatccggcatggatctttacaacaacccgttttcaaaagctgtatggttggctgtaccaccttatgagcagtcatttctaggaatgataaatacaattggattccctaacagagcagaggttatgccgcacattttgaccacagagatcagaggccctcggggtcgttgccatgttcccatagaattgtcccgccgggttgatgacgatatcaagatcgggtccaacatggtcatattgccgacgatggacctgaggtatattacagccacttatgatgtttccaggagcgagcatgcaatcgtgtactatatctatgacacgagtcgctcatcatcttacttctacccagttcgactgaatttcaaaggcaatcctctctctttgaggatagagtgtttcccctggcgtcataaggtgtggtgctaccatgattgtcttatatacaacaccataacaggtgaagaggtccatacgagagggctgaccggcatagaggtaacatgcaatccagtctgagcagagctacgaccgtcactcaggcagtcccttgagtcgccaccgagtccaagcagcacagcctgggacaatcaacggcacagcccagccaacaatgttataaaaaacttaggagccagggttgtaggggccatggactctctatcagtcaatcaggtcttgtaccctgaggttcatctagacagccctattgtcacaaacaaactagttgccatccttgagtactcgggtatcgaccataactatgttcttgaagaccagactcttgtcaagaatattaggtataggctggggtgcggtttttcaaatcaaatgatcatcaataacagggggttgggtgaaacagtcaattctaaacttaaaagttacccccataatcgtcatatcatatacccggattgcaataaggagttgttctgtatcaaggatagctgcatatctaagaagctctcggagctattcaagaagggtaattccttgtactctaagataagtcaccaggtactggattgtcttaagagagtcaatgggaaattaggtctgggcacagatcttacccatggtctgaaggagggtatccttgacctagggttgcacatgcatagctctcaatggttcgagactttcctgttctggttcactatcaagacagagatgagatcaatgatcaaagaacagtcccatatatgccacaagaggaggtataacccgacttttgtgtcgggggatgcattcgaggtgctcgtatcgcgagacctcgttgtgataattgataagaatacccagtatgtcttctacttaacatttgagctggtccttatgtattgtgatgtcatagagggtagacttatgacggagacagccatggccatagaccagagatattcagaacttctaagccgggtcagatacttgtgggatcttattgatgggttcttcccaacactaggcaacaccacataccaaattgttgctctgcttgaaccattatcgttggcttatcttcaacttcaggatgtcactctggagttaagaggtgcttttttagaccattgcttcaaagaactctatgagatactggaacattgcggcattgacacagcaggcacttacaattccatcactgaaggattggattacgtattcatcacccacgatatacatctaactggggagattttttcatttttccggagtttcggacacccccgtctagaagcagtcaccgctgcggaaaatgtcagaaaacatatgaaccaaccgaaggtaatcagttatgagactatgatgaaaggccatgcggtattttgcgggataatcataaatggttttcgggaccgacatggcggcagctggccccctgttgcactgccagaacatgcctctgctgcgatccggaatgcgcaggcatcaggtgagggactgacccatgacctgtgtatagacaactggaaatcctttgttggattcaaatttggctgctttatgccactcagcctagatagtgatttgaccatgtatctcaaagacaaggcattggctgcactgaagaatgagtgggattcagtttacccgaaagaatacctccgttataacccacctagagggacagagtcaaggcgattggtagaggtattcctgaatgactccagctttgatccttataacatgataatgtacgtggtgaatggctcctaccttaaagaccctgagtttaacctctcatacagcctaaaagagaaggagataaaggagacagggcggttgtttgccaaaatgacctataagatgcgggcctgtcaggtaatcgctgaaaatctgatatcgaatggtgttgggaagtatttccgagacaatgggatggcaaaggacgagcatgacctaacaaaagcccttcacaccctggcagtctcaggtgttcccaaaaacaacaaagataaccaccgaggtgggcctcccagaaggacaacaaaccgaggggtgagatcaagccaaggcaccaaaacacaagatagagacaaggttcaagggggacctatgtacaactatttgcgatgccaaccgatcagccctgatcagggtgagtcatacgagactgttagtgcattcatcaccgctgaccttaagaagtattgcctgaattggagatacgagacaatcagcatatttgcacagaggctgaatgaaatatatggactaccatccttctttcaatggttacacaggatattggaaaaatccgtactctacgtcagtgacccacattgtcctcccgatctagataatcatatccctctggacagtgtccccaatgcccaaatattcatcaagtacccaatgggcggaatagaaggttattgccaaaaactatggacaatcagcactataccatacttgtatctggcagcctatgagagcggagtaagaatcgcctcactggtgcagggtgacaatcagacaatcgcagtgacaaaaagagttccaagttcttggccttactcactaaaaaagagggaggcctccaaggccgctcaaaattacttcatagtcttaaggcaaagattgcacgatgtaggtcatcacttaaaggctaatgagaccatagtatcttctcacttttttgtatattccaaagggatttattatgacggcctgctagtctcacaatcactaaagagcatcgccagatgtgtcttctggtccgagactatagtggatgaaaccagagcggcgtgcagtaatattgcaacaactgtcgctaagagtatagagaggggttatgataggtaccttgcatattctttgaatatcctcaagattttccaacagatccttatatcccttaacttcactattaacacaacaatgactcaggatgtcgtggcaccgatcatcgagaacggtgatttgctgataaggatggcactcctgccagcacccatcgggggtctcaattatcttaacatgagcaggttatttgtgagaaatatcggtgacccggtcacctcctctatagccgacctgaagaggatgatagatgccgggctaatgccagaagaaacattgcatcaagtgatgacccagaccccgggagaatcatcctacctagattgggcaagtgacccttattctgccaacctaccctgcgtacagagtatcactcgccttctcaaaaacatcactgcacgatatattttaatcagcagcccaaacccgatgctgaaagggttatttcatgaggggagtagagatgaagacgaggagcttgcaagtttcctaatggatcggcatataattgttccaagagctgcacatgaaatcttagaccatagtataaccggagcaagagaagctatagccgggatgttggacaccaccaagggtctgattagaacaagtatgaaacggggcggcctcacccctcgagtcttagcccgcctttccaattatgattatgaacaattccgatccgggataacactattaacaaagaaagggcagtgttatctcattgacaaggactcgtgctcggtgcagctcgctatagctctgaggggccacatgtgggctaagttggctcgcgggagaccaatctatgggttggaggtgcctgatgtattagaatcgatgaacggctaccttatcaaacgccacgagtcgtgtgctatctgtgaaacgggctcaagtcactacggatggtttttcgtccctgcagggtgccagcttgacgatgtatctagagagacttcggctcttcgtgtaccttatgtcggatcaacaactgaggaaaggacagacatgaaacttgcatttgttaggtctccaagtagatccctcaaatcagcagttagaattgcaacagtttactcatgggcttacggggatgatgagaaatcatggagtgaagcctggatgctagctaggcagagagccaatatcaccttagatgaattgagaatgatcactccagtctccacatccaccaacttagcccaccggttgagggatcggagtacccaggtgaaatactcggggacatcccttgtgagggttgcaagatacacaaccatctccaatgataacttgtcgtttgtgatatctgagaaaaaagtggataccaacttcatttatcagcaagggatgttgcttggtcttgggatccttgagaacctctttaggctagaggccaccaccggagtatccaacacagtgctacacctgcacgtggaaacagaatgttgtgttgtacctatggtggatcacccaaggataccgagtctccgtaatataaaagttacggatgagctatgcacaaaccctttgatctacgacaggtcccccatcatagagcacgatgcaacccggctatattcacagagccacaggagacatttggtggagtttgttacctggtcaacaagccagctttaccatatactggctaagtctacagcaatgtccatgattgaattgatcacgagattcgagaaagatcacatgaatgaaatagccgccctgattggcgatgacgacatcaacagcttcatcacagaatttttgctagtggagcctagattatttatagtttaccttggtcagtgtgctgccatcaattgggcctttgatatacattatcatcggccctcgggcaagtaccagatgggggaacttctctactctctgctctctcggatgagcaaaggagtatataagatctttactaatgctttgagccaccccaaagtttacaagaaattttggcgaagtggcatcattgagccgattcatggcccatccttggatacacagaatttacatgtcactgtctgtgacatgatatatgggtcctatgtcacctatttggatcttttgctgaatgatgagctagatgattacccgtatttgctctgcgagagtgatgaggacgtggtcacagacaggttcgacaacattcaagccaaacatctctgtgtattggccgatgtatattgcagctccaagaggtgtccttcaataatcgggatgtctcctatagaaaaatgtaccattctcacacattacatcaagggagaatcagtacaatccccgtctgggacctcatggaacactgatccccttgtagtagatcattactcatgctctctgacctaccttcgccgcggttccatcaaacaaatcaggttgagggtggatcctgggtttgtattcgaggcgttgacagacgtcgactttaaacaacctcgcaaagctaagttagatatatcggtcgttgggttgactgatttctctcccccttacgacaacgtcggtgattttttagggactatcaacacattgaggcacgatctgcctgtcaccggaaccggggtctcgaactatgaagtccacgcttatcgtagaattggcctgaattcatcagcatgttataaagccgtagagatctccacgttaatcaagccatccttagaagtcggagagcatggattgttcttaggagaaggttccggttcaatgctggctgcgtacaaagaagttcttaaattagcaaattgttactacaacagcggagtaacagcggagggcagagccggacagagggaaatatctccctatccttcagagatgagcctagtagagaatcaaatggggatagagcggagtgttaaagtgctgttcaacgggaaacctgaagtaacttgggtggggaccacagattgctacaagtacataatcagtaacatccaaacctctagtctgggtttcatacattcagatattgagacgctcccaaccaaagatgctgttgagaaattggaggaatttgcctctatcctatccttatccttgattttggggaaaatcggctctattacagttgtcaaaattatgcccattagcggggattttacccaaggcttcatagcctatgccatccaatattttagggagagcctgcttgcctatccgagatatagtaacttcatctcgactgagtgttaccttattatgataggattaaaggccaatcgattgataaacccagaagccattaagcaaagcataatcagagcgggggtcaggactgcaccaggacttgtgagccatgtattatcagagaaacaaaaaggttgtattcaatctttcttgggtgatccttatatccaaggagacttcaataagcaccttaaatctctaacctctattgagaaaatcctggtaaattgtggtctctcgatcaatggcataaaaatctgtagagatctaatccaccatgatatcgcctccggtccagacggccttatgagctctacaattattttatatagggaattggctcatttcaaagataatataaggagtcagcacggaatgttccacccctacccggtactggccaacagcaggcaacgtgaattaatccttcgaatagccaagaaattctgggggtatgtcttgctatattctgatgacccggcactaatcagacaaacaatcaagaacttgaaacggaatcatctaacctttgacttacatagtaacccatttattaaagggctatccaaggctgagaaactgctggtgcggacgagttcactaagaagagaatggttgttcactctcgaaacgaaagaagtaaaagagtggttcaaattggtaggttacagtgcactcatcagaggctagtcagctatgcatctgcccccttctcctccgccatgagaccccactggcgatccagaagattaaagaaaactacatattggataagtatctattcccagctttgtctggt

>KP260624/China/2014

accaaacaaagttgggtaaggatagatcttataataactatggactggcaaacttaggagtaaagatcctactgtcggggggaggaggaggagcaagatccttgatcatggcgactctccttaaaagcttagcattgttcaaaaggaacaaagacaaagcgccgacagcatcaggttcaggaggggccatccgggggattaagaatgttatcatagtcccgattcccggagactcgtccatcattacccgttcaagactgctcgacaggcttgtcagattggccggagatcctgacatcaacgggtcaaagctgaccggggtgatgatcagcatgttatccttgttcgtagagtcacccgggcaattgatacagcgaatcacagatgatccggatgttagtatccgccttgttgaggtagttcaaagtactaggtcccagtccgggttgacctttgcatcacgtggtgctgatttagacaacgaggcagacatgtatttttcaactgaggggccctcgagtggaggtaagaaaaggatcaactggtttgagaacagagaaataatagacatagaggtgcaggatccagaagagttcaatatgttgttagcctccatactagcacaagtctggatccttctggccaaggctgttacggcaccagatacggcagctgactcagaactgagaaggtgggttaaatacacacaacaaaggagagtgattggggaatttcgccttgacaaggggtggctggacgcagtccgcaacagaattgcagaagatctatcactccggcggttcatggtatctctcatacttgacatcaagaggaccccaggcaacaagccaaggattgcagaaatgatctgcgacattgacaactatattgtcgaagcaggactcgccagtttcatccttactatcaagtttggtatcgaaaccatgtatcctgcattagggctccacgagtttgccggagaattgtccactattgaatccttgatgaacttgtatcaacagctaggagaggttgcaccctacatggtaattctagagaattcaattcagaacaagtttagtgcaggagcttatcccctcctctggagctatgcgatgggtgtcggagtcgagctggagaactcaatggggggcttgaactttggcaggtcatattttgaccctgcctattttcgtctcggacaggagatggtcagaagatctgcaggaaaggtcagctctgtgattgcggccgagctcggcatcacagcagaggaagctaaactagtctcggaaatcgcctcgcaggctggggacgaaagaaccgctagagggactgggcctcgacaggcgcaggtctccttccttcagcacaaaacaggagagggagagtcgtccgcaccagcaaccagagagggggtcaaagctgcgatcccaaacggagccgaagaaagggacaggaagcaaacacgctcaggaaggcccagaggagagacctccggccaactgctcctggacatcatgccagaggatgagatcttgcgagagtctggtcaaaaccctcgtgaggctcaaagatcggccgaggcactcttcaggctgcaggccatggccaagattctggaggaccaggaggagggagaagacaacagtcaggtctacaacgacaaggatctcctcggctaagcagacgcaccctctgtcgaaatcagtgacaagacatctcctaccagtattataaaaaacttaggacccaggtccaagcaagcacacatcgacactccaaccattcgagcggagaccaccgatggcagaagaacaagcataccatgtcaacaaggggctggaatgtatcaagtctctcaaagcctcacccccggatctatccaccatcagagataccatcgagagctggagagaggggcttagcccctcgggccgtgcaacaccgaaccctgatacgtccgagggagatcatcagaatatcaaccaatcatgctcaccagcaatcggaccaaacaaagtctacttgtctcctgaagataatctcggatttagagagatcactggcaacgactgtgaggctgggctcggaggggtccagagagaaggatccaactctcaagtacagcgttaccatgtttatagtcacgggggtgaagagattgaaggactcgaggatgctgactctctcgtggttcaagcagatcctccagttgctaacgtcttcaatggaggagaggatggatctgacgacagcgatgtggactctggcccagatgatcccggcagagatactctatatgaccggggatctgttgccggcaatggcatcgctaggtccacagatgtcgaaaaactagaaggtgctgatattcaagaagttcttaactcccagaaaggcaaaggaggaagattcaagggcgggaaaaccttgcgagtcccggaaatacccgatgtcaagcactccagaccatcagcccaatcaattaaaaagggcacagacgggaactcagtctcatctggaacggtgatagagtgtttatcgataagtggtgcaacccaaactgtgccagagtcaagatgggagtcatcagagcaaaatgcgtctgtggggagtgtcctcaagtctgcgaggagtgcaaagacgatccaggggtcgacacaagaatctggtaccatagcatcactgactcagcctaaagagaatgactccgagtatgagtatgaggatgacctatttacagagattcaggacatccgtgcaagcattgccaagatccatgatgacaataaaactatcctctcaaagcttgattctatactgttattgaaaggggaagtcgacactatcaagaaacaaatcagcaagcagaatataagtatatccaccattgagggccatctctccagtataatgatagccatccctggctttgggaaggacatcaaggacccaacatccgaagtcgagttgaacccagatttaagacctataataagccgtgactctggcagagctctcgcggaggtcctcaagaaacccgctgtggataggtctcagaaaattggaaccaaagccaactccagctcaaagggtcagcttcttaaggatctccagctaaaacctgtcgacaagcaggcaagctctgcaatcgggtttgtcccgtccgaccatgaatcatccagaaatgtcatccgctccataatcaagtcgagcaagctaaatattgatcacaaggactatcttctagatttactaaatgatgtgaaaggctccaaggatcttaaggaatttcacaagatgctaacagcaattctcgccaagcacccgtaacacatcctccagtcatcatctcatactcgactaaaaacatcctttcaatcaggctattacaaaaaacttaggagcaagggcaactgagcttcgcagacaggatgaccgagatctacgacttcgataaatcagcatgggatgtcaaagggtcaattgcccgcatagaacccaccacctaccacgacggccgactgataccccaggtgagggtcatcgatcctggtctgggagacagaaaagatgagtgctttatgtacctgtttctcctaggagtgattgaggataacgaccccctgtctccccccgtcggaagaacctttggctctctacctctaggggttggtaggtcaactgccaagccagaagaactactaagggaggccacagaattagatatagtggtgaggcgcactgcaggagtaaatgagaaactggtattttacaacaacactccgctgtccttgttaacgccctggaagaaagttctgacaaccggaagtgtgtttagcgctaaccaggtttgcaatgcagtcaacctagtcccgcttgatactccccagagattcagggttgtgtacatgagcataactagattatcagacaatggttactatagtgtgcccagaagaatgctggagttccgctcggccaatgcagtagccttcaacatcttggttacactaagaattgaaaatggcacaaaccctagaagatacatagtcggctcatgggagaattcagaggtcacatttatggtacacgtgggcaactttaggagaaagaagaacgaagtatactctgctgattactgcaaaatgaagattgaaaagatgggtttagtttttgccctgggtggaataggcggaacaagtctccatattagaagcaccgggaaaatgagcaaaaccctccatgcacagctggggttcaagaaaatcttatgttaccccctaatggatgtcaatgaggatcttaaccgatatctctggcgggcagagtgccgaatagtcaaaatccaagctgtcttacagccatcagtaccccaagagttccgtgtctacgatgatgtcatcatcaacgatgatcaaggcttgttcaagatcttgtagttcatttgcaacatcatgacgcgggtcgcaattttgacatttctgtttcttttcccaaatgttgttgcgtgtcagattcactggggcaatctatccaagatcgggattgtaggaacagggagtgccagctacaaggtgatgactaggccaagccaccagactctggttataaagttaatgccaaatataacggccatcgacaattgtacaaagtcagagattgcagagtacaagagattgctgatcacagtgttaaagcctgtagaggatgctctgtcggtgataaccaagaatgtaagaccaattcaaactctaacacctgggcgtagaacccgccgttttgctggagctgttctggccggggtagcacttggagttgcgacagccgctcagataactgcaggagtcgcccttcatcaatcattgatgaactcccaagcaattgagagtttaaaaaccagtcttgagatgtcgaatcaggcaatagaagaaatcagacttgcaaataaggagaccatactggcagtacagggcgtccaggattatatcaacaatgagctcgtcccttctgttcatagaatgtcatgcgagctggtaggtcacaagctcggcctcaagctccttaggtactacaccgagatcctgtccatattcgggcccagtcttcgagacccgatatctgccgaaatatcaatccaggcacttagttatgcattaggcggagacattaataaaatcctggacaagcttgggtatagcggtggggatttccttgccatcctagaaagcaagggaataaaggcccgggttacatatgtggacacaagagattactttataatccttagcatcgcctacccaaccttatctgagatcaagggagtgatagttcacaagatagaagctataacatacaacattggggcacaggagtggtatactactatccctaaatatgtagccactcaggggtatctgatatcgaactttgatgagacgtcatgcgtattcactccagaggggacagtttgcagccagaatgcgttgtacccaatgagcccattgcttcaggaatgtttcagggggtcaacaaaatcgtgcgccagaaccttagtttcagggaccataagtaatagatttatcctatcaaaagggaacctgattgcaaattgtgcgtcagttttgtgcaaatgttacacaacagagacagttatcagccaagatcctgacaaactattaactgttgtagcatccgacaagtgtcctgtagttgaggtggatggagtgacaatacaggtcggcagtcgagagtatccggattctgtatacttacacaaaatagatttaggtccagcaatctccctagaaaaactggatgtaggcaccaatttaggcaatgcagtcacaagactggagaatgcaaaggagctcctagatgcatcagaccaaatactgaagactgttaaaggggtacctttcagtgggaatatgtacatagcactggcagcttgcataggagtatccctgggccttgtcacattaatatgctgctgtaaggggaggtgtaagaacaaggaaatccctatctccaaaatcaacccagggctcaaacccgacctgaccgggacctcaaagtcgtacgtaagatcactgtagtcagaatcacctggatcatctggcatcacacacatacatgcacgacacaggcagtccgaggacgcaagaaacccagcctccggtcacccacccgaccccactccacgctccaccacacattagtcatcaaacaaaacttaggacgaaaggtcaatcaccatgtccgcacaaagggagagaatcaatgccttctacaaagacaatcctcacaataagaaccatagggtgatcctggatagagaacgcttggtcattgaaagaccctacatcttgcttggagtcctgctggtaatgttcctgagtctaatcggactgctggccattgcagggatcaggcttcaccgggccaccgttggaacttcagagatccagagtcggctgaataccaatattaagttgaccgaatctattgatcaccagactaaggatgtcttaactcccctttttaaaatcattggcgatgaagtcggcatcagaattccacagaaattcagtgatcttgtcaagttcatctccgataagattaaattcctcaaccctgatagagagtatgatttcagagatctccggtggtgtatgaatccccccgagagagtcaaaattaattttgatcagttttgtgagtacaaggctgcggttaagtcaattgaacatatatttgagtcaccactcaacaagtcaaaaaagctgcaatctttgactctcgggcccggaacaggctgtctaggcaggacagtaacaagagcccatttctcagaacttacaatgaccttaatggacctggatctagagatgaagcacaacgtgtcctcagtgtttaccgtagttgaagagggattattcggaagaacatataccgtctggagatccgatgccagggatccgagcaccgatctaggtatcggccattttttaagagtcttcgagattggactggtaagtgatcttgggctgggtccccctgtttttcatatgaccaactatctcacggtgaacatgagtgatgactatcggagatgtcttttagcggtaggggagttgaagttgacagccctatgcacctcatctgagactgtgacactgagtgagagaggagttccaaggagggaacctcttgtggttgtgatacttaatctagctggacccactctagggggagagctatacagtgtcttgcctacctctgatctcatggtggagaaactctatttgtcttcacatagagggatcatcaaagatgatgaggccaattgggtagtgccgtctaccgatgttcgtgatcttcagaacaaaggtgaatgtctggtggaagcatgcaagactcgacctccttcattttgcaatggcacaggatcaggcccgtggtcagaagggagaatccctgcctacggggtgatcagggtcagtcttgacttagcgagtgacccggatgtagttatcacttcagtgtttggcccactgatacctcacctatccggcatggatctttacaacaacccgttttcaaaagctgtatggttggctgtaccaccttatgagcagtcatttctaggaatgataaatacaattggattccctaacagagcagaggttatgccgcacattttgaccacagagatcagaggccctcggggtcgttgccatgttcccatagaattgtcccgccgggtggatgacgatatcaagatcgggtccaacatggtcatattgccgacgatggacctgaggtatattacagccacttatgatgtttccaggagcgagcatgcaatcgtgtactatatctatgacacgagtcgctcatcatcttacttctacccagttcgactgaatttcaaaggcaatcctctctctttgaggatagagtgtttcccctggcgtcataaggtgtggtgctaccatgattgtcttatatacaacaccataacaggtgaagaggtccatacgagagggctgaccggcatagaggtaacatgcaatccagtctgagcagagctacgaccgtcactcaggcagtcccttgagtcgccaccgagtccaagcagcacagcctgggacaatcaacggcacagcccagccaacaatgttataaaaaacttaggagccagggttgtaggggccatggactctctatcagtcaatcaggtcttgtaccctgaggttcatctagacagccctattgtcacaaacaaactagttgccatccttgagtactcgggtatcgaccataactatgttcttgaagaccagactcttgtcaagaatattaggtataggctggggtgcggtttttcaaatcaaatgatcatcaataacaggggggtgggtgaaacagtcaattctaaacttaaaagttacccccataatcgtcatatcatatacccggattgcaataaggagttgttttgtatcaaggatagctgcatatctaagaagctctcggagctattcaagaagggtaattccttgtactctaagataagtcaccaggtactggattgtcttaagagagtcaatgggaaattaggtctgggcacagatcttacccatggtctgaaggagggtatccttgacctagggttgcacatgcatagctctcaatggttcgagactttcctgttctggttcactatcaagacagagatgagatcaatgatcaaagaacagtcccatatatgccacaagaggaggtataacccgacttttgtgtcgggggatgcattcgaggtgctcgtatcgcgagacctcgttgtgataattgataagaatacccagtatgtcttctacttaacatttgagctagtccttatgtattgtgatgtcatagagggtagacttatgacggagacagccatggccatagaccagagatattcagaacttctaagccgggtcagatacttgtgggatcttattgatgggttcttcccaacactaggcaacaccacataccaaattgttgctctgcttgaaccattatcgttggcttatcttcaacttcaggatgtcactctggagttaagaggtgcttttttagaccattgcttcaaagaactctatgagatactggaacattgcggcattgacacagcaggcacttacaattccatcactgaaggattggattacgtattcatcacccacgatatacatctaactggggagattttttcatttttccggagtttcggacacccccgtctagaagcagtcaccgctgcggaaaatgtcagaaaacatatgaaccaaccgaaggtaatcagttatgagactatgatgaaaggccatgcggtattttgcgggataatcataaatggttttcgggaccgacatggcggcagctggccccctgttgcactgccagaacatgcctctgctgcgatccggaatgcgcaggcatcaggtgagggactgacccatgacctgtgtatagacaactggaaatcctttgttggattcaaatttggctgctttatgccactcagcctagatagtgatttgaccatgtatctcaaagacaaggcattggctgcactgaagaatgagtgggattcagtttacccgaaagaatacctccgttataacccacctagagggacagagtcaaggcgattggtagaggtattcctgaatgactccagctttgatccttataacatgataatgtacgtggtgaatggctcctaccttaaagaccctgagtttaacctctcatacagcctaaaagagaaggagataaaggagacagggcggttgtttgccaaaatgacctataagatgcgggcctgtcaggtaatcgctgaaaatctgatatcgaatggtgttgggaagtatttccgagacaatgggatggcaaaagacgagcatgacctaacaaaagcccttcacaccctggcagtctcaggtgttcccaaaaacaacaaagataaccaccgaggtgggcctcccagaaggacaacaaaccgaggggtgagatcaagccaaggcaccaaaacacaagatagagacaaggttcaagggggacctatgtacaactatttgcgatgccaaccgatcagccctgatcagggtgagtcatacgagactgttagtgcattcatcaccgctgaccttaagaagtattgcctgaattggagatacgagacaatcagcatatttgcacagaggctgaatgaaatatatggactaccatccttctttcaatggttacacaggatattggaaaaatccgtactctacgtcagtgacccacattgccctcccgacctagataataagggcactctgcagagtgtccccaatgcccaaatattcatcaagtacccaatgggcggaatagaaggttattgccaaaaactatggacaatcagcactataccatacttgtatctggcagcctatgagagcggagtaagaatcgcctcactggtgcagggtgacaatcagacaatcgcagtgacaaaaagagttccaagttcttggccttattcactaaaaaagagggaggcctccaaggccgctcaaaattacttcgtagtcttaaggcaaagattgcacgatgtaggtcatcacttaaaggctaatgagaccatagtatcttctcacttttttgtatattccaaagggatttattatgacggcctgctagtctcacaatcactaaagagcatcgccagatgtgtcttctggtccgagactatagtggatgaaaccagagcggcgtgcagtaatattgcaacaactgtcgctaagagtatagagaggggttatgataggtaccttgcatattctttgaatatcctcaagattttccaacagatccttatatcccttaacttcactattaacacaacaatgactcaggatgtcgtggcaccgatcatcgagaacggtgatttgctgataaggatggcactcttgccagcacccatcgggggtctcaattatcttaacatgagcaggttatttgtgagaaatatcggtgacccggtcacctcctctatagccgacctgaagaggatgatagatgccgggctaatgccagaagaaacattgcatcaagtgatgacccagaccccgggagaatcatcctacctagattgggcaagtgacccttattctgccaacctaccctgcgtacagagtatcactcgccttctcaaaaacatcactgcacgatatattttaatcagcagcccaaacccgatgctgaaagggttatttcatgaggggagtagagatgaagacgaggagcttgcaagtttcctaatggatcggcatataattgttccaagagctgcacatgaaatcttagaccatagtataaccggagcaagagaagctatagccgggatgttggacaccaccaagggtctgattagaacaagtatgaaacggggcggcctcacccctcgagtcttagcccgcctttccaattatgattatgaacaattccgatccgggataacactattaacaaagaaagggcagtgttatctcattgacaaggactcgtgctcggtgcagctcgctatagctctgaggggccacatgtgggctaagttggctcgcgggagaccaatctatgggttggaggtgcctgatgtattagaatcgatgaacggctaccttatcaaacgccacgagtcgtgtgctatctgtgaaacgggctcaagtcactacggatggtttttcgtccctgcagggtgccagcttgacgatgtatctagagagacttcggctcttcgtgtaccttatgtcggatcaacaactgaggaaaggacagacatgaaacttgcatttgttaggtctccaagtagatccctcaaatcagcagttagaattgcaacagtttactcatgggcttacggggatgatgagaaatcatggagtgaagcctggatgctagctaggcagagagccaatatcaccttagatgaattgagaatgatcactccagtctccacatccaccaacttagcccaccggttgagggatcggagtacccaggtgaaatactcggggacatcccttgtgagggttacaagatacacaaccatctccaatgataacttgtcgtttgtgatatctgagaaaaaagtggataccaacttcatttatcagcaagggatgttgcttggtcttgggatccttgagaacctctttaggctagaggccaccaccggagtatccaacacagtgctacacctgcacgtggaaacagaatgttgtgttgtacctatggtggatcacccaaggataccgagtctccgtaatataaaagttacggatgagctatgcacaaaccctttgatctacgacaggtcccccatcatagagcacgatgcaacccggctatattcacagagccacaggagacatttggtggagtttgttacctggtcaacaagccagctttaccatatactggctaagtctacagcaatgtccatgattgaattgatcacgagattcgagaaagatcacatgaatgaaatagccgccctgattggcgatgacgacatcaacagcttcatcacagaatttttgctagtggagcctagattatttatagtttaccttggtcagtgtgctgccatcaattgggcctttgatatacattatcatcggccctcgggcaagtaccagatgggggaacttctctactctctgctctctcggatgagcaaaggagtatataagatctttactaatgctttgagccaccccaaagtttacaagaaattttggcgaagtggcatcattgagccgattcatggcccatccttggatacacagaatttacatgtcactgtctgtgacatgatatatgggtcctatgtcacctatttggatcttttgctgaatgatgagctagatgattacccgtatttgctctgcgagagtgatgaggacgtggtcacagacaggttcgacaacattcaagccaaacatctctgtgtattggccgatgtatattgcagctccaagaggtgtccttcaataatcgggatgtctcctatagaaaaatgtaccattctcacacattacatcaagggagaatcagtacaatccccgtctgggacctcatggaacactgatccccttgtagtagatcattactcatgctctctgacctaccttcgccgcggttccatcaaacaaatcaggttgagggtggatcctgggtttgtattcgaggcgttgacagacgtcgactttaaacaacctcgcaaagctaagttagatatatcggtcgttgggttgactgatttctctcccccttacgacaacgtcggtgattttttagggactatcaacacattgaggcacgatctgcctgtcaccggaaccggggtctcgaactatgaagtccacgcttatcgtagaattggcctgaattcatcagcatgttataaagccgtagagatctccacgttaatcaagccatccttagaagtcggagagcatggattgttcttaggagaaggttccggttcaatgctggctgcgtacaaagaagttcttaaattagcaaattgttactacaacagcggagtaacagcggagggcagagccggacagagggaaatatctccctatccttcagagatgagcctagtagagaatcaaatggggatagagcggagtgttaaagtgctgttcaacgggaaacctgaagtaacttgggtggggaccacagattgctacaagtacataatcagtaacatccaaacctctagtctgggtttcatacattcagatattgagacgctcccaaccaaagatgctgttgagaaattggaggaatttgcctctatcctatccttatccttgattttggggaaaatcggctctattacagttgtcaaaattatgcccattagcggggattttacccaaggcttcatagcctatgccatccaatattttagggagagcctgcttgcctatccgagatatagtaacttcatctcgactgagtgttaccttattatgataggattaaaggccaatcgattgataaacccagaagccattaagcaaagcataatcagagcgggggtcaggactgcaccaggacttgtgagccatgtattatcagagaaacaaaaaggttgtattcaatctttcttgggtgatccttatatccaaggagacttcaataagcaccttaaatctctaacctctattgagaaaatcctggtaaattgtggtctctcgatcaatggcacaaaaatctgtagtgatctaatccaccatgatatcgcctccggtccagacggtcttatgagctctacaattattttatatagggaattggctcatttcaaagataatataaggagtcagcacggaatgttccacccctacccggtactggccaacagcaggcaacgtgaattaatccttcgaatagccaagaaattctgggggtatgtcttgctatattctgatgacccggcactaatcagacaaacaatcaagaacttgaaacggaatcatctaacctttgacttacatagtaacccatttattaaagggctatccaaggctgagaaactgctggtgcggacgagttcactaagaagagaatggttgttcactctcgaaacgaaagaagtaaaagagtggttcaaattggtaggttacagtgcactcatcagaggctagtcaactatgcatctgcccccttctcctccgccatgagaccccactggcgatccagaagattaaagaaaactacatattggataagtatctattcccagctttgtctggt

>KP789375/Senegal/1969

accaaacaaagttgggtaaggatagttcttataataactatggatcaggaaacttaggagtaaagatcctactgtcggggggaggaggaggagcaagatctttgaccatggcgactctccttaaaagcttggcattgttcaagaggaacaaagataaagcgccgacagcatcaggttcaggaggggccatccgggggattaagaatgttatcatagtcccaattcccggagattcatctatcattacccgttcaagactgcttgacaagcttgtcagattggctggagatcctgacatcaatgggtcaaagctgaccggcgtgatgatcagcatgttatctttgttcgtagagtcacccgggcaattgatacagcggatcacagatgacccagatgtcagtatccgccttgttgaggtggttcaaagtactaggtctcagtcagggttgacctttgcatcacgtggtgctgatttggacaatgaggcagacatgtatttttcaactgaaggaccctcgagtggaagtaagaaaaggatcaactggtttgagaacagagaaataatagacatagaggtccaggatgcagaagagttcaatatgttattagcatccatactagcacaagtttggatcctcctggccaaggcagttacggcaccggatacggcagctgactcagaactgagaaggtgggttaaatacacacaacagaggagagtgattggggaatttcgccttgacaaaggttggctggacgcagtccgcaataggattgcagaagatctatcacttcggcggttcatggtatctctcatacttgacatcaagaggacccccgggaacaagccaaggattgcagaaatgatctgcgacatcgacaactatattgtcgaagccggacttgccagtttcatccttaccatcaagtttggtattgaaaccatgtatcctgcattaggtcttcacgagtttgccggggaattgtccactatagaatccctgatgaacttgtatcaacaactaggggaggttgcaccctacatggtaattctagagaactcaattcagaacaagtttagtgcaggagcttatcccctcctgtggagctatgccatgggtgtcggagttgagctggagaactcaatggggggcctgaactttggcaggtcatattttgacccggcctattttcgtcttggacaggagatggtcagaagatctgcagggaaggtcagctctgtaatcgcggctgagcttggcatcacagcagaggaagctaaactagtctcggaaatcgcctcgcagactggggacgaaaggaccgttagggggactgggccacgacaggcgcaggtttctttcctccagcataaaacaggagagggagagtcatctgcaccagtgaccagagaagaagtcaaggctgcgattccaaatgggtccgaagaaagggacaaaaggcgaacccgctcaggaagacccagaggagagaccccgggccaaccgctcttggaaatcatgccagaggatgaggtctcgcgagaatctggtcaaacccctcgtgaggctcagaggtcggccgaggcactcttcaggctgcaagctatggccaagattctagagggccaggaggagggagaagataacagtcagatctacaacgacaaggatctcctcagttgagcagaagctccctctgtcaacatcagcagcaaaatatcgcccatcagtgttataaaaaacttaggacccaggtccaagcagtcacacatcgaaactctagccaaccgagcagagaccaccgatggcagaggaacaagcataccatgtcaacaagggactggaatgtatcaagtccctcaaaacctctcccccagatctatccaccatcagagatacccttgagagctggagagaggggcttggcccctcggatcgtgcaacaccgaaccctgatgcgtccgagggagaccatcaggatatcaaccaatcatgctcaccagcagtcggatcaggcaaaatcgacgtgtctcctgaaggtaatctcggatttagagagatcacttacgatgacagtgaggcagggctcagagggattcaggacaaaggatccaactctcaagtacagcgttaccatgtttatagccacgggggtgaagagattgaaggactcgaggatgctgactctctcgtggttcaagcagatcctccagttgctaacaccttcagtggaggagaagatggatctgacgacagcgatgtggactctggcccagatgatcccagcagagatcctctatatgactggggatctgttaccggccatggtgtcgctaagtccacagatgtcgaaaaattagaaggtgatgacattcaagcagttcttaactcccagaagaataaaggaggaagattccaaggcgggaaaaccttgcgagtcccggaaatacccgatgtcaagcactccggaccatcagcccaatcaattaaaaagggcacagacgggaactcagtcttatctggaacggcgacagagtgtttattgataaatggtgcaacccgagctgtgccagagtcaagatgggagtcgtcagagcgaaatgcatctgtggagagtgtccccaaatctgcgaggagtgcaaagacgatccaggagttgacacaagaatctggtaccatagcatcactgactcagcctaaagagaatgactccgagtatgagtatgaggatggtctatttacagagatacaggacattcgtgcaagcattgccaagatccatgaagacaataaaactatcctctcaaagcttgaatctatactgttattgaaaggagaaatagatactatcaagaaacaaatcagcaagcagaatataagtatatctaccattgagggccatctctccagtataatgatagcaatcccgggttttgggaaggatatcaaggacccaacatccgaggttgagttgaacccagatttaagacctataatcagccgtgattctggcagagctctcgcggaggtactcaagaaacccgctgttgataggtctcagaaaaatggaatcaaagccaactcaagttcaaagggtcagctccttaaggacctccagctaaaacctgttgacaaacaagcaagctctgcaatagggtttatcccgtctgaccatgagtcatccagaagtgtcatccgctctataatcaagtcgagcaagctaaacattgatcacaaagactaccttctagacttactgaatgatgtgaaaggctccaaggatcttaaggaattccacaaaatgctaacaaccattcttgccaagcaaccgtaacacaacctccaaacaacatcccatactcggctgaacacatcttctcaaccaggctattacaaaaaacttaggagcaagggcaaccgagcttcacagacaagatgaccgagatctacgacttcgataaatcagcatgggatgtcaaagggtcaattgctcccatcgaacccaccacctatcacgacggccgactgataccccaagtgagagtcatcgaccctgggctaggagatagaaaagatgagtgcttcatgtacctgtttcttttaggagtgattgaggataacgaccccctatccccccctgtcggaagaacctttggttctttacctctaggggtcggtaggtcaactgctaagccagaggaactactaagggaggccacggagctagacatagtggtgaggcgcacggcaggattaaacgagaagctggtattttacaacaacactccactgtccttgctaacaccctggaggaaagtcttgacaaccggaagtgtgttcagcgctaaccaggtttgcaatgcagtcaatctggtcccacttgatagtccccagagattcagggtagtgtacatgagcatcactagattgtcagataatggttattacagtgtccccaggagaatgttggagttccgctcagccaatgcagtcgccttcaatatcttggttacactgagaatcgaaaatggcacaaaccctagaagatacatagtcggctcatgggagaatccagaggtcacatttatggtacacgtgggcaactttagaagaaagaagaatgaagtatattctgctgattactgcaaaatgaagattgaaaagatgggtctagtttttgccctgggaggaataggaggaacaagtctccatattagaagcaccgggaaaatgagcaagactctccatgcacagctagggttcaaaaaaatcttatgctaccccctaatggatattaatgaggatcttaaccgatatctttggcgggcagagtgccgaatagtcaaaatccaagccgtcttacagccatcagtaccccaagaattccgtgtctacgatgatgtcatcatcaacgatgaccaaggcttgttcaagatcttgtaattcactcgcaacatcatgacacgggtcgcaaccttgacatttctgtttcttttcccaaataccattgcatgccagattcactggggcaatctatccaagatcgggattgtgggaacagggagtgccagttacaaggtgatgactaggccaagccaccaaactctggttataaagctgatgccaaatataacagccatcgacaattgtacgaaatcagagatttcagagtacaaaagattgctgatcacagtgttaaagcctgtagaggatgctctgtcagtgataaccaagaatgtaaaaccaattcagactctaacgcctgggcgcaggacccgccgttttgccggggctgttctggccggagtggcacttggagtcgcgacggctgctcagataactgccggagtcgcacttcatcaatccttgatgaactcccaagcgattgaaagtttaaagaccagtcttgagaagtcgaatcaggcaattgaagaaataaggcttgcaaataaggagaccatactggcagtacagggtgtccaagactatatcaacagtgaacttgtcccttctgtccataaaatgtcatgtgagctggtaggtcacaaactcagcctcaagctccttaggtattacaccgagatcttgtctatattcgggcctagcctccgagacccgatagctgctgaaatatcaatccaggcactcagttatgcattaggtggagatattaataaaatcctggataagcttgggtatagcggcggggatttccttgctatcctagaaagcaaggggataaaggctcgagtcacatatgtggatacaagagattactttataattctaagcatagcctacccaactttatctgaaatcaagggggtgatagttcataaaatagaagccatatcatacaacattggggcacaggaatggtacactactatccctaaatatgtagccactcaggggtatttgatatcgaattttgatgagacgtcgtgcgtattcactccagaggggacagtctgtagtcagaatgctttgtacccaatgagcccattgcttcaggaatgtttcagggggtcgacaaaatcgtgcgccagaactctagtttcagggactacaagcaatagatttatcctgtcaaaagggaacttgattgcaaattgtgcatcagttttgtgcaagtgttacacaacggagacagttatcagccaagatcctgataaattactaactgttatagcctccgataagtgtcctgtagttgaagtggatggagtgacaatacaggtcggcagtcgtgagtacccagactccgtgtacctacacaaaatagacttaggcccagccatctctctggaaaaactggacgtaggcaccaatttaggtaatgcagttacaagactggagaatgcaaaggagctcttagatgcatcagaccagatattgaagactgttaaaggggtacccttcggtggcaatctatacataggactggcagcttgcatcggagtatccctagggcttgtcacattaatatgctgctgtaaggggagatgtaggaacaaagagattcctacgtccaaaatcaacccagggctcaaacctgacctgaccgggacctcaaagtcatacgtgagatcactgtagtcagaataacccgaatcaccaaacatcacacacatacatgtgtgatacaagcggtcagaggacgcagaagattcaacttccgatcaccaacctggccccgctctatgctccattacacattgatcatcaaacaaaacttaggacgaaaggtcagtcaccatgtccgcacaaagggaaagaatcaatgccttctacaaagacaatcttcataacaagaatcatagggtaatcctggatagagaacgcttgactattgaaagaccctacatcttacttggggtcctgctggtaatgtttctgagcctaatcgggctgctggccattgcagggatcagacttcaccgagccaccgtcggaactgcagagatccagagtcggcttaataccaacattgagttaactgaatctattgatcaccaaaccaagaatgtcctaactccgctgtttaaagtcattggtgatgaagtcggcataagaatcccacaaaaattcagtgatcttgtcaagttcatctccgataagattaagttcctcaaccctgacagagaatatgattttagggatctccggtggtgtatgaatccccccgagagagtcaaaattaattttgatcagttttgtgaatacaaggctgcagataagtcaattgaacatatatttgagtcatcactcaacagatcaagaaggttgcgattgttgacccttgggcctggaacaagctgtctcggtagggcagtaacaagagctcagttctcaaagcttactctgaccctgttggatctggatctcgagatgaaacacaacgtgtcctcagtgttcaccgtagttgaagagggattattcggaagaacgtacactgtctggagatctgatgccgggaatccgagcaccgacccaggtactggccattttttaagagtcttcgagatcgggctggtaagagatctcgggctgagtgctcctgttttccatatgaccaatcatctcacagtgaacatgagcgatggctatcggagctgtcttttggcagtaggggagttgaagctggcagccctatgcaccccatccgagactgtgacactgagtgagagaggagttccaaagagagagcctcttgtggttgtgatacttaacctagccgggcctactctaggtggcgaactatacagtgtcttgcctacctctgacctcacggtagagaaacttcatctatcctcacacagagggattatcaaagataacgaggccaattgggtagtaccgtccaccgatgttcgtgatcttcaaaacaaaggagaatgtttagtggaagcatgtaagactcgacctccttcattttgcaatggcacaggagtgggcccatggtcagaggggagaatccctgcctacggggtgatcagggtcagtcttgacttagccagtgaccctgacgtagttataacttcagtatttggcccattgatacctcacctgtccggcatggatctttacaacaacccgttttcaagcgctgtgtggttggcggtaccaccttatgagcagtcatatctgggaatgataaatacaattggcctcccaaatagagcagaggttatgccgcacattttgaccacagagatcaaggggcctcggggtcgttgtcatgttcccatagaattgtcccgcaggattgatgatgatatcaagattgggtccaacatggttgtattgccgacgaaggatctgaggtacataacagccacttatgatgtttccaggagcgagcatgcaatcgtgtactatatctatgacacaggtcgctcatcatcttacttctacccggctcgattgaagttcaaaggcaatcctctctctctgaggatagagtgtttcccctggcatcataaggtatggtgctaccatgattgtcttatatacaacaccataacaaacgaagaagtccataagagaggactgatcggcatagaggtaacatgtaatccagcctgagcggagctgcgaccatcactcaagcaggcctccgagctaatcccaagcccaggcagcacagctcgggacactcgacaacacaacccagccaacaatgttataaaaaacttaggggccaaggctataggagccatggactccctgtcagtcaatcaggtcttgtaccctgaggtccatctggatagccctattgtcacaaacaagctggttgccatcctcgagtactcaggcattgaccacaactatgtccttgaagaccagacccttatcaagaatattagatatagactagggtgcggtttctcaaatcaaatgatcatcaacaacaggggggtaggtgaaacagtcaactctaaacttaaaagttacccccgtaattgtcatatcatatacccagattgcaataaggatctgttttgtatcaaagacagctgcatatctaggaagctctctgagctattcaagaagggtaattccctatactcgaagataagtcaccaggtactggattgtcttacaagagtcaacggtaaattaggcttgggcacagatcttactcatggcctgaaggagggtatcctcgacttggggttacacatgcatagctctcaatggttcgagactttcctgttctggttcactatcaagacagagatgagatcaatgataaaagaacagtcccatatatgccacaagaggaggtataacccggtttttgtgtcgggggatgcattcgaggtgctcgtatcacgagacctcgttgcgataattgacaagaatacccagtatgtctactatttgacatttgagctggtccttatgtattgtgatgtcatagagggcagacttatgacggagacagccatggctatagaccagagatattcagaacttctaagccgggtcagatatttgtgggatcttattgacgggttcttcccaacactaggtaacaccacataccaggttgttgctctgcttgaaccattgtcgttggcttatcttcaacttcaggatgtcaccctggagttaagaggtgcttttttagatcactgcttcaaggaactctatgagatactagagcattgcggcattgacacggaaggcacctacaattccatcactgaaggattggactacgtatttatcacccacgatatccatttaactggggagatattttcgtttttccggagtttcggacacccccgcctcgaagcggtcaccgccgcagagaatgtcagaaaacatatgaaccagccgaaggtaattagttatgagactatgatgaaagggcatgcagtgttttgcgggataatcataaatgggtttagagaccggcatggcggcagctggccccccgttgcattgccagaacatgcttctgctgcgattcggaatgcgcaggcatccggtgaggggctgacccatgacttgtgtatagacaactggaagtcctttgttggattcagatttggctgtttcatgccgctcagcctagatagtgatttgaccatgtaccttaaagacaaagcactggctgcactgaagaatgagtgggattcagtttacccgaaagaatacctccgctataatccacccagagggacagagtcaaggcggctggtggaggtgttcctgaatgactccagctttgatccttataacatgataatgtacgtggtgaatggctcttaccttaaagaccctgagtttaatctctcatatagcttaaaggagaaggagataaaagagacagggcggttgtttgcaaaaatgacttacaagatgcgggcctgtcaggtaattgcggagaatctgatatcaaatggggtcgggaagtatttccgagacaatggaatggcgaaagacgagcatgacctaacaaaagccctccacactctggcagtctcaggtgttcccaagaacaataaagataaccaccgaggtggccctcctagaaggaccacaagccgagagatgagatcaaaccaagacatcaacagacaaactagagacaaggtccagggggagcccatgtacaactatttgcggtgccaaccaatcagccctgaccagagtgagtcatacgagactgttagtgcattcatcaccgctgaccttaagaagtattgcctgaattggagatacgagacaatcagcatatttgcacaaagacttaatgaaatttacggactgccatccttctttcaatggttacacagggtattggaaaaatctgtgctctacgtcagtgacccacattgcccccccgacttagatgatcacgtccccctggacagtgtccctaatgcccaaatattcatcaagtacccaatgggcggaatagaaggttattgtcaaaaactatggacaatcagtactataccatatttgtatctggcagcctatgagagcggagtaagaattgcctcactagtgcaaggtgataatcagacaattgcagtgacaaaaagagtcccgagttcttggccttactcactaaaaaagagggaggcatccaaagcagctcagaattacttcgtggttttaaggcagaggttgcacgatgtaggccatcacttaaaggctaatgaaaccatagtatcttctcacttttttgtatactcgaaagggatttattatgatggcctattagtctcacaatcactaaagagcattgccaggtgtgttttctggtccgagactattgtagacgaaaccagagcggcctgtagcaatattgcaacaactattgccaagagtatagagaggggttatgataggtaccttgcatactctttgaatatcctcaagattttccaacagatccttatatccctcaacttcactattaacacgacaatgactcaagatgttgtggcacctatcatcgagaatggtgatctactaataaggatggcacttttgccagcacccattgggggtctcaactatcttaacatgagcagattatttgtgagaaatatcggtgacccggtcacttcctctatagccgacctgaagaggatgatagacgctgggctaatgccagaggaaacattgcatcaagtgatgacccagacccctggagaatcgtcttaccttgattgggcaagtgacccttattctgctaacctgacctgcgtacagagtataactcgccttctcaagaacatcactgctcgatatattttaatcagcagccctaatccgatgctaaaaggattgttccatgaggggagtagagatgaagacgaagagcttgcgagtttcttgatggataggcatataattgttcctagagctgcacatgaaatcttagaccatagcataaccggagcaagagaagctatagctgggatgttggataccaccaagggtctgattagaacaagtatgaaacgaggtggcctcacccctcgagtcttagctcgcctttccaattatgattatgaacaattcagatccggaataacattattgaccaagaaagggcagtgttatctcattgacaaggactcgtgctcggtgcagctcgctatagctctgaggggccatatgtgggctaggttagctcgcgggagacctatctatggattagaggtgcccgatatactagaatcgatgaacggctaccttatcaaacgccacgagtcttgtgccatttgcgaaacgggctcaaatcactacgggtggttcttcgtccctgctgggtgccagcttgacgatgtctctagagagacttctgcccttcgtgtaccttatgtcggatcgaccactgaggaaaggacagatatgaaacttgctttcgttagatctccaagtcgatccctcaagtcagcagtcagaattgctacagtttactcatgggcctacggggatgatgaaaaatcatggaatgaagcttggatgctagccaggcagagagctgatatcaccttagatgaattgagaatgatcactccaatctctacatccaccaacctagcccatcggctgagggataggagtacccaggtgaaatattcggggacatcccttgtgagggtagcaagatacacaaccatttccaatgataatttgtcatttgtgatatctgagaagaaagtagatactaacttcatttaccagcaagggatgctgctcggtcttgggatccttgagaatcttttcaggttggaggccaccacaggggtatccaacacagtgttacatctgcacgtggaaacagaatgctgtgttgtacctatggttgatcacccaaggataccgagtctccgtaacattaaagttacgagtgagctatgcacaaaccccttgatctacgacaagtctcccatcatagatcacgatgcaacccgattatactcacagagccacaggagacatttggtggagtttgtgacctggtcaacaagccagctttaccatatactggctaagtctacagcaatgtccatgattgagttgatcacaagattcgagaaagatcacatgaatgaaatagccgccctgattggagatgacgacatcaacagtttcatcacagaatttttgctagtagagcccagattgtttatagtctacctcggccagtgtgccgccatcaattgggcttttgacatacattatcatcggccctcgggcaagtaccagatgggggagctcctctactctttactctctaggatgagcaaaggggtatataagatcttcaccaatgctttgagccaccccaaagtttacaagaaattttggcgaagcggtatcattgagccgactcatggcccatccctagatacacagaacttacatgtcactgtctgtgacatgatatatggatcctacgtcacctatctggatcttttactgaatgatgagctagacgattacacgtatttgctctgcgagagtgatgaggacgtggtcacagacaggttcgataacattcaagccaagcatctctgtgtattggccgacttatattgcacctccaaaaggtgcccctcgataatcgggatgtcccctatagaaaaatgtaccgtcctcacacattacatcaagggagaatcaatccaatccccgtctgggacctcatggaacactggtccccttgtagtagatcattactcgtgctctctgacctaccttcgccgaggttccattaaacaaatcaggttgagagtggatcctgggtttgtattcgaggcgttgacagacatcgacatcaaacagcctcgcaaggctaaactggatgtatcggttgtggggttgactgatttctctcccccttgggacaacgtcggtgattttctagggactatcaacacattgagacacaatctgcccgtcaccggaaccggggtcttgaactatgaagtccatgcttatcgtagaattggcctgaattcatcagcatgttataaggctgtagaaatttccacgttaatcaagtcatcattagaagtcggagagcatggattgttcttaggggaaggttccggttccatgctggctgcgtacaaggaagttctcaaattagcaaactgttattacaacagcggggtgacagcggaggacagagccggacagagggaaatatccccctatccttcagagatgagcctagtagagaaccagatggggatagagaggagtgttaaggtgctgttcaacggcaaacccgaagtaacttgggtagggaccaccgattgctataagtatataatcagtaacattcaaacctctagtttaggtctcatacactcagatattgagacactcccaaacaaagatgctgttgaaaaattagaagaatttgcctctatcctatccttatccctaattttaggaaaaatcggctctattacggttatcaaaattatgcccattagcggagactttactcaaggcttcataggctatgccattcaatattttagggagagcctgcttgcctatccgagatatagcaacttcatctccactgagtgttaccttattatgataggattgaaagccaatcggttgataaacccggaggccattaagcaaagcatagtcagagtggggaccaggactgcaccaggactagtgagccacatattatcagagaaacagaaaggctgtattcaatcttttctgggtgatccttatatccaaggagacttcaacaaacaccttaaagctctaacccctattgagagaatcctagtaaattgtggtctctcgatcaatggtacaaaaatctgtagggatctaatccaccatgatatcgcctccggtccagacggtctgatgagctccacaatcattttatatagggagttggctcatttcaaagacaacataagaagtcagcacgggatgttccacccctaccccgtactggccagtagcaggcaacgtgaattaatcctccgaatagccaagaaattctgggggtatgtcttgctatattctgatgacccgagactgatcggacaaacaatcaagaacttaaagaggaaccacctaacctttgacttacacagtaatccgtttattaagggcctatccaaagctgagaaactactggtgcggacaagctcactcaggagagaatggttgttcactctcgagacgaaagaagtgaaagagtggttcaaattggtgggttacagtgcactcgtcagaggttgattagcgatacatctgcccccttctcctccaccaggtgactctactggcaatctaaaagattaaagaaaactacatattggataagtatctattcccagctttgtctggt

>KP868655/China/Guangdong/2014

accaaacaaagttgggtaaggatagatcttataataactatggactggcaaacttaggagtaaagatcctactgtcggggggaggaggaggagcaagatctttgattatggcgactctccttaaaagcttagcattgttcaaaaggaacaaagacaaagcgccgacagcatcaggttcaggaggggccatccgggggattaagaatgttatcatagtcccgattcccggagactcgtccatcactacccgttcaaggctgctcgacaggcttgtcagattggccggagatcctgacatcaacgggtcaaagctgaccggggtgatgatcagcatgttatccttgttcgtagagtcacccgggcaattgatacagcgaatcacagatgatccggatgttagtatccgccttgttgaggtagttcaaagtactaggtcccagtccgggttgacctttgcatcacgtggtgctgatttagacaacgaggcagacatgtatttttcaactgaggggccctcgagtggaggtaagaaaaggatcaactggtttgagaacagagaaataatagacatagaggtgcaggatccagaagagttcaatatgttgttagcctccatactagcacaagtctggatccttctggccaaggctgttacggcaccagatacggcagctgactcagaactgagaaggtgggttaaatacacacaacaaaggagagtgattggggaatttcgccttgacaaggggtggctggatgcagtccgcaacagaattgcagaagatctatcactccggcggttcatggtatctctcatacttgacatcaagaggaccccaggcaacaagccaaggattgcagaaatgatctgcgacattgacaactatattgtcgaagcaggactcgccagtttcatccttgctatcaaatttggtatcgaaaccatgtatcctgcattagggctccacgagtttgccggagaattgtccactattgagtccttgatgaacttgtatcaacagctaggagaggttgcaccctacatggtaattctagagaattcaattcagaacaagtttagtgcaggagcttatcccctcctctggagctatgcgatgggtgtcggagtcgagctggagaactcaatggggggcttgaactttggcaggtcatattttgaccctgcctattttcgtctcggacaggagatggtcagaagatctgcaggaaaggtcagctctgtgattgcggctgagctcggcatcacagcagaggaagctaaactagtctcggaaatcgcctcgcaggctggggacgaaagaaccgccagagggactgggcctcgacaggcgcaggtttccttccttcagcaccaaacaggagggggagagtcgtccgcaccagcgaccagagaaggggtcaaagctgcgatcccaaacggagctgaagaaagggacagaaagcaaacacgcccaggaaggcccagaggagagacctccggccaactgctcctggacatcatgccagaggatgagatcttgcgagagtctggtcaaaaccctcgtgaggctcaaagatcggccgaggcactcttcaggctgcaggccatggccaagattctggaggaccaggaggagggagaagacaacagtcaggtctacaacgacaaggatctcctcggctaagcagacgcaccctctgtcgaaatcagtgacaagacatctcttaccagtattataaaaaacttaggacccaggtccaagcaagcacacatcgacactccaaccagtcgagcggagaccaccgatggcagaagaacaagcataccatgtcaacaaggggctggaatgtatcaagtctctcaaagcctcacccccggatctatccaccatcagagataccatcgagagctggagagaggggcttagcccctcgggccgtgcaacaccgaaccctgatacgtccgagggagatcatcagaatatcaaccaatcatgctcaccggcaatcggaccaaacaaagtctacttgtctcctgaagataatctcggatttagagagatcactggcaacgactgtgaggctgggctcggaggggtccagagagaaggatccaactctcaagtacagcgttaccatgtttatagtcacgggggtgaagagattgaaggactcgaggatgctgactctctcgtggttcaagcagatcctccagttgctaacgtcttcaatggaggagaggatggatctgacgacagcgatgtggactctggcccagatgatcccggcagagatactctatatgaccggggatctgttgccggcaatggtatcgctaggtccacagatgtcgaaaaactagaaggtgctgatattcaagaagttcttaactcccagaaaggcaaaggaggaagattccagggc-ggaaaaccttgcgagtcccggaaatacccgatgtcaagcactccagaccatcagcccaatcaattaaaaagggcacagacgggaactcagtctcatctggaacggtgatagagtgtttatcgataagtggtgcaacccaaactgtgccagagtcaagatgggagtcatcagagcaaaatgcgtctgtggggagtgtcctcaagtctgcgaggagtgcaaagacgatccaggggtcgacacaagaatctggtaccatagcatcaccgactcagcctaaagagaatgactccgagtatgagtatgaggatgacctatttacagagattcaggacatccgtgcaagcattgccaagatccatgatgacaataaaactatcctctcaaagcttgattctatactgttattgaaaggggaagtcgacactatcaagaaacaaatcagcaagcagaatataagtatatccaccattgagggccatctctccagtataatgatagccatccctggctttgggaaggacatcaaggacccaacatccgaagtcgagttgaacccagatctaagacctataataagccgtgactctggcagagctctcgcggaggtcctcaagaaacccgctgtggataggtctcagaaaattggaaccaaagccaactccagctcaaagggtcagcttcttaaggatctccagctaaaacctgtcgacaagcaggcaagctctgcaatcgggtttgtcccgtccgaccatgaatcatccagaaatgtcatccgctccataatcaagtcgagcaagctaaatattgatcacaaggactatcttctagatttactaaatgatgtgaaaggctccaaggatcttaaggaatttcacaagatgctaacagcaattctcgccaagcacccgtaacacatcctccagtcatcatctcatactcgactaaaaacatcctttcaatcaggctattacaaaaaacttaggagcaagggcaactgagcttcgcagacaggatgaccgagatctacgacttcgataaatcagcatgggatgtcaaagggtcaattgcccgcatagaacccaccacctaccacgacggccgactgataccccaggtgagggtcatcgatcctggtctgggagacagaaaagatgagtgctttatgtacctgtttctcctaggagtgattgaggataacgaccccctgtctccccccgtcggaagaacctttggctctctacctctaggggttggtaggtcaactgccaagccagaagaactactaagggaggccacagaattagatatagtggtgaggcgcactgcaggagtaaatgagaaactggtattttacaacaacactccgctgtccttgttaacgccctggaagaaagttctgacaaccggaagtgtgtttagcgctaaccaggtttgcaatgcagtcaacctagtcccgcttgatactccccagagattcagggttgtgtacatgagcataactagattatcagacaatggttactatagtgtgcccagaagaatgctggagttccgctcggccaatgcagtagccttcaacatcttggttacactaagaattgaaaatggcacaaaccctagaagatacatagtcggctcatgggagaattctgaggtcacatttatggtacacgtgggcaactttaggagaaagaagaacgaagtatactctgctgattactgcaaaatgaagattgaaaagatgggtttagtttttgccctgggtggaataggcggaacaagtctccatattagaagcaccgggaaaatgagcaaaaccctccatgcacagctggggttcaagaaaatcttatgttaccccctaatggatgtcaatgaggatcttaaccgatatctctggcgggcagagtgccgaatagtcaaaatccaagctgtcttacagccatcagtaccccaagagttccgtgtctacgatgatgtcatcatcaacgatgatcaaggcttgttcaagatcttgtagttcatttgcaacatcatgacgtgggtcgcaattttgacatttctgtttcttttcccaaatgttgttgcgtgtcagattcactggggcaatctatccaagatcgggattgtaggaacagggagtgccagctacaaggtgatgactaggccaagccaccagactctggttataaagttaatgccaaatataacggccatcgacaattgtacaaagtcagagattgcagagtacaagagattgctgatcacagtgttaaagcctgtagaggatgctctgtcggtgataaccaagaatgtaagaccaattcaaactctaacacctgggcgtagaacccgccgttttgctggagctgttctggccggggtagcacttggagttgcgacagccgctcagataactgcaggagtcgcccttcatcaatcattgatgaactcccaagcaattgagagtttaaaaaccagtcttgagatgtcgaatcaggcaatagaagaaatcagacttgcaaataaggagaccatactggcagtacagggcgtccaggattatatcaacaatgagctcgtcccttctgttcatagaatgtcatgcgagctggtaggtcacaagctcggcctcaagctccttaggtactacaccgagatcctgtccatattcgggcccagtcttcgagacccgatatctgccgaaatatcaatccaggcacttagttatgcattaggcggagacattaataaaatcctggacaagcttgggtatagcggtggggatttccttgccatcctagaaagcaagggaataaaggcccgggttacatatgtggacacaagagattactttataatccttagcatcgcctacccaaccttatctgagatcaagggagtgatagttcacaagatagaagctataacatacaacattggggcacaggagtggtatactactatccctaaatatgtagccactcaggggtatctgatatcgaactttgatgagacgtcatgcgtattcactccagaggggacagtttgcagccagaatgcgttgtacccaatgagcccattgcttcaggaatgtttcagggggtcaacaaaatcgtgcgccagaaccttagtttcagggaccataagtaatagatttatcctatcaaaagggaacctgattgcaaattgtgcgtcagttttgtgcaaatgttacacaacagagacagttatcagccaggatcctgacaaactactaactgttgtagcatccgacaagtgtcctgtagttgaggtggatggagtgacaatacaggtcggcagtcgagagtatccggattctgtatacttacacaaaatagacttaggtccagccatctccctagaaaaactggatgtaggcaccaatttaggcaatgcagtcacaagactggagaatgcaaaggagctcctagatgcatcagaccaaatactgaagactgttaaaggggtacctttcagtgggaatatgtacatagcactggcagcttgcataggagtatccctgggccttgtcacattaatatgctgctgtaaggggaggtgtaagaacaaggaaatccctatctccaaaatcaacccagggctcaaacccgacctgaccgggacctcaaagtcgtacgtaagatcactgtagtcagaatcacctggatcatctggcatcgcacacatacatgcacgacacaggcagtccgaggacgcaagaaacccagcctccggtcacccacccgaccccactccacgctccaccacacattagtcatcaaacaaaacttaggacgaaaggtcaatcaccatgtccgcacaaagggagaggatcaatgccttctacaaagacaatcctcacaataagaaccatagggtgatcctggatagagaacgcttggtcattgaaagaccctacatcttgcttggagtcctgctggtaatgttcctgagtctaatcggactgctggccattgcagggatcaggcttcaccgggccaccgttggaacttcagagatccagagtcggctgaataccaatattaagttgaccgaatctattgatcaccagactaaggatgtcttaactcccctttttaaaatcattggcgatgaagtcggcatcagaattccacagaaattcagtgatcttgtcaagttcatctccgataagattaaattcctcaaccctgatagagagtatgatttcagagatctccggtggtgtatgaacccccccgagagagtcaaaattaattttgatcagttttgtgagtacaaggctgcggttaagtcaattgaacatatatttgagtcaccactcaacaagtcaaaaaagctgcaatctttgactctcgggcccggaacaggctgtctaggcaggacagtaacaagagcccatttctcagaacttacaatgaccttaatggacctggatctagagacgaagcacaacgtgtcctcagtgtttaccgtagttgaagagggattattcggaagaacatataccgtctggagatccgatgccagggatccgagcaccgatctaggtatcggccattttttaagagtcttcgagattggactggtaagagatcttgggctgggtccccctgtttttcatatgaccaactatctcacggtgaacatgagtgatgactatcggagatgtcttttagcggtaggggagttgaagttgacagccctatgcacctcatctgagactgtgacactgagtgagagaggagttccaaggagggaacctcttgtggttgtgatacttaatctagctggacccactctagggggagagctatacagtgtcttgcctacctctgatctcatggtggagaaactctatttgtcttcacatagagggatcatcaaagatgatgaggccaattgggtagtgccgtctaccgatgttcgtgatcttcagaacaaaggtgaatgtctggtggaagcatgcaagactcgacctccttcattttgcaatggcacaggatcaggcccgtggtcagaagggagaatccctgcctacggggtgatcagggtcagtcttgacttagcgagtgacccggatgtagttatcacttcagtgtttggcccactgatacctcacctatccggcatggatctttacaacaacccgttttcaaaagctgtatggttggctgtaccaccttatgagcagtcatttctaggaatgataaatacaattggattccctaacagagcagaggttatgccgcacattttgaccacagagatcagaggccctcggggtcgttgccatgttcccatagaattgtcccgccgggttgatgacgatatcaagatcgggtccaacatggtcatattgccgacgatggacctgaggtatattacagccacttatgatgtttccaggagcgagcatgcaatcgtgtactatatctatgacacgagtcgctcatcatcttacttctacccagttcgactgaatttcaaaggcaatcctctctctttgaggatagagtgtttcccctggcgtcataaggtgtggtgctaccatgattgtcttatatacaacaccataacaggtgaagaggtccatacgagagggctgaccggcatagaggtaacatgcaatccagtctgagcagagctacgaccgtcactcaggcagtcccttgagtcgccaccgagtccaagcagcacagcctgggacaatcaacggcacagcccagccaacaatgttataaaaaacttaggagccagggttgtaggggccatggactctctatcagtcaatcaggtcttgtaccctgaggttcatctagacagccctattgtcacaaacaaactagttgccatccttgagtactcgggtatcgaccataactatgttcttgaagaccagactcttgtcaagaatattaggtataggctggggtgcggtttttcaaatcaaatgatcatcaataacagggggttgggtgaaacagtcaattctaaacttaaaagttacccccataatcgtcatatcatatacccggattgcaataaggagttgtcttgtatcaaggatagctgcatatctaagaagctctcggagctattcaagaagggtaattccttgtactctaagataagtcaccaggtactggattgtcttaagagagtcaatgggaaattaggtctgggcacagatcttacccatggtctgaaggagggtatccttgacctagggttgcacatgcatagctctcaatggttcgagactttcctgttctggttcactatcaagacagagatgagatcaatgatcaaagaacagtcccatatatgccacaagaggaggtataacccgacttttgtgtcgggggatgcattcgaggtgctcgtatcgcgagacctcgttgtgataattgataagaatacccagtatgtcttctacttaacatttgagctggtccttatgtattgtgatgttatagagggtagacttatgacggagacagccatggccatagaccagagatattcagaacttctaagccgggtcagatacttgtgggatcttattgatgggttcttcccaacactaggcaacaccacataccaaattgttgctctgcttgaaccattatcgttggcttatcttcaacttcaggatgtcactctggagttaagaggtgcttttttagaccattgcttcaaagaactctatgagatactggaacattgcggcattgacacagcaggcacttacaattccatcactgaaggattggattacgtattcatcacccacgatatacatctaactggggagattttttcatttttccggagtttcggacacccccgtctagaagcagtcaccgctgcggaaaatgtcagaaaacatatgaaccaaccgaaggtaatcagttatgagactatgatgaaaggccatgcggtattttgcgggataatcataaatggttttcgggaccgacatggcggcagctggccccctgttgcactgccagaacatgcctctgctgcgatccggaatgcgcaggcatcaggtgagggactgacccatgacctgtgtatagacaactggaaatcctttgttggattcaaatttggctgctttatgccactcagcctagatagtgatttgaccatgtatctcaaagacaaggcattggctgcactgaagaatgagtgggattcagtttacccgaaagaatacctccgttataacccacctagagggacagagtcaaggcgattggtagaggtattcctgaatgactccagctttgatccttataacatgataatgtacgtggtgaatggctcctaccttaaagaccctgagtttaacctctcatacagcctaaaagagaaggagataaaggagacagggcggttgtttgccaaaatgacctataagatgcgggcctgtcaggtaatcgctgaaaatctgatatcgaatggtgttgggaagtatttccgagacaatgggatggcaaaggacgagcatgacctaacaaaagcccttcacaccctggcagtctcaggtgttcccaaaaacaacaaagataaccaccgaggtgggcctcccagaaggacaacaaaccgagggttgagatcaagccaaggcaccaaaacacaagatagagacaaggttcaagggggacctatgtacaactatttgcgatgccaaccgatcagccctgatcagggtgagtcatacgagactgttagtgcattcatcaccgctgaccttaagaagtattgcctgaattggagatacgagacaatcagcatatttgcacagaggctgaatgaaatatatggactaccatccttctttcaatggttacacaggatattggaaaaatccgtactctacgtcagtgacccacattgtccccccgatctagataatcatatccctctggacagtgtccccaatgcccaaatattcatcaagtacccaatgggcggaatagaaggttattgccaaaaactatggacaatcagcactataccatacttgtatctggcagcctatgagagcggagtaagaatcgcctcactggtgcagggtgacaatcagacaatcgcagtgacaaaaagagttccaagttcttggccttattcactaaaaaagagggaggcctccaaggccgctcaaaattacttcgtagtcttaaggcaaagattgcacgatgtaggtcatcacttaaaggctaatgagaccatagtatcttctcacttttttgtatattccaaagggatttattatgacggcctgctagtctcacaatcactaaagagcatcgccagatgtgtcttctggtccgagactatagtggatgaaaccagagcggcgtgcagtaatattgcaacaactgtcgctaagagtatagagaggggttatgataggtaccttgcatattctttgaatatcctcaagattttccaacagatccttatatcccttaacttcactattaacacaacaatgactcaggatgtcgtggcaccgatcatcgagaacggtgatttgctgataaggatggcactcttgccagcacccatcgggggtctcaattatcttaacatgagcaggttatttgtgagaaatatcggtgacccggtcacctcctctatagccgacctgaagaggatgatagatgccgggctaatgccagaagaaacattgcatcaagtgatgacccagaccccgggagaatcatcctacctagattgggcaagtgacccttattctgccaacctaccctgcgtacagagtatcactcgccttctcaaaaacatcactgcacgatatattttaatcagcagcccaaacccgatgctgaaagggttatttcatgaggggagtagagatgaagacgaggagcttgcaagtttcctaatggatcggcatataattgttccaagagctgcacatgaaatcttagaccatagtataaccggagcaagagaagctatagccgggatgttggacaccaccaagggtctgattagaacaagtatgaaacggggcggcctcacccctcgagtcttagcccgcctttccaattatgattatgaacaattccgatccgggataacactattaacaaagaaagggcagtgttatctcattgacaaggactcgtgctcggtgcagctcgctatagctctgaggggccacatgtgggctaagttggctcgcgggagaccaatctatgggttggaggtgcctgatgtattagaatcgatgaacggctaccttatcaaacgccacgagtcgtgtgctatctgtgaaacgggctcaagtcactacggatggtttttcgtccctgcagggtgccagcttgacgatgtatctagagagacttcggctcttcgtgtaccttatgtcggatcaacaactgaggaaaggacagacatgaaacttgcatttgttaggtctccaagtagatccctcaaatcagcagttagaattgcaacagtttactcatgggcttacggggatgatgagaaatcatggagtgaagcctggatgctagctaggcagagagccaatatcaccttagatgaattgagaatgatcactccagtctccacatccaccaacttagcccaccggttgagggatcggagtacccaggtgaaatactcggggacatcccttgtgagggttgcaagatacacaaccatctccaatgataacttgtcgtttgtgatatctgagaaaaaagtggataccaacttcatttatcagcaagggatgttgcttggtcttgggatccttgagaacctctttaggctagaggccaccaccggagtatccaacacagtgctacacctacacgtggaaacagaatgttgtgttgtacctatggtggatcacccaaggataccgagtctccgtaatataaaagttacggatgagctatgcacaaaccctttgatctacgacaggtcccccatcatagagcacgatgcaacccggctatattcacagagccacaggagacatttggtggagtttgttacctggtcaacaagccagctttaccatatactggctaagtctacagcaatgtccatgattgaattgatcacgagattcgagaaagatcacatgaatgaaatagccgccctgattggcgatgacgacatcaacagcttcatcacagaatttttgctagtggagcctagattatttatagtttaccttggtcagtgtgctgccatcaattgggcctttgatatacattatcatcggccctcgggcaagtaccagatgggggaacttctctactctctgctctctcggatgagcaaaggagtatataagatctttactaatgctttgagccaccccaaagtttacaagaaattttggcgaagtggcatcattgagccgattcatggcccatccttggatacacagaatttacatgtcactgtctgtgacatgatatatgggtcctatgtcacctatttggatcttttgctgaatgatgagctagatgattacccgtatttgctctgcgagagtgatgaggacgtggtcacagacaggttcgacaacattcaagccaaacatctctgtgtattggccgatgtatattgcagctccaagaggtgtccttcaataatcgggatgtctcctatagaaaaatgtaccattctcacacattacatcaagggagaatcagtacaatccccgtctgggacctcatggaacactgatccccttgtagtagatcattactcatgctctctgacctaccttcgccgcggttccatcaaacaaatcaggttgagggtggatcctgggtttgtattcgaggcgttgacagacgtcgactttaaacaacctcgcaaagctaagttagatatatcggtcgttgggctgactgatttctctcccccttacgacaacgtcggtgattttttagggactatcaacacattgaggcacgatctgcctgtcaccggaaccggggtctcgaactatgaagtccacgcttatcgtagaattggcctgaattcatcagcatgttataaagccgtagagatctccacgttaatcaagccatccttagaagtcggagagcatggattgttcttaggagaaggttccggttcaatgctggctgcgtacaaagaagttcttaaattagcaaattgttactacaacagcggagtaacagcggagggcagagccggacagagggaaatatctccctatccttcagagatgagcctagtagagaatcaaatggggatagagcggagtgttaaagtgctgttcaacgggaaacctgaagtaacttgggtggggaccacagattgctacaagtacataatcagtaacatccaaacctctagtctgggtttcatacattcagatattgagacgctcccaaccaaagatgctgttgagaaattggaggaatttgcctctatcctatccttatccttgattttggggaaaatcggctctattacagttgtcaaaattatgcccattagcggggattttacccaaggcttcatagcctatgccatccaatattttagggagagcctgcttgcctacccgagatatagtaacttcatctcgactgagtgttaccttattatgataggattaaaggccaatcgattgataaacccagaagccattaagcaaagcataatcagagcgggggtcaggactgcaccaggacttgtgagccatgtattatcagagaaacaaaaaggttgtattcaatctttcttgggtgatccttatacccaaggagacttcaataagcaccttaaatctctaacctctattgagaaaatcctggtaaattgtggtctctcgatcaatggcataaaaatctgtagagatctaatccaccatgatatcgcctccggtccagacggccttatgagctctacaattattttatatagggaattggctcatttcaaagataatataaggagtcagcacggaatgttccacccctacccggtactggccaacagcaggcaacgtgaattaatccttcgaatagccaagaaattctgggggtatgtcttgctatattctgatgacccggcactaatcagacaaacaatcaagaacttgaaacggaatcatctaacctttgacttacatagtaacccatttattaaagggctatccaaggctgagaaactgctggtgcggacgagttcactaagaagagaatggttgttcactctcgaaacgaaagaagtaaaagagtggttcaaattggtaggttacagtgcactcatcagaggctagtcagctatgcatctgcccccttctcctccgccatgagaccccactggcgatccagaagattaaagaaaactacatattggataagtatctattcccagctttgtctggt

>KR140086/India/Izatnagar/1994

accaaacaaagttgggtaaggatagatcttataataactatggactggcaaacttaggagtaaagatcctactgtcggggggaggaggaggagcaagatctttgattatggcgactctccttaaaagcttagcattgttcaaaaggaacaaagacaaagcgccgacggcatcaggttcaggaggggccatccgggggattaagaatgttatcatagtcccgattcccggagactcgtccatcattacccgttcaagactgctcgacaggcttgtcagattggccggagatcctgacatcaacgggtcaaagctgaccggcgtgatgatcagcatgttatctttgttcgtagagtcacccgggcaattgatacagcgaatcacagatgatccagatgttagtatccgccttgttgaggtagttcaaagtactaggtcccagtccgggttgacctttgcatcacgtggtgctgatttggacaacgaggcagacatgtatttttcaactgaggggccctcgagtggaggtaagaaaaggatcaactggtttgagaacagagaaataatagacatagaggtgcaggatccagaagagttcaatatgttgttagcctccatactagcacaagtctggatccttctggccaaggctgttacggcaccggatacggcagctgactcagaactgagaaggtgggttaaatacacacaacaaaggagagtgattggggaatttcgccttgacaaagggtggctggatgcagtccgcaacagaattgcagaagatctatcactccggcggttcatggtatctctcatacttgacatcaagaggacccccggcaacaagccaaggattgcagaaatgatctgcgacattgacaattatattgtcgaagccggactcgccagtttcatccttactatcaagtttggtattgaaaccatgtatcctgcattagggctccacgagtttgccggggaattgtccactattgagtccttgatgaacttgtatcaacagctaggagaggttgcaccctacatggtaattctagagaactcaattcagaacaagtttagtgcaggagcttatcccctcctctggagctatgcgatgggtgtcggagttgagctggagaactcaatggggggcttgaactttggcaggtcatattttgaccctgcctattttcgtctcggacaggagatggtcagaagatctgcaggaaaggtcagctctgtgattgcggctgagctcggcatcacagcagaggaagctaaactagtctcggaaatcgcctcgcaggctggggacgaaagaaccgctagagggactgggcctcgacaggcgcaggtctccttcctccagcacaaaacaggagagggagagtcgtccgcaccagcgaccagagaaggggtcaaggctgcgatcccaaacggatccgaagaaagggacaggaagcaaacacgctcaggaaggcccagaggagagacccccagccaactgctcctggaaatcatgccagaggatgaggtcycgcgagagtctggccaaaaccctcgtgaggcccagagatcggccgaggcactcttcaggctgcaggccatggccaagattctggaggaccaggaggagggagaagacaacagtcaggtctacaacgacaaggatctcctcggctaagcagacgcaccctctgtcgaaatcagtgacaagacatctcctaccagtattataaaaaacttaggacccaggtccaagcaagcacacatcgacactccaaccagtcgagcggagaccaccgatggcagaagaacaagcataccatgtcaacaaggggctggaatgtatcaagtctctcaaagcctctcccccggatctatccaccatcagagataccatcgagagctggagagaggggcttagcccctcgggccgtgcaacaccgaaccctgatacgtccgagggagatcatcagaatatcaaccaatcatgctcaccagcaatcggaccaaacaaagtctacttgtctcctgaagataatctcggatttagagagatcactggcaacgactgtgaggctgggctcggaggagtccagggaaaaggatccaactctcaagtacagcgttactatgtttatagccacgggggtgaagagattgaaggactcgaggatgctgactctctcgtggttcaagcagattctccagttgctaacatcttcaatggaggagaggatggatctgacgacagcgatgtggactctggcccagatgatcccggcagagatactctatatgaccggggatctgttgccggcaatgatgtcgctaggtccacagatgtcgagaaactagaaggtgctgatattcaagaagttcttaactcccagaaaggcaaaggaggaagattccaaggcgggaaaaccttacgagtcccggaaatacccgatgtcaagcactccagaccatcagcccaatcaattaaaaagggcacagacgggaactcagtctcatctggaacggtgacagagtgtttatcgataagtggtgcaacccaagctgtgccagagtcaagatgggagtcatcagagcaaaatgcgtctgtggggagtgtcctcaagtctgcgaggagtgcaaagacgatccaggagtcgacacaagaatctggtaccatagcatcactgactcagcctaaagagaatgactccgagtatgagtatgaggatgacctatttacagagattcaggacatccgtgcaagcattgccaagatccatgatgacaacaaaactatcctctcaaagcttgattctatactgttattgaaaggagaagtcgacactatcaagaaacaaatcagcaagcagaatataagtatatccaccattgagggccatctctccagtataatgatagccatcccgggctttgggaaggatatcaaggacccaacatccgaggttgagttgaacccagatttaagacctataataagccgtgactctggcagagctctcgcggaggtcctcaagaaacccgctgtggataggtctcagaaaattggaaccaaagccaactccagctcaaagggtcagcttcttaaggatctccagctaaaacctgtcgacaagcaggcaagctctgcaatcgggtttgtcccatccgaccatgaatcatccagaaatgtcatccgctccataatcaagtcgagcaagctaaacattgatcacaaggactatcttctagatttactaaatgatgtgaaaggctccaaggatcttaaggaattccacaagatgctaacagcaattctcgccaaacacccgtaacacaccctccagtcaccatctcagactcggctgaaaacatcctctcaatcaggctattacaaaaaacttaggagcaagggtaactgagcttcgcagacaagatgaccgagatctacgacttcgataaatcagcatgggatgtcaaagggtcaattgcccgcatagaacccaccacctaccacgacggccgactgataccccaggtaagggtcatcgatcctggtctgggagacagaaaagatgagtgcttcatgtacctgtttctcctaggagtgattgaggataacgaccccctgtctccccccgtcggaagaacctttggctctttacctctaggggtcggtaggtcaactgctaagccagaagaactactaagggaggccacagaactagatatagtggtgaggcgcactgcaggagtaaatgagaaactggtattttacaacaacactccgctgtccttgttaacgccctggaagaaagttctgacaaccggaagtgtgtttagcgctaaccaggtttgcaatgcagtcaacctagtcccgcttgatactccccagagattcagggttgtgtacatgagcataactagattatcagacaatggttactatagtgtgcccagaagaatgctggagttccgctcagccaatgcagtagccttcaacatcttggttacactaagaattgaaaatggcacaaaccctagaagatacatagtcggctcatgggagaatccagaggtcacatttatggtacacgtgggcaactttagaagaaagaagaacgaagtatactctgctgattactgcaaaatgaagattgaaaagatgggtttagtttttgccctgggaggaataggtggaacaagtctccatattagaagcaccgggaaaatgagcaaaaccctccatgcacagctggggttcaagaaaatcttatgttaccccctaatggatgttaatgaggatcttaaccgatatctctggcgggcagagtgccgaatagtcaaaatccaagccgtcttacagccatcagtaccccaagaattccgtgtctacgatgatgtcatcatcaacgatgatcaaggcttgttcaagatcccgtagttcaccctcaacatcatgacacgggtcgcaatcttgacatttctgtttcttttcccaaatgctgttgcgtgccagattcactggggcaatctatccaagatcgggattgtaggaacagggagtgccagctacaaggtgatgactaggccgagccaccaaactctggttataaagttaatgccaaatataacagctatcgacaattgtacaaagtcagagattgcagagtacaagagattgctgatcacagtgttaaagcctgtagaggatgctctgtcagtgataaccaagaatgtaagaccaattcaaactctgacacctgggcgtagaactcgccgttttgctggagctgttctggccggagtagcacttggagttgcgacagccgctcagataactgcaggagtcgcccttcatcaatcattgatgaactcccaagcaattgagagtttaaaaaccagtcttgagaagtcgaatcaggcaatagaagaaatcagacttgcaaataaggagaccatactggcagtacagggcgtccaggattatatcaacaatgagcttgtcccttctgttcatagaatgtcatgcgagctggtaggtcacaagctcggcctcaagctccttaggtactacaccgagatcctgtccatattcgggcccagccttcgagacccgatagctgccgaaatatcaatccaggcactcagttatgcattaggcggagacattaataaaatcctggacaagcttgggtatagcggtggggatttccttgccatcctagaaagcaagggaataaaggcccgggtcacatatgtggacacaagagattactttataattcttagcatcgcctacccaaccttatctgagatcaagggagtgatagttcacaagatagaagctataacatacaacattggggcacaggagtggtatactactatccctaaatatgtagccactcaggggtatctgatatcgaactttgatgagacgtcatgcgtattcactccagaggggacagtttgcagccagaatgcgttgtacccaatgagcccattgcttcaggaatgtttcagggggtcaacaaaatcgtgcgccagaaccctagtttcagggaccataagtaatagatttatcctatcaaaagggaacctgattgcaaattgtgcgtcagttttgtgcaaatgttacacaacggagacagttatcagccaagatcctgacaaactactaactgttgtagcatccgacaagtgtcctgtagttgaggtggatggagtgacaatacaggtcggcagtcgagagtatccggattctgtatacttacacaaaatagacttaggtccagccatctccctagaaaaactggatgtaggcaccaatttaggcaatgcagtcacaagactggagaatgcaaaggagctcctagatgcatcagaccaaatactgaagactgttaaaggggtacctttcggtgggaatatgtacatagcactggcagcttgcattggggtatccctagggcttgtcacattaatatgctgctgtaaggggaggtgtaagaacaaggaaatccctatctccaaaatcaacccagggctcaaacccgacctgaccgggacctcaaagtcgtacgtgagatcactgtagtcagaatcacctgaatcatccggcatcacacacatacatgtacgacacaagcagtctgaggacgcaggaaacccagcctccgatcacccacctgaccctactccacactccaccacacattagtcatcaaacaaaacttaggacgaaaggtcaatcaccatgtccgcacaaagagaaaggatcaatgccttctacaaagacaatcctcacaataagaaccatagggtgatcctggatagagaacgcttggtcattgaaagaccctacatcttgcttggagtcctgctggtaatgttcctgagtctaatcggactgctggccattgcagggatcaggcttcaccgggccaccgttggaactttagagatccagagtcggctgaataccaatattgagttgaccgaatctattgatcaccaaactaaggatgtcttaactcccctttttaaaatcattggcgatgaagtcggcatcagaattccacagaaattcagtgatcttgtcaagttcatctccgataagattaaattcctcaaccctgatagagagtatgatttcagggatctccggtggtgtatgaatccccccgagagagtcaaaattaattttgatcagttttgtgagtacaaagctgcggttaagtcaattgaacatatatttgagtcaccactcaacaagtcaaaaaagctgcaatcattgactcttgggcccggaacaggctgtcaaggcaggacagtaacaagagctcatttctcagaacttacactgaccttaatggacctggatctagagatgaagcacaacgtgtcctcagtgtttaccgtagttgaagagggattattcggaagaacatataccgtctggagatccgatgccagggatccgagcaccgatccaggtatcggccattttttaagagtcttcgagattggactggtaagagatctcgggctgggtccccctgttttccatatgaccaactatctcacagtgaacatgagtgatgactatcggagatgtcttttagcggtaggggagttgaagttgacagccctatgcacctcatctgagactgtgacactgggcgagagaggagttccaaagaggaagcctcttgtggttgtgatacttaacctggctgggcccactctagggggcgaactatacagtgtcttgcctacctctgatctcatggtggagaaactctatttatcttcacatagagggattatcaaagatgacgaggccaattgggtagtgccgtctaccgatgttcgtgatcttcaaaacaaaggtgaatgtctggtggaagcatgcaagactcgacctccttcattttgcaatggcacaggatcaggcccgtggtcagaggggagaatccctgcttacggggtgatcagggtcagtcttgacttagctagtgacccgggtgtagttatcacttcagtgtttggcccactgatacctcacctatccggcatggatctttacaacaacccgttttcaagagctgtatggttggctgtgccaccttatgagcagtcatttctaggaatgataaatacaattggattccctaacagagcagaggttatgccgcacattttgaccacagagatcagaggccctcggggtcgttgccatgttcccatagaattgtcccgcagggttgatgacgatatcaagatcgggtccaacatggtcatattgccgacgatggacctgaggtatattacagccacttatgatgtttccaggagcgagcatgcaatcgtgtactatatctatgacacaggtcgctcatcatcttacttctacccagttcgactgaatttcaaaggcaatcctctctctctgaggatagagtgtttcccttggcgtcataaggtgtggtgctaccatgattgtcttatatacaacaccataacagatgaagaagtccatacgagagggctgaccggtatagaggtaacatgtaatccagtctgagcagagctacgaccgtcactcaggcagtcccttgagtcgccaccgagtccaagcagcacagcctgggacactcaacagcacagcccggccaacaatgttataaaaaacttaggagccagggytgtaggggccatggactccctatcagtcaatcaggtcttgtaccctgaggttcatctagatagccctattgtcacaaacaaactagttgccatccttgagtactcgggtatcgaccataactatgttcttgaagaccagacccttgtcaagaatattaggtatagactggggtgcggtttctcaaatcaaatgatcatcaataacaggggggtgggtgaaacagtcaattctaaacttaaaagttacccccataatcgtcatatcatatacccggattgcaataaggagttgttttgtatcaaagatagctgcatatctaagaagctctcggagctattcaagaagggtaattccttgtactctaagataagtcaccaggtactggattgtcttaagagagtcaatgggaaattaggcctgggcacagatcttacccatggtctgaaggagggtattcttgacctagggttgcacatgcatagctctcaatggttcgagactttcctgttctggttcactatcaagacagagatgagatcaatgatcaaagaacagtcccatatatgccacaagaggaggtataacccgatttttgtgtcgggggatgcattcgaggtgctcgtatcacgagacctcgttgtgataattgataagaatacccagtatgtcttctacttaacatttgagctggtccttatgtattgtgatgtcatagagggcagacttatgacggagacagccatggccatagaccagagatattcagaacttctaagccgggtcagatacttgtgggatcttattgatgggttcttcccaacactaggcaacaccacataccaaattgttgctctgcttgaaccattatcgttggcttatcttcaacttcaggatgtcactctggagttaagaggtgcttttttagaccattgcttcaaagaactctatgagatactggagcattgcggcatcgacacagaaggcacctacaattccatcactgaaggattggattacgtattcatcacccacgatatacatctaactggggagattttttcattttttcggagtttcggacacccccgtctagaagcagtcaccgctgcagaaaatgtcaggaaacatatgaaccaaccgaaggtaatcagttatgagactatgatgaaagggcacgcagtattttgcgggataatcataaatggttttagggaccgacatggcggcagctggccccctgttgcattgccggaacatgcttctgctgcgatccggaatgcgcaggcatcaggtgagggactgacccatgacctgtgtatagacaattggaaatcctttgttggattcaaatttggctgctttatgccactcagcctagatagtgatttgactatgtacctcaaagacaaggcattggctgcactgaagaatgagtgggattcagtttacccgaaagaatacctccgttataacccacctagggggacagagtcaaggcgattggtggaggtgttcctgaatgactccagctttgatccttataacatgataatgtacgtggtgaatggctcctaccttaaagaccctgagtttaatctctcatacagcctaaaagagaaggagataaaggagacagggcggttgtttgccaaaatgacctataagatgcgggcctgtcaggtaatcgctgaaaatctgatatcgaatggtgttgggaagtatttccgagacaatgggatggcaaaggacgagcatgacctaacaaaagcccttcacactctggcagtctcaggtgttcccaagaacaacaaagataaccaccgaggtggccctcccagaagaacaacaagccgagaggtgagatcaagccaagacaccaaaacacaaaatagagacaaggtccaaggggggcctatgtacaactatttgcgatgccaaccaatcagccctgatcagggtgagtcatacgagactgttagtgcattcatcaccgctgaccttaagaagtattgcctgaattggagatacgagacaatcagcatatttgcacagaggctgaatgaaatatatggactaccatccttctttcaatggttacacaggatattggaaaaatccgtactctacgtcagtgacccacattgccctcccgacctagataatcatatccctctggacagtgtccctaatgcccaaatattcattaagtacccaatgggcggaatagaaggttattgccaaaaactatggacaatcagtactataccatacttgtatctggcagcctatgagagcggagtaagaatcgcctcactagtgcagggtgacaatcagacaatcgcagtgacaaaaagagttccaagttcttggccttattcactaaaaaagagggaggcatccaaagccgctcaaaattacttcgtagtcttaaggcaaagattgcacgatgtaggtcatcacttaaaggctaatgaaaccatagtatcttctcacttttttgtatattccaaagggatttattatgacggcctgctagtctcacaatcactaaagagcatcgccagatgtgtcttctggtccgagactatagtggatgaaaccagagcggcctgcagcaatattgcaacaactgtcgctaagagtatagagaggggttatgataggtaccttgcatattctttgaatatcctcaagattttccaacagatccttatatcccttaacttcactattaacacaacaatgactcaggatgtcgtggcaccgatcatcgagaacggtgatttgctgataaggatggcactcttgccagcacccatcgggggtctcaattatcttaacatgagcaggttatttgtgagaaatatcggtgacccggtcacctcctctatagccgacctgaagaggatgatagatgctgggctaatgccagaagaaacattgcatcaagtgatgacccagaccccgggagaatcatcctacctcgattgggcaagtgacccttattctgccaacctaccctgcgtacagagtataactcgccttctcaaaaacatcactgcacgatatattttaatcagcagcccaaacccgatgctgaaaggattgtttcatgaggggagtagagatgaagacgaggagcttgcaagtttcctaatggatcggcatataattgttccaagagctgcacatgaaatcttagaccatagcataaccggggcaagagaagctatagccgggatgttggacaccaccaagggtctgattagaacaagtatgaaacggggtggcctcacccctcgagtcttagcccgcctttccaattatgattatgaacaattccgatccgggataacactattgacaaagaaagggcagtgttatctcattgacaaggactcgtgctcggtgcagctcgctatagctctgaggagccacatgtgggctaggttggctcgcgggagaccaatctatgggttggaggtgcccgatatattagaatcgatgaacggctaccttatcaaacgccacgagtcttgtgctatctgtgagacgggctcaagtcactacggatggtttttcgtccctgcagggtgtcagcttgacgatgtatctagagagacttcggctcttcgtgtaccttatgtcggatcaacaactgaggaaaggacagacatgaaacttgcatttgttaggtctccaagtcgatccctcaagtcagcagtcagaattgcaacagtttactcatgggcctacggggatgatgagaaatcatggagtgaagcctggatgctagctaggcagagagccaatatcaccttagatgaattaagaatgatcactccagtctctacatccaccaacctagcccaccggttgagggatcggagtacccaggtgaaatactcggggacatcccttgtgagggttgcaagatacacaaccatctccaatgataacttgtcatttgtgatatctgagaaaaaagtggataccaacttcatttatcagcaagggatgctgctcggtcttgggatccttgagaacctctttaggctagaggccaccaccggagtatccaacacagtgctacacctgcacgtggaaacagaatgttgtgttgtacctatggtggatcacccaaggataccgagtctccgtaatataaaagtgacggatgagctatgcacaaaccctttgatctacgacaggtctcccatcatagagcacgatgcaacccgactatactcacagagccacaggagacatttggtggagtttgttacctggtcaacaagccagctttaccatatactggctaagtctacagcaatgtccatgattgagttgatcacgagattcgagaaagatcacatgaatgaaatagccgccctgattggcgatgacgacatcaacagcttcatcacagaatttttgctagtagagcctagattatttatagtttaccttggtcagtgtgctgccatcaattgggcctttgatatacattatcatcgcccctcgggcaagtaccagatgggggaactcctctactctctgctctctcggatgagcaaaggagtatataagatcttcaccaatgctttgagccaccccaaagtttacaagaaattttggcgaagtggtgtcattgagccgattcatggcccatccctggatacacagaatttacatgtcactgtctgtgacatgatatatgggtcctatgtcacctatttggatcttttgctgaatgatgagctagatgattatccgtatttgctctgcgagagtgatgaggacgtggtcacagacaggttcgacaacattcaagccaaacatctctgtgtattggccgatgtatattgcagctccaagaggtgtccttcgataatcgggatgtctcctatagaaaaatgtaccattctcacacattacatcaagggagaatcagtacaatccccgtctgggacctcatggaacactgatccccttgtagtagatcattactcatgctctctgacctaccttcgccgcggttccatcaaacaaatcaggttgagagtggatcctgggtttgtattcgaggcgttgacagacgtcgactttaaacaacctcgcaaagctaagttagatatatcggtcattgggttgactgatttctctcccccttgcgacaacgtcggtgattttctagggactatcaacacattgaggcacaatctgcctgtcaccggaaccggggtctcgaactatgaagtccacgcttatcgtagaattggcctgaattcatcagcatgttataaagccgtagagatctccacgttaatcaagccatccttagaagtcggagagcatggattgttcttaggagaaggttccggttcaatgctggctgcgtacaaggaagttcttaaattagcaaattgttattacaacagcggggtaacagcggagggcagagccggacagagggaaatatctccctatccttcagagatgagcctagtagagaatcaaatggggatagagcggagtgttaaagtgctgttcaacggcaaacctgaagtaacttgggtggggaccaccgattgctacaagtacataattagtaacatccaaacctctagtctgggtttcatacattcagatattgagacactcccaaccaaagatgctgttgagaaattagaagaatttgcctctatcctatccttatccttgattttgggaaaaatcggctctattacagttgtcaaaattatgcccattagcggggattttacccaaggcttcatagcctatgccatccaatattttagggagagcctgcttgcctatccgagatatagtaacttcatctcgactgagtgttaccttattatgataggattaaaggccaatcgattgataaacccagaagccattaagcaaagcataatcagagcgggggtcaggactgcaccaggacttgtgagccatatattatcagagaaacagaaaggttgtattcaatctttcttgggtgatccttatatccaaggggacttcaataagcaccttaaatctctaacccctattgagaaaatcctggtaaattgtggtctctcgatcaatggcacaaaaatctgtagagatctactccaccatgatatcgcctccggtccagacggtcttatgagctctacaattattttatatagggaattggctcatttcaaagataatataaggagtcagcacggaatgttccacccctacccagtactggccaacagcaggcaacgtgaattaatccttcgaatagccaagaaattctgggggtatgtcttgctatattctgatgacccggcactaatcagacaaacaatcaagaacttgaaacggaatcatctaacctttgacttacatagtaacccgtttattaaagggctatccaaagctgagaaactgctggtgcggacgagttcactaagaagggaatggttgttcactctcgatacgaaagaagtgaaagagtggttcaaattggtaggttacagtgcactcatcagaggctagttggctatgcatctgcccccttttcctccgccataagaccccactgatgatccaaaagattaaagaaaactacatattggataagtatctattcccagctttgtctggt

>KR781449/Benin/Tchaouro/2011/10

accaaacaaagttgggtaaggatagatcttacaatgactatagactggcaaacttaggagtaaagatcctactgtcggggagaggaggaggagcaagatctttgaccatggcgactctcctcaaaagcttagcactgttcaagaggaacaaagacaaagcgcctactgcgtcaggttcaggaggggccatccgggggattaagaatgttatcatagtccctatccccggggactcatccatcattacccgttcaagactgctcgacaggcttgtcagactggccggagatcctgacatcaacgggtcaaagctgaccggcgtgatgatcagcatgttatctttgttcgtggagtcacccgggcaattgatacagcggatcacagatgatccagatgttagcatccgtcttgttgaggtagttcaaagtaccaggtcccagtccggattgacctttgcatcacgtggtgctgatttggataatgaggcagatatgtatttttcaactgaaggaccctcgagtggaagtaagaaaaggatcaactggtttgagaacagagaaataatagacatagaagtgcaagatgcagaagagttcaatatgttgttagcctccatcttagcacaagtttggatcctcctggccaaggcggttacggcaccagatacggcagccgactcagaactgagaaggtgggttaaatacacacagcaaaggagagtgattggggaatttcgctttgacaaagggtggctggacgcagtccgtaacaggattgcagaagatctatcactccggcggttcatggtatccctgatacttgacatcaagaggacccccggcaacaagccaaggattgcagaaatgatctgcgacattgacaactatattgtcgaggccggactcgcaagtttcattcttactatcaagtttggtattgaaaccatgtatcctgcactaggccttcacgagttcgcaggggaattgtccactattgaatccttgatgaacttgtatcaacagctaggagaagttgcaccctatatggtaattctagagaactcaattcagaacaagtttagtgcaggagcctatcctctcctctggagctatgcgatgggtgtcggagtcgagttggagaactcaatggggggcttgaactttggcaggtcatattttgacccggcctattttcgtctcggacaggagatggttaggagatctgcaggaaaggtcagctctgtaattgcggctgagcttggcatcacagcagaggaagccaaactagtctcggaaatcgcctcgcagactggggaagaacgaaccgttagagggactgggcctcggcaggcgcaggtttccttcctccagcacaaaatgggtgagggagagtcgcctacaccagcgaccaaagaagaagtcaaagctgcgatcccaaacgggtccgaaggaagggacataaagcgaacacgcccagggaagcccagaggagaaactcccggccaactgcttctggagatcatgccagaggatgaagtctcgcgagagtctggtcaaaaccctcgtgaggctcaaagatcggctgaggcacttttcaggctgcaggccatggccaagattctggaggaccagggggagggagaagacaacagtcagatctacaacgacaaggatctcctcagctgagcagatacaccctccgtccgaatcagtgacaagacatcacctgccagtattataaaaaacttaggacccaggtccaaccaaccgcacatcgacaccccagtcaatcgagcggagaccaccgatggcagaagaacaagcatatcatgtcaacaaggggctggaatgtatcaagtccctcaaagcctctcccccggatctatccaccatccaagatgcccttgagagctggagagaggggtttaacccctcaggccgtgcaacaccgaaccctgatacgtccgagggggaccatcagaatatcaaccaatcatgctcatcagcaatcggatcagacaaagtcgacatgtctcctgaaggtaatctcggatttagagagatcacttgtgatgacaatgagactgggctcggaggagttcaggacaaaggatccgactctcaagtacagcgttactatgtttataaccacgggggtgaagagattgaaggactcgaggatgctgactctctcgtggttcaagcaaatcctccggttgctaacaccttcgatggaggagaggatggatctgacaacagcgatgtggactctggcccagatgatcccggcagagatcctctatatgaccggggacctgctgccggcaatgatgtctctaggtcaacagatgtcgaaaaattagaaggtgatgacattcaagaagttcttaactcccagaggagtaaaggaggaagattccagggagggaaaatcttgcgagtcccggaaatacccgatgtcaagaactccagaccatcggcccaatcaattaaaaagggcacagacgggagctcagtcttatctggaacggtgacagagtattcatcgataagtggtgcaacccaagctgtgctagagtccagatgggagtcatcagagcgaaatgcatctgtggggagtgtccccaaatctgcgaggagtgcagagacgatccaggggttaacacaagaatctggtaccatagcatcactgactcagcctaaagaaaatgactccgagtatgagtatgaggatgagttattcactgagatgcaggagattcgtgcaagcattgctaagatccatgatgacaacaaaactatcctctcaaaacttgactctctgctgttactgaaaggagaaatcgacactatcaagaaacaaatcagcaaacaaaatataagtatatctaccattgagggccatctatccagtataatgatagccatcccaggttttgggaaggacatcaaggacccaacatctgaggttgagttgaacccagatttgagacctataatcagccgtgattctggcagggctcttgcggaggtccttaagaaacccgctgttgataggtctcagaagagcggaatcaaagccaactccggttcaaagggtcagatcctcaaggatctccagctaaagcccgtcgacaagcaggcaagctctgcaatcgggttcgttccatccgaccatgaatcatccagaagtgtcatccgctccataatcaagtcaagcaagcttaacattgatcacaaagactatcttctagatttactgaatgatgtgaaaggatctaaggatctcaaggaattccacaagatgctaacagccattcttgccaagcagccgtaacacattccacaatctatatctcatactcggttgaaagcatcctctcaaccaggctattacaaaaaacttaggagcaagggcaactgagcttcacagacaggatgaccgagatctatgatttcgataaatcagcatgggatgtcaaagggtcaattgctcgcatagaacccaccacctatcacgacggccgactggtaccccaggtgagggtcatcgaccctggtctgggagacagaaaagatgagtgcttcatgtatctgtttcttctaggagtgattgaggataacgaccccctttctcccccagtcgggagaaccttcgggtctttacctctaggtgtcggtaggtcaactgctaagccagaggaactactaagggaggccacagaactagatatagtggtgaggcgcacggcaggactaaatgagaaactggtattttacaacaacactccgctatctttgttaacaccctggaggaaagtcttgacgaccgggagtgtgttcagcgccaaccaggtttgcaatgcggtcaacctagtcccactcgatactccccagagattcagggttgtgtacatgagtataactagattgtcagataatggttattatagtgtccccagaagaatgttggagttccgctcagccaatgcagtcgctttcaatatcttggttacactgagaattgaaaatggcacaaaccctagaagatacatagtcggctcatgggagaatccggaggtcacatttatggtacacgtgggcaactttagaagaaagaagaacgaagtatactctgctgattattgcaaaatgaagattgaaaagatgggtctagtttttgccttgggaggaataggcggaacaagtctccatattcgaagcacagggaaaatgagcaagaccctccatgcacagctggggttcaagaaaattctatgctatcctctgatggatatcaatgaggatcttaaccgatatctctggcgggcagagtgccgaatagtcaaaatccaggccgtcttacagccgtcagtaccccaagaattccgtgtctatgatgatgtcatcatcaacgatgaccaaggcctgttcaagatcctgtaagtcacctgcaacatcatgacacgggtcgtaaccttggtatttctgtttcttttcccaaacactgtctcgtgccagattcactggggcaatctatctaagatagggattgtaggaacagggagtgccagttacaaggtgatgactaggccaagccaccaaactctggttataaagttgatgccaaatataacagccatcgacaattgtacgaaatcagagatttcagagtacaaaagattgctaatcacagtgttaaagcctgtagaggatgctctgtcagtaataaccaagaatgtaagaccaattcaagctctaacacctgggcgcaggacccgccgtttcgccggagctgttctggccggagtagcacttggagtcgcgacggctgctcaaataactgccggagtcgcactccatcagtcattgatgaattcccaagcaattgaaagtttaaaaaccagtcttgagaagtcaaatcaggcaatagaagaaatcagacttgcaaataaggagaccatactggcagtacagggcgtccaagactatatcaacaacgagcttgtcccctctgttcatagaatgtcatgtgagctaataggtcacaaactcagtctcaaactccttaggtattataccgagatcctgtctatattcgggcctagccttcgagacccgatagctgctgaaatatcaatccaggcactcagctatgcactaggcggagacatcaataaaattctggacaagcttgggtatagcggcggggatttccttgctattctagaaagcaaggggataaaggcccgtgtcacatatgtggacacaagagattactttataattcttagcatagcctacccaaccttatctgagatcaaaggggtgatagttcataagatagaagctatatcatacaacattggggcacaggaatggtatactactatccctaaatatgtagccactcagggatatctgatatcaaatttcgatgagacgtcatgcgtcttcactccagaggggacagtctgcagccagaatgcgttgtatccaatgagcccattgcttcaggaatgtttcagggggtcgacaaaatcgtgtgccagaaccctagtttcagggaccacaagcaatagatttatcctatcaaaagggaacttgattgcaaattgtgcgtcagttttgtgcaagtgttacacaacggagacagtcatcaaccaagatcctgataaactactaactgttatagcctcagataagtgtcccgtagttgaggtggatggagtgacaatacaagtcggcagtcgagagtacccagattctgtatacctacataaaatagacttaggcccagccatctctctggaaaaactggacgtaggcaccaatttaggcaatgcagtcacaagactggagaatgcaaaggagctcctagacgcatcagaccagatactgaagactgttaaaggggtacccttcagtggcaatatgtacatagcactggcagcttgcattggggtatccctagggcttgtcacattaatatgctgctgtaaggggagatgtaggaacaaggagattcctgcctccaaaatcaacccagggctcaaacccgacctgaccgggacttcaaagtcgtacgtgagatcactgtagtcagaataacccgaatcatccggcatcacgcgtatacatgtgcgacacaagcagtcagaggacgcagaagactcaacctccgatcaccgaccagaccccactctacgccctactacacattggtcatcaaacaaaacttaggacgaaaggtcaatcaccatgtccgcacaaagggagaggatcaatgccttctacaaagacaaccctcacaataaaaaccataggataatcctggatagggaacgcttaactattgaaagaccctacatcttacttggagtcctgctggtaatgttcctgagtctaatcgggcttctagccattgcagggatcaggcttcaccgggccaccgttgggactgcggagatccagagtcggctgaataccaacattgagttgaccgaatccattgatcatcaaactaaggatgtcttaacacccctgtttaaaatcattggtgatgaagtcggcatcagaattccacagaagtttagtgatcttgtcaagttcatctccgataagattaagttcctcaaccctgacagagaatacgattttagggatctccggtggtgtatgaaccctcctgagagagtcaaaattaactttgatcaattctgtgaatacaaagccgcggtcaagtcagttgaacatatatttgagtcatcattcaacaggtcagaaagattgcgactattgactcttgggcccggaacaggctgtctcggcagggcagtaacaagagctcagttctcagagcttacgctgaccctgatggacctggatctcgagatgaagcacaacgtgtcctcagtgtttaccgtagttgaagaggggttattcggaagaacatatattgtctggagatctgacaccaggaaaccgagcaccagtccagttattggccagtttttaagagtcttcgagatcgggttggtgagagatctcgagctgggtgcccccattttccatatgaccaactacctcacagtgaacatgagtgatgactatcggagttgccttttagcggtaggggagttgaagctgacagccctatgcactccatctgagactgtgactctgagtgagagaggagttccaaagagagagcctcttgtggttgtgatactcaacctagttgggcctactctagggggcgaactatacagtgtcttgcctacctctgacctcatggtggagaaactctatttatcctcacatagagggataatcaaagataacgaggccaattgggtagtgccgtctaccgatgttcgtgatctccaaaacaaaggggaatgtctggtggaagcatgcaaaactcgacctccttcattttgcaatggcacaggaataggcccatggtcagaggggagaatccctgcctacggagtgatcagggtcagtcttgacttagctagtgacccaggtgtggttatcacttcagtgtttggcccactgatacctcacctatctggcatggatctttacaacaatccgttttcaagaggtgcatggctggctgtaccaccctatgagcagtcatttctaggaatgataaatacaattgacttcccggacagagcagaggtcatgccgcacattttgaccacagagatcagagggcctcgaggtcgttgccatgttcctatagagttgtcccgcaggattgatgatgatatcaagatcgggtccaatatggttgtattgccgacgagggatctgaggtacgtaacagccacttatgatgtttccaggagcgagcatgcaatcgtgtactatatctatgacacgggtcgctcatcatcttacttctacccagttcgattgaatttcaggggcaatcctctctctctgaggatagagtgtttcccctggtctcataaggtgtggtgctaccatgattgtcttatatacaacaccatgacaaacgaagaagtccatacgagagggctgaccggtatagaggtaacatgcaatccagtctgagtcgagctgaaaccatcgctcaagcaggcttccgagccatcccctagtccaagcagcatagtctgggacactcagcagcacaacccagccaacaatgttataaaaaacttaggagccaaggttgtatgagccatggactcactatcagtcaatcaggttttgtaccctgaggtccatctagatagccctattgttacaaacaaactagttgctatccttgaatactcggggatcaaccacaactatgttcttgaagaccagacccttatcaagaatattagatatagactggggtgcggtttttcaaatcaaatgatcatcaataataggggggtaggtgaaacagtcaattccaaacttaaaagttacccccgtaactgtcatatcatatacccagactgcaataaggagttgttttgtatcaaagatagctgcatatctagaaagctctcggagctattcaagaagggtaattccttgtactctaaggtgagtcaccaggtactggattgtctcaagagagtcaacgggaaactagggctgggcacagatcttactcacggcctgaaggatggtatcctcgacttggggttgcacatgcatagctctcaatggttcgagacctttctgttctggttcactatcaagacagagatgagatcaatgatcaaagaacagtcccatatatgccacaagaggaggtataacccaatttttgtgtcgggggatgcattcgaggtgcttgtatcacgagacctcgtagtgataattgataagaacacccagtatgtcttctacctgacatttgagctggtccttatgtattgtgatgtcatagagggcagacttatgacggagacagccatggctatagaccagagatattcagagcttctaaaccgggtcagatacttgtgggaccttatcgatgggtttttcccaacactgggtaacaccacataccaagttgttgctctgcttgaaccactgtcgttggcttatcttcaacttcaggatgtcactctagagttaagaggtgcctttttggaccactgcttcaaagaactttatgagatactggagcattgtggcattgacacggaaggtacctacaattccatcactgagggattggattacgtatttatcactcacgatatacacttaactggggagattttttcatttttccggagtttcggacacccccgtctcgaagcggtcaccgctgcagagaatgtcagaaaacatatgaaccaaccgaaggtaatcagttatgagactatgatgaaagggcatgcagtattttgcgggataatcataaatggttttagggaccggcacggcggcagctggccccctgttgcattgccagaacatgcttctgctgcgatccggaatgcgcaggcatccggcgaaggactgacccacgacctgtgtatagacaactggaagtcctttgtaggattcagatttggctgcttcatgccactcagcctagatagtgatttgaccatgtacctcaaagacaaagcactggctgcactgaagaatgagtgggattcagtttacccgaaagaatacctccgttataatccacctagagggacagagtcaaggcgactagtagaggtgttcctgaatgactccagctttgatccttataacatgataatgtacgtggtgaatggctcctaccttaaagaccctgagtttaatctctcatacagcttaaaagagaaggagataaaagagacagggcggttgtttgccaagatgacctacaagatgcgggcctgtcaggtgattgctgaaaatctgatatcaaatggtgttgggaagtatttccgagacaatgggatggcaaaagacgagcatgacctaacaaaagcccttcacactctggcagtctcgggcgttcccaagaacaacaaagacaaccaccgaggcgggccccccagaaggaacgcaagccgagagacgagatcaagtcaagccgtcaacacacaaaatagagacaagatccaagggggccctatgtacaactacatgcgatgccaaccaaccagccctgatcagggtgagtcatacgagactgttagtgcattcatcaccgctgaccttaagaagtattgcctaaattggagatacgagacaatcagcatatttgcacagagactgaatgaaatatatgggttgccatccttctttcaatggttacataaggtattggaaaaatccgtgctctacgtcagtgatccgcattgccctcccgacttagatgatcatatccctctggacagtgtccctaatgcccaaatattcatcaagtacccaatgggcggaatagaaggttattgtcaaaaattatggacaatcagtactataccatacttgtatctagcagcctatgagagtggagtaagaatcgcctcactagtgcaaggtgacaatcagacaattgcagtgacaaaaagagttccaagttcttggccttattcactaaaaaagagggaggcatctaaagcagctcaaaattactttgtggtcttaaggcaaaggttgcacgatgtaggtcatcacttaaaggctaatgagaccatagtatcttctcacttttttgtatactctaaagggatttattatgacggcctgttagtttcacaatcactaaagagcatcgccagatgtgtcttctggtccgagactatcgtggatgaaactagagcggcctgcagcaatattgcaacaactatcgctaagagtatagagaggggttatgatcggtacctcgcatactctttgaatatcctcaaaattttccaacagatccttgtatcccttgacttcacgattaatacaacaatgactcaagatgtcgtggcaccgatcattgagaacggtgatttactgataagaatggcactcttgccagcacccattgggggtctcaattatctcaacatgagcaggttgtttgtgagaaatatcggtgacccggtcacttcctctatagccgacctgaagaggatgatagacgctgggctaatgccagaagaaacattgcatcaagtgatgacccagaccccgggagaatcatcctaccttgattgggcaagtgatccttattctgccaacctaccctgcgtacagagtataactcgccttctcaagaacatcactgcacggtacattttaatcagcagcccaaacccgatgctgaaagggttgtttcacgaggggagtagggatgaagacgaggagcttgcgagtttcttaatggatcggcatataattgttccgagagctgcacatgaaatcttagaccacagcataaccggggcaagggaagctatagccgggatgttggacaccaccaagggtctgattagaacaagtatgaaacggggtggcctcactcctcgagtattggcccgcctttccaattatgattatgaacaattcagatccgggataacactattgacaaagaaagggcagtgttatctcattgacaaggactcgtgctcggtgcagctcgctatagctctgagaggccatatgtgggcaagattggcccgcgggagacctatctatggcttggaggtgcctgatgtactggaatcgatgaacggctaccttatcaaacgtcacgagtcttgtgccatctgtgaaacgggctcaagtcactacgggtggtttttcgtccctgcagggtgtcagcttgacgatgtttcaagagagacttcagctcttcgtgtaccttatgtcggatcaaccactgaggaaaggactgatatgaaacttgctttcgttagatctccaagccgatccctcaaatcagcagtcaggattgccacagtctactcatgggcctacggggatgatgagaaatcatggagtgaagcttggatgctagctaggcagagagccgatatcaccttagatgaactgagaatgatcactccggtctctacatccaccaacctagctcatcggttaagggatcggagcactcaggtgaaatattcggggacatcccttgtgagggttgcaaggtacacaaccatctccaatgacaatttgtcatttgtgatatctgagaaaaaagtagatactaacttcatttaccaacaagggatgctgctcggtcttgggatccttgagaatctcttcaggttagaggccaccacaggggtatccaacacagtgctacacctgcacgtggaaacagaatgttgtgttgtacctatgattgatcacccaaggataccgagtctccgcaatatcaaagttacaaacgagctatgcacaaaccctcttatctacgacaagtcccccatcatagaacacgatgcaactcgattatactcacaaagccataggagacatttggtagagtttgttacctggtcaacaagccagctctaccatatactggccaaatctacagcaatgtccatgattgagctgatcacaagatttgagaaagatcacatgaatgaaatagccgctctgattggtgatgacgacatcaacagtttcatcacagaatttttgcttgtggagcccagactgtttatagtttacctcggccagtgtgccgccatcaattgggcttttgatatacattatcatcggccctcgggcaagtaccagatgggggaactcctctactctttactctctcggatgagcaagggagtatataagatcttcactaatgctctgagccaccccaaagtttacaagaaattttggcgaagtggtataattgagccggttcatggcccatccctagatacacagaatttacatgtcactgtctgtgacatgatatacggatcatacgtcacctatctggatcttttgctgaatgatgagctggatgcttacccgtatttgctctgcgagagtgatgaggacgtggtcacagacaggttcgacaacattcaagccaaacatctctgtgtactggccgatgtatactgcagctccaagagatgtccctcgataatcgggatgtcccctatagaaaaatgtaccatcctcacacattacatcaagggagaatcggtacaatccccgtccgggatctcatggaacactgatccccttgtagtagatcattactcatgctctctgacctaccttcgccgtggttccatcaaacaaatcagattgagagtggatcctgggtttgtgttcgaggccttgacagacatcgactccaaacagcctcgcaaggctaagttggacgtatcgattgtggggttgactgatttttcccccccttgggataacgtcggtgattttctagggactatcaacacattgaggcacaatctgcccgtcaccgggaccggggtctcgaactatgaagtccacgcttatcgtagaattggtctgaattcatcagcatgttataaagctgtagagatctccacgttaataaagtcatccttagaggtcggggagaatggattgttcttaggagaaggctccggctcgatgctggctgcgtacaaggaagttcttaaattggcaaactgttattacaacagtggggtaacagcagagggtagagccggacagagggaaatctctccctatccctcagaggtgagcctggtagagagtcagatggggatagagagaagtgttaaagtcctgttcaatggcaaacctgaagtaacctgggtagggaccaccgattgctacaagtatataatcagtaacattcagacctctagtctgggtttcatacactcagatatcgagacactcccaaccaaggatgccgttgagaagttggaggaatttgcctctattctatctctatccctaattttgggaaaaatcggctctattgcagttgtcaaaattatgcccattagcggagattttacccaaggcttcatagcctatgccattcaatatttcagggagagcctgcttgcctatccgagatatagtaacttcatctcgactgagtgttaccttattatgataggattaaaggccaatcggttgataaacccagaaaccattaagcaaagcataatcagggtggggactaggactgcaccaggacttgtgagccacatattatcagagaaacagaaaggttgtattcaatcttttctgggtgatccttatgtccaaggagacttcaataagcaccttaaagctctaactcctattgagaaaatccttgtaaattgtggtctctcgatcaatggtacaaaaatctgtagggatctaatccaccatgatattgcctccggtccagacggtctgatgagctccacaattattttatacagggaactggcccatttcaaagacaatataagaagtcagcacggcatgttccacccctacccagtattggccagtagcaggcaacgtgaattgatccttcgaatagccaagaagttttgggggtatgtcttgctatattctgatgacccggcactaatcaaacaaacgatcaggaacttgaagcggaatcacctaacctttgacttacacagtaatccgtttattaaggggttatccaaagctgagaaactgctagtgcggacaagttcactcagaagggaatggttgttcactctcgacacgaaagaagtgaaagagtggttcaaattggtgggttacagtgcactcgtcagaggttaattcgcgatacatctgcccccttctcctccaccatgagactttactggcaatccaaaagattaaagaaaactacatattggataaggatctattcccagctttgtctggt

>KR781450/Benin/1969

accaaacaaagttgggtaaggatagatcttataataactatagactggcaaacttaggagtaaagatcctactgtcggggagaggaggaggagcaagatctttgaccatggcgactctccttaaaagcttggcattgttcaagaggaacaaagacaaagcgcctactgcgtcgggttcaggaggggccatccgggggattaagaatgttatcatagtccctattcccggggactcatccatcattacccgttcaagactgctcgacaggcttgttagattggccggagatcctgacatcaacgggtcaaagctgaccggcgtgatgatcagcatgttatctttgttcgtggagtcacccgggcaattgatacagcggatcacagatgatccagatgttagcatccgccttgttgaggtagttcaaagtactaggtcccagtccgggttgacctttgcatcacgtggtgctgatttggacaatgaggcagatatgtatttttcaactgaaggaccctcgagtggaagtaagaaaaggatcaactggtttgagaacagagaaataatagacatagaggtgcaagatgcagaagagttcaatatgttgttagcctccatcttagcacaagtttggatcctcctggccaaggcggttacggcaccggatacggcagctgactcagaactgagaaggtgggttaaatacacacaacaaaggagagtgattggggaatttcgccttgacaaagggtggctggacgcagtccgcaacaggattgcagaagatctatcacttcggcggttcatggtatctctcatacttgacatcaaaaggacccccggcaacaagccaaggattgcagaaatgatctgcgacattgacaactatattgtcgaagccggactcgccagtttcattcttactatcaagtttggtattgaaaccatgtatcctgcattaggccttcacgagttcgccggggaattgtccactattgaatccttgatgaacttgtatcaacagctaggagaggttgcaccctacatggtaattctagagaactcaattcagaacaagtttagtgcaggagcctatcccctcctctggagctatgcgatgggtgtcggagtcgagttggagaactcaatggggggcctgaactttggcaggtcatattttgacccggcctatttccgtctcggacaggagatggtcagaagatctgcaggaaaggtcagctctgtaatcgcggctgagcttggcatcacagcagaggaagccaaactagtctcggaaatcgcctcgcagactggagatgaacgaaccgttagagggactgggcctcgacaggcgcaggtttccttcctccagcataaaatagatgagggagagtcgcctacaccagcgaccagagaagaagtcaaagctgcgatcccaaatgggtctgaaggaagggacataaagcgaacacgctcaggaaagcccagaggagaaactcccggccaactgcttctggagatcatgccagaggatgaagtctcgcgagagtctagtcaaaaccctcgtgaggctcaaagatcggctgaggcactcttcaggctgcaggccatggccaagattctggaggaccaggaggagggagaagacaacagtcagatctacaacgacaaggatctcctcagctgagcagacgcaccctccgtctaaatcagtgacaagacatcgcccgccagtattataaaaaacttaggacccaggtccaagcaaccacacatcgacaccccagtcaatcgagcagagaccaccgatggcagaagaacaagcataccatgtcaacaagggactggaatgtatcaagtctctcaaagcctctcccccggatctatccaccatcaaagatgcccttgagagctggagagaggggcttagcccctcaggccgtgcaacaccgaaccctgatacgtccgagggagaccatcagaatatcaaccaatcatgctcaccagcaatcggatcagacaaagtcgacatgtctcctgaagataatctcggatttagagagatcacttgtaatgacagtgaggctgggctcggaggagttcaggacaaaggatccaactctcaagtacagcgttactatgtttatagccacgggggtgaagagattgaaggactcgaggatgctgactctctcgtggttcaagcaaatcctccagttgctaacaccttcaatggaggagaggatggatctgacgacagcgatgtggactctggcccagatgatcccggcagagatcctctatctgaccggggatctgctgccggcaatgatgtctctaggtcaacagatgtcgaaaaattagaaggtgatgacattcaagaagttcttaactcccagaagagtaaaggaggaagattccaaggcgggaaaatcttgcgggtcccggaaatacccgatgtcaagaactccagaccatcagcccaatcaattaaaaagggcacagacgggaactcagtcttatctggaacggtgacagagtgttcatcgataagtggtgcaacccaagctgtgccagagtccagatgggagtcatcagggcgaaatgcgtctgtggggagtgtccccaaatctgcgaggagtgcaaagacgatccaggggttgacacaagaatctggtaccatagcatcactgactcagcctaaagagaatgactccgagtatgagtatgaggatgatctattcacagagatgcaggagattcgtgcaagcattgctaagatccatgatgacaacaaaactatcctctcaaaacttgattctctactgttattgaaaggagaaatcgatactatcaagaaacaaatcagcaaacaaaatataagtatatctaccattgaggggcatctatccagtataatgatagccatcccgggctttgggaaggacatcaaggacccaacatctgaggttgagttgaacccggatttgagacctataatcagccgtgattctggcagggctcttgcggaggtcctcaagaaacccgctgttgataggtctcagaaaagcggaatcaaagtcaactccggttcaaagggtcagctccttaaggatctccagctaaaacctgtcgacaaacaggcaagctctgcaatcgggtttgttccatctgaccatgaatcatccaggagtgtcatccgctccataatcaagtcgagcaagcttaacattgatcacaaggactatcttctagatttactgaatgatgtgaaaggctctaaggatcttaaggaattccacaagatgctaacagccattcttgccaagcagccgtaacacatcctataatcaacatctcatactcggttgaaaacatcctctcaatcaggctattacaaaaaacttaggagcaagggcaactgagcttcacagacaagatgaccgagatctacgatttcgataaatcagcatgggatgtcaaagggtcaattgctcgcatagaacccaccacctatcacgacggccgactgataccccaggtgagggtcatcgatcctggtctgggagacagaaaagatgagtgcttcatgtacctgtttcttctaggagtgattgaggataacgaccccctgtctcccccagtcgggagaaccttcgggtctttacctctaggggtcggtagatcaaccgctaagccagaagaactactaagggaggccacagaactagatatagtggtgaggcgcacggcaggactaaatgagaaactggtattttacaacaacactccgctgtctttgttaacaccttggaggaaagtcttgacaaccggaagtgtgtttagcgctaaccaggtttgcaatgcagtcaacctagtcccacttgatactccccagagattcagggttgtgtacatgagtataactagattgtcagataatggttattatagtgtccccagaagaatgttggagttccgctcagccaatgcagtcgccttcaatatcttggttacactgagaattgaaaatggcacaaaccctagaagatacatagtcggctcatgggagaatccagaagtcacatttatggtacacgtgggcaactttagaagaaagaagaacgaagtatactctgctgattattgcaaaatgaagattgaaaagatgggtctagtttttgccttgggaggaataggtggaacaagtctccatattcgaagcacagggaaaatgagcaagaccctccatgcacagctggggttcaagaaaatcctatgctaccccctaatggatattaatgaggatcttaaccgatatctctggcgggcagagtgccgaatagtcaaaatccaggccgtcttacagccatcagtaccccaagaattccgtgtctatgatgatgtcatcatcaacgatgatcaaggcctgttcaagatcctgtaattcacttgcaacatcatgacacgggtcgcaaccttggtatttctgtttcttttcccaaacactgtcgcgtgccagattcactggggcaatctatccaagatcgggattgtaggaacagggagtgccagttacaaggtgatgactaggccaagccaccaaactctggtcataaagttgatgccaaatataacagccatcgacaattgtacgaaatcagagatttcagagtacaaaagattgctgatcacagtgttaaagcctgtagaggatgccctgtcagtgataaccaagaatgtaagaccaattcaaactctaacacctgggcgcaggacccgccgttttgccggagctgttctggccggagtagcacttggagtcgcgacggctgctcaaataactgccggagtcgcactccatcagtcattgatgaattcccaagcaattgaaagtttaaaaaccagtcttgagaagtcgaatcaggcaatagaagaaatcagacttgcaaataaggagaccatactggcggtacagggcgtccaagattatatcaacaatgagcttgtcccctctgttcatagaatgtcatgtgagctggtaggtcacaaactcagtctcaagctccttaggtattataccgagatcctgtctatattcgggcctagccttcgagacccgatagctgctgaaatatcaatccaggcactcagctatgcattaggcggagacatcaataaaattctggacaagcttgggtatagcggcggggatttccttgctatcctagaaagcaaggggataaaggcccgggtcacatatgtggacacaagagattactttataattcttagcatagcctacccaaccttatctgagatcaaaggggtgatagttcataagatagaagctatatcatacaacattggggcacaggaatggtatactactatccctagatatgtagccactcagggatatctgatatcgaatttcgatgagacgtcatgtgtcttcactccagaggggacagtctgcagccagaatgcgttgtatccaatgagcccattgcttcaggaatgcttcagggggtcgacaaaatcgtgcgccagaaccctagtttcagggaccacaagtaatagatttatcctatcaaaagggaacttgattgcaaattgtgcgtcagttttgtgcaagtgttacacaacggagacagttatcaaccaagatcctgataaactactaactgttatagcctccgataagtgtcccgtagtcgaggtggatggagtgacaatacaggtcggcagtcgagagtacccagattctgtatacctacataaaatagacttaggcccagccatctccctggaaaaactggatgtaggcaccaatttaggcaatgcagtcacaagactggagaatgcaaaggagctactagatgcatcagaccagatactgaagactgttaaaggggtacctttcagtggcaatatgtacatagcactggcagcttgcattggggtatccctagggcttgtcacattgatatgctgctgtaaggggagatgtaggaacaaggagattcctgcctccaaaatcaacccagggctcaaacccgacctgaccgggacttcaaagtcgtacgtgagatcactgtagtcagaataacccgaatcatccagcatcacacatatacatgtgcgacacaagcagtcagaggacgcagaagattcaacttccgatcaccgaccagaccccactctacgccctattacacattggtcatcaaacaaaacttaggacgaaaggtcaatcaccatgtccgcacaaagggaaaggatcaatgccttctacaaagacaatcttcataataagaaccatagggtaatcctggatagggaacgcttaactattgaaagaccctacatcttacttggagtcctgctggtaatgttcctgagtctaatcgggctgctggccattgcagggatcaggcttcaccgggccaccgttggaactgcggagatccagagtcggctgaataccaacattgagttgaccgaatccattgatcatcaaactaaggatgtcttaactcccctgtttaaaatcattggtgatgaagtcggcatcagaattccacagaagttcagtgatcttgtcaagttcatctccgataagattaagttcctcaaccctgacagagaatatgattttagggatctccggtggtgtatgaacccccctgagagagtcaaaattaactttgaccagttttgtgaatacaaagccgcggtcaagtcagttgaacatatatttgagtcatcactcaacaggtcagaaaggttgcgattattgactcttgggcccggaacaggctgtctcggcaggacagtaacaagagctcagttctcagagcttacgctgaccctgatggacctggatctcgagatgaagcacaacgtgtcctcagtgcttaccgtagtcgaagagggattattcggaagaacatatactgtctggagatccgacaccggaaaaccgagcaccagtccaggtattggccattttttaagagtcttcgagatcgggctggtgagagatctcgagctgggtgcccctattttccatatgaccaactacctcacagtgaacatgagtgatgactatcggagctgtcttttagcagtaggggagttgaagctgacagccctatgcaccccatctgagactgtgactctgagtgagagaggagttccaaagagagagcctcttgtggttgtgatactcaacctagctgggcctactctagggggcgaactatacagtgtattgcctacctctgaccccacggtggagaaactctatttatcctcacatagggggattatcaaagataacgaggccaattgggtagtaccgtctaccgatgttcgtgatcttcaaaacaaaggagaatgtctggtggaagcatgcaagactcgacctccttcattttgcaatggcacaggaataggcccatggtcagaggggagaatccctgcctacggggtgatcagggtcagtcttgacttagctagtgacccgggtgtagttatcacttcagtgtttggcccattgatacctcacctatccggcatggatctttacaacaatccgttttcaagagctgcatggttggctgtaccaccttatgagcagtcatttctaggaatgataaatacaattggcttcccggacagagtagaggttatgccgcacattttgaccacagagatcagagggcctcggggtcgttgtcatgttcctatagagttgtcccgcaggattgatgatgatatcaagatcgggtccaacatggttgtattgccgacgaaggacctgaggtacataacagccacttatgatgtttccaggagcgagcatgcaatcgtgtactatatctatgacacgggtcgctcatcatcttacttctacccagttcgattgaatttcaagggcaatcctctctctctgaggatagagtgttttccctggtatcataaggtgtggtgctaccatgattgtcttatatacaacaccataacaaacgaagaagtccatacgagagggctgaccggtatagaggtaacatgtaatccagtctgagtagagctgcaaccatcgctcaagcaggcctccgagctatcccctagtccaagcagcatagcctgggacactcagcagcacaacccagccaacaatgttataaaaaacttaggagccaaggttgtaggagccatggactcactatcagtcaatcaggtcttgtaccctgaggtccatctagatagccctattgtcacaaacaaactagttgccatccttgaatactcggggatcgaccacaactatgttcttgaagaccagacccttatcaagaatattagatatagactggggtgcggtttttcaaatcaaatgatcatcaataataggggggtaggtgaaacagtcaattccaaacttaaaagttacccccgtaattgtcatatcatatacccagactgcaataaggatttgttttgtatcaaagatagctgcatatctaggaagctctcggagctattcaagaagggtaattccttgtactctaagataagtcaccaggtactggattgtcttaagagagtcaacgggaaattaggcctgggcacagatcttactcacggcctgaaggagggtatcctcgacttggggttgcacatgcatagctctcaatggttcgagacctttctgttctggttcactatcaagacagagatgagatcaatgatcaaagaacagtcccatatatgccacaagaggaggtataacccaatttttgtgtcgggggatgccttcgaggtgctcgtatcacgagacctcgttgtgataattgataagaacacccagtatgtcttctacctgacatttgagctggtccttatgtattgtgatgtcatagagggcagacttatgacggagacagccatggctatagaccagagatattcagagcttctaaaccgggtcagatacttgtgggatcttattgatgggttcttcccaacactgggtaacaccacataccaagttgttgctctgcttgaaccactgtcattggcttatcttcaacttcaggatgtcactctagagttaagaggtgcttttttggaccactgcttcaaagaactttatgagatactggagcattgtggcattgacacggaaggtacctacaattccatcactgaaggattggattacgtatttatcacccacgatatacacttaactggggagattttttcattttttcggagtttcggacacccccgcctcgaagcggtcaccgctgcagagaatgtcagaaaacatatgaaccaaccgaaggtaatcagttatgagactatgatgaaagggcatgcagtattttgcgggataatcataaatggttttagggaccggcacggcggcagctggccccctgttgcattgccagaacatgcttctgctgcgatccggaatgcgcaggcatccggtgaaggactgacccatgacctgtgtatagacaactggaagtcctttgttggattcagatttggctgcttcatgccgctcagcctagatagtgatttgaccatgtacctcaaagacaaagcactggctgcactgaagaatgagtgggattcagtttacccgaaagaatacctccgttataatccacctagagggacagagtcaaggcgactggtagaggtgttcctgaatgactccagctttgatccttataacatgataatgtacgtggtgaatggctcctaccttaaagaccctgagtttaatctctcatacagcttaaaggagaaggagataaaagagacagggcgattgtttgccaaaatgacctacaagatgcgggcctgtcaggtaattgctgaaaatctgatatcaaatggtgttgggaagtatttccgagacaatgggatggcaaaagacgagcatgacctaacaaaagcccttcacactctggcagtctcaggtgttcctaagaataacaaagataaccaccgaggtggacctcccagaaggaccacaagccgagagatgagatcaagccaagacatcaacacacaaaatagagacaagatccaagggggccctatgtacaactacttgcgatgccaaccgaccagccctgatcagggtgagtcatacgagactgttagtgcattcatcaccgctgaccttaagaagtattgcctaaattggagatacgagacaatcagcatatttgcacagagactgaatgaaatatatgggttgccatccttctttcaatggttacacaggatattggaaaaatccgtgctctacgtcagtgatccgcattgccctcccgacttagatgatcatatccctctggacagtgtccctaatgcccaaatattcatcaagtacccaatgggcggaatagaaggttattgtcaaaaactatggacaatcagtactataccttacttgtatctggcagcctatgagagcggagtaagaatcgcctcactagtgcaaggtgacaatcagacaattgcagtgacaaaaagagttccaagttcttggccttattcactaaaaaagagggaggcatccaaagcagctcaaaattacttcgtggtcttaaggcaaaggttgcacgatgtaggtcatcacttaaaggctaatgagaccatagtatcttctcatttttttgtatactctaaagggatttattatgacggcctgttagtctcacaatcactaaagagcatcgccagatgtgtcttctggtccgagaccattgtggatgaaaccagagcggcctgcagcaatattgcaacaactatcgccaagagtatagagaggggttatgataggtacctcgcatactctttgaatatcctcaaaattttccaacagatccttatatctcttgacttcacgattaacacaacaatgactcaagatgtcgtggcaccgatcatcgagaacggtgatttactaataaggatggcactcttgccagcacccattgggggtctcaattatcttaacatgagcaggttatttgtgagaaatatcggtgacccggtcacttcctccatagccgacctgaagaggatgatagacgctgggctaatgccagaagaaacattgcatcaagtgatgacccagaccccgggagaatcatcctacctcgattgggcaagtgacccttattctgccaacctaacctgcgtacagagtataactcgccttctcaagaacatcactgcacggtatatcttaatcagcagcccaaatccgatgctgaaaggattgtttcatgaggggagtagagatgaagacgaggagcttgcgagtttcttgatggatcggcatataattgttccgagagctgcacatgaaatcttagaccatagcataaccggagcaagagaagctatagccgggatgttggacaccaccaagggtctgattagaacaagtatgaagcggggtggcctcacccctcgagtattagcccgcctttccaattatgattatgaacaattcagatccgggataacgttattgacaaagaaagggcagtgttatctcattgacaaggactcgtgctcggtgcagctcgctatagctctgaggggccacatgtgggccaggttagctcgcgggagacctatctatgggttggaggtgcctgatatactggaatcgatgaacggctaccttatcaaacgccacgagtcttgtgccatctgtgaaacgggctcaagtcactacgggtggtttttcgtccctgcagggtgccagcttgacgatgtctcaagagagacttcggctcttcgtgtaccttatgtcggatcaaccactgaggaaaggacagatatgaaacttgctttcgttagatctccaagccgatccctcaaatcagcagtcagaattgccacagtttactcatgggcctacggggatgatgagaaatcatggagtgaagcttggatgctagctaggcagagagctgatatcaccttagatgaattgagaatgatcactccagtctctacatccaccaacctagcccatcggttgagggaccggagcacccaggtgaaatattcggggacatcccttgtgagggttgcaagatacacaaccatctccaatgacaatttgtcatttgtgatatctgagaaaaaagtagataccaacttcatttaccagcaagggatgctgctcggtcttgggatccttgagaatctcttcaggttagaggccaccacaggggtatccaacacagtgctacacctgcacgtggaaacagaatgttgtgttgtacccatggttgatcacccaaggataccgagtctccgtaatattaaagttacgaatgagctatgcacaaaccctctgatctacgacaggtcccccatcatagaacacgatgcaactcgattatactcacaaagccacaggagacatttggtggagtttgttacctggtcaacaagccagctttatcatatactggccaaatctacagcaatgtccatgattgagttgatcacaagatttgagaaagatcacatgaatgaaatagccgccctgattggcgatgacgacatcaacagtttcatcacagaatttttgctagtagagcccagactgtttatagtttaccttggccagtgtgctgccatcaattgggcttttgatatacattatcatcggccctcgggcaagtaccagatgggggaactcctctactctttactctctcggatgagcaaaggagtatataagatcttcactaatgctttgagccaccccaaagtttacaagaaattttggcgaagtggtgtaattgagccgattcatggcccatccctagatacacagaatttacatgtcactgtctgtgacatgatatacggatcatacgtcacctatctggatcttttgctgaatgatgagctagatgattacccgtatttgctctgcgagagtgatgaggacgtggtcacagacaggttcgacaacattcaagccaaacatctctgtgtactggccgatgtatactgcagctccaagagatgtccctcgataattgggatgtctcctatagaaaaatgcaccatcctcacacattacatcaagggagaatcggtacaatccccgtccgggatctcatggaacactgacccccttgtagtagatcattactcatgctctctgacctaccttcgccgcggttccatcaaacaaatcaggttgagagtggatcctgggtttgtattcgaggcgttgacagacatcgacttcaaacagcctcgcaaggctaagttggatgtatcggttgtggggttgactgatttttctcccccttgggataacgtcggtgattttctagggactatcaacacattgaggcacaatctgcccgtcaccggaaccggggtctcgaactatgaagtccacgcttatcgtagaatcggcctgaattcatcagcatgttataaagctgtagagatctccacgttaatcaagtcatctttagaagtcggagagaatggattgttcttaggagaaggttccggttctatgctggctgcgtacaaggaagttcttaaattggcaagctgttattacaacagcggggtaacagcggagggcagagccggacagagggaaatctctccctatccctcagagatgagcctagtagagaatcagatggggatagagaggagtgttaaagtgctgttcaatggcaaacctgaagtaacctgggtagggaccaccgattgctacaagtatataatcagtaacattcagacctctagtctgggtttcatacactcagatattgagacactcccaaccaaggatgccgttgagaagttagaagaatttgcctctatcctatccctatccctaattttgggaaaaatcggctctattacagttgtcaaaattatgcccattagcggagattttacccaaggcttcatagcctatgccattcaatattttagggagagcctgcttgcctacccgagatatagtaacttcatctcgactgagtgttaccttattatgataggattaaaggccaatcggttgataaacccagaagccattaagcaaagcataatcagagtggggactaggactgcaccaggacttgtgagccacatattatcagagaaacagaaaggttgtattcaatcttttctgggtgatccttatatccaaggagacttcaataagcaccttaaagctctaacccctattgagaaaatcctagtaaattgtggtctctcgatcaatggcacaaaaatctgtagggatctaatccaccatgatatcgcctccggtccagacggtctgatgagctccacaattattttatatagggaactggcccatttcaaagacaatataagaagtcagcacggtatgttccacccctacccagtattggccagtagcaggcaacgtgaattaatccttcgaatagccaagaaattctgggggtatgtcttgctatattctgatgacccggcactaatcaaacaaacgatcaagaacttgaagcggaatcacctaacctttgacttacacagtaatccgtttattaagggcttatccaaagctgagaaactgctagtgcggacaagttcactcagaagggaatggttgttcactctcgatacgaaagaagtgaaagagtggttcaaattggtgggttacagtgcactcgtcagaggttaattcgcgatacatctgcccccttctcctcctccatgagactctactggcaatctaaaagattaaagaaaactacatattggataagtatctattcccagctttgtctggt

>KR781451/Cote_dIvoire/2009

accaaacaaagttgggtaaggatagatcttacaatgactatagactagcaaacttaggagtaaagatcctactgtcggggagaggaggaggagcaagatctttgaccatggcgactctccttaaaagcttagcactgttcaagaggaacaaagacaaggcgccaactgcgtcaggttcaggaggggccatccgggggattaagaatgttatcatagtccctatccccggggactcatccatcattacccgttcaagactgctcgacaggcttgtcagactggccggagaccctgacatcaacgggtcaaagttgaccggcgtgatgatcagcatgttatctttgttcgtggagtcacccgggcaattgatacagcggatcacagatgatccagatgttagcatccgtcttgttgaggtagttcaaagtaccaggtcccagtccggattgacctttgcatcacgtggtgctgatttggacaatgaggcagatatgtatttttcaactgaaggaccctcgagtggaagtaagaaaaggatcaactggtttgagaacagagaaataatagacatagaagtgcaagatgcagaagagttcaatatgttgttagcctccatcttagcacaagtttggatcctcctggccaaggcggttacggcaccagatacagcagccgactcagaactgagaaggtgggttaaatacacacaacaaaggagagtgattggggaatttcgctttgacaaagggtggctggacgcagtccgtaacaggattgcagaagatctatcactccggcggttcatggtatccctgatacttgacatcaagaggacccccggcaacaagccaaggattgcagaaatgatctgcgacattgacaactatattgtcgaggccggactcgcaagtttcattcttactatcaagttcggtattgaaactatgtaccctgcactaggccttcacgagttcgctggggaattgtccactattgaatccttgatgaacttgtatcaacagctaggagaagttgcaccctacatggtaattctagagaactcaattcagaacaagtttagtgcaggagcctatcctctcctctggagctatgcgatgggtgtcggagtcgagctggagaactcaatggggggcttgaactttggcaggtcatattttgacccggcctattttcgtctcggacaggagatggtcagaagatctgcaggaagggtcagctctgtaattgcggctgagcttggcatcacagcagaggaagccaaactggtctcggaaatcgcctcgcagactggggaagaacgaacagttagagggactgggcctcgacaggcgcaggtttccttcctccagcacaaaataggtgagggagagtcgcctacaccagcaaccaaagaagaagtcaaagctgcgatcccaaacggatctgaaggaagggacataaagcgaacacgcccagggaagcccagaggagaaactcccgggcaactgcttctggagatcatgccagaggatggagtctcgcgagagtctggtcaaaaccctcgtgaggctcaaagatcggctgaggcacttttcaggctgcaggccatggccaaaattttggaggaccaggaggagggagaagacaacagtcagatctacaacgacaaggatctcctcagctgagcagatacaccctctgcccaaatcagtgacaagacatcacctgccagtattataaaaaacttaggacccaggtccaaccaaccgcacatcgacaccccagtcaatcgagcggagaccaccgatggcagaagaacaagcataccatgtcaacaaggggctggaatgtatcaagtccctcaaagcctctcccccggatctatccaccatccaagatgcccttgagagctggagagaggggttcaacctctcaggtcgtgcaacaccgaaccatgatacgtccaagggggaccatcagaatatcaaccaatcatgctcatcagcaatcggatcagacaaagtcgacatgtctcctgaaggtaatctcggatttagagagatcacttgtgatgacaatgaggctgggctcggaggagttcaggacaaaagatccgactctcaagtacagcgttactatgtttataaccacgggggtgaagagattgaaggactcgaggatgctgactctctcgtggttcaagcaaatcctccggttgctaacaccttcgatggaggagaggatggatctgacaacagcgatgtggactttggcccagatgatcccgacagagatcctctatatgaccggggacctgctgccggcaatgatgtctctaggtcaacagatgtcgaaaaattagaaggtgatgacattcaagaagttcttaactcccagaagagtaaaggaggaagattccagggcgggaaaatcttgcgagtcccggaaatacccgatgtcaagaactccagaccatcggcccaatcaattaaaaagggcacagacgggagctcagtcttatctggaatggtgacagagtgttcatcgataagtggtgcaacccaagctgtgctagagtccagatgggagtcatcagagcgaaatgcatctgtggggagtgtccccaaatctgtgaggagtgcaaagacgatccaggggttaacacaagaatctggtaccatagcatcactgactcagcctaaagaaaatgactccgagtatgagtatgaggatgatctattcgctgagatgcaggacattcgtgcaagcattgctaagatccatgatgacaacaaaactatcctctcaaaacttgattctctactgttattgaaaggagaaatcgatactatcaagaaacaaatcagcaaacaaaatataagtatatctaccattgagggccatctatccagtataatgatagccatcccaggttttgggaaggacatcaaggacccaacatctgaggttgagctgaacccagatttgagacctataatcagccgcgattctggcagggctcttgcggaggtccttaagaaacccgctgttgataggtctcagaagagcggaatcaaagtcaactccggttcaaagggtcagctcctcaaggatctccagctaaaacccgtcgacaagcaggcaagctctgcaatcgggtttgttccatccgaccatgaatcatccagaagtgtcatccgctccataatcaagtcgagcaagcttaacaatgatcacaaagactatcttctagatttactgaatgatgtgaaaggctctaaggatctcaaggaattccacaagatgctaacagccattcttgccaagcagccgtaacacatcccacaatctacatcccatactcggttgaaagcatcctctcaatcaggctattacaaaaaacttaggagcaagggcaactgagcttcacagacaggatgaccgagatttacgatttcgataaatcagcatgggatgtcaaagggtcaattgctcgcatagaacccaccacctatcacgacggccgactggtaccccaggtgagggtcatcgaccctggtctgggagacagaaaagatgaatgcttcatgtacctgtttcttctaggagtgattgaggataacgaccccctttctcccccagtcgggagaaccttcgggtctttacctctaggtgtcggcaggtcaactgctaagccagaggaactactaagggaggccactgaactagatatagtggtgaggcgcacggcaggactaaatgagaaactggtattttacaacaacactccgctatctttgttaacaccctggaggaaagtcttgacgaccggaagtgtgttcagcgccaaccaggtttgcaatgcggtcaacctagtcccactcgatactccccaaagattcagggttgtgtacatgagtataactagattgtcagataatggttattatagtgtccccagaagaatgttggagttccgctcagccaatgcagtcgctttcaatatcttggttacactgagaattgaaaatggcacaaaccctagaagatacatagtcggctcatgggagaatccagaggtcacatttatggtacacgtgggcaactttagaagaaagaagaacgaagtatactctgctgattattgcaaaatgaagattgaaaagatgggtctagtttttgccttgggaggaataggtggaacaagtctccatattcgaagcacagggaaaatgagcaagaccctccatgcacagctggggttcaagaaaattctatgctaccctctgatggatatcaatgaggatcttaaccgatatttctggcgggcagagcgccgaatagtcaaaatccaggccgtcttacagccatcagtaccccaagaattccgtgtctacgatgatgtcatcatcaacgatgaccaaggcctgttcaagatcctgtaagtcacctgcaacatcatgacacgggtcgcaaccttggtattgctgtttcttttcccaaacactgtcgcgtgccagattcactggggcaatctatccaagatagggattgtcggaacagggagtgccagttacaaggtgatgaccaggccaagccaccaaactctggttataaagttgatgccaaatataacagccatcgacaattgtacgaaatcagagatttcagagtacaaaagattgctaatcacagtgttaaagcctgtagaggatgctttgtcagtaataaccaagaatgtaagaccaattcaagctctaacacctgggcgcaggacccgccgtttcgccggagctgttctggccggagtagcacttggagtcgcgacggctgctcaaataactgccggagtcgcactccatcagtcattgatgaattcccaagcaattgaaagtttaaaaaccagtcttgagaagtcaaatcaggcaatagaagaaatcagacttgcaaataaggagaccatactggcggtacagggcgtccaagattatatcaacaacgagcttgtcccctctgttcatagaatgtcatgtgagctaataggtcacaaactcagtctcaaactccttaggtattataccgagatcctgtctatattcgggcctagccttcgagacccgatagctgctgaaatatcaatccaggcactcagctatgcactaggcggagacatcaataaaattctggacaagcttgggtatagcggcggggatttccttgctattctagaaagcaaggggataaaggcccgggtcacatatgtggacacaagagattactttataattcttagcatagcctacccaaccttatctgagatcaaaggggtgatagttcataagatagaagctatatcatacaacattggggcacaggaatggtatactactatccctaaatatgtagccactcaaggatatttgatatcgaatttcgatgagacatcatgtgtattcactccagaggggacagtctgcagccagaatgcgctgtatccaatgagcccattgcttcaggaatgtttcagggggtcgacaaaatcgtgtgccagaaccctagtttcagggaccacaagtaacagatttatcctatcaaaagggaacttgattgcaaattgtgcgtcagttttgtgcaagtgttacacaacggagacagtcatcaaccaagatcctgataaactactaactgttatagcctcagataagtgtcccgtagttgaggtggatggagtgacaatacaagtcggcagtcgagagtacccagattctgtatacctacataaaatagacttaggcccagccatctctctggaaaaactggatgtaggcaccaatttaggcaatgcagtcacaagactggagaatgcaaaggagctcctagacgcatcagaccagatactgaagactgttaaaggggtacccttcagtggcaatatgtacatagcactggcagcttgcattggggtatccctagggcttgtcacattagtatgctgctgtaaggggagatgtaggaacaaggagattcctgcctccaaaatcaacccagggctcaaacccgacctgaccgggacttcaaagtcttacgtgagatcactgtagtcagaataacccgaatcacccggcatcacgcgtatacatgtgtgacaccagcagtcagaggacgcagaagactcaacctccgatcaccgaccagaccccactctacgccctattacacattggtcatcaaacaaaacttaggacgaaaggtcaatcaccatgtccgcacaaagggagaggatcaatgccttctacaaagacaaccctcacaataaaaaccataggataatcctggatagggaacgcttaactattgaaagaccctacatcttacttggagtcctgctggtaatgttccttagtctaatcgggctgctagccattgcagggatcaggcttcaccgggccaccgttggaactgcggagatccagagtcggctgaataccaacattgagttgaccgaatccattgatcatcaaactaaggatgtcttaacacccctgttcaaaatcattggtgatgaagtcggtatcagaattccacagaagttcagtgatcttgtcaagttcatctccgataagattaagttcctcaaccctgacagagaatacgattttagggatctccggtggtgtatgaacccccctgagagagtcaaaattaactttgatcaattctgtgaatacaaagccacggtcaagtcagttgaacatatatttgagtcatcattcaacaggtcagaaagattgcgactattgactcttggacccggaacaggctgtctcggcaggacagtaacaagagctcagttctcagaacttacgctgaccctgatggacctggatctcgagatgaagcacaacgtgtcctcagtgtttaccgtagttgaagagggattattcggaagaacatatattgtttggagatctgacaccgggaaaccgagcaccagtctagatattggccagtttttaagagttttcgagatcgggttggtgagggatctcgagctgggtgcccccattttccatatgaccaactacctcacagtgaacatgagtgatgactatcggaattgccatttagcggtaggggagttgaagctgacagccctatgcaccccatctgagactgtgactctgagtgagagaggagttccaaagagagagcctcttgtggttgtgatactcaacctagttgggcctactctagggggcgaactatacagtgtcttgcctacctctgacctcatggtggagaaactccatctatcctcacatagagggatcatcaaagacaacgaggccaattgggtagtaccgtctaccgatgttcgtgatctccaaaacaagggagaatgtctggtggaagcatgcaaaacccgacctccttcattttgcaatggcacaggaataggcccatggtcagaggggagaatccctgcctacggagtgatcagggtcagtcttgactttgctagtgacccgggtgtggttatcacttcagtgtttggcccactgatacctcacctatctggcatggatctttacaacaatccgttttcaagagctgcatggctggctgtaccaccctacgagcagtcatttctaggaatgataaatacaattggcttcccggacagagcagaggtcatgccgcacattttgaccacagagatcaaagggcctcgaggtcgttgtcatgttcctatagagttgtcccgcaggattgatgatgatatcaagatcgggtccaatatggttgtattgccgacgagggatctgaggtacataacagccacttatgatgtttccaggagcgagcatgcaattgtgtactatatctatgacacgggtcgatcatcatcttacttctacccagttcgattgaatttcaagggcaatcctctctctctgaggatagagtgtttcccgtggtctcataaggtgtggtgctaccatgattgtcttatatacaacaccatgacaaacgaagaagtccgtacgagagggctgaccggtatagaggtaacatgcaatccagtctgagtcgagctgaaaccatcgctcaagcaggcttccgagccatcccctagttcaagcagcatagtctgggacacgcagcagcacaacccagccaacaatgttataaaaaacttaggagccaaggttgtaggagccatggactcactatcagtcaatcaggttttgtaccctgaggtccatctagatagccctattgtcacaaacaaactagttgctatccttgaatactcggggatcaaccacaactatgttcttgaagaccagactcttatcaaaaatattagatatagactggggtgtggtttctcaaatcaaatgatcatcaataataggggggtaggtgaaacagtcaattccaagcttaaaagttacccccgtaactgtcatatcatatacccagactgcaataaggagttgttttgtatcaaagatagctgcatatctagaaagctctcggagctattcaagaagggtaattccttgtactctaagataagtcaccaggtactggattgtcttaagagagtcaacgggaaattagggctgggcacagatcttaatcacggcctgaaggatggtatcctcgacttggggttgcacatgcatagctctcagtggttcgagacctttctgttctggttcactatcaagacagagatgagatcaatgatcaaagaacagtcccatatatgccacaagaggaggtataacccaatttttgtgtcgggggatgcattcgaggtgcttgtatcacgagacctcgtagtgataattgataagaacacccagtatgtcttctacctgacgtttgagctggtccttatgtattgtgatgtcatagagggtagacttatgacggagacagccatggctatagaccagagatattcagagcttctaaaccgagtcagatacttgtgggaccttatcgatgggttcttcccaacactgggtaacaccacataccaagttgttgctctgcttgaaccactgtcgttggcttatcttcaacttcaggatgtcactctagagttaagaggtgcctttttggaccactgcttcaaagaactttatgagatactggagcattgtggcattgacacggaaggtacctacaattctatcactgagggattggattacgtatttatcactcatgatatacacttaactggggagattttttcatttttccggagtttcggacacccccgcctcgaagcggtcaccgctgcagagaatgtcagaaaacatatgaaccaaccgaaggtaatcagttatgagactatgatgaaagggcatgcagtattttgcgggataatcataaatggttttagggaccggcacggcggcagctggccccctgttgcattgccagaacatgcttctgctgcgatccggaatgcgcaggcatccggcgaaggactgacccacgacctgtgtatagacaactggaagtcctttgtaggattcagatttggctgcttcatgccgctcagcctagatagtgatttgaccatgtacctcaaagacaaagcattggctgcattaaagaatgagtgggattcagtttacccgaaagaatacctccgttataatccacctagagggacagagtcaaggcgactagtagaggtgttcctgaatgactccagctttgatccttataacatgataatgtacgtggtgaatggctcctaccttaaagaccctgagtttaatctctcatacagcttaaaagaaaaggagataaaagagacagggcggttgtttgccaagatgacctacaagatgcgggcctgtcaggtgattgctgaaaatctgatatcaaatggtgttgggaagtatttccgagacaatgggatggcaaaagacgagcatgacctaacaaaagcccttcacactctggcagtctcaggcgttcccaagaacaacaaagacaaccaccgaggtgggccccccagaaggaacgcaagccgagagacgagaccaagccaagccgtcaacacacaaaatagagacaagatccaggggggccctatgtacaactacttgcgatgccaaccaaccagccctgatcagggtgagtcatacgagactgttagtgcattcatcaccgctgaccttaagaagtattgcctaaattggagatacgagacaatcagcatatttgcacagagactgaatgaaatatacgggttgccatccttctttcaatggttacataaggtattggaaaaatccgtgctctacgtcagtgatccgcattgccctcccgacttagatgatcatatccctctggacagtgtccctaatgcccaaatattcatcaagtacccaatgggcggaatagaaggttattgtcaaaaactatggacaatcagtactataccatacttgtatctagcagcctatgagagtggagtaagaatcgcctcactagtgcaaggtgacaatcagacaattgcagtgacaaaaagagttccaagttcttggccttattcactaaaaaagagggaggcatctaaagcagctcaaaattactttgtggtcttaaggcaaaggttgcatgatgtaggtcatcacttaaaggctaatgagaccatagtatcttctcacttttttgtgtactctaaagggatctattatgacggcctgttagtttcacaatcactaaagagcatcgccagatgtgtcttctggtctgagactatcgtggatgaaactagagcagcctgcagcaatattgcaacaactatcgctaagagtatagagaggggttatgataggtacctcgcatactctttgaatatcctcaaaattttccaacagatccttgtatcccttgacttcacgattaatacaacaatgactcaagatgtcgtggcaccgatcatcgagaacggtgatttactgataaggatggcactcttgccagcacccattgggggtctcaattatctcaacatgagcaggttgtttgtgagaaatatcggggacccggtcacttcctccatagccgacctgaagaggatgatagacgctgggctaatgccagaagaaacattgcatcaagtgatgacccagaccccgggagaatcatcctaccttgattgggcaagtgatccttattctgccaacctaccctgcgtacagagtataactcgccttctcaagaacatcactgcacggtatattttaatcagcagcccaaatccgatgctgaaagggttgtttcacgaggggagtagagatgaagacgaggagcttgcgagtttcttaatggatcggcatataattgttccaagagctgcacatgaaatcttagaccacagcataaccggggcaagggaagctatagccgggatgttggacaccaccaagggtctgattagaacaagtatgaaacggggtggcctcacccctcgagtattagcccgcctttccaattatgattatgaacaattcagatccgggataacactattgacaaagaaagggcagtgttatctcattgacaaggactcgtgctcggtgcagctcgctatagctctgaggagccatatgtgggcaaggttggctcgcgggagacctatctatggcttggaggtgcctgatgtactggaatcgatgaacggctaccttatcaaacgtcacgagtcttgtgccatctgtgaaacgggctcaagtcactacgggtggtttttcgtccctgcagggtgtcagcttgacgatgtctcaagagagacttcagctcttcgtgtgccttatgtcggatcaaccactgaggaaaggacagatatgaaacttgctttcgttagatctccaagccgatccctcaaatcagcagtcaggattgccacagtctactcatgggcctacggggatgatgagaaatcatggagtgaagcttggatgctagctaggcagagagccgatgtcaccttagatgaactgagaatgatcactccagtctctacatccaccaacctagctcatcggttaagggatcggagcactcaggttaaatattcggggacatcccttgtgagggttgcaaggtacacaaccatctccaatgacaatttgtcatttgtgatatctgagaaaaaagtagataccaacttcatttaccagcaagggatgctgctcggtcttgggatccttgagaatctcttcaggttagaggccaccacaggggtatccaacacagtgctacacctgcacgtggaaacagaatgttgtgttgtacctatggttgaccacccaaggataccgagtctccgcaatatcaaagttacgaacgagctatgcacaaaccctcttatctacgacaagtcccccatcatagaacacgatgcaactcgattatactcacaaagccacaggagacatttggtggagtttgttacctggtcaacaagccagctctatcatatactggccaaatctacagcaatgtccatgattgagctgatcacaagatttgagaaagatcacatgaatgaaatagccgctctgattggcgatgacgacatcaacagtttcatcacagaatttttgcttgtggagcccagactgtttatagtttacctcggccagtgtgctgccatcaattgggcctttgatatacattatcatcggccctcgggcaagtaccagatgggggaactcctctactctttactctctcggatgagcaagggagtatataagatcttcactaatgctctgagccaccccaaagtttacaagaaattctggcgaagtggtataattgagccggttcatggcccatccctagatacacagaatttacatgtcactgtctgtgacatgatattcggatcatacgtcacctatctggatcttttgctgaatgatgagctggatgcttacccgtatttgctctgcgagagtgatgaggacgtggtcacagacaggttcgacaacattcaagccaaacatctctgtgtactggccgatgtatactgcagctccaagaggtgtccctcgataatcgggatgtcccctatagaaaaatgtaccatcctcacacattacatcaagggagaatcggtacaatccccgtccgggatctcatggaacaccgatccccttgtagtagatcattactcatgctctctgacctacctccgccgtggttccatcaaacaaatcagattgagagtggatcctgggtttgtgttcgaggcgttgacagacatcgacttcaaacagcctcgcaaggctaagttggacgtatcgattgtgggattgactgatttttctcccccttgggataacgtcggtgattttctagggactatcaacacattgaggcacaatctgcccgtcaccgggaccggggtctcgaactatgaagtccacgcttatcgtagaattggtctgaattcatcagcatgttataaagctgtagagatctccacgttaataaagtcatccttagaagctggggagaatggattgttcttaggagaaggctccggctcgatgctggctgcgtacaaggaagttcttaaattggctaactgttattacaacagtggggtaacagcagagggtagagccggacagagggaaatctctccctatccctcagaggtgagcctggtagagagtcagatggggatagagagaagtgttaaagtcctgttcaatggcaaacctgaagtaacctgggtagggaccaccgattgctacaagtatataatcagtaacattcagacctctagtctgggtttcatacactcagatatcgagacactcccaaccaaggatgccgttgagaagttggaggaatttgcctctattctatctctatccctaattttgggaaaaatcggctctattacagttgtcaaaattatgcccattagcggagattttacccaaggcttcatagcctatgccattcaatatttcagggagagcctgcttgcctatccgagatatagtaacttcatctcgactgagtgttaccttattatgataggattaaaggccaatcggttgataaacccagaagccattaagcaaagcataatcaggatggggactaggactgcaccagggcttgtgagccacatattatcagagaaacagaaaggttgtattcaatcttttctgggtgatccttatgtccaaggagacttcaataagcaccttaaagctctaactcctattgagaaaatccttgtaaattgtggtctctcgatcaatggtacaaaaatctgtagggatctaatccaccatgatattgcctccggtccagacggtctgatgagctccacaattattttatacagggaactggcccatttcaaagacaatataagaagtcagcacggcatgttccacccctacccagtattggccagtagcaggcaacgtgaattgatccttcgaatagccaagaagttttgggggtatgtcttgctatattctgatgacccggcactaatcaaacaaacgatcaagaacttgaagcggaatcacctaacctttgacttacacagtaatccgtttattaagggcttatccaaagctgagaaactgctagtgcggacaagttcactcagaagggaatggttgttcactctcgatacgaaagaagtgaaagagtggttcaaattggtgggttacagtgcactcgtcagaggttaattcgcgatacatctgcccccttcccctccaccatgagactctactggcaatccaaaagattaaagaaaactacatattggataagtatctattcccagctttgtctggt

>KR828813/Nigeria/Yobe/2013/N14

accaaacaaagttgggtaaggatagatcttataataactatgaactgacaaacttaggagtaaagatcctattgtcggggagaggaggaggagcaagatcttcgattatggcaactctccttaaaagcttagcattgttcaaaaggaacaaagacaaagcgccgacggcatcaggttcaggaggggccatccgggggattaagaatgttatcatagtcccgattcccggagactcgtccatcattacccgttcaagactgctcgacaggcttgtcagattggccggagatcctgacatcaacgggtcaaagctgaccggcgtgatgatcagcatgttatctttgttcgtagagtcacccgggcaattgatacagcgaatcacagatgatccagatgttagtatccgccttgttgaggtagttcaaagtactaggtctcagtccgggctgacctttgcatcacgcggtgctgatttggacagcgaggcagacatgtatttttcaactgaggggtcctcgagtggaggcaagaaaaggatcaactggtttgagaacagagaaataatagacatagaggtgcaggatccagaagagttcaatatgttgttagcttccatactagcacaagtctggatcctcctggccaaggcggttacggcaccagatacggcagccgactcagagctgagaaggtgggttaaatacacacagcaaaggagagtgattggggaatttcgctttgacaaaggatggctggacgcagttcgtaacaggattgcagaagatctatcacttcggcggttcatggtatctctgatacttgacatcaagaggacccccggcaacaagccaaggattgcagaaatgatctgcgacattgacaactatattgtcgaggccggactcgccagtttcattcttactatcaagtttggtatcgaaaccatgtatcctgcactaggccttcacgagtttgcaggggaattgtccactattgaatccttgatgaacttgtatcaacagctaggagaagttgcaccctacatggtaattctagagaactcaattcagaacaagtttagtgcaggagcttatcctctcctctggagctatgcgatgggtgtcggagttgagctggagaactcaatggggggcttgaactttggcaggtcatattttgacccggcctattttcgtctcggacaggagatggtcagaagatctgcaggaaaggtcagctctgtaattgcggctgagcttggcatcacagcagaggaagctaaactggtctcggaaatcgcctcgcagactggggacgaaagaaccgctagggggactgggcctcgacaagcgcaggtctccttcctccagcacagaccgggagagggagagtcgcccacaccagcgaccagagaaggggtcaaagctgcgatctcaaacggatctgaagagagggaaagaaagcaaacacgctcaggaaggcctagaggggagacccctggccaactgctcctggaaatcatgccagaggatgaagtctcgcgagagtctggtcaaaaccctcgggaggctcaaagatcggccgaggcactcttcaggctgcaggccatggccaagattctagaggaccaggaggagggagaagacaacagtcagatccacaacgacaaagatctcctcagctgagcagacgcaccctctgtcgaaatcagtgaagagacatcgcccaccagtattataaaaaacttaggacccaggtccaagcgatcacacatcgacactccagtcagtcgagcggagaccaccgatggcagaagaacaagcataccatgtcaacaaggggctggaatgcatcaagtctctcaaagcctctcccccggatctatccaccatcaggtacaccattgagagctggagagaggggcttagcccctcgggtcgtgcaacaccgagccccgatacgtccgagggagatcatcagaatatcagccaatcatgcttgccagcaatcggatcaaacaaagtctacttgtctcctgaagataatctcggatttagagagatcactggcaacgactgtgaggttgggctcggaggagtccaggggaaaggatccaactctcaagtacagcgttactatgtttatagtcacgggggtgaagagattgagggactcgaggatgctgactctctcgtggttcaagcagatcctccagttgctgacatcctcaatggaggagaggatggatctgacgacagcgatgtggaatctggcccagatgatcccggcagagatactctatatgaccggggatctgttgccggcaatggtgtcgctagatccacagatgtcgaaaaactagaaggtgctgatattcaagaagttcttaactcccagaaaagcaaaggaggaagattccaaggcgggaaaaccttgagggtcccggaagtacccgatgtcaagcactccagaccatcagcccaatcaattaaaaagggcacagacgggaactcagtctcatctggaacggggacagagtgtttatcgataagtggtgcaacccaagctgtgccagagtcaagatgggagttatcagagcaaaatgcgtctgtggggagtgtcctcaagtctgcgaggagtgcaaagacgatccaggagttgacacaagaatctggtaccatagcatcactgactcagcctaaagagaatgactccgagtatgagtatgaggatgatctatttacagagattcaggacatccgtgcaagcattgccaagatccatgatgacaacaaaactatcctctcaaagcttgattctatactgttattgaaaggagagatcgacactatcaagaaacaaatcagcaagcagaatataagtatatccaccattgagggccatctctccagcataatgatagccatcccgggctttgggaaggacatcaaggacccaacatccgaggtagagctgaacccagatttaagacctataataagccgtgactctggcagagctctcgcggaggtcctcaagaaacccgctgtggatagatctcagaaaaatggaatcaaagtcaactccagctcaaagggtcagcttcttaaggatctccagctaaaacctgtcgacaagcaggtaagctctgcaatcggatttgtcccatccgaccatgaatcatccagaaatgtcatccgctccataatcaagtcgagcaagctaaacatcgatcacaaggactatcttctagatttactaaatgatgtgaaaggctccaaggatcttaaggaattccacaagatgctaacagctattctcgccaagcacccgtaacgtaccctccaatcaccatctcacactcggttgaaaacatcctctcaatcaggctattacaaaaaacttaggagcaagggcaactgagcttcgcagacaagatgactgagatctacgacttcgataaatcagcatgggatgtcaaaggatcaatcgctcgcatagaacccaccacctatcacgacggccgactgatacctcaggtaagggtcattgatcctggtctgggagacagaaaagatgagtgcttcatgtatctgtttctcctaggagtgattgaggataacgaccccctgtctcccccagtcggaagaacctttggttctttacctctaggggttggtaggtcaactgctaagccggaagaactactaagggaggccacagaactagatatagtggtgaggcgcactgcaggactaaatgagaaactggtattttacaacaacactccgctgtccttgttaacgccctggaggaaagtcctgacaaccggaagtgtgtttagcgctaaccaggtttgcaatgcagtcaacctagtcccacttgatactccccagagattcagggttgtgtacatgagcataactagattatcagacaatggttactatagtgtccccagaaaaatgctggagttccgctcagccaatgcagtagccttcaacatcttggttacactaagaattgaaaatggcacaaaccccagaagatacatagtcggctcatgggagaatccagaagtcacatttatggtacacgtgggcaatttcagaagaaagaagaacgaagtatactctgcggattactgcaaaatgaagattgaaaagatgggtttagtttttgccctgggaggaataggcggaacaagtctccatattagaagcaccgggaaaatgagcaaaacccttcatgcacagctggggttcaagaaaatcttatgttaccccctaatggatgttaacgaggatcttaaccgatatctctggcgggcagagtgccgaatagtcaaaatccaagctgtcttacagccatcagtaccccaagaattccgtgtctacgatgatgtcatcatcaacgatgatcaaggcttgttcaagatcttgtaattcatttgcaacatcatgacacgggtcgcaatcttgacatttctgattcttttcccgaatgctgttatgtgccagattcactggggcaatctatccaagatcgggattgtaggaacagggagtgccagctataaggtgatgactaggccaagccaccaaactctggttataaagttaatgccaaatataacagccatagacaattgtacaacatcggagattgcagagtacaagagattgctgatcacagttttaaagcctatagaggatgctctgtcagtgataaccaagaacgtaagaccaattcaaactctgacatctgggcgcagaactcgccgttttgctggagctgttctggccggagtagcacttggggtagcgacagctgctcagataactgccggagtcgcccttcatcaatcattgatgaattcccaagcaatcgagagtttaaaaaccagtcttgagaagtcgaatcaggcaatagaagaaatcagacttgccaataaggagaccatactggcggtacagggtgtccaggattatatcaacaatgagcttgtcccttctgtccatagaatgtcatgcgagctggtaggtcacaagctcggcctcaagctccttaggtactacaccgagatcctgtccatcttcgggcccagccttcgagacccgatagctgccgaaatatcaatccaggcactcagttatgcattaggcggagacattaataaaatcctggaaaagcttgggtatagcggtggggatttccttgctattctagaaagcaagggaataaaggcccgggtcacatatgtggacacaagagattactttataattcttagcatcgcctacccaaccttatctgagatcaagggagtgatagttcacaagatagaagctataacatacaacatcggggcacaggagtggtatactactatccctaaatatgtagccactcaggggtatctgatatcaaactttgatgagacgtcatgtgtattcactccggaagggacagtttgcagccaaaatgcgttgtacccaatgagcccattgcttcaggaatgtttcagggggtcgacaaaatcatgcgccagaaccctcgtttcagggaccataagtaatagattcatcctatcaaaagggaacctgattgcaaattgtgcgtcagtcttgtgcaaatgttacacaacggagacagttatcagccaagatcctgataaactactaactgttgtagcatccgataagtgtcctgtagttgaggtggatggagtgacaatacaggtcggcagtcgagagtatccggactctgtatacttacacaaaatagacttaggtccagccatctccctagaaaaactggatgtaggcaccaatttaggcaatgcagttacaagactggagaatgcaaaggagctcctagatgcatcagaccaaatactgaagactattaaaggggtacctttcagtgggaatgtgtacatagcactggcagcttgcattggggtatccctaggcctcgtcacattaatatgctgctgtaaggggaggtgtaagaataaagaaatccctatctccaaaatcaaaccagggctcaaacccgacctgaccgggacctcaaagtcgtacgtgagatcactgtagtcagaatcaactgaaccatccggcatcacacacatacatgtgcgacacaagcagtcggaggacgcaggaaacccagcttatgatcacccacctgaccccattccacgctccactacacattagtcatcaaacaaaacttaggacgaaaggtcaatcaccatgtccgcacaaagggaaaggatcaatgccttctacaaagacaatcctcataataagaaccatagggtgatcctggatagagaacgcttgatcattgaaagaccctacatcttgctaggagtcctgctagtaatgttcctgagtctaatcggactgctggccattgcagggatcaggcttcaccgggccaccgttggaacttcagagatccagagtcggctgaataccaatattgagttgaccgaatctattgatcaccaaactaaggatgtcttaactcccctgtttaaaatcattggtgatgaagtcggcatcagaattccacagaaattcagtgatcttgtcaagttcatctccgataagattaagttcctcaaccctgatagagagtatgatttcagggatctccggtggtgtatgaacccccccgagagagtcaaaattaattttgatcagttttgtgagtacaaagccgcggttaagtcaattgaacatatatttgagtcaccactcagcaagtcaaaagagttgcgatcattgactcttgggcccagaacaggttgtctaggcagaacagtaacaagagctcacttctcagaacttacactgaccctaatggacctggacttagagatgaagcacaacgtgtcctcagtgtttaccgtagttgaagaggggttattcggaagaacatataccgtctggagatcagatgccagggatttgagcaccgatatagatactggccattttttaagggtcttcgagatcggactggtaagagatcttgggctgggtcctcctgttttccatatgaccaactatctcacagtgaacatgagtgatgactatcggagatgtcttttagcggtaggggggttgaagctgacagccctatgcaccccgtccgagactgtgacactgagtgagagaggggttccaaagagggagcctcttgtggttgtgatacttaacctcgctgggcccactctagggggtgaactatacagtgtcttgcctacctctgacctcatggtggagaaactctatttatcttcacatagagggattatcaaagatgatgaggccagttgggtagtgccgtctaccgatgttcgtgatcttcaaaacaaaggagaatgtctggtggaagcgtgcaagactcgacctccctcattttgcaatggcacaggatctggcccgtggtcagaggggagaatccctgcttacggggtgatcagagtcagtcttgacttggctagggacccaggtgtagttatcaattcagtgtttggcccattgatacctcacctatccggcatggatctttacaacaatccgttctcaagagctgtatggttggctgtaccaccttatgagcagtcatttctaggaatgataaatacaattggattcccgaacagagcagaggttatgccgcacattttgaccacagagatcagaggccctcggggtcgttgccacgttcccatagaattgtcccgcagggttgatgacgatatcaagatcgggtccaacatggtcatattaccgacgaaggacctgaggtacattacagccacttatgatgtttccaggagcgagcatgcgatcgtgtactatatctatgacacaggccgttcatcatcttacttctacccggttcgactgaatttcaaaggcaatcctctctctctgaggatagagtgtttcccttggcatcataaggtgtggtgctatcatgattgtctgatatacaacaccacatcaggtgaagaggtccatacgagagggctaaccggtatagaggtaacatgtaatccagcctgagcagagctacaaccatcactcaggcagacccttgagtcgtcaccgagtccaagcagcacagcctgggacactcaacagcacagctcagccaacaatgttataaaaaacttaggagccaaggttttagaggccatggactccctatcagtcaatcaggtcttgtaccctgaggttcatctagatagccctattgtcacaaacaaactagttgccatccttgagtactcggggatcgaccataactatgttcttgaggaccagactcttatcaaaaatattaggtatagactgggatgcggcttttcaaatcaaatgatcatcaataataggggggtgggtgaaacagtcaactctaaacttaaaagttatcctcataatcgtcatatcatatacccagattgcaataaggagttattttgtatcaaagatagctgcatatctaagaagctctcggagctattcaagaagggtaattccttgtactctaagataagtcaccgggtactggattgtcttaagagagtcaacgggaaattagggctgggcacagatcttactcatggtctgaaggagggtatccttgacctagggttgcacatgcatagctctcaatggttcgagacttttcttttctggttcactatcaagacagagatgagatcaatgatcaaagaacagtcccacatatgccacaagaggaggtataacccaatttttgtgtcaggggatgcattcgaggtgctcgtatcacgagacctcgttgtgataattgataaaaatacccagtatgtcttctacttaacatttgagctggtccttatgtactgtgatgtcatagagggcagacttatgacggagacagccatggccatagaccagagatattcagaacttctaagccgggtcagatacttgtgggatcttattgatgggttcttcccaacactaggcaacaccacataccaagttgttgctctgcttgaaccattatcgttagcttatcttcaacttcaggatgtcactctggagttaagaggtgcttttttagaccattgtttcaaagaactctatgaaatactggagcattgcggcattgacacagaaggcacctacaattccatcactgaaggattggattacgtattcatcacccatgatatacatttaactggggagattttttcatttttccggagtttcggacacccccgtctggaagcggtcaccgctgcagaaaatgtcagaaaacatatgaaccaaccgaaggtaatcagttatgagaccatgatgaaagggcatgcagtattttgcgggataatcataaatggttttagggaccgacatggtggcagctggccccctgttgcattgccagaacatgcttctgctgcgatccgaaatgcgcaggcatcaggtgagggactgacccatgacctgtgtatagacaattggaagtcttttgttggattcagatttggctgcttcatgccactcagcctagatagtgatttgaccatgtacctcaaagacaaggccttggctgcactgaagaatgagtgggattcggtttacccgaaagagtacctccgttacaacccacctagagggacagagtcaaggcgactggtggaggtgttcctgaatgactccagctttgatccttataacatgataatgtacgtggtgaatggctcctaccttaaagaccctgagtttaatctctcatacagcctaaaagagaaagagataaaagagacagggcggttgtttgccaaaatgacctataagatgcgggcctgtcaggtaatcgccgaaaatctgatatcgaatggtgttgggaaatatttccgagacaatgggatggcaaaagacgagcatgacctaacaaaagcccttcacaccctggcagtctcaggtgttcccaagaataacaaagacaaccaccgaggtgggcctcccagaaggacaaccagccgagagttgagatcgagccaatacaccaaaaaacagaatagagacaaggtccaaggagggcctatgtacaactatttgcgatgccaaccaatcagccctgatcagggtgagtcatacgagactgtcagtgcattcatcaccgctgaccttaagaagtattgcctgaattggaggtacgagacaatcagcatatttgcacagagactgaatgagatatatggactaccatccttctttcaatggttacacagaatattggaaaaatccgtactttatgtcagtgacccacattgccctcccgacctagataatcatatccctctggacagtgtccctaatgcccaaatattcattaaatacccaatgggcggaatagaaggttattgtcaaaaactatggacaatcagcactataccatacttgtacctggcagcctatgagagcggagtaagaatcgcctcactagtgcagggtgacaatcagacaatcgcagtgacaaaaagagttccaagttcttggccttattcactaaaaaagagagaggcatccaaagctgctcaaaattacttcgtagtcttgaggcaaagattgcacgatgtaggtcatcacttaaaggctaatgaaaccatagtatcttctcacttttttgtatattctaaagggatttattatgatggcctgctagtctcacaatcactaaagagcatcgccaggtgtgtcttctggtccgagactatagtggatgaaaccagagcggcctgcagcaatattgcaacaactatcgctaagagtatagagaggggttatgataggtaccttgcatattctttgaatatcctaaagatcttccagcagatccttatatcccttaacttcactattaagacaacaatgactcaagatgtcgtggcaccgatcatcgagaacggtgatttgctgacaaggatggcactcttgccagcacccatcgggggtctcaattatcttaacatgagcagggtatttgtgagaaatatcggtgacccggtcacctcatctatagccgacctgaagaggatgatagacgctgggctaatgccagaagaaacattgcaccaagtgatgacccagaccccgggagaatcgtcctatctcgattgggcaagtgacccttattctgccaacctaccctgcgtacagagtataactcgccttctcaaaaccatcactgcacgatatattttaatcagcagcccaaaaccgatgctgagaggactgtttcatgagggaggtaaaaatgaagatgaggagcttgcaagtttcttgatggatcggcatataattgttccaagagctgcacatgaaattctagaccatagcataaccgaggcaagaaaggctatagccgggatgttggataccaccaaaggtctgataagaacaagtatgaaacggggtggcctcacccctcgagtcttagcccgcctttccaattatgattatgaacaattccgatccgggataatactattgacaaagaaagggcagtgttatctcattgacaaggactcgtgctcggtgcagctagctatagctctgaggagccacatgtgggctaggttggctcgcgggagaactatctatggattggaggtgcccgatatattggaatcgatgaacggctacctcatcaaacgccacgagtcttgtgctatctgtgaaacgggctcaaatcactacggatggtttttcgtccctgcagggtgccagcttgacgatgtatctagagagacctcggctcttcgtgtaccttatgtcgggtcaaccactgaggaaaggacagatatgaaacttgcttttgttaggtctccaagtcgatcccttaaatcagcagtcaggatcgcaacagtttactcatgggcctacggggatgatgagaaatcatggaatgaagcctggatgctagctaggcagagagccaatatcaccttagatgaattaagaatgatcactccagtctctacatccaccaacctagcccaccggttgagggatcggagcacccaggtgaaatactcggggacatctcttgtgagggttgcaagatacacaaccatctccaatgataacttgtcatttgtgatatctgagaaaaaagtggataccaacttcatttaccagcaagggatgctgcttggtcttgggatccttgagaacctcttcaggctagaggccaccaccggagtatccaacacagtgttacacctgcatgtggaaacagaatgttgtgttgtacctatggttgatcacccaaggataccgagtctccgtaatataaaagttacgaatgagctatgcacaaaccctttgatctacgacaggtctcccatcatagaacacgatgcaacccgactatactcacagagccacaggagacacttggtggagtttgttacctggtcaacaagccagctttaccatatactagccaagtctacagcaatgtccatgattgagttgatcacgagattcgagaaagatcacatgaatgaaatagccgccctgattggcgatgacgacatcaacagcttcatcacagaattcttgctagtagagcctagattatttatagtttaccttggtcagtgtgctgccatcaattgggcttttgatatacattatcatcggccctcgggcaagtaccagatgggggaactcctctactctctactctctcggatgagcaaaggggtatataagatcttcactaatgctttgagccaccccaaagtttacaagaaattttggcgaagtggtgtcatcgagccgattcatggaccgtccctggatacacagaatttacatgtcactgtctgtgacatgatatatgggtcctacgtcacctatctagatcttttgctgaatgatgagctcgatgattatccgtatttgctctgcgagagtgatgaggacgtggtcacagacaggttcgacaacattcaagccaaacatctctgtgtattggccgatgtatattgcagctctaagaggtgtccttcgataatcgggatgtctcccatagaaaaatgtaccattctcacacattacatcaagggagaatcaatacaaaccccgtctgggacctcatggaacactgatcctcttgtagtagatcattactcatgctctctgacctaccttcgccgcggttctatcaaacaaatcaggttgagagtggatcctgggtttgtattcgaggcattgacagacgtcgactttaaacagcctcgcaaagccaagttggatatatcggttgcggggttgactgatttctctcccccttgggacaacatcggtgattttctagggactatcaatacactgaggcataatctgcccgtcaccggaaccggggtctcgaactatgaagtccacgcttatcgtagaattggcctgaattcatcagcatgttataaagccgtagagatctccacgttaatcaagccatccttagaagtcggagagcatggattgttcttaggagaaggttccggttcaatgctggctgcgtacaaggaagttcttaaattggcaaactgttattacaacagcggggtaacagcggagggcagagccggacagagggaaatatctccctatccttcagagatgagcctagtagagaatcaaatggggatagagaggagtgttaaagtactattcaacggcaaacctgaagtaacttgggtggggaccaccgattgctacaagtacataatcagtaacatccaaacctctagtctaggtttcatacactcagatattgagacactcccaaccaaggatgctgttgagaaattagaagaatttgcctctatcctatccttatccctcattttgggaaaaatcggctctatcacagttgtcaaaattatgcccattagcggggattttacccaaggcttcatagcctatgcaatccaatattttagggagagcctgcttgcctatccgagatatagtaacttcatctcgactgagtgttaccttattttgataggattaaaggccaatcgattgataaacccggaagccattaagcaaagcataatcagagcgggggccaggactgcaccaggacttgtaagccatatattatcagagaaacagaaaggttgtatccaatctttcctgggtgatccttatatccaaggagacttcaacaagcaccttaaatctctaacccctattgagaaaatcctggtaaattgtggtctctcgatcaatggcacaaaaatctgtagagatctaatccaccatgatatcgcctccggtccagacggtcttatgagctctacaattattttatatagggaattggctcatttcaaagacaatataaggagtcagcacggaatgtttcacccctacccagtactggccagtagccggcaacgtgaattaatccttcgaatagccaagaaattctggggttatgtcttgctatattctgatgacccggcactaatcaggcaaacaatcaagaacttgaagcggaatcatttaacctttgacttacacagtaacccgtttattaaagggctatccaaagctgagaaactgctggtgcgtacgagttcactcagaagggaatggctgtttactctcgatacgaaagaagtgaaagagtggttcaaattagtgggttacagtgcactcatcagaggctagttagctatacgtctgcccccttctcctctgccatgagaccctactgacggtctaaaagattaaagaaaactacatattggataagtatctattcccagctttgtctggt

>KT270355/India/Gingee/2014/01

accaaacaaagttgggtaaggatagatcttataataactatggactagcaaacttaggagtaaagatcctactgtcggggggaggaggaggagcaagacctctgattatggcgactctccttaaaagcttagcattgttcaaaaggaacaaagacaaagcgccgacggcatcaggttcaggaggggccatccgagggattaagaatgttatcatagtcccgattcccggagactcgtccatcactacccgttcaagactgctcgacaggcttgtcagattggccggagatcctgacatcaacgggtcaaagctgaccggcgtgatgatcagcatgttatctttgttcgtagagtcacccgggcagttgatacagcgaatcacagatgatccggatgttagtatccgccttgttgaggtagttcaaagtactaggtcccagtccgggttgacctttgcatcacgtggtgctgatttggataacgaggcagacatgtatttttcaactgaggggccctcgagtggaggtaagaaaaggatcaactggtttgagaacagagaaataatagacatagaggtgcaggatccagaagagttcaatatgttgttagcctccatactagcacaagtctggatccttctggccaaggctgtcacggcaccggatacggcagctgactcagaactgagaaggtgggttaaatacacacaacaaaggagagtgattggggaatttcgccttgacaaagggtggctggacgcagtccgcaacagaattgcagaagatctatcactccggcggttcatggtatctctcatacttgacatcaagaggacccccggcaacaagccaaggatcgcagaaatgatctgcgacattgacaactatattgtcgaagcaggactcgccagtttcatccttactatcaaatttggtatagaaaccatgtatcctgcattagggctccacgagtttgccggggaattgtccactattgagtccttgatgaacttgtatcaacagctaggagaggttgcaccctacatggtaattctagagaactcaattcagaacaagtttagtgcaggagcttatcccctcctctggagctatgcgatgggtgtcggagttgagctggagaactcaatggggggcttgaactttggcagatcatattttgaccctgcctattttcgtctcggacaggagatggtcagaagatctgcaggaaaggtcagctctgtgattgcggctgagctcggcatcacagcagaggaagctaaactagtctcggaaatagcctcgcaggctggggacgaaagaaccgctagaggaactgggcctcgacaggcgcaggtctccttcctacagcaccaaacaggagggggagagtcgtccgcaccagcgaccagagaaggggtcaaagctgcgatcccaaacggatccggagaaagggacagaaagcaaacacgcccagggaggcccagaggagagacccccggccaactgctcctggaattcatgccagatgatgaggtctcgcgagaatctggtcaaaaccctcgtgaggctcaaaggtcggccgaggcactcttcaggctgcaggccatggccaagattctggaggaccaggaggagggagaagacgacaatcaggtctacaacgacaaggatctcctcggctaagcagacgcaccctctgtcgaaatcagtgacgagacatctcctaccagtattataaaaaacttaggacccaggtccaagcaagcacacatcgacactccaaccagtcgagcggaggccaccgatggcagaagaacaggcataccatgtcaacaaggggctggaatgtatcaagtccctcaaagcctctcccccggatctatccaccattagagataccatcgcgagctggagagaggggcttagcccctcgggccgtgcaacaccgaaccctgatacgtccgagggagatcatcagaatatcaaccaatcatgctcaccagcaatcggaccaaacaaagtctacttgtctcctgaagataatctcggatttagagagatcactggcaacgactgtggggctgagctcggaggagtccagggaaaaggatccaactctcaagtacagcgttactatgtttatagccacgggggtgaagagattgaaggactcgaggatgctgactctctcgtggttcaagcagatcctccagttgctaacatattcaatggaggagaggatggatctgacgacagcgatgtggactctggcccagatgatcccagcagagatactctatatgaccggggacctgctgccggcaatgatgtcgctaggtccacagatgtcgaaaaactagaaggtgctgatattcaagaagttctcaactcccagaaaagcagaggaggaagattccaaggcgggaaaaccttacgagtcccggaagtacccgatgtcaagaactccggaccatcagcccaatcaattaaaaagggcacagacgggaactcagtctcatctggaacggtgacagagtgtttatcgataagtggtgcaacccaagctgtgccagagtcaagatgggagtcatcagagcaaaatgcgtctgtggggagtgtcctcaagtctgcgaagagtgcaaagacgatccaggggtcgacacaagaatctggtaccacagcatcaccgactcagcctaaagagaatgactccgagtatgagtatgaggatgacctatttacagagattcaggacatccgtgcaagcattgccaagatccatgatgacaacaagactatcctctcaaagcttgattctatactgttattgaaaggagaagtcgacactatcaagaaacaaatcagcaagcagaatataagtatatccaccattgagggccatctctccagtataatgatagccatcccgggctttgggaaggatatcaaggacccaacatccgaggttgagttgaacccagatttaagacctataataagccgtgactctggcagagctctcgcggaggtcctcaagaaacccgctgtggataggtctcagaaaactgaaaccaaagccaactccagctcaaagggtcagcttcttaaggatctccagctaaaacctgtcgacaagcaggcaagctctgcaatcgggtttgtcccatccgaccatgaatcatccagaaatgtcatccgctccataatcaagtcgagcaagctaaacattgatcacaaggactatcttctagatttactgaatgatgtgaaaggctcaaaggatcttaaggaattccacaagatgctaacagcaattctcgccaagcacccgtaacacatcccccagtcaccatctcatactcggctgaagacatcctttcaatcagactattacaaaaaacttaggagcaagggcaactgagcttcgcagacaagatgaccgagatctacgacttcgacaaatcagcatgggatgtcaaagggtcaattgcccgcatagaacccaccacctaccacgacggccgactgataccccaggtaagggtcatcgatcctggtctgggagacagaaaagatgagtgctttatgtacctgtttctcctgggagtgattgaggataacgaccccctgtctccccccgtcggaagaacctttggctctttacctctaggggtcggtaggtcaactgctaagccagaagaactactaagggaggccacagaactagacatagtggtgaggcgcactgcaggagtaaatgagaaactggtattttacaacaacactccgctgtccttgttaacgccctggaagaaagttctgacaaccggaagtgtgtttagcgctaaccaggtttgcaatgcagtcaacctagtcccgcttgatactccccagaggttcagggttgtgtacatgagcataactagattatcagacaatggttactatagtgtgcccaggagaatgctagagttccgctcagccaatgcagtagccttcaacatcttggttacactaagaattgagaatggcacaaaccctagaagatacatagtcggctcatgggagaattcagaggtcacatttatggtacacgtgggcaactttagaagaaagaagaacgaagtatactctgctgattactgcaaaatgaagattgaaaagatgggtttagtttttgccctgggaggaataggtggaacaagtctccatattagaagcaccgggaaaatgagcaaaaccctccatgcacagctggggttcaagaaaatcttatgttaccccctaatggatgttaatgaggatcttaaccgatatctctggcgggcagagtgccgaatagtcaaaatccaggccgtcttacagccatcagtgccccaagaattccgtgtctacgatgatgtcatcatcaacgatgaccaaggcttgttcaagatcctgtagttcatctgcaacattatgacacgggttgcaatcttgacatctctgtttcttctcccaaatgttgttgcgtgccagattcactggggcaatctatccaagatcgggattgtgggaacagggagtgccagctacaaggtgatgactaggccgagccaccagactctggttataaagttaatgccaaatataacagccatcgacaattgtacaaagtcagagattgcagagtacaagagattgctgatcacagtgttaaagcctgtagaggatgctctgtcggttataaccaagaatgtaagaccaattcaaaccctgacacctgggcgtagaacccgtcgttttgctggagctgttctggccggagtagcgcttggagttgcgacagccgctcagataactgcaggagtcgctcttcatcaatcattgatgaactcccaagcaattgagagtttaaaaaccagtcttgagaagtcgaatcaggcaatagaagaaatcagacttgcaaataaggagaccatactggcagtacagggcgtccaggattatatcaacaatgagcttgtcccttctgttcatagaatgtcatgcgagctggtaggccacaagctcggcctcaagctccttaggtactacaccgagatcctgtccatattcgggcccagccttcgagacccgatagctgccgaaatatcaatccaggcactcagttatgcattaggcggagacattaataaaatcctggacaagcttgggtatagcggtggggatttccttgccatcctagaaagcaagggaataaaggcccgggttacatatgtggacacaagagattactttataatgcttagcatcgcctacccaaccttatctgagatcaagggagtgatagttcacaagatagaagctataacatacaacattggggcacaggagtggtatactactatccctaaatatgtagccactcagggatatctgatatcgaactttgatgagacgtcatgcgtattcactccagaggggacagtttgcagccagaatgcattgtacccaatgagcccattgcttcaggaatgtttcagggggtcaacaaaatcgtgcgccagaaccctagtttcaggtaccataagtaatagatttatcctatcaaaaggaaacctgatcgcgaattgtgcgtcagttttgtgcaaatgttacacaacggagacagtcatcagccaagatcctgacaaactactaactgttgtagcatccgacaagtgtcctgtagttgaggtggatggagtgacaatacaggtcggcagtcgagagtatccggattctgtatacttacacaaaatagacttaggcccagccatctccctagaaaaactagatgtaggtaccaatttaggcaatgcagttacaagactggagaatgcaaaggagctcctagatgcatcagaccaaatactgaagactgtcaaaggggcaccttttggtgggaacatgtacatagcactggcagcttgcattggagtatccctagggcttgtcacattaatatgctgctgtaaggggaggtgtaagaacaaggaaatccctatctccaaaatcaacccagggctcaaacccgatctgaccgggacctcaaagtcgtacgtgagatcactgtagccagaatcacctgaatcatctggcatcacacacatacatgtacgacacaagcagtctgaggacgcaggaaacccagcccccgatcacccacctgaccctactccacgctccactacacattagtcattaaacaaaacttaggacgaaaggtcaatcaccatgtccgcacaaagggaaaggatcaatgccttctacaaagacaatcctcacaataagaaccatagggtaatcctggatagagaacgcttgatcattgaaagaccctacatcttgcttggagccctgctggtaatgttcctgagtctaatcggactgctggccattgcagggatcaggcttcaccgggccaccgttggaacttcagagatccagagtcggctgaataccaatattaagttgaccgaatctattgatcaccagactaaggatgtcttaactcccctctttaaaataattggcgatgaagtcggcatcaggattccacagaaattcagtgatcttgtcaagttcatctccgacaagattaaattcctcaaccccgatagagagtatgatttcagggatctccggtggtgcatgagtccccccgagagagtcaaaattaattttgatcagttttgtgagtacaaagctgcggttaattcaattgaacatatatttgagtcaccgctcaacaagtcaaaaaagctgcaatcattgactctcgggcccggaacaagttgtctaggcaggacagtaacaagagctcatttctcagaacttacactgaccttaatggacttggatctagagatgaagcacaacgtgtcctcagtgtttaccgtagttgaagaggggttattcggaagaacatataccgtctggagatccgatgccagggatccgagcaccgatctaggtatcggccattttttaagagtcttcgagattggactggtaagagatctcgggctgggtccccccgtttttcatatgactaactatctcacagtgaacatgagtgatgtctatcggagatgtcttttagcggtaggggagttgaagttgacagccctatgcacctcatctgagactgtaacactgagtgagagaggagttccaaagagggagcctcttgtagttgtgatacttaacctggctggaccaactctagggggcgaactatacagtatcttgcctacctctgatctcatggtggagaaactctatttatcttcacatagagggatcatcaaggatgacgaggccaattgggtagtgccgtctaccgatgttcgtgatcttcagaacaaaggtgagtgtctagtggaagcatgcaagactcgacctccttcattttgcaatggcacaggatcaggcccgtggtcagaggggagaatccctgcttacggggtgatcagggtcagtcttgacttagctagtgacccggatgtagttatcacttcagtgtttggcccactgattcctcacctatccggcatggatctttacaacaacccgttttcaagagctgtatggttggctgtaccaccttacgagcagtcatttctaggaatgataaatacaattggattccctaacagaacagaggttatgccgcacattttgaccacagagatcagaggccctcggggtcattgccatgtccccatagaattgtcccgcagggttgatgacgatatcaagatcgggtccaacatggtcatattgccgacgatggacctgagatatattacagccacttatgatgtttccaggagcgagcatgcaatcgtgtactatatctatgacacaggtcgctcatcatcttacttctacccagttcgactgaatttcaaaggcaatcctctctctctgaggatagagtgtttcccttggcgtcataaggtgtggtgctaccatgattgtcttatatacaacaccataacagatgaagaggtccatatgagagggctgaccggtatagaggtaacatgcaatccagtctgagcagagctacgaccgtcactcaagcagtcccttgagtcgccaccgagtccaagcagcacagcctgggactctcaacagcacagcccagctaacagtgttataaaaaacttaggagccagggttgtaggggccatggactccctatcagtcaatcaggtcttgtaccctgaggttcatctagacagccctattgtcacaaacaaactagttgccatccttgagtactcgggtatcgaccataactatgttcttgaagaccagacccttgtcaagaatattaggtatagactggggtgcggtttttcaaatcaaatgatcatcaataacaggggggtgggtgaaacagtcaattctaaacttaaaagttatccccataatcgtcatatcatatacccggattgcaataaggagttgttttgtatcaaagatagctgcatatctaagaagctctcggagctattcaagaagggtaattccttgtactctaagataagtcaccaggtactggactgtcttaagagagtcaatgggaaattaggcctgggcacagatcttacccatggtctgaaggagggtatccttgacctagggttgcacatgcatagctctcaatggttcgagactttcctgttctggttcactatcaagacagagatgagatcaatgatcaaagaacagtcccatatatgccacaagaggagatataacccgatttttgtatcaggggatgcattcgaggtgctcgtatcacgagacctcgttgtgataattgataagaatacccagtatgtcttctacttaacatttgagctggtccttatgtattgtgatgtcatagagggcagacttatgacggagacagccatggtcatagaccagagatattcagaacttctaagccgggttagatacctgtgggatcttattgatgggttcttcccaacactaggcaataccacctaccaaattgttgctctgcttgaaccattatcgttggcttatcttcaacttcaggatgtcacgctggagttaagaggtgcttttttagaccattgcttcaaagaactctatgagatactggagcattgcggcattgacacagaaggcacctataattccatcactgaaggattggattacgtattcatcacccacgatatacatctaactggggagattttttcatttttccggagtttcggacacccccgtctagaagcagtcaccgccgcagaaaatgtcaggaaacatatgaatcaaccgaaggtaatcagttatgagactatgatgaaaggacatgcggtattctgcgggataatcatcaatggttttagagaccgacatggcggcagctggccccctgttgcattgccagagcatgcttctgctgcgatccggaatgcgcaagcatcaggtgagggactgacccatgacctgtgtatagacaactggaaatcctttgttggattcaaatttggctgctttatgccactcagcctagacagtgatttgaccatgtatctcaaagacaaggcattggctgcactgaagaatgagtgggattcagtttacccgaaagaatacctccgttataacccacctagagggacagagtcaaggcgactggtggaggtgttcctgaatgactccagcttcgatccttataacatgataatgtacgtggtgaatggctcctatcttaaagaccctgagtttaatctctcatacagcctaaaagagaaggagataaaggagacagggcggttgtttgccaaaatgacctataagatgcgggcctgtcaggtaattgctgagaatctgatatcgaatggtgttgggaagtacttccgagacaatgggatggcgaaggacgagcatgacctaacaaaagcccttcacactttggcagtctcaggtgttcccaagaacaacaaagataaccgccgaggtgggcctcccagaaggacaacaacccgagaggtgagatcaagccgggacaccagaacacaaaatagagacaaggtccaagggggacctatgtacaactatttgcaatgccaaccaatcagccctgatcaaggtgagtcatacgagactgttagtgcatttatcactgctgaccttaagaagtattgcctgaattggagatacgagacaatcagcatatttgcacagaggctgaatgaaatatatggactaccatccttctttcaatggttgcacaggatattggaaaaatccgtactctacgtcagtgacccacattgccctcccgatctagataatcatatccctctggacagtgtccctaatgcccaaatattcattaagtacccaatgggtggaatagaaggttattgccaaaaactatggacaatcagtactataccatacttgtatctggcagcctatgagagcggagtaagaatcgcctcactggtgcagggtgacaatcagacaatcgcagtgacaaaaagagttccaagttcttggccttattcactaaaaaagagggaggcatccaaagccgctcaaaattacttcgtagtcttaaggcaaagattgcacgatgtaggtcatcacttgaaggctaatgaaaccatagtatcttctcacttttttgtatattccaaagggatttattatgacggcctgctagtctcacaatcactaaagagcatcgccagatgtgtcttctggtccgagactatagtggatgaaaccagagcggcttgcagcaatatcgcaacaactgtcgctaagagtatagagaggggttatgataggtaccttgcatattctttgaatatcctcaagattttccaacagatccttatatcccttaacttcactattaacacaacaatgactcaggatgtcgtggcaccgatcgtcgagaacggtgatttgctgataaggatggcactcttgccagcacccatcggaggtctaaattatcttaacatgagcaggctattcgtgagaaatatcggcgacccggtcacctcctctatagccgacctgaagaggatgatagatgccgggctcatgccagaagaaacattgcatcaagtgatgacccagaccccgggagaatcatcctaccttgattgggcaagtgacccttattctgccaacctaccctgcgtacagagtataactcgtcttctcaaaaacatcactgcacgatatattttaatcagcagcccaaatccgatgctgaaaggattgtttcatgaggggagtagagatgaagacgaggagcttgcaagtttcctaatggatcggcatataattgttccaagagctgcacatgaaatcttagaccatagtataaccggggcaagagaggctatagccgggatgttggacaccaccaagggtctgattagaacaagtatgaaacggggtggcctcacccctcgagtcttagcccgcctttccaattatgattatgaacaattccgatccgggataacactattgacaaagaaagggcagtgttatctcattgacaaggattcgtgctcggtgcagctcgctatagctctgaggagccacatgtgggctaggttggctcgcgggagaccaatctatgggttggaggtgcccgatatattagaatcgatgaacggctaccttatcaaacgccacgagtcttgtgctatctgtgaaacgggctcaagtcactacggatggtttttcgtccctgcagggtgccagcttgacgatgtatctagagagacttcggctctccgtgtaccttatgtcggatcaacaactgaggaaaggactgacatgaaacttgcatttgttagatctccaagtagatccctcaaatcagcagttagaattgcaacagtttactcatgggcctacggggatgatgagaagtcatggagtgaagcctggatgctagctaggcagagagccaatatcaccttagatgaattgagaatgatcactccagtctctacatccaccaacctagcccaccggttgagggatcggagcacccaggtgaaatactcggggacatcccttgtgagggttgcaagatacacaaccatctccaatgataacttgtcatttgtgatatctgagaagaaagtagataccaacttcatttatcagcaagggatgttgcttggtctcgggatccttgagaacctctttaggctagaggccaccaccggagtatccaacacagtgctacacctgcacgtggaaacagaatgttgtgttgtacctatggtggatcacccaaggataccgagtctccgtaatataaaagttacggatgagctatgcacaaaccctttgatctatgataggtctcccatcatagagcacgatgcaacccgactatactcacagagccacaggagacatttggtggagtttgttacctggtccacaagccagctttaccatatactggctaaatctacagcaatgtccatgattgaattgatcacgagattcgagaaagatcacatgaatgaaatagccgccctgattggcgatgacgacatcaacagcttcatcacagaatttttgctagtggagcccagattatttatagtttaccttggtcagtgtgctgccatcaattgggcctttgatatacattatcatcggccctcgggcaagtaccagatgggggaactcctctactctctgctctcacggatgagcaaaggagtatataagatcttcaccaatgctttgagccaccccaaagtttacaagaaattttggcgaagtggtgtcattgagccgattcatggcccatccctggatacacagaatttacatgtcactgtttgtgacatgatatatgggtcctatgtcacctacctggatcttttgctgaatgatgagctagatgattacccgtatttgctctgcgagagtgatgaggatgtggtcacagacaggttcgacaacattcaagccaaacatctctgtgtattagccgatgtatattgcagctccaagaggtgtccttccataatcgggatgtctcctatagaaaaatgtaccattctcacacattacatcaagggagaatcagtacaatccccgtctggaacctcatggaacactgatccccttgtagtagatcattactcatgctctctgacctaccttcgccgaggttccatcaaacaaatcaggttgagggtggatcctgggtttgtattcgaggcgttgacagacgtcgactttaaacaacctcgcaaagctaagttagatatatcggtcgttgggttgactgatttctctcccccttgcgacaacgtcggtgattttctagggactatcaacacattgaggcacaatctgcctgtcaccggaaccggggtctcgaactatgaagtccacgcttatcgtagaattggcctgaattcatcagcatgttacaaagccgtagagatctccacgttaatcaagccatccctagaagtcggagagcatggattgttcttaggagaaggttccggttcaatgctggctgcgtacaaggaagttcttaaattagcaaattgttattacaacagcggggtaacagcggagggcagagccggacagagggaaatatctccctatccttcagagatgagcctagtagagaatcaaatggggatagagcggagtgttaaagtgctgttcaacggcaaacctgaagtaacttgggtggggaccaccgattgctacaagtacataatcagtaacatccaaacctctagtctgggtttcatacattcagatattgagacgctcccaaccaaagatgctgttgagaaattagaagaatttgcctctatcctatccttatccctgattttggggaaaatcggctctattacagttgtcaaaattatgcccattagcggggatttcacccaaggcttcatagcctatgccatccaatattttagggagagcctgcttgcctatccgagatatagtaactttatctcgactgagtgttaccttatcatgataggattaaaggccaatcgattgattaacccagaagccattaagcaaagcataatcagagcgggggtcaggactgcaccaggacttgtgagccatatattatcagagaaacagaaaggttgtattcaatcttttttgggtgatccctatatccaaggagacttcaataagcaccttaaatctctaacccctattgagaaaatcctggtaaattgtggtctctcgatcaatggcacaaaaatctgtagggatctaatccaccatgatatcgcctccggtccagacggtcttatgagctctacaattattttatatagggaattggctcatttcaaagataatataaggagtcagcacggaatgttccacccctacccagtactggccaatagccggcaacgtgaattaatccatcgaatagccaagaaattctgggggtatgtcttgctatattctgatgacccggcactaatcagacaaacaatcaagaacttgaaacggaatcatctaacctttgacttacatagtaacccgtttattaaagggctatccaaagctgagaaactgctggtgcggacgagttcactaagaagggaatggttgttcactctcgatacgaaagaagtgaaagagtggttcaaattggtaggttacagtgcactcatcagaggctaattggttatacatctgcccccttctcctccgccatgagaccccactgacgattcagaagattaaagaaaactacatattggataagtatctattcccagctttgtctggt

>KT860063/India/Tamil_Nadu/2014/2

accaaacaaagttgggtaaggatagatcttataataactatggactagcaaacttaggagtaaagatcctactgtcggggggaggaggaggagcaagacctctgattatggcgactctccttaaaagcttagcattgttcaaaaggaacaaagacaaagcgccgacggcatcaggttcaggaggggccatccgagggattaagaatgttatcatagtcccgattcccggagactcgtccatcactacccgttcaagactgctcgacaggcttgtcagattggccggagatcctgacatcaacgggtcaaagctgaccggcgtgatgatcagcatgttatctttgttcgtagagtcacccgggcagttgatacagcgaatcacagatgatccggatgttagtatccgccttgttgaggtagttcaaagtactaggtcccagtccgggttgacctttgcatcacgtggtgctgatttggataacgaggcagacatgtatttttcaactgaggggccctcgagtggaggtaagaaaaggatcaactggtttgagaacagagaaataatagacatagaggtgcaggatccagaagagttcaatatgttgttagcctccatactagcacaagtctggatccttctggccaaggctgtcacggcaccggatacggcagctgactcagaactgagaaggtgggttaaatacacacaacaaaggagagtgattggggaatttcgccttgacaaagggtggctggacgcagtccgcaacagaattgcagaagatctatcactccggcggttcatggtatctctcatacttgacatcaagaggacccccggcaacaagccaaggatcgcagaaatgatctgcgacattgacaactatattgtcgaagcaggactcgccagtttcatccttactatcaaatttggtatagaaaccatgtatcctgcattagggctccacgagtttgccggggaattgtccactattgagtccttgatgaacttgtatcaacagctaggagaggttgcaccctacatggtaattctagagaactcaattcagaacaagtttagtgcaggagcttatcccctcctctggagctatgcgatgggtgtcggagttgagctggagaactcaatggggggcttgaactttggcagatcatattttgaccctgcctattttcgtctcggacaggagatggtcagaagatctgcaggaaaggtcagctctgtgattgcggctgagctcggcatcacagcagaggaagctaaactagtctcggaaatagcctcgcaggctggggacgaaagaaccgctagaggaactgggcctcgacaggcgcaggtctccttcctacagcaccaaacaggagggggagagtcgtccgcaccagcgaccagagaaggggtcaaagctgcgatcccaaacggatccggagaaagggacagaaagcaaacacgcccagggaggcccagaggagagacccccggccaactgctcctggaattcatgccagatgatgaggtctcgcgagaatctggtcaaaaccctcgtgaggctcaaaggtcggccgaggcactcttcaggctgcaggccatggccaagattctggaggaccaggaggagggagaagacgacaatcaggtccacaacgacaaggatctcctcggctaagcagacgcaccctctgtcgaaatcagtgacgagacatctcccaccagtattataaaaaacttaggacccaggtccaagcaagcacacatcgacactccaaccagtcgagcggaggccaccgatggcagaagaacaggcataccatgtcaacaaggggctggaatgtatcaagtccctcaaagcctctcccccggatctatccaccattagagataccatcgcgagctggagagaggggcttagcccctcgggccgtgcaacaccgaaccctgatacgtccgagggagatcatcagaatatcaaccaatcatgctcaccagcaatcggaccaaacaaagtctacttgtctcctgaagataatctcggatttagagagatcactggcaacgactgtggggctgagctcggaggagtccagggaaaaggatccaactctcaagtacagcgttactatgtttatagccacgggggtgaagagattgaaggactcgaggatgctgactctctcgtggttcaagcagatcctccagttgctaacatattcaatggaggagaggatggatctgacgacagcgatgtggactctggcccagatgatcccagcagagatactctatatgaccggggacctgctgccggcaatgatgtcgctaggtccacagatgtcgaaaaactagaaggtgctgatattcaagaagttctcaactcccagaaaagcagaggaggaagattccaaggcgggaaaaccttacgagtcccggaagtacccgatgtcaagaactccggaccatcagcccaatcaattaaaaagggcacagacgggaactcagtctcatctggaacggtgacagagtgtttatcgataagtggtgcaacccaagctgtgccagagtcaagatgggagtcatcagagcaaaatgcgtctgtggggagtgtcctcaagtctgcgaagagtgcaaagacgatccaggggtcgacacaagaatctggtaccacagcatcaccgactcagcctaaagagaatgactccgagtatgagtatgaggatgacctatttacagagattcaggacatccgtgcaagcattgccaagatccatgatgacaacaagactatcctctcaaagcttgattctatactgttattgaaaggagaagtcgacactatcaagaaacaaatcagcaagcagaatataagtatatccaccattgagggccatctctccagtataatgatagccatcccgggctttgggaaggatatcaaggacccaacatccgaggttgagttgaacccagatttaagacctataataagccgtgactctggcagagctctcgcggaggtcctcaagaaacccgctgtggataggtctcagaaaactgaaaccaaagccaactccagctcaaagggtcagcttcttaaggatctccagctaaaacctgtcgacaagcaggcaagctctgcaatcgggtttgtcccatccgaccatgaatcatccagaaatgtcatccgctccataatcaagtcgagcaagctaaacattgatcacaaggactatcttctagatttactgaatgatgtgaaaggctcaaaggatcttaaggaattccacaagatgctaacagcaattctcgccaagcacccgtaacacatcccccagtcaccatctcatactcggctgaagacatcctttcaatcagactattacaaaaaacttaggagcaagggcaactgagcttcgcagacaagatgaccgagatctacgacttcgacaaatcagcatgggatgtcaaagggtcaattgcccgcatagaacccaccacctaccacgacggccgactgataccccaggtaagggtcatcgatcctggtctgggagacagaaaagatgagtgctttatgtacctgtttctcctgggagtgattgaggataacgaccccctgtctccccccgtcggaagaacctttggctctttacctctaggggtcggtaggtcaactgctaagccagaagaactactaagggaggccacagaactagacatagtggtgaggcgcactgcaggagtaaatgagaaactggtattttacaacaacactccgctgtccttgttaacgccctggaagaaagttctgacaaccggaagtgtgtttagcgctaaccaggtttgcaatgcagtcaacctagtcccgcttgatactccccagaggttcagggttgtgtacatgagcataactagattatcagacaatggttactatagtgtgcccaggagaatgctagagttccgctcagccaatgcagtagccttcaacatcttggttacactaagaattgagaatggcacaaaccctagaagatacatagtcggctcatgggagaattcagaggtcacatttatggtacacgtgggcaactttagaagaaagaagaacgaagtatactctgctgattactgcaaaatgaagattgaaaagatgggtttagtttttgccctgggaggaataggtggaacaagtctccatattagaagcaccgggaaaatgagcaaaaccctccatgcacagctggggttcaagaaaatcttatgttaccccctaatggatgttaatgaggatcttaaccgatatctctggcgggcagagtgccgaatagtcaaaatccaggccgtcttacagccatcagtgccccaagaattccgtgtctacgatgatgtcatcatcaacgatgaccaaggcttgttcaagatcctgtagttcatctgcaacattatgacacgggttgcaatcttgacatctctgtttcttctcccaaatgttgttgcgtgccagattcactggggcaatctatccaagatcgggattgtgggaacagggagtgccagctacaaggtgatgactaggccgagccaccagactctggttataaagttaatgccaaatataacagccatcgacaattgtacaaagtcagagattgcagagtacaagagattgctgatcacagtgttaaagcctgtagaggatgctctgtcggttataaccaagaatgtaagaccaattcaaaccctgacacctgggcgtagaacccgtcgttttgctggagctgttctggccggagtagcgcttggagttgcgacagccgctcagataactgcaggagtcgctcttcatcaatcattgatgaactcccaagcaattgagagtttaaaaaccagtcttgagaagtcgaatcaggcaatagaagaaatcagacttgcaaataaggagaccatactggcagtacagggcgtccaggattatatcaacaatgagcttgtcccttctgttcatagaatgtcatgcgagctggtaggccacaagctcggcctcaagctccttaggtactacaccgagatcctgtccatattcgggcccagccttcgagacccgatagctgccgaaatatcaatccaggcactcagttatgcattaggcggagacattaataaaatcctggacaagcttgggtatagcggtggggatttccttgccatcctagaaagcaagggaataaaggcccgggttacatatgtggacacaagagattactttataatgcttagcatcgcctacccaaccttatctgagatcaagggagtgatagttcacaagatagaagctataacatacaacattggggcacaggagtggtatactactatccctaaatatgtagccactcagggatatctgatatcgaactttgatgagacgtcatgcgtattcactccagaggggacagtttgcagccagaatgcattgtacccaatgagcccattgcttcaggaatgtttcagggggtcaacaaaatcgtgcgccagaaccctagtttcaggtaccataagtaatagatttatcctatcaaaaggaaacctgatcgcgaattgtgcgtcagttttgtgcaaatgttacacaacggagacagtcatcagccaagatcctgacaaactactaactgttgtagcatccgacaagtgtcctgtagttgaggtggatggagtgacaatacaggtcggcagtcgagagtatccggattctgtatacttacacaaaatagacttaggcccagccatctccctagaaaaactagatgtaggtaccaatttaggcaatgcagttacaagactggagaatgcaaaggagctcctagatgcatcagaccaaatactgaagactgtcaaaggggcaccttttggtgggaacatgtacatagcactggcagcttgcattggagtatccctagggcttgtcacattaatatgctgctgtaaggggaggtgtaagaacaaggaaatccctatctccaaaatcaacccagggctcaaacccgatctgaccgggacctcaaagtcgtacgtgagatcactgtagccagaatcacctgaatcatctggcatcacacacatacatgtacgacacaagcagtctgaggacgcaggaaacccagcccccgatcacccacctgaccctactccacgctccactacacattagtcattaaacaaaacttaggacgaaaggtcaatcaccatgtccgcacaaagggaaaggatcaatgccttctacaaagacaatcctcacaataagaaccatagggtaatcctggatagagaacgcttgatcattgaaagaccctacatcttgcttggagccctgctggtaatgttcctgagtctaatcggactgctggccattgcagggatcaggcttcaccgggccaccgttggaacttcagagatccagagtcggctgaataccaatattaagttgaccgaatctattgatcaccagactaaggatgtcttaactcccctctttaaaataattggcgatgaagtcggcatcaggattccacagaaattcagtgatcttgtcaagttcatctccgacaagattaaattcctcaaccccgatagagagtatgatttcagggatctccggtggtgcatgagtccccccgagagagtcaaaattaattttgatcagttttgtgagtacaaagctgcggttaattcaattgaacatatatttgagtcaccgctcaacaagtcaaaaaagctgcaatcattgactctcgggcccggaacaagttgtctaggcaggacagtaacaagagctcatttctcagaacttacactgaccttaatggacttggatctagagatgaagcacaacgtgtcctcagtgtttaccgtagttgaagaggggttattcggaagaacatataccgtctggagatccgatgccagggatccgagcaccgatctaggtatcggccattttttaagagtcttcgagattggactggtaagagatctcgggctgggtccccccgtttttcatatgactaactatctcacagtgaacatgagtgatgtctatcggagatgtcttttagcggtaggggagttgaagttgacagccctatgcacctcatctgagactgtaacactgagtgagagaggagttccaaagagggagcctcttgtagttgtgatacttaacctggctggaccaactctagggggcgaactatacagtatcttgcctacctctgatctcatggtggagaaactctatttatcttcacatagagggatcatcaaggatgacgaggccaattgggtagtgccgtctaccgatgttcgtgatcttcagaacaaaggtgagtgtctagtggaagcatgcaagactcgacctccttcattttgcaatggcacaggatcaggcccgtggtcagaggggagaatccctgcttacggggtgatcagggtcagtcttgacttagctagtgacccggatgtagttatcacttcagtgtttggcccactgattcctcacctatccggcatggatctttacaacaacccgttttcaagagctgtatggttggctgtaccaccttacgagcagtcatttctaggaatgataaatacaattggattccctaacagaacagaggttatgccgcacattttgaccacagagatcagaggccctcggggtcattgccatgtccccatagaattgtcccgcagggttgatgacgatatcaagatcgggtccaacatggtcatattgccgacgatggacctgagatatattacagccacttatgatgtttccaggagcgagcatgcaatcgtgtactatatctatgacacaggtcgctcatcatcttacttctacccagttcgactgaatttcaaaggcaatcctctctctctgaggatagagtgtttcccttggcgtcataaggtgtggtgctaccatgattgtcttatatacaacaccataacagatgaagaggtccatatgagagggctgaccggtatagaggtaacatgcaatccagtctgagcagagctacgaccgtcactcaagcagtcccttgagtcgccaccgagtccaagcagcacagcctgggactctcaacagcacagcccagctaacagtgttataaaaaacttaggagccagggttgtaggggccatggactccctatcagtcaatcaggtcttgtaccctgaggttcatctagacagccctattgtcacaaacaaactagttgccatccttgagtactcgggtatcgaccataactatgttcttgaagaccagacccttgtcaagaatattaggtatagactggggtgcggtttttcaaatcaaatgatcatcaataacaggggggtgggtgaaacagtcaattctaaacttaaaagttatccccataatcgtcatatcatatacccggattgcaataaggagttgttttgtatcaaagatagctgcatatctaagaagctctcggagctattcaagaagggtaattccttgtactctaagataagtcaccaggtactggactgtcttaagagagtcaatgggaaattaggcctgggcacagatcttacccatggtctgaaggagggtatccttgacctagggttgcacatgcatagctctcaatggttcgagactttcctgttctggttcactatcaagacagagatgagatcaatgatcaaagaacagtcccatatatgccacaagaggagatataacccgatttttgtatcaggggatgcattcgaggtgctcgtatcacgagacctcgttgtgataattgataagaatacccagtatgtcttctacttaacatttgagctggtccttatgtattgtgatgtcatagagggcagacttatgacggagacagccatggtcatagaccagagatattcagaacttctaagccgggttagatacctgtgggatcttattgatgggttcttcccaacactaggcaataccacctaccaaattgttgctctgcttgaaccattatcgttggcttatcttcaacttcaggatgtcacgctggagttaagaggtgcttttttagaccattgcttcaaagaactctatgagatactggagcattgcggcattgacacagaaggcacctataattccatcactgaaggattggattacgtattcatcacccacgatatacatctaactggggagattttttcatttttccggagtttcggacacccccgtctagaagcagtcaccgccgcagaaaatgtcaggaaacatatgaatcaaccgaaggtaatcagttatgagactatgatgaaaggacatgcggtattctgcgggataatcatcaatggttttagagaccgacatggcggcagctggccccctgttgcattgccagagcatgcttctgctgcgatccggaatgcgcaagcatcaggtgagggactgacccatgacctgtgtatagacaactggaaatcctttgttggattcaaatttggctgctttatgccactcagcctagacagtgatttgaccatgtatctcaaagacaaggcattggctgcactgaagaatgagtgggattcagtttacccgaaagaatacctccgttataacccacctagagggacagagtcaaggcgactggtggaggtgttcctgaatgactccagcttcgatccttataacatgataatgtacgtggtgaatggctcctatcttaaagaccctgagtttaatctctcatacagcctaaaagagaaggagataaaggagacagggcggttgtttgccaaaatgacctataagatgcgggcctgtcaggtaattgctgagaatctgatatcgaatggtgttgggaagtacttccgagacaatgggatggcgaaggacgagcatgacctaacaaaagcccttcacactttggcagtctcaggtgttcccaagaacaacaaagataaccgccgaggtgggcctcccagaaggacaacaacccgagaggtgagatcaagccgggacaccagaacacaaaatagagacaaggtccaagggggacctatgtacaactatttgcaatgccaaccaatcagccctgatcaaggtgagtcatacgagactgttagtgcatttatcactgctgaccttaagaagtattgcctgaattggagatacgagacaatcagcatatttgcacagaggctgaatgaaatatatggactaccatccttctttcaatggttgcacaggatattggaaaaatccgtactctacgtcagtgacccacattgccctcccgatctagataatcatatccctctggacagtgtccctaatgcccaaatattcattaagtacccaatgggtggaatagaaggttattgccaaaaactatggacaatcagtactataccatacttgtatctggcagcctatgagagcggagtaagaatcgcctcactggtgcagggtgacaatcagacaatcgcagtgacaaaaagagttccaagttcttggccttattcactaaaaaagagggaggcatccaaagccgctcaaaattacttcgtagtcttaaggcaaagattgcacgatgtaggtcatcacttgaaggctaatgaaaccatagtatcttctcacttttttgtatattccaaagggatttattatgacggcctgctagtctcacaatcactaaagagcatcgccagatgtgtcttctggtccgagactatagtggatgaaaccagagcggcttgcagcaatatcgcaacaactgtcgctaagagtatagagaggggttatgataggtaccttgcatattctttgaatatcctcaagattttccaacagatccttatatcccttaacttcactattaacacaacaatgactcaggatgtcgtggcaccgatcgtcgagaacggtgatttgctgataaggatggcactcttgccagcacccatcggaggtctaaattatcttaacatgagcaggctattcgtgagaaatatcggcgacccggtcacctcctctatagccgacctgaagaggatgatagatgccgggctcatgccagaagaaacattgcatcaagtgatgacccagaccccgggagaatcatcctaccttgattgggcaagtgacccttattctgccaacctaccctgcgtacagagtataactcgtcttctcaaaaacatcactgcacgatatattttaatcagcagcccaaatccgatgctgaaaggattgtttcatgaggggagtagagatgaagacgaggagcttgcaagtttcctaatggatcggcatataattgttccaagagctgcacatgaaatcttagaccatagtataaccggggcaagagaggctatagccgggatgttggacaccaccaagggtctgattagaacaagtatgaaacggggtggcctcacccctcgagtcttagcccgcctttccaattatgattatgaacaattccgatccgggataacactattgacaaagaaagggcagtgttatctcattgacaaggattcgtgctcggtgcagctcgctatagctctgaggagccacatgtgggctaggttggctcgcgggagaccaatctatgggttggaggtgcccgatatattagaatcgatgaacggctaccttatcaaacgccacgagtcttgtgctatctgtgaaacgggctcaagtcactacggatggtttttcgtccctgcagggtgccagcttgacgatgtatctagagagacttcggctctccgtgtaccttatgtcggatcaacaactgaggaaaggactgacatgaaacttgcatttgttagatctccaagtagatccctcaaatcagcagttagaattgcaacagtttactcatgggcctacggggatgatgagaagtcatggagtgaagcctggatgctagctaggcagagagccaatatcaccttagatgaattgagaatgatcactccagtctctacatccaccaacctagcccaccggttgagggatcggagcacccaggtgaaatactcggggacatcccttgtgagggttgcaagatacacaaccatctccaatgataacttgtcatttgtgatatctgagaagaaagtagataccaacttcatttatcagcaagggatgttgcttggtctcgggatccttgagaacctctttaggctagaggccaccaccggagtatccaacacagtgctacacctgcacgtggaaacagaatgttgtgttgtacctatggtggatcacccaaggataccgagtctccgtaatataaaagttacggatgagctatgcacaaaccctttgatctatgataggtctcccatcatagagcacgatgcaacccgactatactcacagagccacaggagacatttggtggagtttgttacctggtccacaagccagctttaccatatactggctaaatctacagcaatgtccatgattgaattgatcacgagattcgagaaagatcacatgaatgaaatagccgccctgattggcgatgacgacatcaacagcttcatcacagaatttttgctagtggagcccagattatttatagtttaccttggtcagtgtgctgccatcaattgggcctttgatatacattatcatcggccctcgggcaagtaccagatgggggaactcctctactctctgctctcacggatgagcaaaggagtatataagatcttcaccaatgctttgagccaccccaaagtttacaagaaattttggcgaagtggtgtcattgagccgattcatggcccatccctggatacacagaatttacatgtcactgtttgtgacatgatatatgggtcctatgtcacctacctggatcttttgctgaatgatgagctagatgattacccgtatttgctctgcgagagtgatgaggatgtggtcacagacaggttcgacaacattcaagccaaacatctctgtgtattagccgatgtatattgcagctccaagaggtgtccttccataatcgggatgtctcctatagaaaaatgtaccattctcacacattacatcaagggagaatcagtacaatccccgtctggaacctcatggaacactgatccccttgtagtagatcattactcatgctctctgacctaccttcgccgaggttccatcaaacaaatcaggttgagggtggatcctgggtttgtattcgaggcgttgacagacgtcgactttaaacaacctcgcaaagctaagttagatatatcggtcgttgggttgactgatttctctcccccttgcgacaacgtcggtgattttctagggactatcaacacattgaggcacaatctgcctgtcaccggaaccggggtctcgaactatgaagtccacgcttatcgtagaattggcctgaattcatcagcatgttacaaagccgtagagatctccacgttaatcaagccatccctagaagtcggagagcatggattgttcttaggagaaggttccggttcaatgctggctgcgtacaaggaagttcttaaattagcaaattgttattacaacagcggggtaacagcggagggcagagccggacagagggaaatatctccctatccttcagagatgagcctagtagagaatcaaatggggatagagcggagtgttaaagtgctgttcaacggcaaacctgaagtaacttgggtggggaccaccgattgctacaagtacataatcagtaacatccaaacctctagtctgggtttcatacattcagatattgagacgctcccaaccaaagatgctgttgagaaattagaagaatttgcctctatcctatccttatccctgattttggggaaaatcggctctattacagttgtcaaaattatgcccattagcggggatttcacccaaggcttcatagcctatgccatccaatattttagggagagcctgcttgcctatccgagatatagtaactttatctcgactgagtgttaccttatcatgataggattaaaggccaatcgattgattaacccagaagccattaagcaaagcataatcagagcgggggtcaggactgcaccaggacttgtgagccatatattatcagagaaacagaaaggttgtattcaatcttttttgggtgatccctatatccaaggagacttcaataagcaccttaaatctctaacccctattgagaaaatcctggtaaattgtggtctctcgatcaatggcacaaaaatctgtagggatctaatccaccatgatatcgcctccggtccagacggtcttatgagctctacaattattttatatagggaattggctcatttcaaagataatataaggagtcagcacggaatgttccacccctacccagtactggccaatagccggcaacgtgaattaatccatcgaatagccaagaaattctgggggtatgtcttgctatattctgatgacccggcactaatcagacaaacaatcaagaacttgaaacggaatcatctaacctttgacttacatagtaacccgtttattaaagggctatccaaagctgagaaactgctggtgcggacgagttcactaagaagggaatggttgttcactctcgatacgaaagaagtgaaagagtggttcaaattggtaggttacagtgcactcatcagaggctaattggttatacatctgcccccttctcctccgccatgagaccccactgacgattcagaagattaaagaaaactacatattggataagtatctattcccagctttgtctggt

>KT860064/India/Tamil_Nadu/2015/3
[truncated: 1,640,728 more chars]
